# Interface-driven boost in the performance and stability of protonic ceramic electrochemical cells

**DOI:** 10.1038/s41467-026-77561-6

**Published:** 2026-09-17

**Authors:** Hyun Sik Yoo, Jongseo Lee, Soyeon Kim, Joon Gyu Kim, Sehee Bang, Yuhan Jung, Donguk Kim, Yue Wen, Min Hyung Bae, Bong Hyun Seo, Ji Won Suk, Wonyoung Lee

**Affiliations:** 1 School of Mechanical Engineering, Sungkyunkwan University, Suwon, Gyeonggi-do Republic of Korea; 2 Advanced Defense Science and Technology Research Institute, Agency for Defense Development, Daejeon, Republic of Korea; 3 Department of Smart Fab. Technology, Sungkyunkwan University, Suwon, Gyeonggi-do Republic of Korea; 4 SKKU Advanced Institute of Nanotechnology (SAINT), Sungkyunkwan University, Suwon, Gyeonggi-do Republic of Korea; 5 SKKU Institute of Energy Science and Technology (SIEST), Sungkyunkwan University, Suwon, Gyeonggi-do Republic of Korea; 6 SKKU National Lab for Intelligent Energy Solution Technology (SIEST), Sungkyunkwan University, Suwon, Gyeonggi-do Republic of Korea

**Keywords:** Fuel cells, Fuel cells, Electrocatalysis

## Abstract

Interfacial resistance remains a key challenge in developing high-performance and durable protonic ceramic electrochemical cells (PCECs), especially at low temperatures. Here, we present a systematic interface engineering strategy that quantitatively correlates interfacial contact coverage with electrochemical performance. A thin and dense PrBa_0.5_Sr_0.5_Co_1.5_Fe_0.5_O_5+δ_ interlayer is fabricated via low-cost and scalable precursor-based electrostatic spray deposition on a BaZr_0.4_Ce_0.4_Y_0.1_Yb_0.1_O_3-δ_ electrolyte, achieving a nearly complete contact coverage of 92.6 ± 0.6%. Equivalent circuit-based impedance modeling confirms that the improved contact coverage reduced both the interfacial ohmic and polarization resistances. The interface-engineered PCEC achieves a peak power density of 1.01 W/cm^2^ in fuel cell mode and a current density of 1.15 A/cm^2^ at 1.3 V in electrolysis mode at 500 °C. It also demonstrates improved interfacial mechanical integrity, preventing delamination under harsh thermal cycling, long-term operational stability, and fuel flexibility with direct ammonia and methane.

## Abstract (web summary)

Interfacial resistance remains a key challenge for high-performance and durable protonic ceramic electrochemical cells. Here, the authors report a quantitative interface engineering strategy to increase contact coverage, reducing interfacial resistance while improving performance and stability.

## Introduction

Protonic ceramic electrochemical cells (PCECs), based on proton-conducting oxides, are promising energy conversion devices, offering efficient power generation with fuel flexibility and green hydrogen production along with high energy conversion efficiencies^1–4^. One of their key advantages is their ability to operate below 500 °C, enabled by the high ionic conductivity and low activation energy of proton conduction, which is challenging for conventional solid oxide electrochemical cells (SOECs) based on oxygen ion conducting oxides, thereby mitigating a number of detrimental issues such as high system costs, low system sustainability, and limited material and process compatibility^5^.

Recent advances in proton-conducting electrolytes and triple conducting electrodes (proton, oxygen ion, and electron) have enabled high electrochemical performance at low temperatures^6–10^. However, these material improvements have not yet been fully realized at the cell level. For example, the measured ohmic resistance often exceeds theoretical predictions based on electrolyte conductivity and thickness (Supplementary Fig. 1), indicating substantial contributions from non-electrolyte sources. Similarly, the polarization resistance remains high, accounting for 70–90% of the total resistance below 500 °C, despite the high oxygen reduction reaction/oxygen evolution reaction (ORR/OER) activity of state-of-the-art electrode materials^5^. These limitations primarily originate from the electrode/electrolyte interface. Limited contact coverage, due to the abrupt structural change from a dense electrolyte to a porous electrode, inevitably restricts active reaction sites and increases interfacial resistance^11^. This issue is further exacerbated by the large mismatch in their thermal expansion coefficients (Supplementary Fig. 2). In addition to performance loss, inadequate contact coverage compromises mechanical integrity with poor interfacial bonding, leading to delamination, a key failure mode in ceramic electrochemical cells and a major barrier to long-term durability^12,13^. Therefore, the interface engineering has become a critical strategy for advancing high-performance and robust PCECs.

To address interface-associated challenges, a variety of structural engineering strategies have been developed, including physical and chemical treatments of the electrolyte surface (e.g., acid etching, laser ablating, and mechanical polishing) and incorporation of thin and dense interlayers between the electrode and electrolyte (e.g., vacuum-based deposition, drop-coating, and spray coating)^6,11,12,14–18^. These approaches have been effective in enhancing the interfacial density and achieving remarkable performance at low temperatures, underscoring the critical role of interface engineering. However, current characterization techniques, largely based on qualitative analyses, remain insufficient for quantitatively assessing key interfacial properties, including the contact coverage, interface reaction area, and interface resistance. To advance interface design, systematic design principles based on quantitative and direct characterization methods are highly required^19^. Furthermore, the vacuum-based deposition processes, commonly used for interlayer fabrication, present challenges in scalability and cost, limiting their commercial viability.

In this study, we present a systematic interface engineering strategy to develop high-performance and robust PCECs. To maximize contact coverage at the interface, which is a key design parameter of interfacial density, a PrBa_0.5_Sr_0.5_Co_1.5_Fe_0.5_O_5+δ_ (PBSCF) interlayer was fabricated by precursor-based electrostatic spray deposition (ESD) in a thickness of ~150 nm on a dense BaZr_0.4_Ce_0.4_Y_0.1_Yb_0.1_O_3-δ_ (BZCYYb) electrolyte, achieving a nearly complete contact coverage of 92.6 ± 0.6%. Equivalent circuit-based impedance modeling revealed a quantitative correlation between the contact coverage and the interface resistance. At 500 °C, Ni-BZCYYb fuel electrode-based single cells incorporating the optimized interlayer achieved a peak power density of 1.01 W/cm^2^ in fuel cell (FC) mode and a current density of 1.15 A/cm^2^ at 1.3 V in electrolysis cell (EC) mode, primarily due to reduced interfacial ohmic and polarization resistances. The interlayer also improved mechanical integrity, preventing delamination under thermal cycling. A long-term stability and fuel flexibility further demonstrated the practical applicability of this approach. Our results highlight an effective and practical interface engineering strategy, providing a promising pathway for further development of high-performance and robust ceramic electrochemical cells.

## Results and discussion

### Characterization of the PBSCF interlayer fabricated by the precursor-based ESD

We designed a precursor-based interlayer in a Ni-BZCYYb fuel electrode-supported single cell configuration, as shown in Supplementary Fig. 3. The desired characteristics for the ideal interlayer include a highly conformal structure to maximize the interfacial density for increasing both the contact coverage and the electrochemical reaction sites, while remaining sufficiently thin to minimize ion diffusion length^11,20^. To achieve these characteristics through effective and scalable nanoscale engineering, we employed ESD, which enables the precise control of nanoscale features by regulating atomized droplet dynamics through tailored process parameters (Supplementary Fig. 4)^14,21–23^. In particular, a precursor-based ESD was employed as a favorable method for producing desired conformal, dense, and thin films due to its finer structural resolution over powder-based ESD^14,20,24^. The characteristics of precursor-based ESD relevant to interlayer fabrication are compared with those of representative deposition methods in Supplementary Table 1. We optimized two key ESD process parameters of the substrate temperature and spray duration, as shown in Supplementary Figs. 5–8. The substrate temperature was optimized to suppress the formation of ring-like structures caused by capillary flow within the droplets (Supplementary Figs. 5 and 6), thereby facilitating the fabrication of the desired dense and uniform interlayer^25^. Subsequently, the spray duration was optimized to achieve a thin and dense interlayer on the electrolyte surface (Supplementary Figs. 7 and 8).

**Fig. 1:**
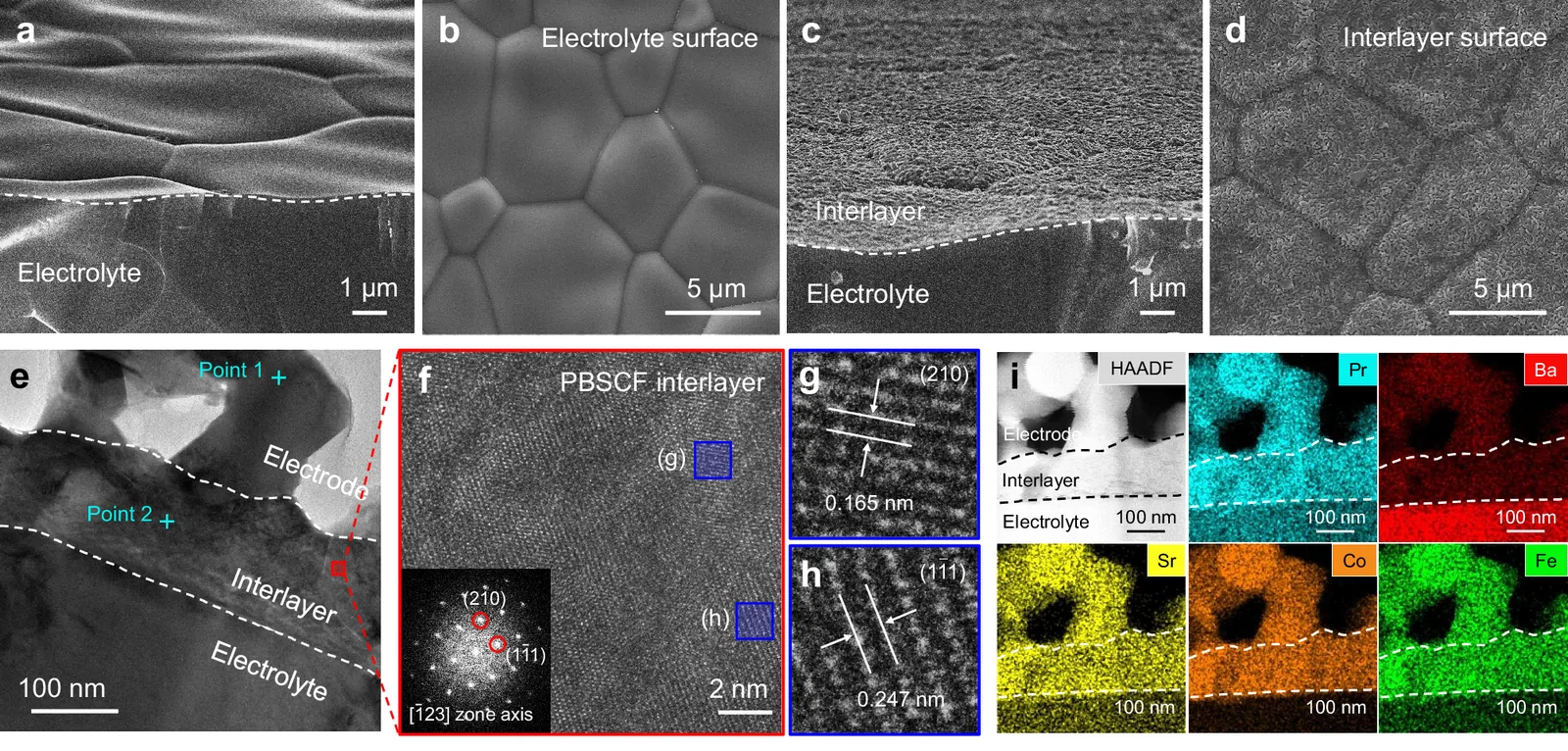
Characterization of the PBSCF interlayer fabricated by the precursor-based ESD. Tilted and top-view SEM images of **a**, **b** the pristine electrolyte surface and **c**, **d** the electrolyte surface coated with the precursor-based interlayer. **e** Cross-sectional HR-TEM images of the electrode/interlayer/electrolyte interface. **f** HR-TEM image of the interlayer, with the corresponding fast Fourier transform (FFT) pattern shown as an inset. **g**, **h** Magnified HR-TEM images of the two selected regions in (**f**). **i** High-angle annular dark field (HAADF)-STEM image of the electrode/interlayer/electrolyte interface and the corresponding EDS mapping of Pr, Ba, Sr, Co, and Fe.

Figure 1a–d present the SEM images of the pristine and modified electrolyte surface with the interlayer. The electrolyte surface with an average grain size of 10 ± 3 µm was uniformly covered by the thin and dense interlayer with an average grain size of 227 ± 39 nm and a thickness of 150 ± 11 nm, as confirmed by both SEM and atomic force microscopy (AFM) (Fig. 1 and Supplementary Fig. 9, respectively). The high-resolution transmission electron microscopy (HR-TEM) images in Fig. 1e–h reveal that the interlayer exhibits a double perovskite PBSCF phase (tetragonal, space group *P4/mmm*), verified by lattice spacings of ~1.65 Å for the (210) plane and ~2.47 Å for the (1–11) plane, respectively^26^. The energy dispersive X-ray spectroscopy (EDS) mapping in Fig. 1i presents a uniform distribution of Pr, Ba, Sr, Co, and Fe throughout the interlayer with no detectable secondary phases. The point EDS analyses of the bulk electrode and the interlayer, denoted as point 1 and point 2 in Fig. 1e, respectively, confirmed the identical atomic ratios (Supplementary Fig. 10 and Supplementary Table 2). The X-ray diffraction (XRD) patterns of the interlayer (Supplementary Fig. 11) further verified the formation of the double perovskite PBSCF phase without any secondary phases. Cross-sectional TEM images (Supplementary Fig. 12) revealed strong structural integration at both the electrolyte/interlayer interface and the electrode/interlayer interface, underscoring the effectiveness of the precursor-based ESD process in achieving robust interfacial bonding.

### Impedance model analysis: correlation between the contact coverage and the interface resistances

**Fig. 2:**
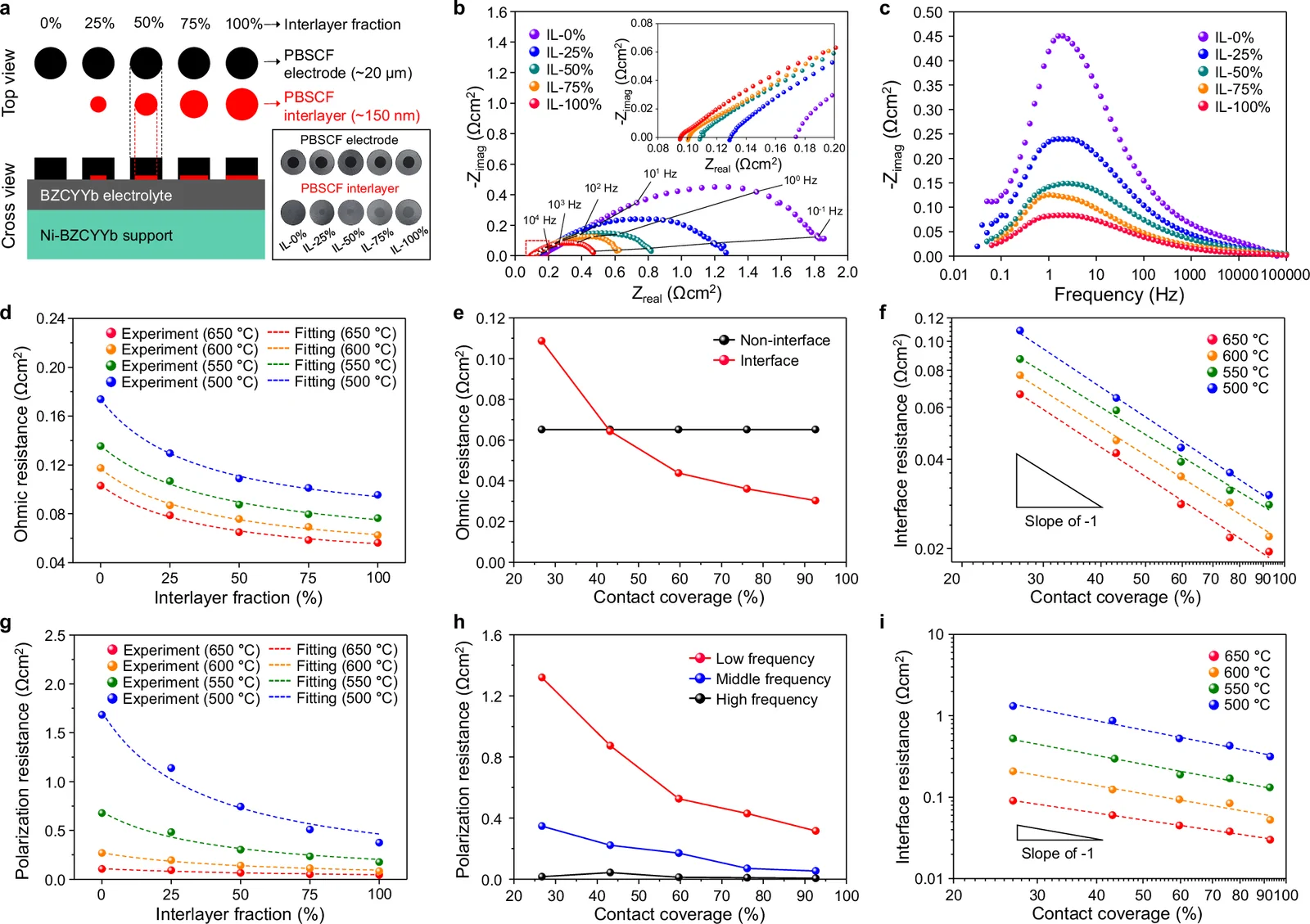
Impedance model analysis: Correlation between the contact coverage and the interface resistances. **a** A schematic illustration of the model sample configuration with optical images. **b** Nyquist plots (inset shows the magnified region of the red dashed box). **c** Bode plots of the model samples at 500 °C under OCV conditions. **d** Total ohmic resistance as a function of the interlayer fraction with corresponding fitting results in the temperature range of 650–500 °C. **e** Decoupled ohmic resistance of the interface and non-interface resistances at 500 °C as a function of the contact coverage. **f** Correlation between the interface component of the ohmic resistance and contact coverage in double logarithmic coordinates. **g** Total polarization resistance as a function of the interlayer fraction with corresponding fitting results in the temperature range of 650–500 °C. **h** Decoupled polarization resistance of the low-, middle-, and high-frequency resistances at 500 °C as a function of the contact coverage. **i** Correlation between the interface component of the polarization resistance and contact coverage in double logarithmic coordinates. Source data for Fig. 2 are provided as a Source Data file.

To quantitatively evaluate the correlation between the interfacial structure and electrochemical properties, we fabricated model cells with varying relative interlayer fractions of 0%, 25%, 50%, 75%, and 100% of the total electrode area, denoted as IL-0% to IL-100%, as shown in Fig. 2a. Electrochemical impedance spectroscopy (EIS) conducted in a Ni-BZCYYb fuel electrode-supported single cell configuration revealed a clear, monotonic reduction in both ohmic and polarization resistances with the increasing interlayer fraction (Fig. 2b, c), confirming a positive effect of the interlayer on the electrochemical performance.

To further evaluate the specific contributions of the interlayer to the interface-related components in ohmic resistance, we developed an equivalent circuit model comprising two components: non-interface resistance and interface resistance (Supplementary Fig. 13)^20^. The non-interface resistance (R_non-interface_), originating from the electrolyte, electrodes, and measurement setup, remained constant for all samples due to the identical cell configurations. R_non-interface_, which primarily originates from ionic conduction through the electrolyte, was independently determined from electrolyte thickness-dependent experiments (Supplementary Fig. 14 and Supplementary Table 3). The calculated electrolyte conductivity (~0.015 S/cm at 500 °C) and activation energy (~0.24 eV) agreed well with reported values for BZCYYb (~0.01–0.02 S/cm at 500 °C and ~0.23–0.33 eV)^6,27,28^, confirming the reliability of the measurements. In contrast, the interface resistance varied with the interlayer fraction and contact properties, including contact coverage and specific contact resistance. The contact coverages of the electrode/electrolyte interface in IL-0% and the interlayer/electrolyte interface in IL-100%, denoted as *θ*_1_ and *θ*_2_, respectively, were independently measured by structural analysis of the electrolyte surface after detaching the electrode and of the interlayer surface (Supplementary Figs. 15, 16 and Supplementary Tables 4, 5). In particular, the interlayer/electrolyte interface exhibited a higher contact coverage of 92.6 ± 0.6% (*θ*_2_), which is ~3.5 times higher than that of the electrode/electrolyte interface (26.7 ± 4.1%, *θ*_1_), consistent with previously reported values^11,20^. For the intermediate samples (IL-25%, IL-50%, and IL-75%), which were physically constructed as composite interfaces with defined area fractions of two configurations, the overall contact coverage was geometrically defined from *θ*_1_, *θ*_2_, and the interlayer fraction (Supplementary Fig. 17). After fixing three parameters (R_non-interface_, *θ*_1_, and *θ*_2_), fitting was conducted for the remaining two parameters, the specific contact resistances (R_contact1_ and R_contact2_). The model accurately reproduced the measured ohmic resistances for all interlayer fractions in the temperature range of 650–500 °C, with an R^2^ value of 0.997 ± 0.001 as shown in Fig. 2d. The detailed fitting results are listed in Supplementary Table 6. Fitting results revealed that the specific contact resistance, defined as the interface resistance normalized by the contact coverage, was nearly identical for both interface types: the electrode/electrolyte interface and the interlayer/electrolyte interface, consistent with the values independently derived from measured structural quantities and experimentally obtained resistance values^20^. Detailed calculations are provided in Supplementary Table 7. This similarity further substantiates that contact coverage is the primary factor governing interface resistance. Figure 2e represents the decoupled interface resistance from the total ohmic resistance as a function of the contact coverage. The interface resistance decreased by 72.1%, from 0.109–0.030 Ωcm^2^, as the contact coverage increased from 26.7–92.6%. Arrhenius plots (Supplementary Fig. 18) showed consistently lower interface resistances at all temperatures, while the activation energies remained nearly unchanged (0.190 ± 0.009 eV), indicating that the reduction in the interface resistance stems from the structural modifications, particularly the increased contact coverage, rather than changes in reaction kinetics^12,29,30^. Fig. 2f shows a clear correlation between the contact coverage and the interface resistance at all temperatures. The interface resistances exhibited linear trends on a double logarithmic scale with slopes close to −1, demonstrating an inverse proportionality to the contact coverage. In the present triple conducting oxide (TCO) interlayer system, these slopes indicate that the effective areas participating in ion transport and electrochemical reactions increase in proportion to the contact coverage. Therefore, the resulting scaling relationship provides quantitative information on how the relevant interfacial structural variable governs the interface-related resistance, while its dependence on that variable may change when transport characteristics alter the spatial extent of the effective transport or reaction region. The nearly parallel slopes observed at different temperatures suggest a consistent underlying mechanism driving the reduction in the interface resistance.

An equivalent circuit model was also developed for the polarization resistance, following the same approach as that for the ohmic resistance. As shown in Fig. 2g, the model accurately reproduced the measured polarization resistance for all interlayer fractions and temperatures (650–500 °C), with a high *R*^2^ value of 0.974 ± 0.015, indicating that the polarization resistance is similarly governed by the contact coverage. This relationship is consistent with the unique characteristics of PCECs, where the low-temperature operation and use of TCO electrodes make surface oxygen exchange reactions the rate-determining step (RDS) in polarization resistance^31–33^. This was clearly supported by the Bode plots (Fig. 2c and Supplementary Fig. 19), exhibiting a dominant impedance response at a specific frequency at approximately 10^−1^–10^1^ Hz, typical of surface exchange reactions^11,30^. Therefore, the interlayer contributes to performance enhancement not only by increasing the contact coverage with the underlying electrolyte, but also creating additional surface reaction sites above the interlayer (Supplementary Fig. 20). The 2D morphology of the interlayer, confirmed by a specific surface area of 1.066 ± 0.019 μm^2^/μm^2^ by AFM analysis (Supplementary Fig. 9), enables these surface reaction sites to scale proportionally with the contact coverage. This structural feature underpins the strong correlation between the contact coverage and polarization resistance, enabling a quantitative assessment of interfacial contribution to electrochemical performance. To decouple the components of polarization resistance and investigate their correlation with the contact coverage, we conducted Distribution of Relaxation Time (DRT) analysis. As shown in Supplementary Fig. 21, the polarization resistance was deconvoluted into three distinct frequency ranges: 1) a low-frequency range (<10^1^ Hz), associated with the surface charge transfer; 2) a middle-frequency range (10^1^–10^3^ Hz), corresponding to ion diffusion; and 3) a high-frequency range (>10^3^ Hz), representing the fuel electrode processes^11,34^. Figure 2h presents the decomposition of the polarization resistance at 500 °C as a function of the contact coverage (Supplementary Fig. 22). The low-frequency resistance exhibited the most pronounced reduction by 76%, from 1.32–0.32 Ωcm^2^, as the contact coverage increased from 26.7–92.6%. Because the porous powder electrode was identical for all model samples, this pronounced decrease is attributed primarily to the newly created surface reaction sites on the exposed interlayer surface. A moderate decrease in the middle-frequency resistance indicates reduced ion diffusion pathways, suggesting a shortened average ion diffusion length due to the thin interlayer (~150 nm)^35^. The high-frequency resistance remained nearly constant due to the identical fuel electrode configuration. Among them, the low-frequency resistance exhibited the most pronounced dependence on the contact coverage and was thus representative of the interface-related polarization resistance. Arrhenius plots (Supplementary Fig. 23) and their correlation with the contact coverage (Fig. 2i) revealed a consistent activation energy (1.04 ± 0.06 eV) and parallel trends in all contact coverages, confirming that increased contact coverage is the dominant factor reducing polarization resistance. However, in electrode systems with different transport characteristics, changes in the dominant reaction process and the associated distribution of electrochemically active sites may alter this scaling relationship. Evaluating such changes can identify the interfacial structural variable governing the polarization resistance and quantify its influence.

In summary, we quantified the contact coverage and its effect on the interface-related components in both ohmic and polarization resistances using impedance modeling. A clear correlation between increased contact coverage and reduced interfacial resistances indicated that both improvements originated from a common structural factor. This conclusion was further supported by the strong linear correlation with an *R*^2^ of ~0.978 observed in the normalized Arrhenius plots of all interface resistance values (Supplementary Fig. 24)^12^. Importantly, this interpretation does not imply that the contact coverage is the only interfacial variable governing electrochemical performance. Therefore, compositionally identical PBSCF was used for the electrode and interlayer to provide a controlled model system that minimizes variations in other material-related factors, including chemistry, microstructure, and defect concentration. This design is supported by TEM point EDS analysis confirming similar chemical composition (Supplementary Fig. 10 and Supplementary Table 2), AFM showing comparable surface roughness (Supplementary Fig. 9), XRD indicating similar crystallite sizes (Supplementary Fig. 11), XPS O 1*s* analysis showing comparable oxygen vacancy-related features (Supplementary Fig. 25 and Supplementary Table 8), and the nearly unchanged activation energies (Supplementary Figs. 18 and 23).

### Enhanced electrochemical performance with the precursor-based interlayer in both fuel cell and electrolysis cell modes

**Fig. 3:**
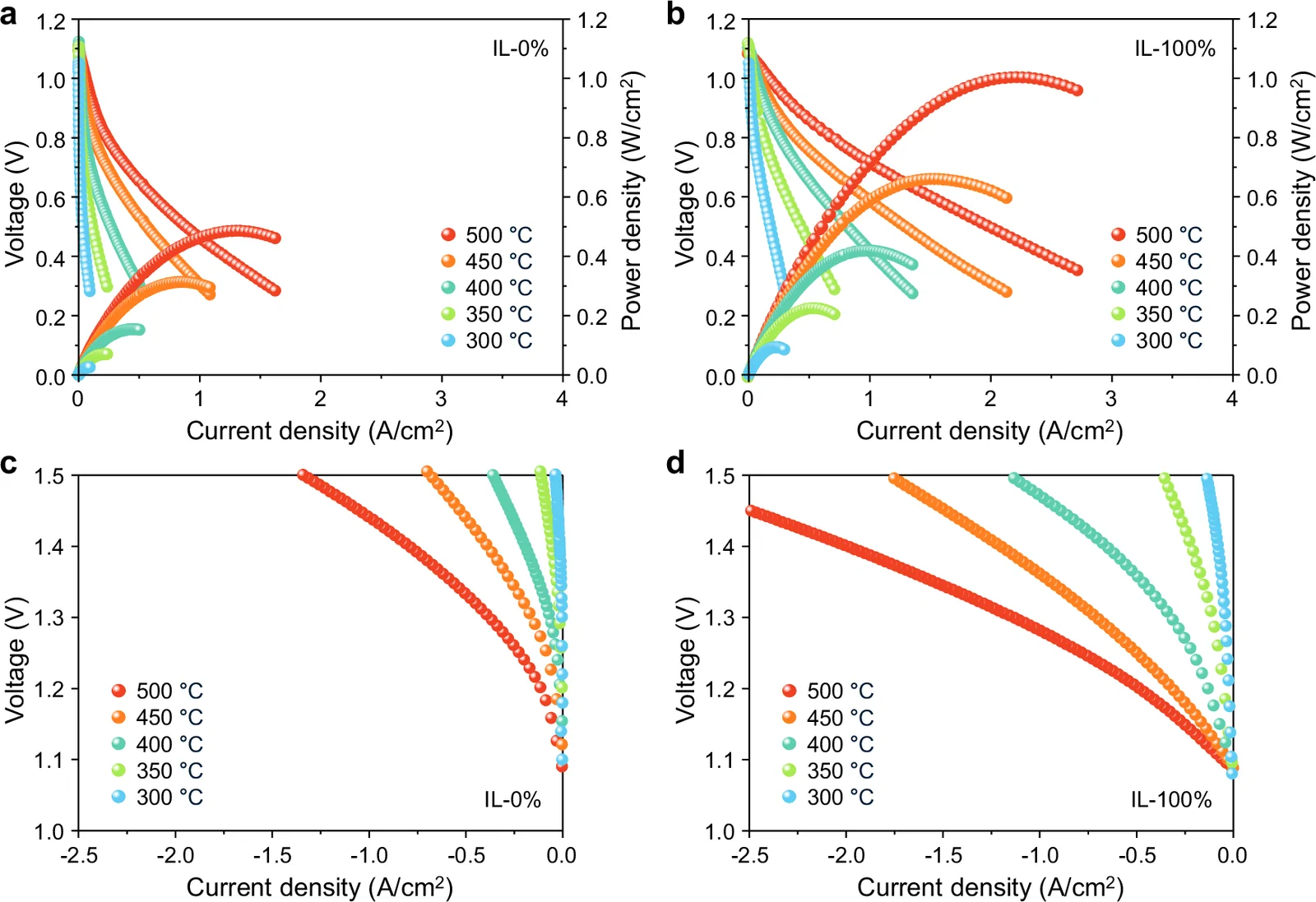
Enhanced electrochemical performance with the precursor-based interlayer in both fuel cell and electrolysis cell modes. I–V–P curves in the FC mode of **a** IL-0% and **b** IL-100% (500–300 °C), with humidified H_2_ at the fuel electrode and air at the air electrode. I–V curves in the EC mode of **c** IL-0% and **d** IL-100% (500–300 °C), with dry H_2_ at the fuel electrode and 80% air/20% H_2_O at the air electrode. Source data for Fig. 3 are provided as a Source Data file.

As a result of reduced interfacial resistances, IL-100% exhibited enhanced electrochemical performance. Despite using a relatively thick electrolyte (~10 μm), IL-100% exhibited a competitive ohmic resistance compared to previously reported PCECs, highlighting the critical role of the engineered contact coverage in minimizing the interface resistance (Supplementary Fig. 26). It also achieved the lowest polarization resistance among PCECs employing PBSCF air electrodes (Supplementary Fig. 27). These results demonstrate that the precise control of the interface structure can enable the full utilization of intrinsic properties and offer the opportunity to further lower polarization resistance by integrating advanced state-of-the-art electrode materials. In the FC mode, IL-100% achieved peak power densities of 1.01 W/cm^2^ at 500 °C and 0.42 W/cm^2^ at 400 °C, 2.1 times and 2.7 times higher than those of IL-0% (0.48 W/cm^2^ at 500 °C and 0.16 W/cm^2^ at 400 °C), respectively (Fig. 3a, b). A practical peak power density of 0.10 W/cm^2^ was also attained at 300 °C under pure air conditions. In the EC mode, IL-100% demonstrated current densities of 1.15 A/cm^2^ at 500 °C and 0.35 A/cm^2^ at 400 °C at 1.3 V under 20% steam, 2.8 times and 3.8 times higher than those of IL-0% (0.40 A/cm^2^ at 500 °C and 0.09 A/cm^2^ at 400 °C), respectively (Fig. 3c, d). These values are highly competitive among reported PCECs, especially those operating below 500 °C (Supplementary Table 9 and Supplementary Figs. 28, 29)^1,5–10,36–43^. The area scalability of the precursor-based ESD process was evaluated using a larger PCEC platform with an active area of 1 cm^2^ and a commercial Ni-YSZ substrate with a deposition area of 100 cm^2^. The larger area PCEC maintained the performance enhancement enabled by the interlayer, and structural and compositional analyses confirmed the uniform formation of a thin, dense, and conformal PBSCF interlayer over a 100 cm^2^ substrate (Supplementary Figs. 30–32). To examine whether the interface-engineering strategy can be extended to other electrode materials, the precursor-based interlayers were fabricated using additional air electrode materials, including Gd_0.3_Ca_2.7_Co_3.82_Cu_0.18_O_9-δ_ (GCCCO)^9^, Ba_0.5_Sr_0.5_Co_0.8_Fe_0.2_O_3-δ_ (BSCF)^44^, and La_0.6_Sr_0.4_CoO_3-δ_ (LSC)^45^ (Supplementary Figs. 33 and 34). The resulting interlayers exhibited morphologies comparable to those of the PBSCF interlayer, and their introduction consistently improved the electrochemical performance in all systems. These results indicate that the interlayer strategy is not limited to a specific electrode material and support its broad applicability. This generality is particularly important because, as PCECs increasingly adopt thinner electrolytes to enhance performance, the interfacial resistance becomes more critical due to its independence from the electrolyte thickness. In addition, at lower temperatures, polarization resistance, primarily governed by interfacial processes, becomes the major performance-limiting factor. Therefore, the interlayer-based approach offers a scalable and effective strategy to overcome the key challenges in advancing high-performance, low-temperature PCECs.

### Dynamic thermal cycling operation: reinforced interfacial adhesion

**Fig. 4:**
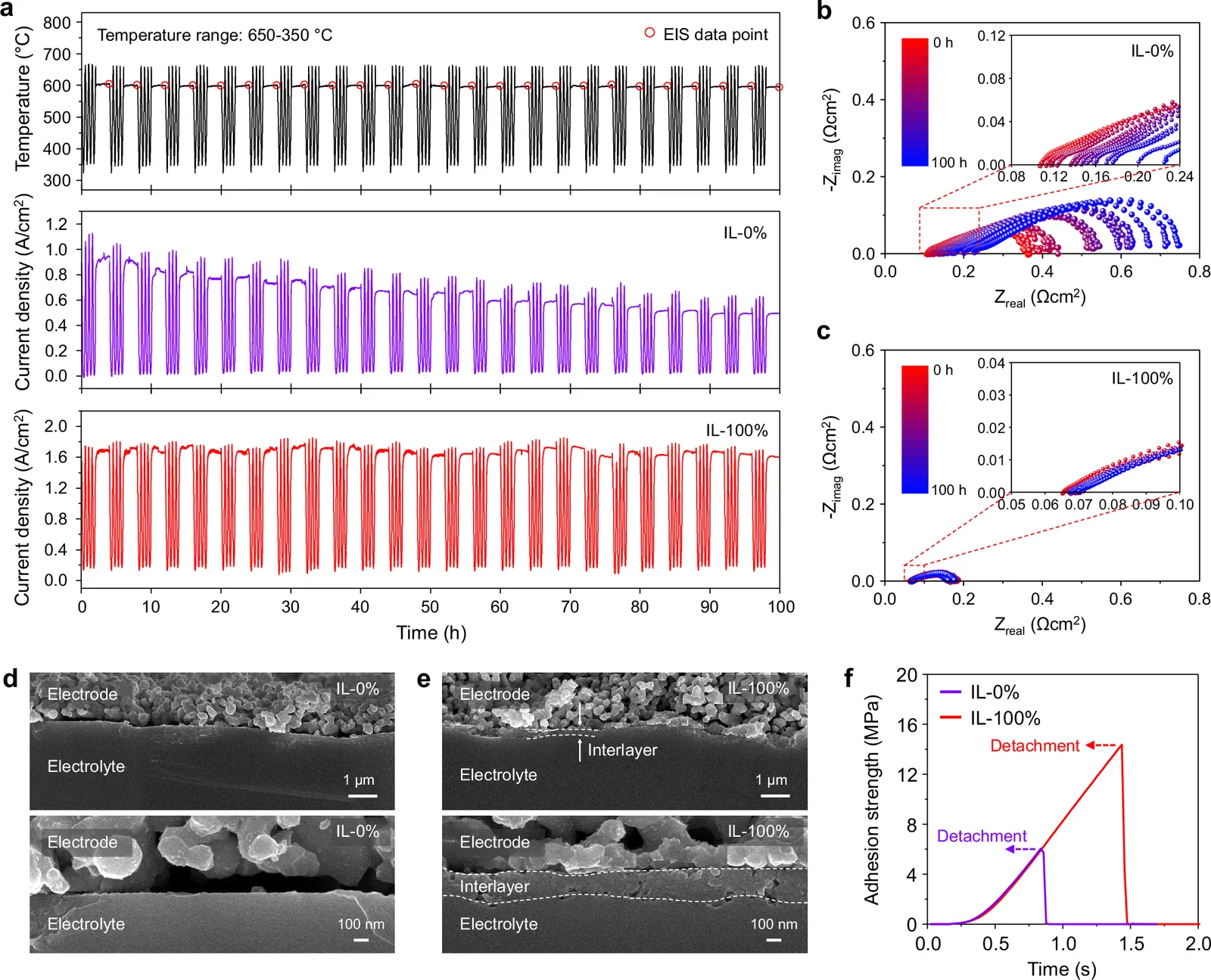
Dynamic thermal cycling operation: reinforced interfacial adhesion. **a** The thermal cycling temperature profile and corresponding output current density under a constant voltage of 0.8 V for IL-0% and IL-100%. Nyquist plots at 600 °C under OCV conditions during thermal cycling for **b** IL-0% and **c** IL-100%. Cross-sectional SEM images at the interface after thermal cycling for **d** IL-0% and **e** IL-100%. **f** Adhesion strengths at the interfaces of IL-0% and IL-100% using the mechanical pull-off test. Source data for Fig. 4 are provided as a Source Data file.

In addition to performance enhancement, the precursor-based interlayer improved the interfacial bonding strength by expanding the contact coverage, which is crucial for cell robustness. Poor interfacial bonding, often associated with limited contact coverage, is considered a primary cause of delamination and irreversible degradation during fabrication and operation^12,13^. By increasing the contact coverage, the interlayer acts as an “adhesive glue,” reinforcing interfacial integrity and enhancing mechanical robustness. To verify the improved interfacial bonding, we conducted an accelerated thermal cycling test on IL-0% and IL-100% under harsh conditions. As shown in Fig. 4a, 100 rapid thermal cycles were performed at 0.8 V with EIS measurements every 4 cycles after stabilization at 600 °C for 2 h (marked by red circles). IL-0% showed progressive deterioration and failed to recover its initial performance, while IL-100% maintained its initial performance with no detectable degradation. Notably, IL-100% operated under a broader current density range (0.2–1.8 A/cm^2^), which was 45% wider than IL-0% (0.0–1.1 A/cm^2^). Nyquist plots (Fig. 4b, c) revealed that IL-0% exhibited 113% and 106% increases in ohmic and polarization resistances after 100 h, respectively, whereas IL-100% maintained its initial resistances. Post-cycling DRT analysis showed that the degradation in IL-0% was dominated by a pronounced increase in the low-frequency resistance, which is associated with the loss of electrochemically active reaction area (Supplementary Fig. 35). Commonly reported degradation mechanisms in PCECs include cation segregation toward the electrode surface^46–49^, grain boundary weakening in the electrolyte^12,50,51^, and electrode-electrolyte delamination^12,45^. Given the identical cell configuration, the observed degradation in IL-0% is attributed to interfacial delamination. This was supported by cross-sectional SEM images after the thermal cycling, which revealed clear delamination in IL-0% and intact interfacial contact in IL-100% (Fig. 4d, e). Post-mortem analyses excluded other degradation mechanisms. The XRD and XPS analyses of the electrode surface showed no evidence of cation segregation or secondary phase formation (Supplementary Figs. 36 and 37). The cross-sectional SEM image of the electrolyte showed no evidence of intergranular cracking (Supplementary Fig. 38). The mechanical pull-off test further validated the enhanced adhesion strength (Fig. 4f); IL-100% showed ~14.4 MPa, which was ~2.4 times higher than that of IL-0% (~6.0 MPa). For the intermediate samples (IL-25%, IL-50%, and IL-75%), the adhesion strength increased gradually with increasing interlayer fraction (Supplementary Fig. 39). It should be noted, however, that this trend represents an experimentally observed correlation under the present conditions rather than a universal mechanical scaling law, given the complex fracture behavior associated with a multi-interface system. SEM images after the pull-off test revealed that the detachment occurred at the electrode/electrolyte interface in IL-0% and at the electrode/interlayer interface in IL-100% (Supplementary Fig. 40), implying even stronger bonding at the interlayer/electrolyte interface, likely due to co-sintering of compositionally identical materials. Raman spectroscopy confirmed the clear detachment of surfaces after the pull-off tests without the residual adhesive, verifying the reliability of the characterization method (Supplementary Fig. 41).

### High-performance and robust PCEC platform demonstrating a versatile energy conversion capability

**Fig. 5:**
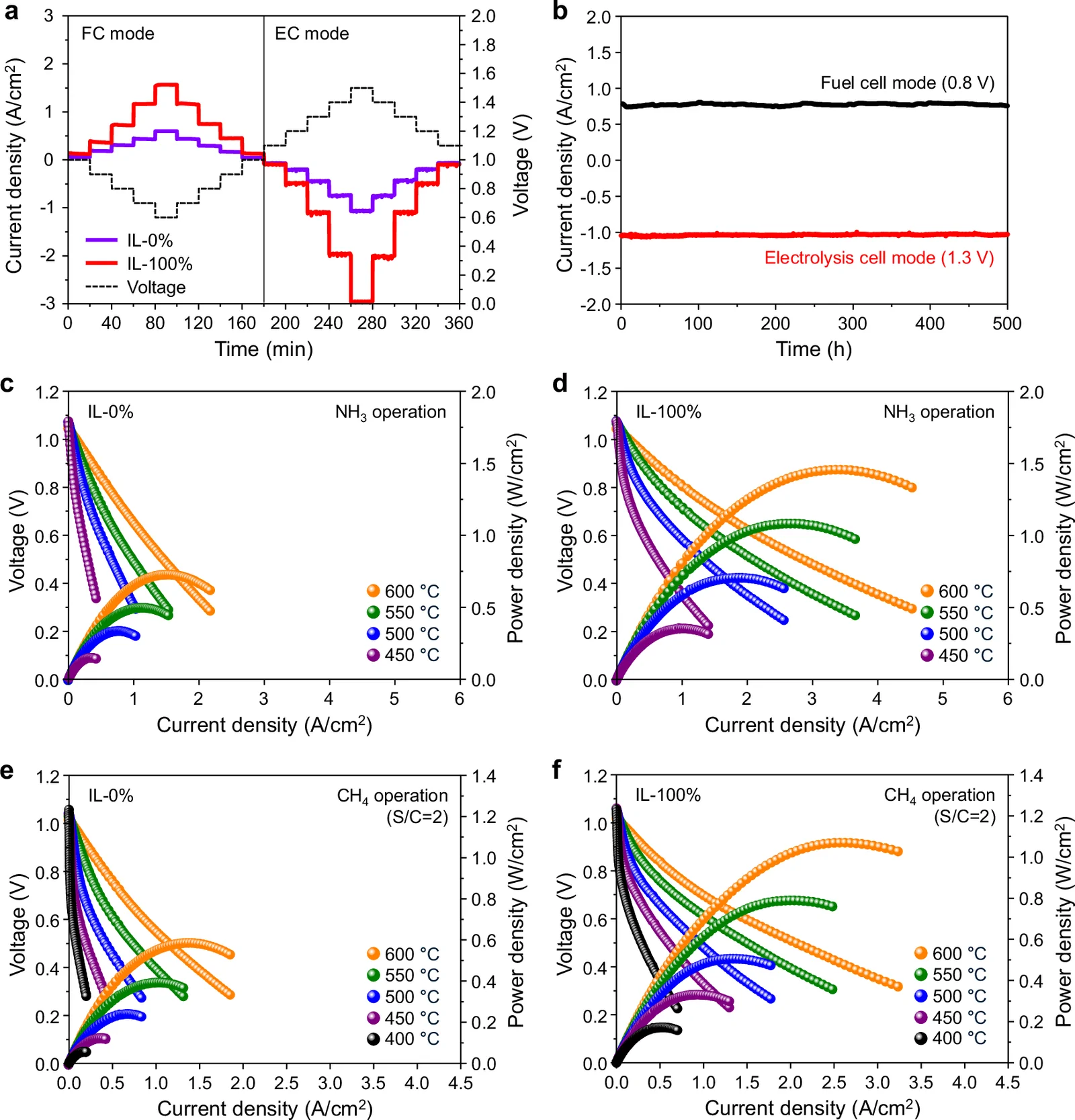
High-performance and robust PCEC platform demonstrating a versatile energy conversion capability. **a** Current densities under voltage ranges of 0.6–1.0 V (FC mode) and 1.1–1.5 V (EC mode) at 500 °C. **b** Long-term stability test at 500 °C under constant voltages of 0.8 V (FC mode) and 1.3 V (EC mode). I–V–P curves using ammonia fuel for **c** IL-0% and **d** IL-100% in the temperature range of 600–450 °C. I–V–P curves using methane fuel with a steam-to-carbon ratio of 2 for **e** IL-0% and **f** IL-100% in the temperature range of 600–400 °C. Source data for Fig. 5 are provided as a Source Data file.

We evaluated the versatility and robustness of our high-performance PCEC platform with a precursor-based interlayer under various operation modes and conditions. To assess its dynamic feasibility, we measured the current densities at 500 °C over a wide voltage range (Fig. 5a). IL-100% consistently showed higher power generation and hydrogen production compared to IL-0%. Stable operation was maintained even at high current densities up to ~3 A/cm^2^, underscoring the robustness of our PCEC and providing a safety margin against potential failure from unexpected current overshoots in real-world applications. The static long-term stability was evaluated at 500 °C under constant voltages of 0.8 V (FC mode) and 1.3 V (EC mode) for 500 h (Fig. 5b). IL-100% exhibited no performance degradation under both FC and EC modes. Post-mortem analyses revealed no discernable changes in the I–V curves or microstructures (Supplementary Figs. 42 and 43), validating the practical applicability of our interlayer for reliable energy conversion applications. Energy conversion efficiency (ECE) during the EC mode was calculated at 500 °C (Supplementary Figs. 44 and 45). At 0.5 A/cm^2^ and 500 °C, the ECE reached ~99.2% with a hydrogen production rate of 3.3 mL min^−1^ cm^−2^, validating its efficient hydrogen production capability.

The PCEC with the interlayer demonstrated fuel flexibility, operating effectively with alternative fuels such as ammonia and methane, both considered promising hydrogen carriers due to their higher volumetric energy density and convenient storage and transportation^39,52–55^. Without additional cracking or reforming catalysts, IL-100% achieved a peak power density of 0.76 W/cm^2^ with direct ammonia fuel at 500 °C, which was 2.2 times higher compared to that of IL-0% (0.34 W/cm^2^) (Fig. 5c, d). A similar enhancement was observed with methane: at a steam-to-carbon ratio (S/C) of 2 without additional reforming catalysts, IL-100% achieved a peak power density of 0.50 W/cm^2^ at 500 °C, which was 2.1 times higher compared to that of IL-0% (0.24 W/cm^2^) (Fig. 5e, f). The higher performance was maintained under lower steam conditions of S/C = 1 (Supplementary Fig. 46). In both ammonia and methane fuels, the ratio of peak power densities relative to hydrogen fuel remained similar for IL-0% and IL-100% (Supplementary Figs. 47 and 48), indicating that the observed performance enhancements stem from the same interfacial improvements that enabled the high performance with hydrogen. Consistently, EIS spectra obtained under ammonia and methane operation showed that IL-100% maintained lower ohmic and polarization resistances than IL-0%, with resistance reduction comparable to those observed under hydrogen operation (Supplementary Figs. 49 and 50). This confirms that the beneficial effect of the precursor-based interlayer remains effective under alternative fuel conditions. These results suggest that further performance improvements could be achieved through ammonia (or methane)-specific strategies to increase their conversion, especially considering the moderate ammonia and methane conversions of ~60% and ~35% at 500 °C, respectively (Supplementary Figs. 51 and 52). Nevertheless, the performance of direct-ammonia and methane PCECs developed in this study remains competitive compared to previously reported values (Supplementary Figs. 53 and 54)^2,5,39,53,55–62^.

We developed a quantitative and scalable interface engineering approach for high-performance and robust PCECs using a precursor-based PBSCF interlayer. Systematic impedance modeling revealed contact coverage as the key factor in determining interfacial resistances, enabling rational interface design. This approach demonstrated enhanced electrochemical performance and long-term stability, even under harsh thermal cycling. Our results provide a scientific insight for establishing design principles and scalable, low-cost fabrication methods to control interfacial properties for PCECs and other interface-sensitive electrochemical systems, offering promising pathways for sustainable energy technologies.

## Methods

### Fabrication of a single cell

A Ni-BZCYYb electrode-supported single cell was fabricated for the electrochemical analysis. NiO powder (Kojundo Chemical, 99.97%), BZCYYb powder (Kceracell, 95%), and poly methyl methacrylate pore former (Sunjin Beauty Science) were uniformly dispersed through ball-milling for 24 h at a weight ratio of 6:4:1 in ethanol with dibutyl phthalate (Sigma Aldrich), KD-6 (Croda), and polyvinyl butyral (PVB, Sigma Aldrich) as plasticizer, dispersant, and binder, respectively. The prepared NiO-BZCYYb powder was uniaxially pressed at 10 MPa and pre-sintered at 1000 °C for 3 h to obtain sufficient mechanical strength. The NiO-BZCYYb functional layer and the BZCYYb electrolyte were deposited through the spin-coating method. For the NiO-BZCYYb functional layer deposition, the slurry containing the NiO (6g) and BZCYYb (4g) powders in a weight ratio of 6:4 was prepared in isopropyl alcohol (20 ml) with a BYK-2012 (BYK; 0.2 ml) dispersant and an ethyl cellulose (Samchun Chemicals; 0.224 g) binder. The BZCYYb slurry was prepared following the same procedure as the NiO-BZCYYb functional layer slurry, except for the use of the NiO powder. After spin-coating, the sintering process was performed at 1500 °C for 5 h to densify the electrolyte. For IL-0%, PBSCF (Kceracell) powder was mixed with the ink vehicle (Fuel Cell Materials) at a 1:1 weight ratio and screen-printed onto the dense electrolyte. For IL-100%, PBSCF slurry was screen-printed onto the precursor-based interlayer, which had been deposited on the BZCYYb electrolyte surface via ESD. After deposition of the air electrode layer, all samples were sintered at 950 °C for 4 h. The cell area was 1 cm^2^, and the active area of the air electrode was 0.2 cm^2^.

### Deposition of the precursor-based interlayer

The precursor-based interlayer was deposited via ESD. The precursor solution was prepared by mixing stoichiometric amounts of Pr(NO_3_)_3_·6H_2_O (Sigma Aldrich, 99.9%), Ba(NO_3_)_2_ (Sigma Aldrich, 99%), Sr(NO_3_)_2_ (Sigma Aldrich, 95%), Co(NO_3_)_2_·6H_2_O (Sigma Aldrich, 98%), Fe(NO_3_)_3_·9H_2_O (Sigma Aldrich, 98%) and urea (Sigma Aldrich, 99.3%) in a propylene glycol solvent (Sigma Aldrich, 99%). The concentration of the chemical solution was 0.00125 M, and the urea-to-cation ratio was 4. A nozzle (Nano NC) was used to spray the precursor solution at a constant flow rate of 4 μL/min, and the ESD process was conducted at a distance of 4 cm between the nozzle and the substrate while applying a voltage of 5 kV. After deposition, the precursor-based interlayer was co-sintered at 950 °C for 4 h with the PBSCF electrode. For model experiments, masking was conducted using aluminum foil.

### Electrochemical performance measurements

Ceramabond 552 (Aremco) was utilized for gas sealing the materials. For the FC mode, humidified hydrogen (100 sccm) and dry air (200 sccm) were supplied to the fuel and air electrodes, respectively. For the EC mode, dry hydrogen (100 sccm) and a mixture of 20% steam and 80% air (200 sccm) were supplied to the fuel and air electrodes, respectively. For the ammonia operation, the ammonia flow rate was set at 66 sccm. For the methane operation, 64 sccm of mixed gases (S/C = 2: 25% CH_4_, 50% H_2_O, and 25% Ar; S/C = 1: 25% CH_4_, 25% H_2_O, and 50% Ar) were supplied to the fuel electrode^2^. To evaluate the faradaic efficiency in the EC mode, the fuel electrode gas was mixed with Ar (H_2_:Ar = 1:4), and gas chromatography (8890 GC, Agilent) was used to analyze H_2_ production. The Faradaic efficiency in the EC mode was calculated using Eq. (1), where ${n}_{{H}_{2},{measrued}}$ is the measured hydrogen production, I is the current, n=2, and F is Faraday’s constant. The energy conversion efficiency in the EC mode was calculated using Eq. (2).

1 $${Faradaic\; efficiency}\left(\%\right)=\frac{{\Delta n}_{{H}_{2},{measrued}}}{\Delta {n}_{{H}_{2},{theoretical}}}=\frac{{\Delta n}_{{H}_{2},{measrued}}}{I\times {\left(n\times F\right)}^{-1}}\times 100$$

2 $${Energy\; conversion\; efficiency}\left(\%\right)=\frac{\Delta {H}_{{LHV}}\times {n}_{{H}_{2},{measrued}}}{I\times V}\times 100$$

The ammonia and methane conversion rates were measured using Fourier transform infrared spectrometry (Nicolet^TM^ iS^TM^ 50 FTIR Spectrometer, Thermo Fisher Scientific) and gas chromatography (8890 GC, Agilent), respectively.

### Characterizations

Electrochemical characteristics, including current-voltage (I–V) measurements and electrochemical impedance spectroscopy (EIS) measurements, were conducted using a potentiostat (IviumStat.h, Ivium Technologies) in the frequency range of 0.1–10^6^ Hz with an AC amplitude of 50 mV. All measured currents and impedance data were normalized by the air electrode area. Scanning electron microscopy (SEM; JSM-IT800, JEOL), transmission electron microscopy (TEM; NEO ARM, JEOL), and atomic force microscopy (AFM; Multimode 8 AFM, Nanoscope V, Bruker) were used to analyze the structural properties. The crystal structures and the crystallite sizes of the precursor-based interlayer were examined using X-ray diffraction analysis (XRD; SmartLab, RIGAKU) with Cu K-alpha radiation (λ = 1.5406 Å) at room temperature (25 °C). The chemical properties were investigated using X-ray photoelectron spectroscopy (XPS; ESCA Lab 250 XPS spectrometer, VG Scientific Instruments) with a monochromatic Al K-alpha source and thermogravimetric analysis (TGA; TG/DTA7300, Hitachi). The mechanical pull-off tests were performed using a high-precision universal testing machine (QC-506 M2; Cometech)^20^, and the tests were carried out using a 2-kN load cell at a constant tensile loading rate of 10 mm min^−1^.

## Supplementary information

Supplementary Information

Transparent Peer Review file

## Source data

Source Data

## Data Availability

The data generated in this study are provided in the Supplementary Information/Source Data file. Source data are provided with this paper.
