# Supplementary material for: Interface-driven boost in the performance and stability of protonic ceramic electrochemical cells
Source: Nat Commun. 2026 Sep 17;17:10945. doi: 10.1038/s41467-026-77561-6 (PMC13680501; doi:10.1038/s41467-026-77561-6)
Supplement: Supplementary file 1 [file 41467_2026_77561_MOESM1_ESM.pdf]

Supplementary information

Interface-Driven Boost in the Performance and Stability of Protonic Ceramic Electrochemical Cells

Hyun Sik Yoo[1], Jongseo Lee[2], Soyeon Kim[1], Joon Gyu Kim[1], Sehee Bang[1], Yuhan Jung[1], Donguk Kim[1], Yue Wen[1], Min Hyung Bae[1], Bong Hyun Seo[1], Ji Won Suk[1, 3, 4], and Wonyoung Lee[1, 5]*

[1] School of Mechanical Engineering, Sungkyunkwan University, Suwon, Gyeonggi-do 16419, Republic of Korea

[2] Advanced Defense Science and Technology Research Institute, Agency for Defense Development, Daejeon 34060, Republic of Korea

[3] Department of Smart Fab. Technology, Sungkyunkwan University, Suwon, Gyeonggi-do 16419, Republic of Korea

[4] SKKU Advanced Institute of Nanotechnology (SAINT), Sungkyunkwan University, Suwon, Gyeonggi-do 16419, Republic of Korea

[5] SKKU Institute of Energy Science and Technology (SIEST), Sungkyunkwan University, Suwon, Gyeonggi-do 16419, Republic of Korea

* Corresponding author : leewy@skku.edu

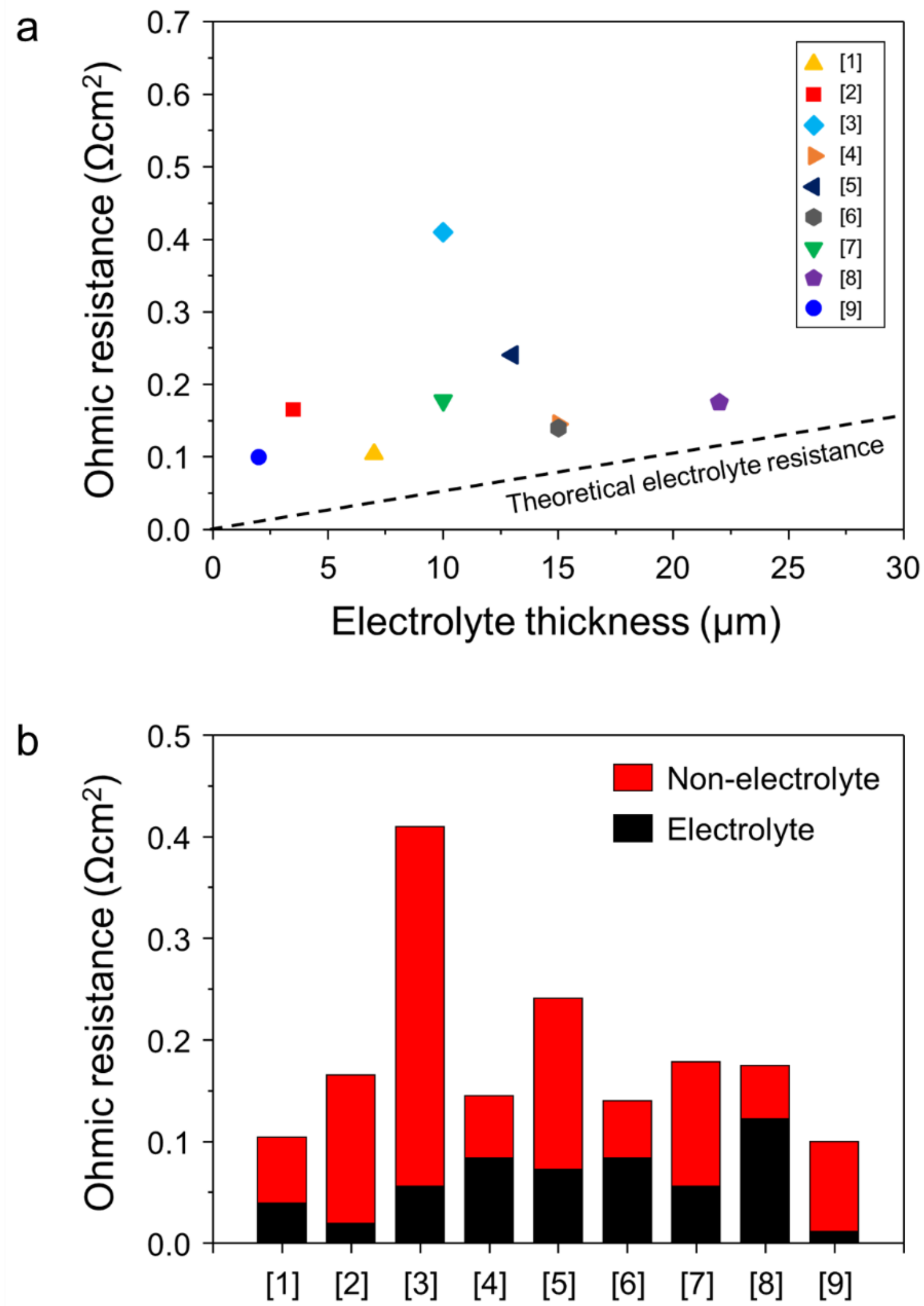

Supplementary Fig. 1. (a) Total ohmic resistance of previously reported PCECs as a function of the electrolyte thickness at 500 °C. The theoretical value was calculated based on electrolyte thickness, assuming a conductivity of 0.018 S/cm at 500 °C for all electrolyte compositions[1-9]. (b) Decoupled ohmic resistance of the theoretical electrolyte and non-electrolyte resistances at 500 °C. Source data for Supplementary Fig. 1 are provided as a Source Data file.

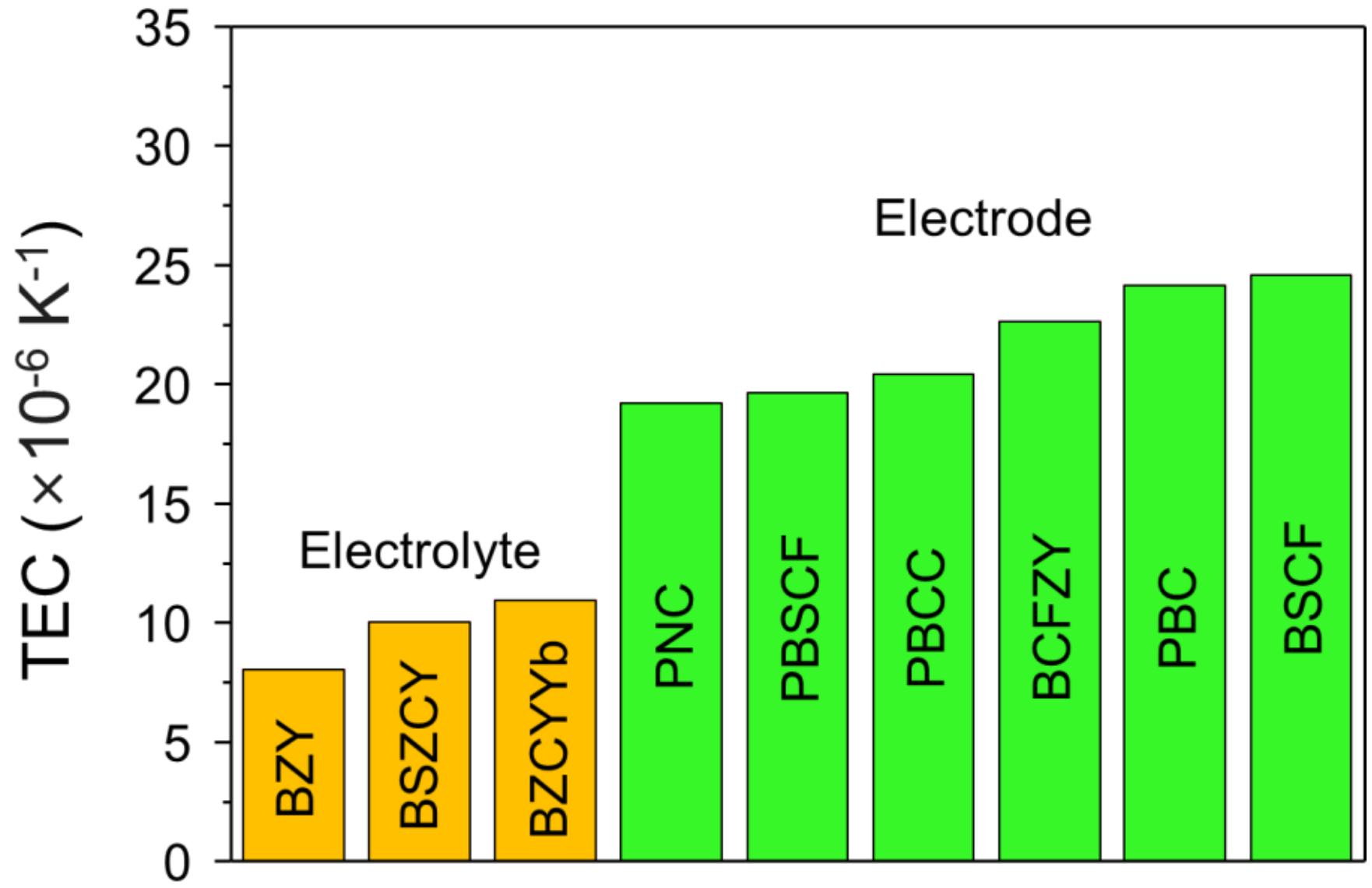

Supplementary Fig. 2. TEC comparison of the electrolyte and the electrode materials in PCECs[10-16]. Source data for Supplementary Fig. 2 are provided as a Source Data file.

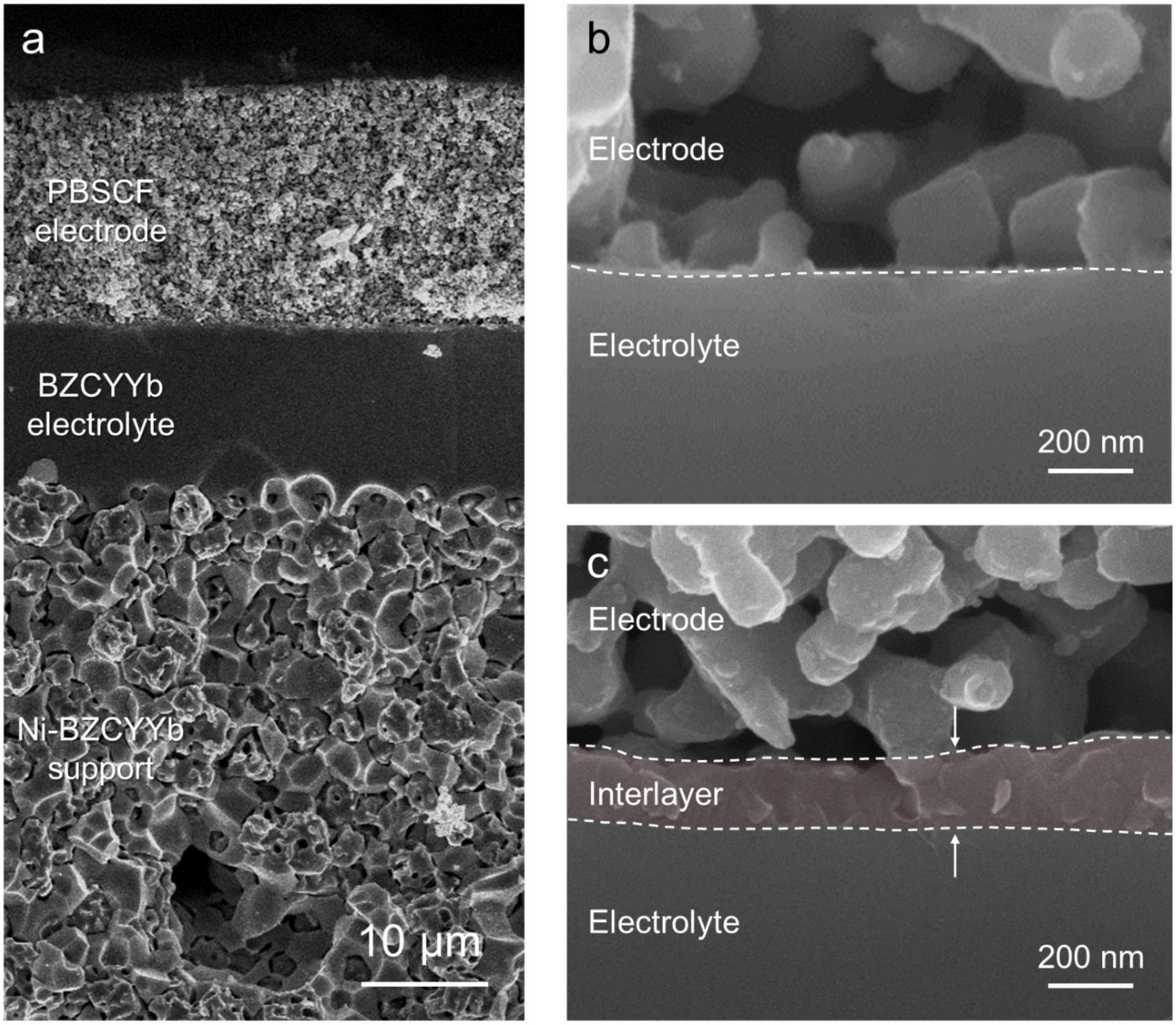

Supplementary Fig. 3. Cross-sectional SEM images of (a) a Ni-BZCYYb fuel electrode-supported single cell and the electrode/electrolyte interface (b) without and (c) with the precursor-based interlayer.

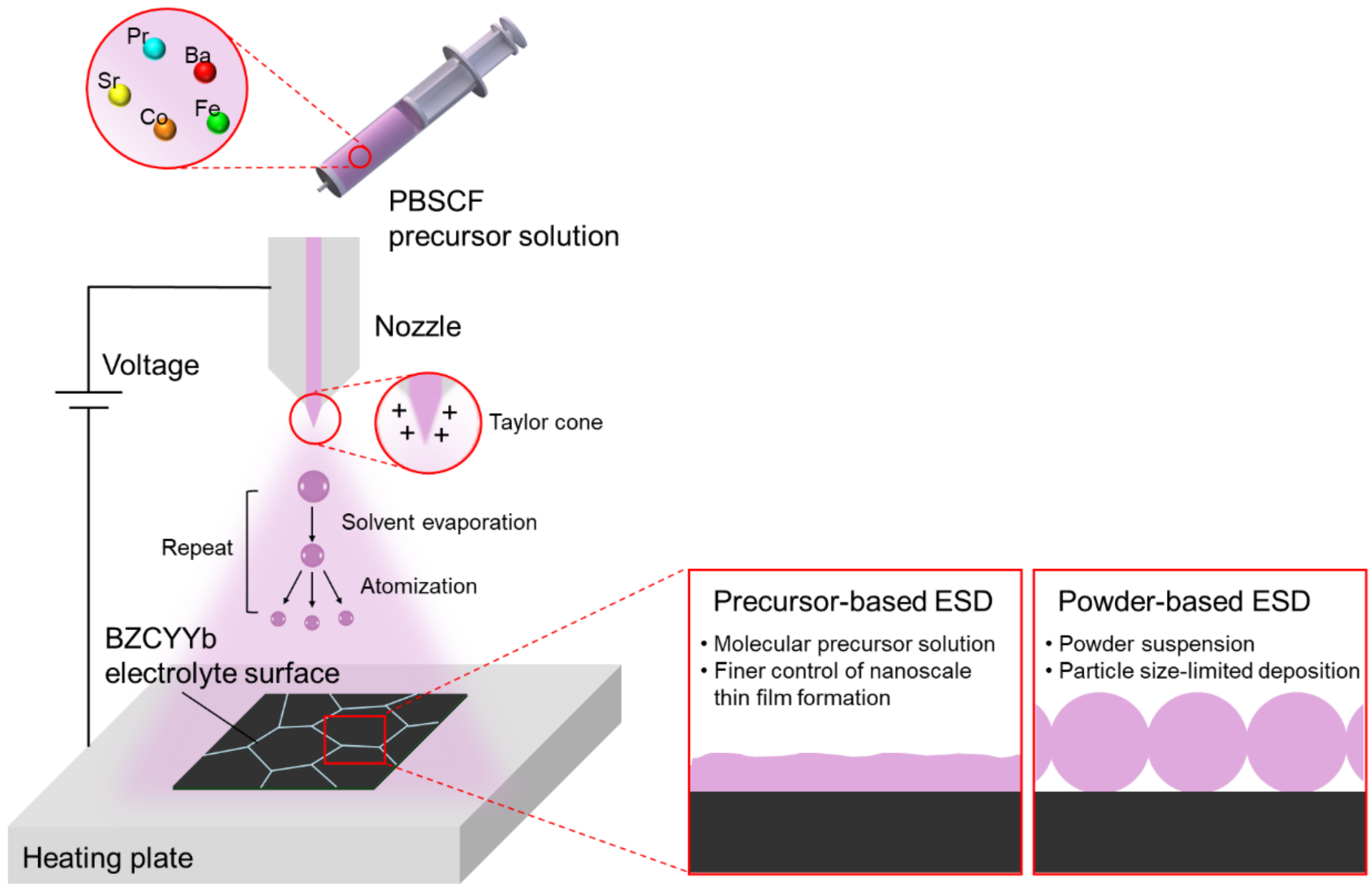

Supplementary Fig. 4. Operating principle of precursor-based electrostatic spray deposition.

Under an applied electric field, a liquid feedstock forms a stable Taylor cone forms at the nozzle tip and emitted as charged droplets. During flight toward the heated substrate, solvent evaporation reduces the droplet size and increases the solute concentration. Further solvent removal after the droplets reach the substrate forms a precursor-derived deposit on the electrolyte surface. In precursor-based ESD process, the feedstock is a homogeneous solution containing dissolved metal precursors rather than a suspension of preformed ceramic particles. Subsequent sintering converts the deposited species into a continuous crystalline oxide interlayer through thermal decomposition and crystallization. This distinction is important because the deposited layer is not formed from preformed ceramic particles, and its nanoscale thickness is therefore not directly constrained by the particle size of the feedstock. This enables finer control over the formation of continuous nanoscale thin films.

Supplementary Table 1. Qualitative comparison of representative coating techniques based on characteristics relevant to interlayer fabrication.

Method	Feedstock	Vacuum	Area scalability	Thickness control	Conformality	Materials flexibility
Pulsed laser deposition	Ceramic target	Required	Limited to moderate	Excellent	Moderate	Moderate
Sputtering	Ceramic or metal target	Required	Moderate	Excellent	Limited to moderate	Moderate
Atomic layer deposition	Volatile chemical precursor	Required or controlled reactor	Moderate to high	Excellent	Excellent	Limited
Screen printing	Powder ink or paste	Not required	High	Limited below µm scale	Limited	High
Powder-based ESD	Powder suspension	Not required	High	Moderate	Moderate	High
Precursor-based ESD (This work)	Molecular precursor solution	Not required	High	High	High	High

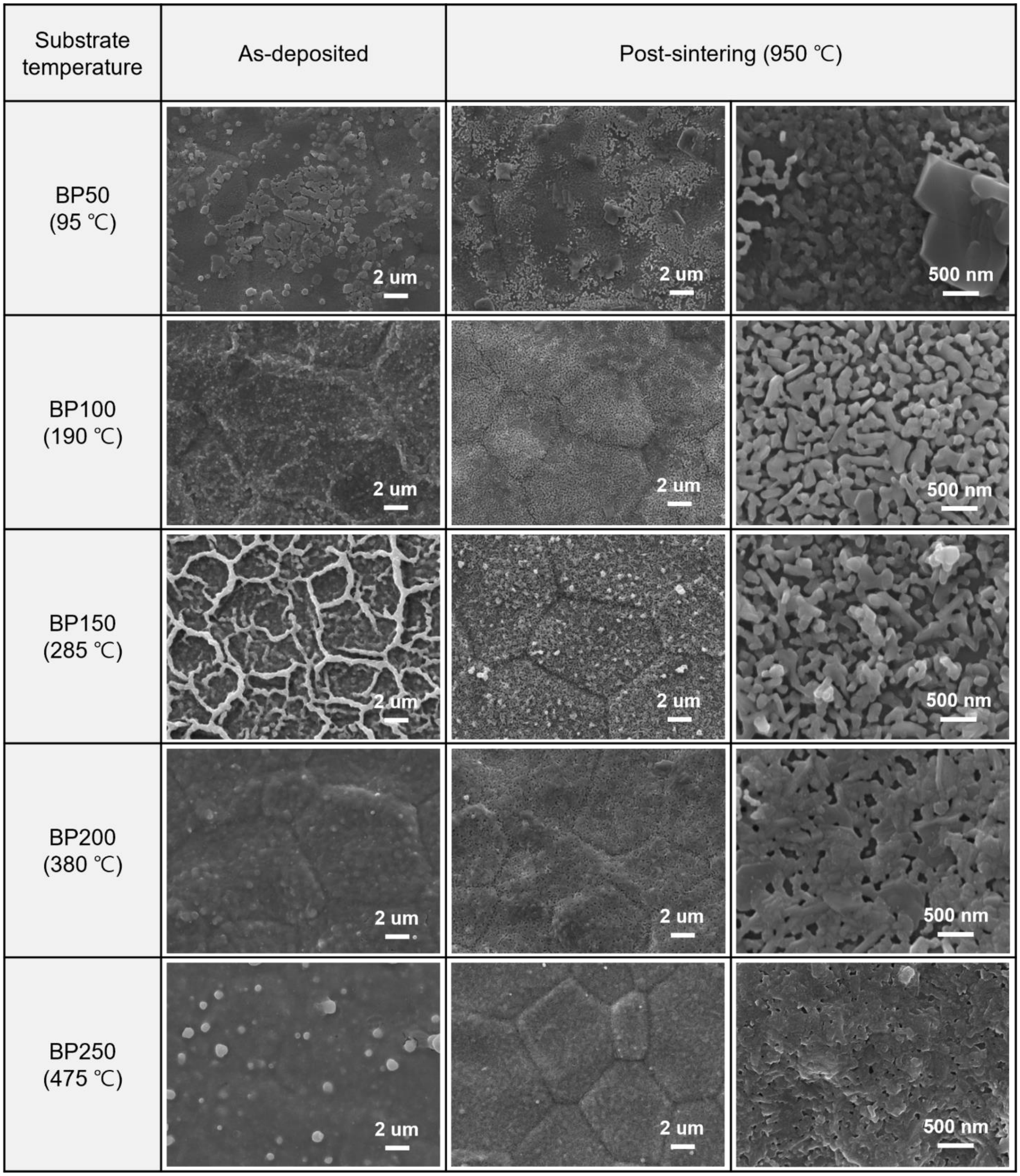

Supplementary Fig. 5. Structural characterization of the precursor-based interlayer was conducted at five substrate temperatures: BP50, BP100, BP150, BP200, and BP250, corresponding to 50%, 100%, 150%, 200%, and 250% of the solvent boiling point (190 ℃), respectively. Surface

SEM images were analyzed to evaluate the interlayer morphology in both the as-deposited state and after sintering at 950 ℃. In droplet-based deposition processes, substrate temperature strongly affects solvent evaporation behavior and droplet size, thereby regulating capillary flow and the resulting film morphology. Five substrate temperatures (BP50, BP100, BP150, BP200, and BP250), corresponding to 50%, 100%, 150%, 200%, and 250% of the solvent boiling point (190 ℃), were investigated. At a substrate temperature of BP50 (95 ℃), insufficient solvent evaporation resulted in partially agglomerated structures. Increasing the substrate temperature to BP100 (190 ℃) and BP150 (285 ℃) intensified capillary flow, driving solutes toward droplet edges and forming non-uniform ring-like structures. At the substrate temperatures of BP200 (380 ℃) and BP250 (475 ℃), the droplet size decreased below the critical threshold for capillary flow generation, resulting in a highly concentrated and relatively uniform deposition.

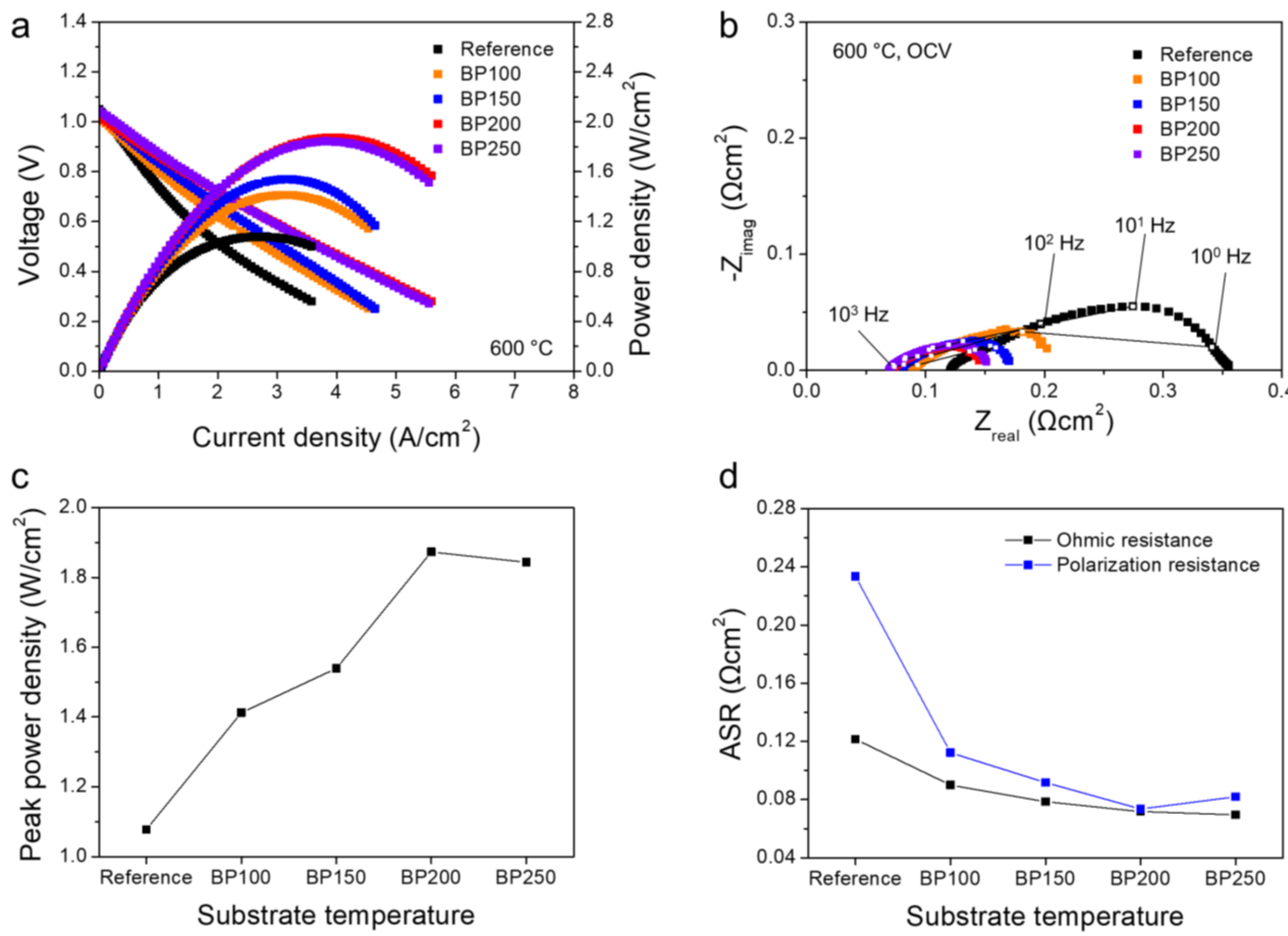

Supplementary Fig. 6. (a) I–V–P curves and (b) Nyquist plots of the reference and interlayer-deposited cells at four substrate temperatures. (c) Peak power density and (d) ohmic and polarization resistances of the corresponding cells measured at 600 °C. The peak power density reached its maximum, while both ohmic and polarization resistances were minimized, as the substrate temperature increase up to BP200 (380 °C), and then became saturated at higher temperature. Source data for Supplementary Fig. 6 are provided as a Source Data file.

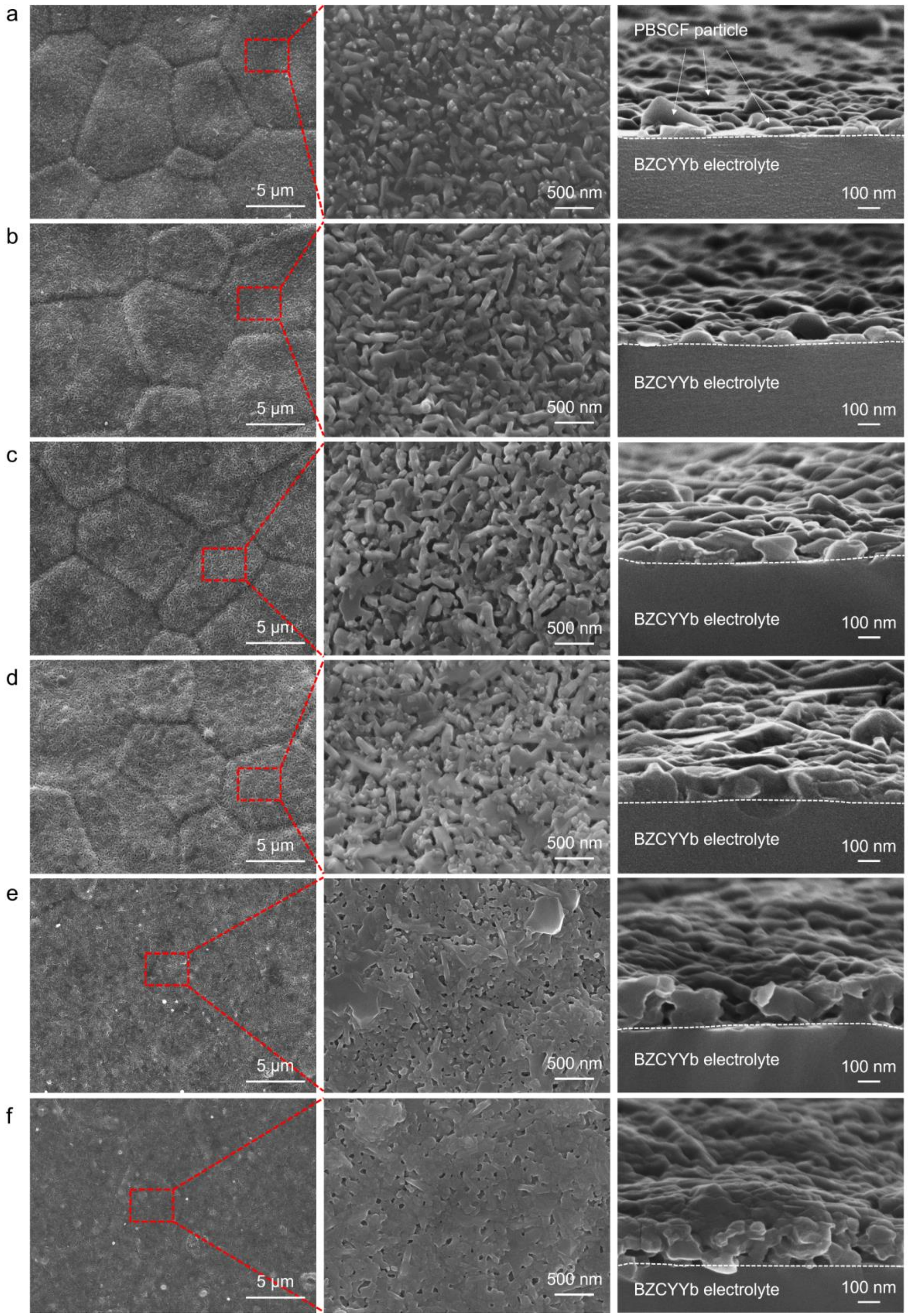

a
5 µm
500 nm
PBSCF particle
BZCYYb electrolyte
100 nm
b
5 µm
500 nm
BZCYYb electrolyte
100 nm
c
5 µm
500 nm
BZCYYb electrolyte
100 nm
d
5 µm
500 nm
BZCYYb electrolyte
100 nm
e
5 µm
500 nm
BZCYYb electrolyte
100 nm
f
5 µm
500 nm
BZCYYb electrolyte
100 nm

Supplementary Fig. 7. Structural characterization of the precursor-based interlayer deposited with varying spray durations. Surface and tilted SEM images for spray durations of (a) 4 h, (b) 6 h, (c) 8 h, (d) 10 h, (e) 16 h, and (f) 24 h. The coverage on the BZCYYb electrolyte surface increased with longer spray duration from 4 to 8 h and became saturated with longer spray duration, while the interlayer thickness continuously increased up to 24 h. (~150 nm for 8 h and ~400 nm for 24 h).

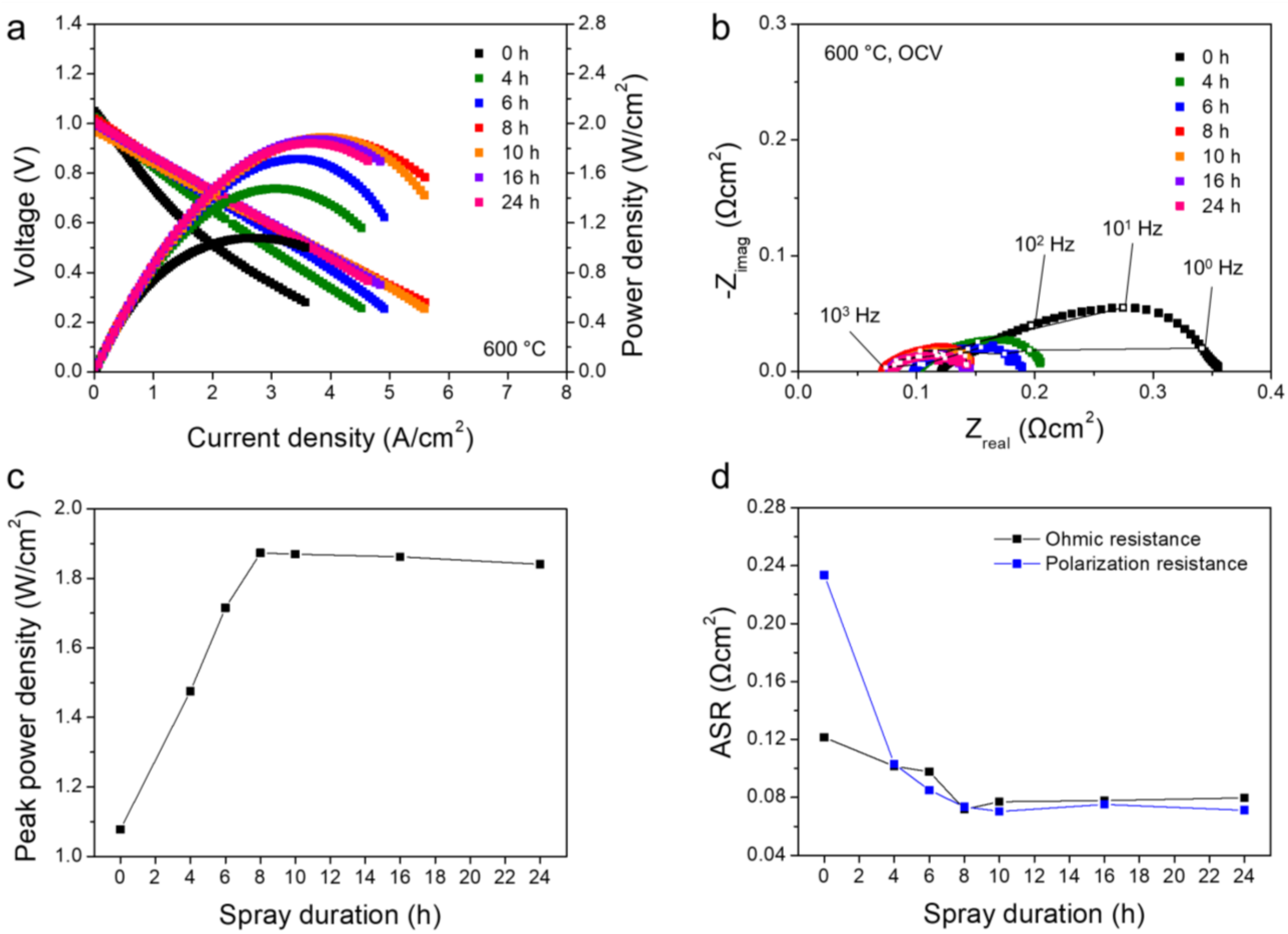

Supplementary Fig. 8. (a) I–V–P curves and (b) Nyquist plots of the reference and interlayer-deposited cells at different spray durations. (c) Peak power density and (d) ohmic and polarization resistances of the corresponding cells measured at 600 °C. Electrochemical analyses showed a concurrent increase in peak power density and a decrease in both ohmic and polarization resistances with increasing spray duration up to 8 h. Beyond 8 h, further thickening of the interlayer led to a slight increase in the ohmic resistance, which we attribute to an increased ion diffusion length through a thicker interlayer. Accordingly, 8 h was selected as the optimal spray duration. Source data for Supplementary Fig. 8 are provided as a Source Data file.

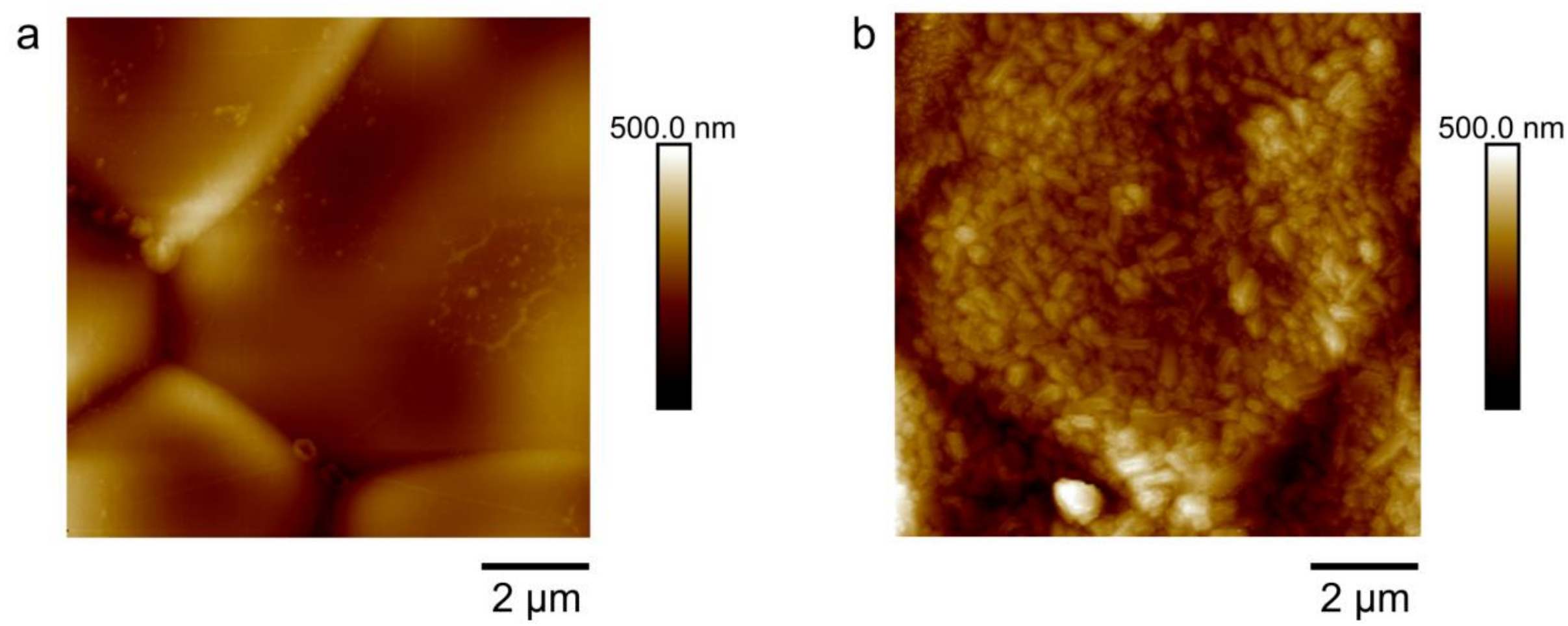

Supplementary Fig. 9. AFM images of (a) the pristine electrolyte surface and (b) the precursor-based interlayer surface. The pristine electrolyte surface exhibited an RMS roughness of 46.7 ± 16.8 nm and a specific surface area of 1.010 ± 0.010 $\mu m^2/\mu m^2$, while the precursor-based interface showed an RMS roughness of 48.6 ± 9.5 nm and a specific surface area of 1.066 ± 0.019 $\mu m^2/\mu m^2$.

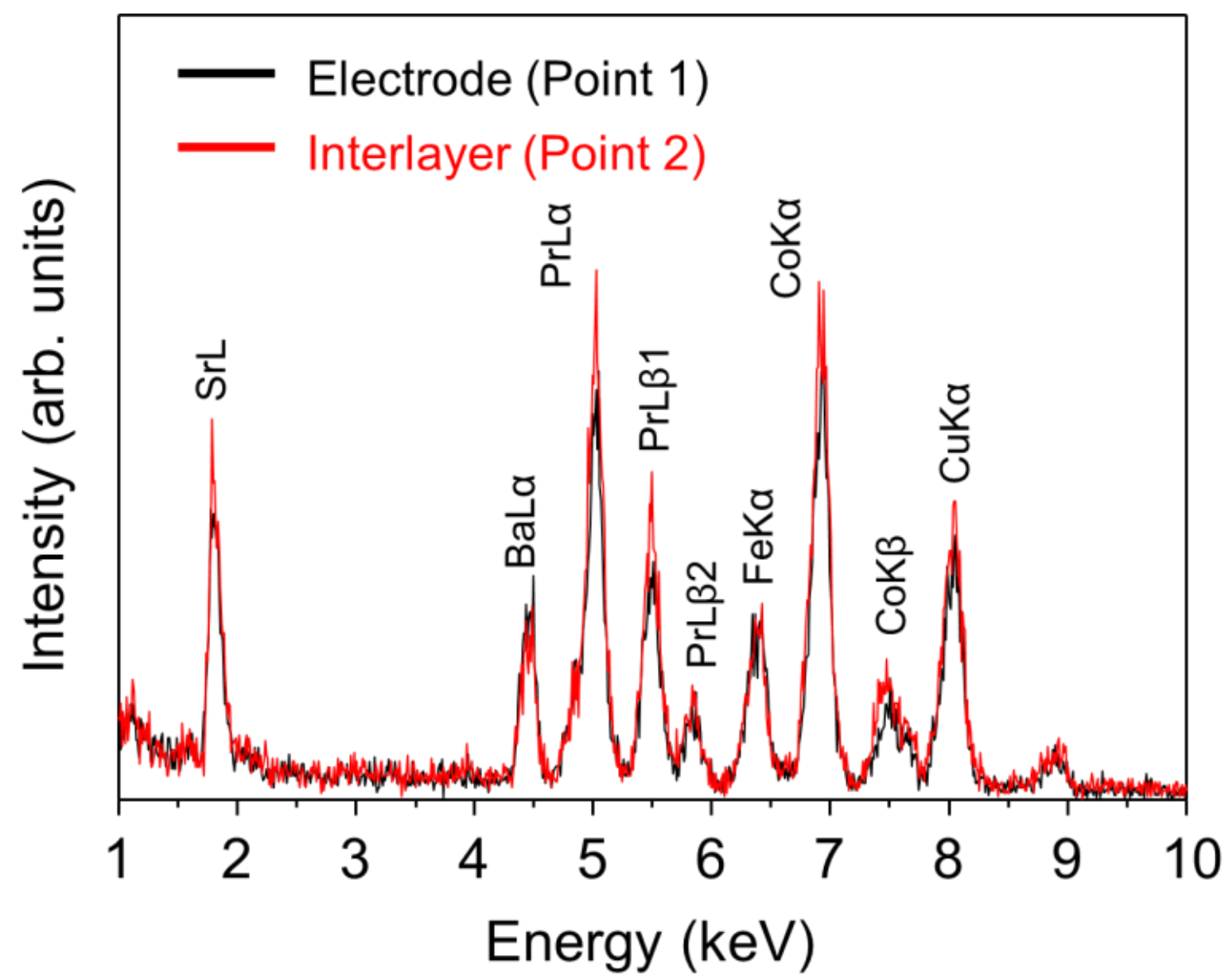

Supplementary Fig. 10. EDS spectra acquired at the powder electrode (Point 1) and the precursor-based interlayer (Point 2). Source data for Supplementary Fig. 10 are provided as a Source Data file.

Supplementary Table 2. Atomic ratios acquired at the powder electrode (Point 1) and the precursor-based interlayer (Point 2) from EDS spectra in Supplementary Fig. 10.

	Atomic ratio over (Pr+Ba+Sr+Co+Fe)				
	Pr	Ba	Sr	Co	Fe
Electrode (Point 1)	0.220	0.082	0.180	0.434	0.083
Interlayer (Point 2)	0.225	0.084	0.171	0.445	0.075

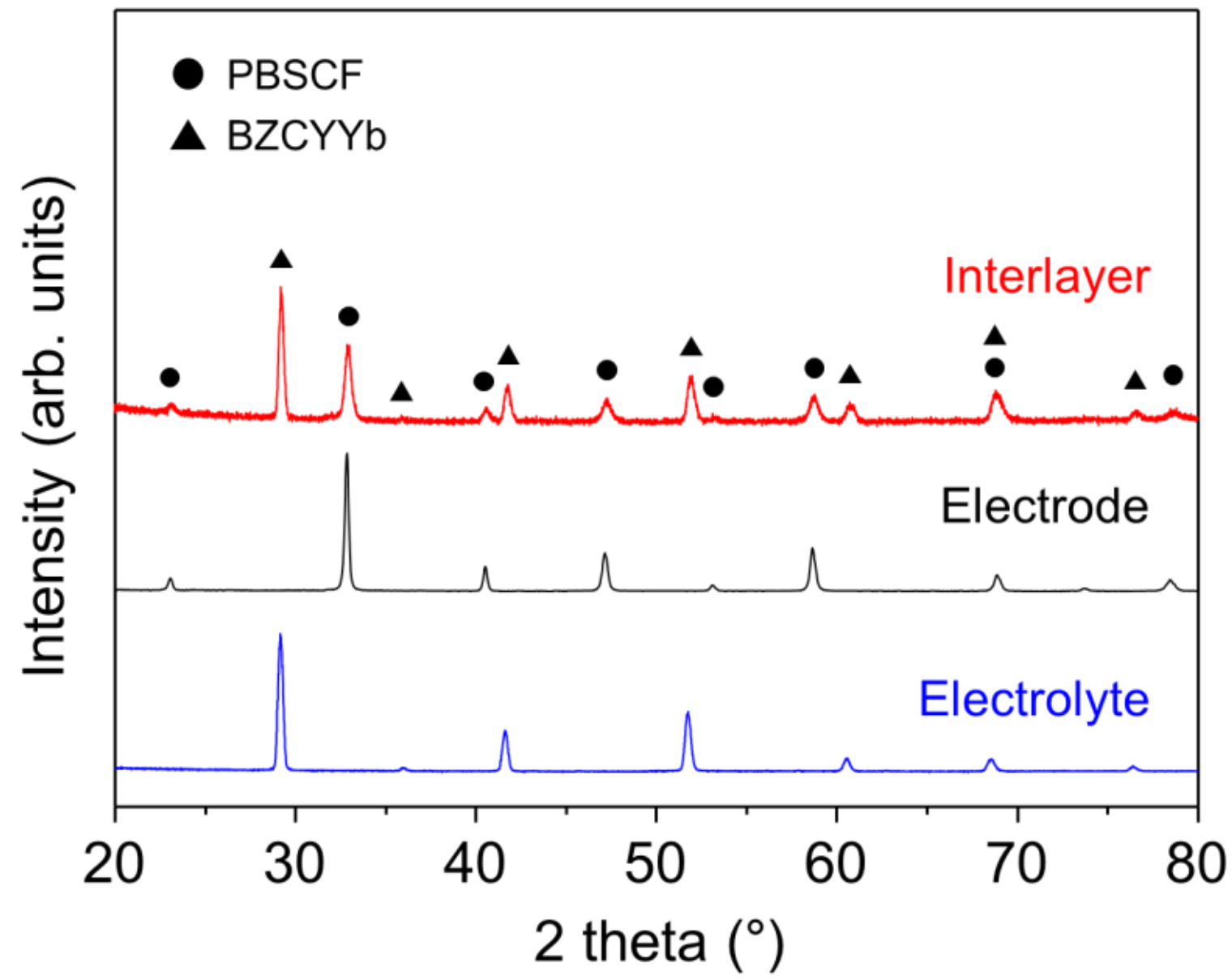

Supplementary Fig. 11. XRD patterns of the precursor-based interlayer, powder electrode, and electrolyte. The crystallite sizes of the electrode and interlayer are 25.1 ± 2.7 nm and 21.1 ± 1.9 nm, respectively. Source data for Supplementary Fig. 11 are provided as a Source Data file.

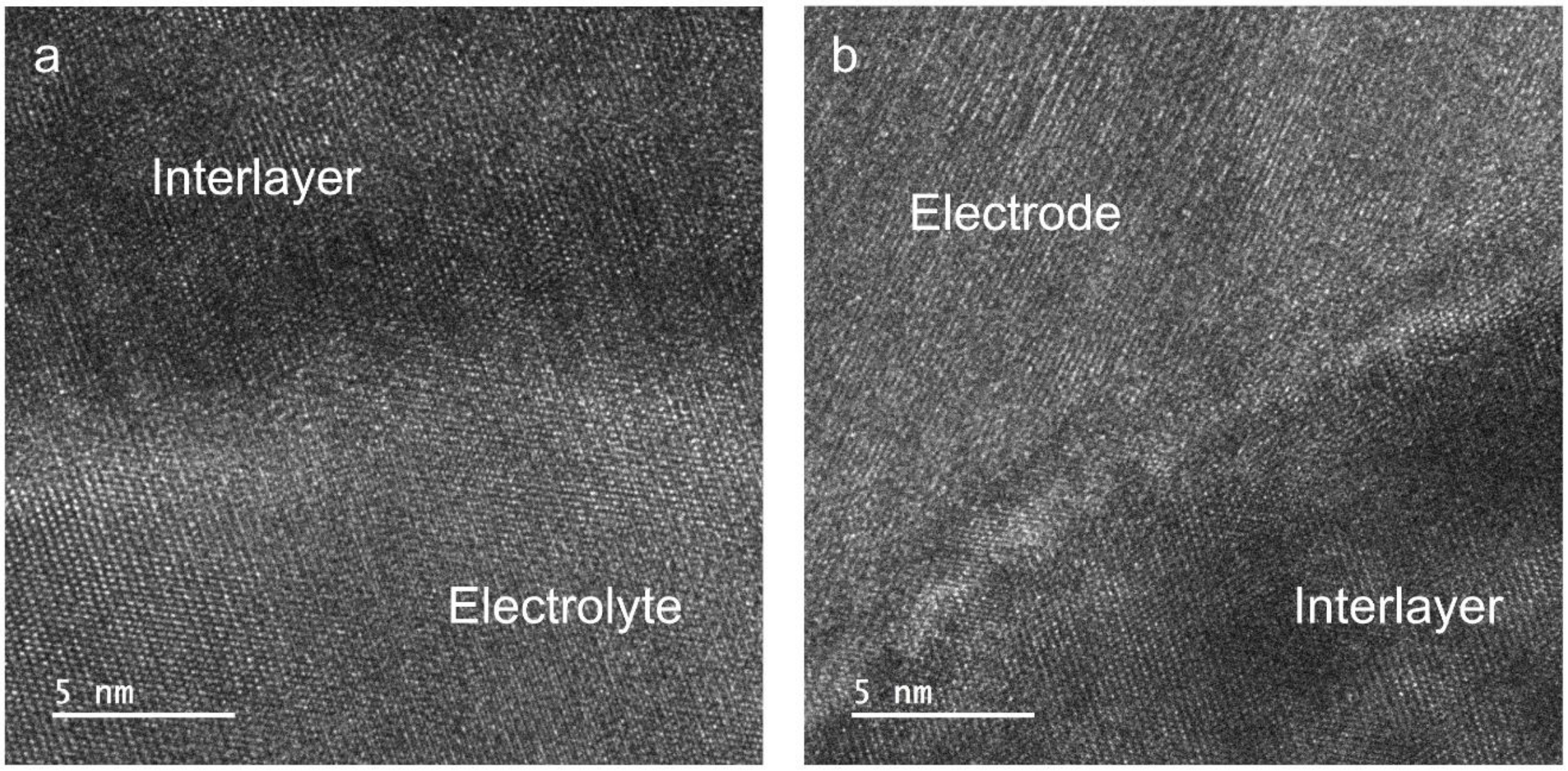

Supplementary Fig. 12. High-resolution cross-sectional TEM images of (a) the interface between the interlayer and the electrolyte and (b) the interface between the electrode and the interlayer.

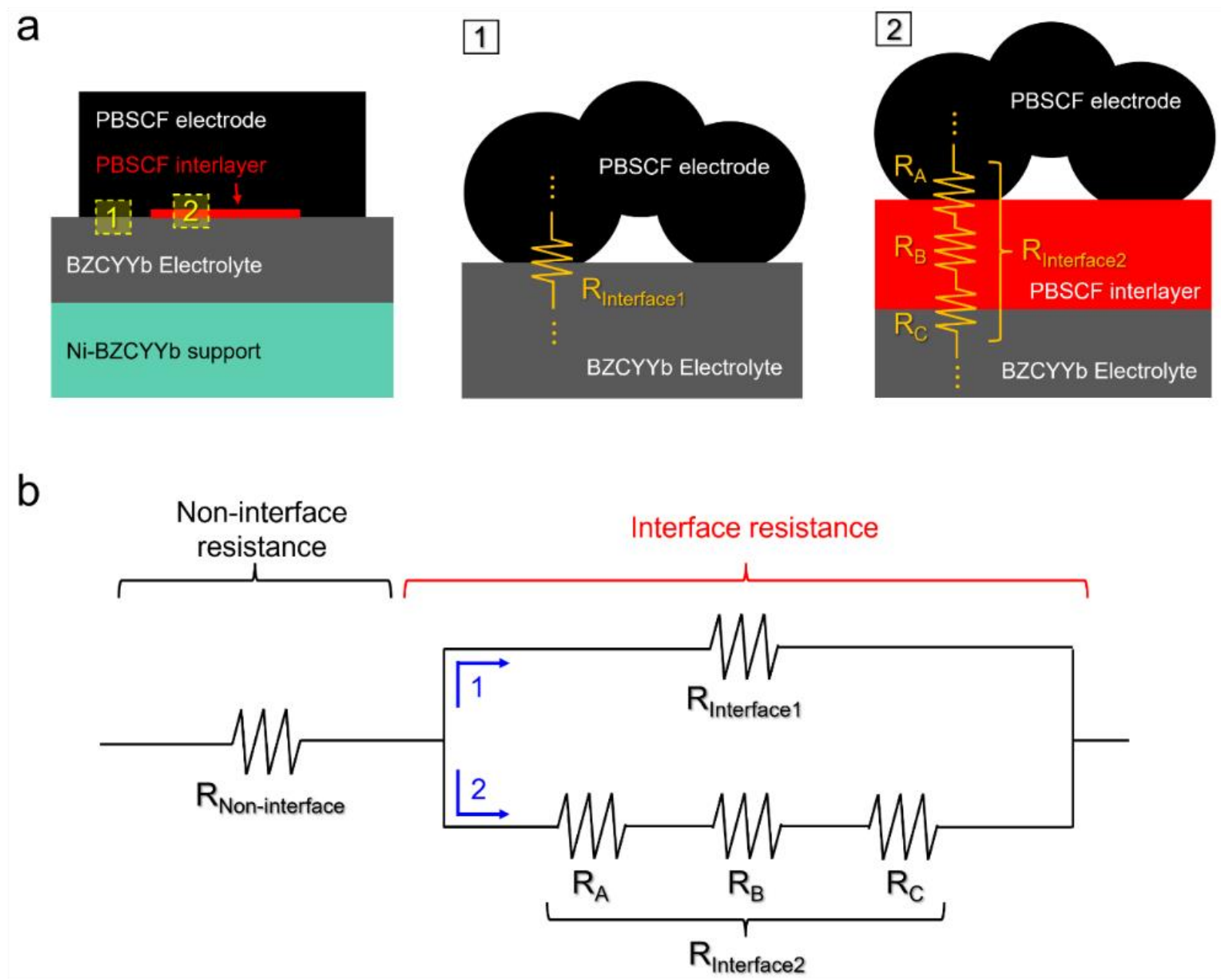

Supplementary Fig. 13. (a) Schematic of the model sample with the corresponding equivalent circuit model for the ohmic resistance at two types of interfaces, with and without the PBSCF interlayer. (b) Equivalent circuit model used to describe the total ohmic resistance.

The resistance terms are defined as follows:

R_{total}: Area-specific total ohmic resistance

$R_{non\text{-}interface}$: Area-specific resistance from all components except the PBSCF electrode/BZCYYb electrolyte interface

$R_{interface}$: Area-specific resistance from the PBSCF electrode/BZCYYb electrolyte interface

$R_{interface1}$: $R_{interface}$ without the PBSCF interlayer

$R_{interface2}$: $R_{interface}$ with the PBSCF interlayer

For the interlayer-containing configuration, $R_{interface2}$ is further decomposed into:

R_A: Area-specific resistance from the PBSCF electrode/PBSCF interlayer interface

R_B: Area-specific resistance from ionic and electronic transport within the PBSCF interlayer

R_C: Area-specific resistance from the PBSCF interlayer/BZCYYb electrolyte interface

The corresponding ohmic resistance model can be expressed as:

$$R_{total} = R_{non-interface} + R_{interface}$$

Considering the PBSCF interlayer fraction, a, defined as the area fraction of the PBSCF interlayer relative to the total electrode area, the interface resistance can be written as:

$$R_{total} = R_{non-interface} + \left(\frac{1-a}{R_{interface1}} + \frac{a}{R_{interface2}}\right)^{-1}$$

Here, $R_{interface1}$ and $R_{interface2}$ correspond to the interface resistances without and with the PBSCF interlayer, respectively. For the interlayer-containing configuration, $R_{interface2}$ is composed of three contributions:

$$R_{interface2} = R_A + R_B + R_C$$

where R_A, R_B, and R_C represent the resistances at the PBSCF electrode/PBSCF interlayer interface, within the PBSCF interlayer, and at the PBSCF interlayer/BZCYYb electrolyte interface, respectively.

However, the ohmic contributions from R_A and R_B are negligible under the present conditions. R_A is expected to be small because the PBSCF electrode and the PBSCF interlayer are co-sintered, promoting strong interfacial integration between identical PBSCF phases. R_B is also minimized by the ultrathin interlayer thickness (~150 nm) and the substantially higher conductivity of PBSCF (~700 S/cm at 500 °C)[17] compared to BZCYYb (~0.018 S/cm at 500 °C)[4]. Accordingly, $R_{interface2}$ can be approximated as:

$$R_{interface2} \approx R_C$$

The interface resistance is then expressed in terms of two key parameters: contact resistance and contact coverage, defined as follows:

$R_{contact1}$: Area-specific contact resistance at the PBSCF electrode/BZCYYb electrolyte interface

$R_{contact2}$: Area-specific contact resistance at the PBSCF interlayer/BZCYYb electrolyte interface

θ_1: Contact coverage at the PBSCF electrode/BZCYYb electrolyte interface

θ_2: Contact coverage at the PBSCF interlayer/BZCYYb electrolyte interface

Based on these definitions, the total resistance can be written as:

$$R_{total} = R_{non-interface} + \left(\frac{1-a}{R_{contact1} \cdot \frac{1}{\theta_1}} + \frac{a}{R_{contact2} \cdot \frac{1}{\theta_2}} \right)^{-1}$$

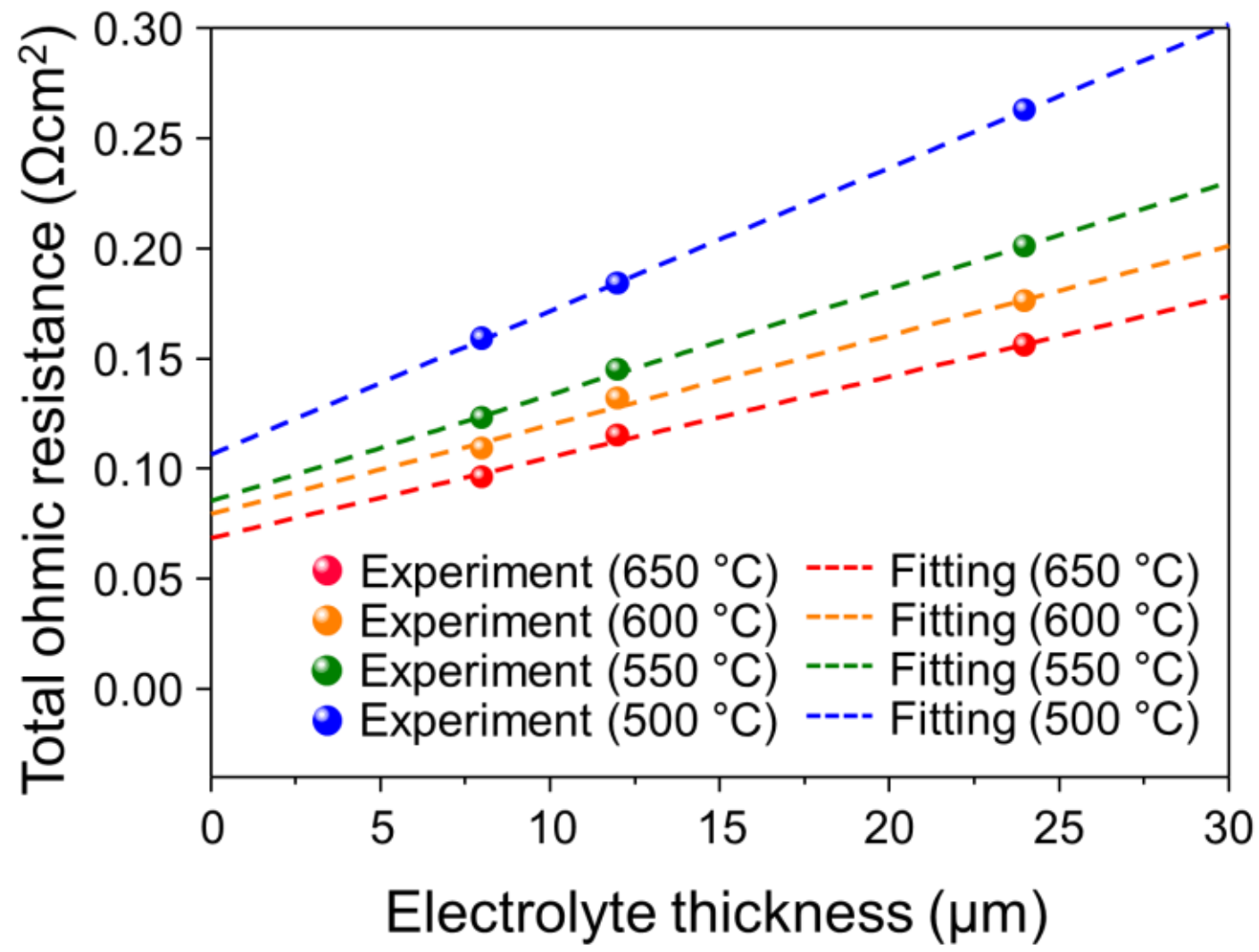

Supplementary Fig. 14. Total ohmic resistance as a function of electrolyte thickness. The electrolyte thickness was controlled by varying the number of spin-coating processes. Source data for Supplementary Fig. 14 are provided as a Source Data file.

Supplementary Table 3. Comparison of the electrolyte conductivity and corresponding activation energy with reported values.

	Conductivity (S/cm)				Activation energy (eV)
	650 °C	600 °C	550 °C	500 °C	
This work (Electrolyte-thickness dependent experiment)	0.027	0.025	0.021	0.015	0.24
[4]	0.033	0.027	0.024	0.018	0.23
[18]	0.018	0.014	0.011	0.008	0.33
[19]	0.014	0.012	0.010	0.007	0.27

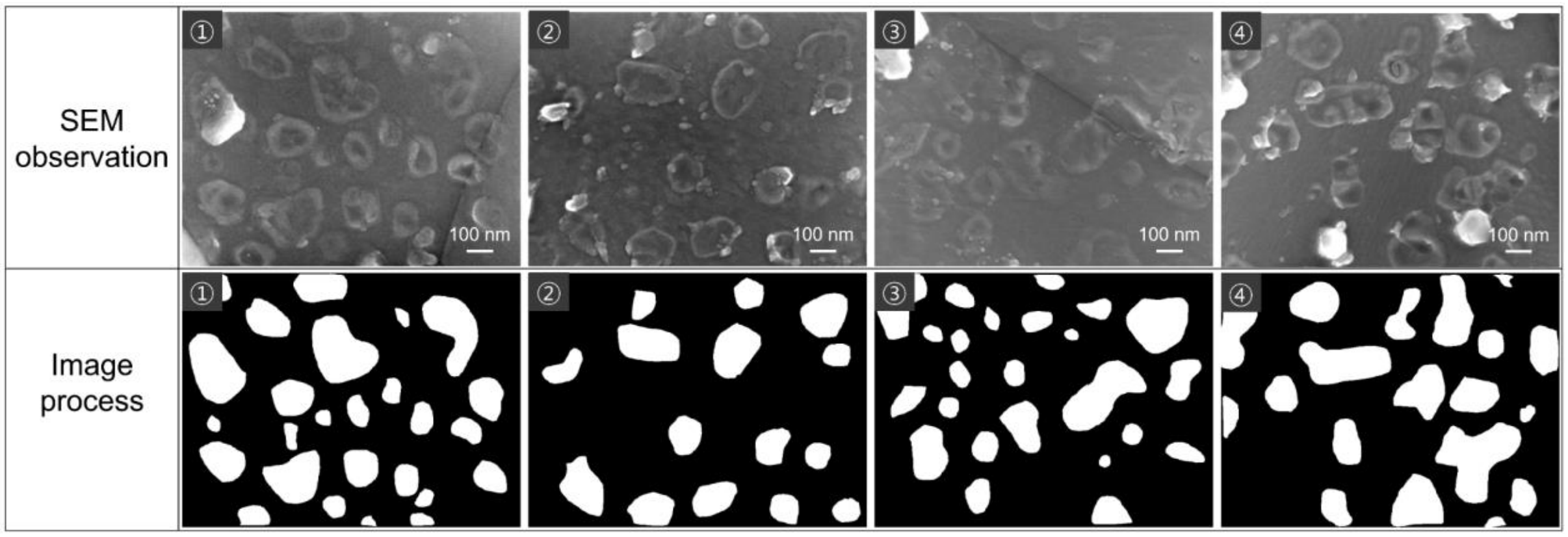

Supplementary Fig. 15. Image processing of the BZCYYb electrolyte surface after detaching the PBSCF electrode particles of IL-0%. Using the image processing software (Image J), the contact areas (crater-like traces) were identified as white regions. Then, the contact coverage was calculated using the equation shown below.

$$Contact\ coverage\ (\%) = \frac{number\ of\ pixels\ for\ contact\ area}{number\ of\ pixels\ for\ electrolyte\ surface} \times 100$$

Supplementary Table. 4. Measured contact coverage of IL-0% derived from the analysis in Supplementary Fig. 15.

Location	1	2	3	4
Number of pixels for electrolyte surface	428652	428652	428652	428652
Number of pixels for contact area	120691	78773	98440	125119
Contact coverage (%)	28.156	18.377	22.965	29.189

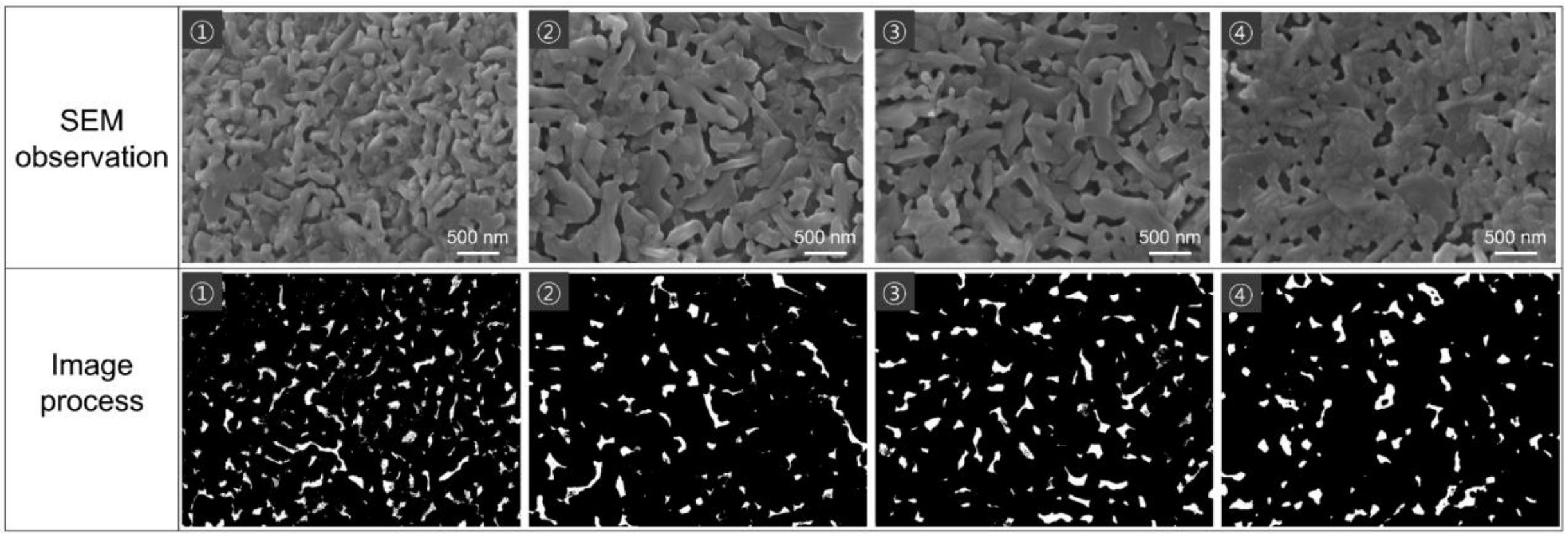

Supplementary Fig. 16. Image processing of the PBSCF interlayer surface. Using the image processing software (Image J), the contact coverage was calculated using the equation shown below.

$$Contact\ coverage\ (\%) = \frac{number\ of\ pixels\ for\ interlayer\ surface}{number\ of\ pixels\ for\ entire\ image} \times 100$$

Supplementary Table. 5. Measured contact coverage of IL-100% derived from the analysis in Supplementary Fig. 16.

Location	1	2	3	4
Number of pixels for entire image	418691	418691	418691	418691
Number of pixels for interlayer surface	384928	391614	386021	388160
Contact coverage (%)	91.936	93.533	92.197	92.708

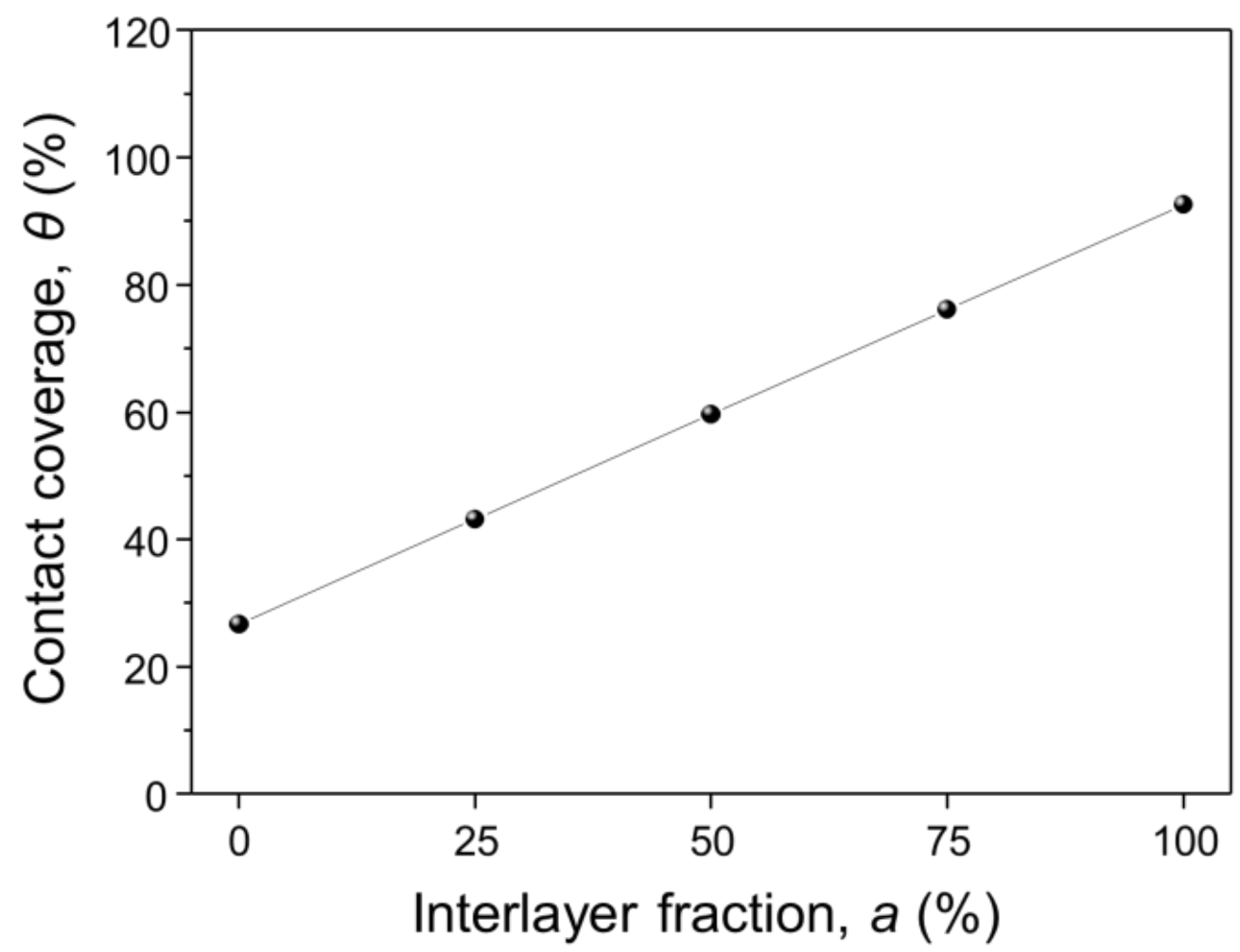

Supplementary Fig. 17. The linear correlation between the interlayer fraction and the contact coverage, determined from the area-weighted combination of the experimentally measured contact coverages of IL-0% and IL-100%. Source data for Supplementary Fig. 17 are provided as a Source Data file.

Supplementary Table 6. The model fitting results at 500–650 °C.

	Temperature (°C)			
	650	600	550	500
$R_{contact1}$ (Ωcm^2)	0.0178	0.0204	0.0234	0.0290
$R_{contact2}$ (Ωcm^2)	0.0174	0.0207	0.0250	0.0268

Supplementary Table 7. Calculated specific contact resistance.

	Temperature (°C)	IL-0%	IL-100%
Total ohmic resistance (Ωcm^2)	650	0.1030	0.0563
	600	0.1176	0.0626
	550	0.1354	0.0754
	500	0.1739	0.0965
Non-interface resistance (Ωcm^2)	650	0.0367	
	600	0.0407	
	550	0.0483	
	500	0.0652	
Interface resistance (Ωcm^2)	650	0.0663	0.0196
	600	0.0769	0.0219
	550	0.0871	0.0271
	500	0.1087	0.0313
Contact coverage (%)	650	26.7 ± 4.1	92.6 ± 0.6
	600		
	550		
	500		
Specific contact resistance (Ωcm^2)	650	0.0177 ± 0.0027	0.0181 ± 0.0001
	600	0.0205 ± 0.0032	0.0203 ± 0.0001
	550	0.0233 ± 0.0036	0.0251 ± 0.0002
	500	0.0290 ± 0.0045	0.0290 ± 0.0002

[Interface resistance] = [Total ohmic resistance] - [Non-interface resistance]

[Specific contact resistance] = [Interface resistance] × [Contact coverage/100]

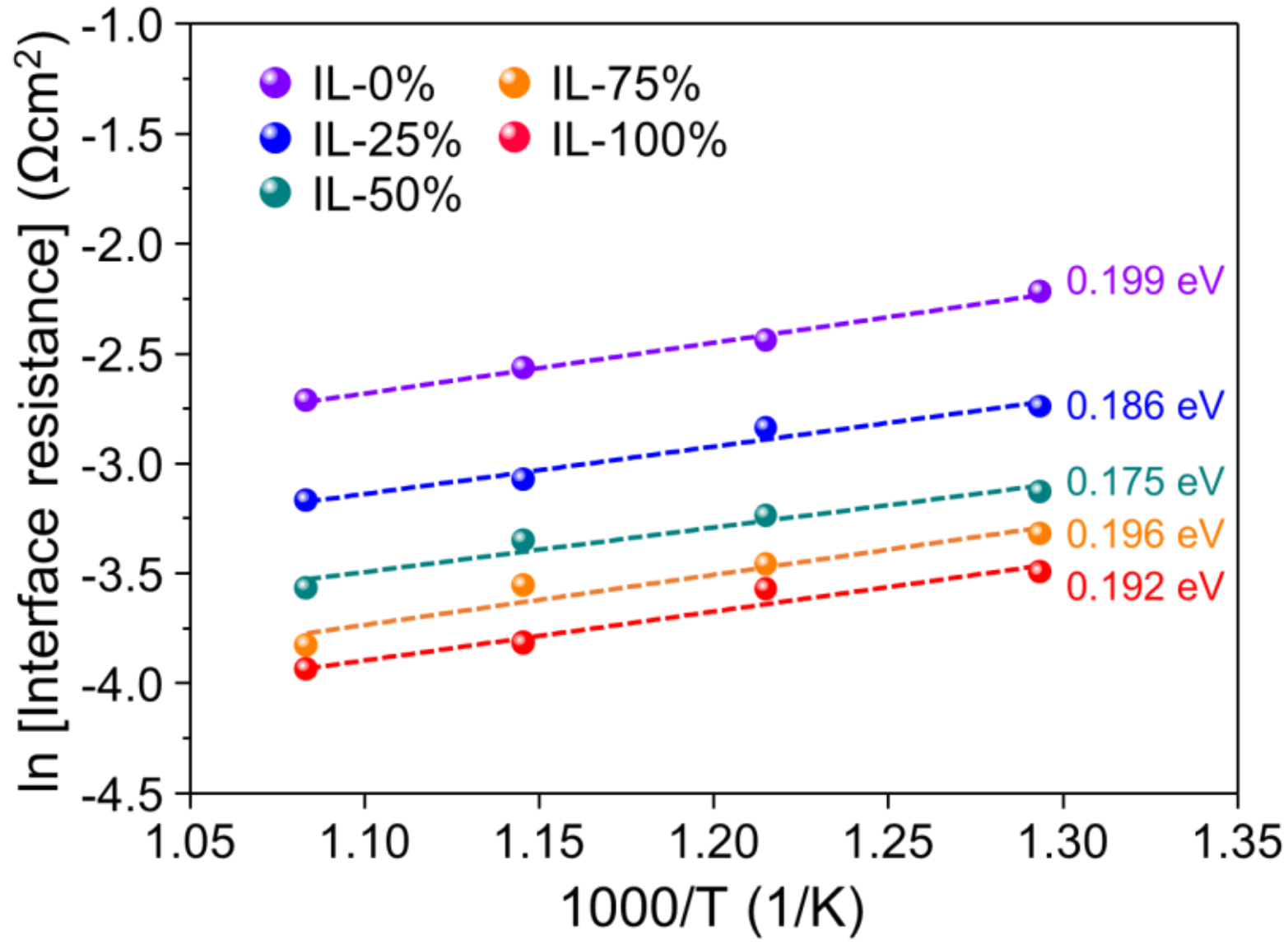

Supplementary Fig. 18. Arrhenius plots of the interfacial ohmic resistances for IL-0%, IL-25%, IL-50%, IL-75%, and IL-100% in the temperature range of 650–500 ℃. Source data for Supplementary Fig. 18 are provided as a Source Data file.

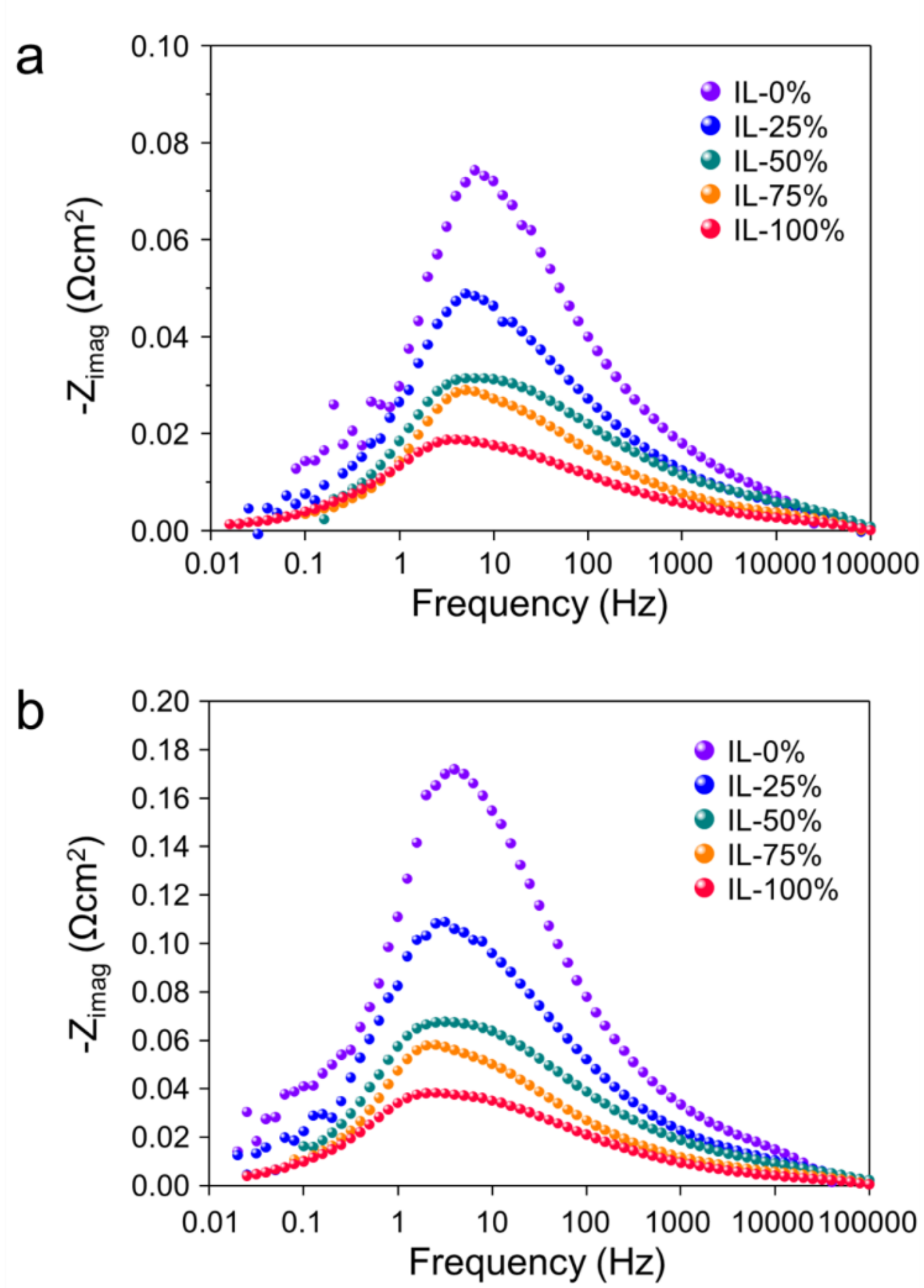

Supplementary Fig. 19. Bode plots of the model samples under OCV conditions at (a) 600 °C and (b) 550 °C. Source data for Supplementary Fig. 19 are provided as a Source Data file.

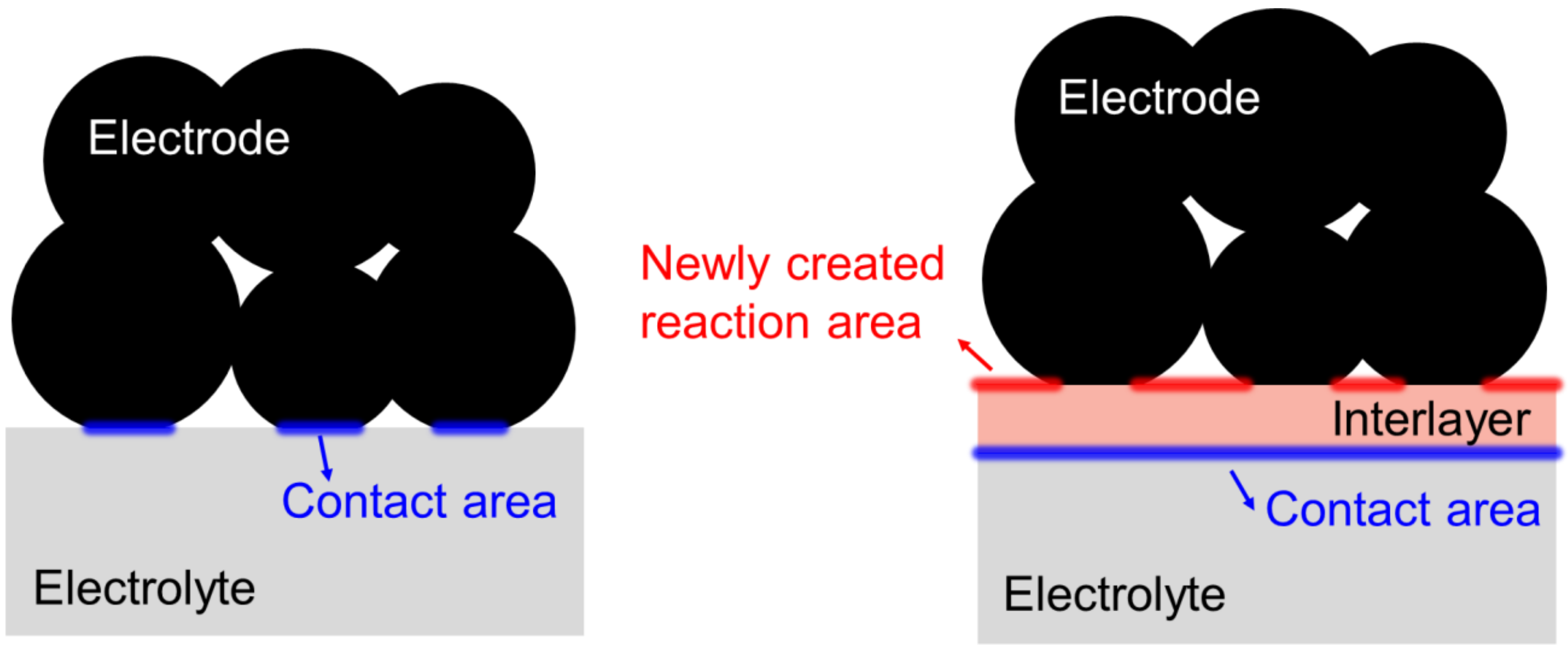

Supplementary Fig. 20. Schematic of the electrode/electrolyte interface with/without the interlayer. The interlayer not only increased the contact coverage with the underlying electrolyte, but also created additional reaction area above the interlayer.

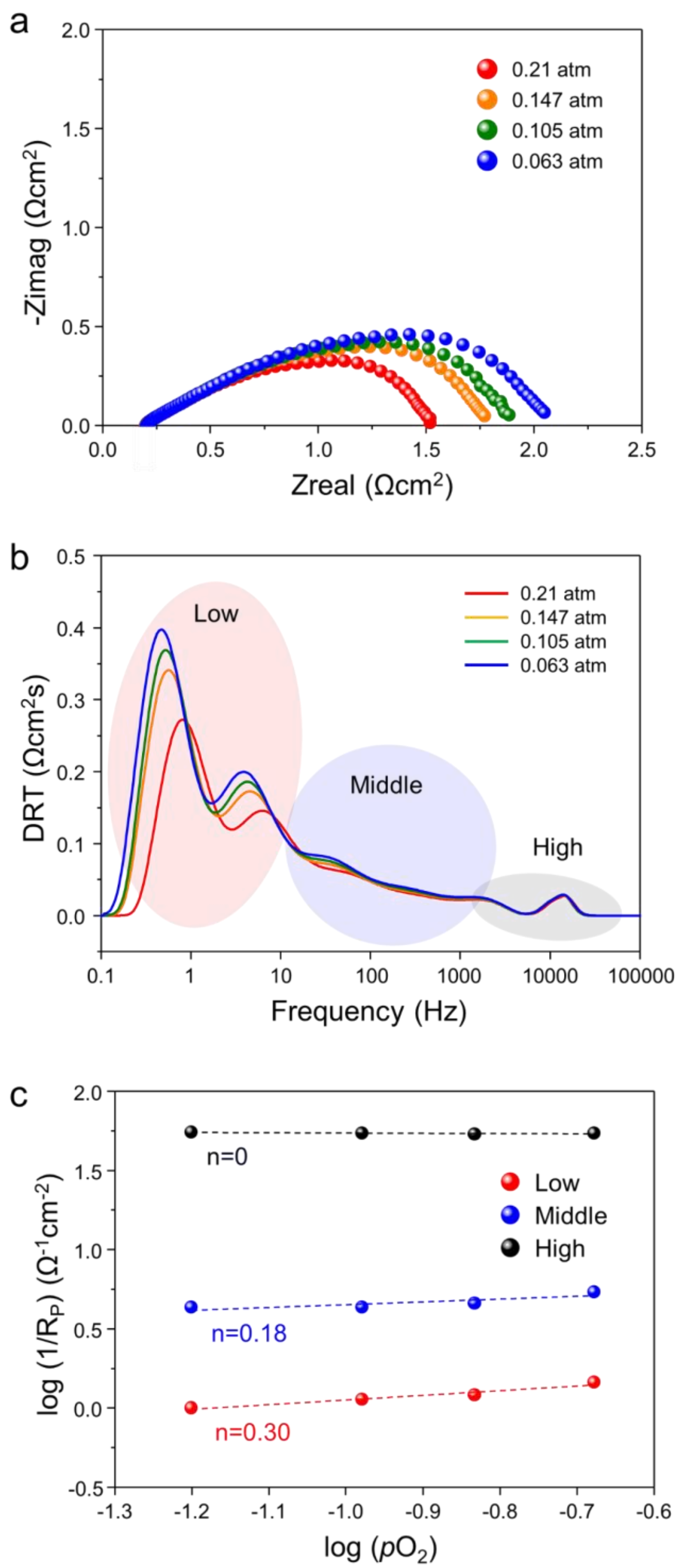

Supplementary Fig. 21. The O_2 partial pressure test at 500 °C. (a) Nyquist plots and (b) DRT analysis under varying O_2 partial pressures at the air electrode under OCV conditions. (c)

Polarization resistances of low-, middle-, and high-frequency regions as a function of the O_2 partial pressure.

Distribution of Relaxation Times (DRT) analysis was employed to deconvolute the polarization resistance with respect to oxygen partial pressure (pO_2) at the air electrode, separating contributions from three distinct frequency ranges. As shown in Supplementary Fig. 18, the polarization resistance exhibited a clear dependence on pO_2, with the low-frequency resistance (R_{low}) and mid-frequency resistance (R_{middle}) components following a power-law relationship with pO_2^{-n}, where n_{low}=0.30 and n_{middle}=0.18, respectively. The reaction order (n) serves as a characteristic indicator of the specific electrochemical reactions involving oxygen ion species. For example, charge transfer, ion diffusion, and gas diffusion exhibit reaction orders of 0.375, 0.25, and 1, respectively, in pO_2-dependent impedance analysis[20,21]. The measured values for the low- (n_{low}=0.30) and middle-frequency (n_{middle}=0.18) ranges are in close agreement with those of charge transfer (n=0.375) and ion diffusion (n=0.25), respectively. Therefore, the dominant reactions for the low- and middle-frequency ranges can be assigned to the charge transfer and ion diffusion reactions, respectively. Source data for Supplementary Fig. 21 are provided as a Source Data file.

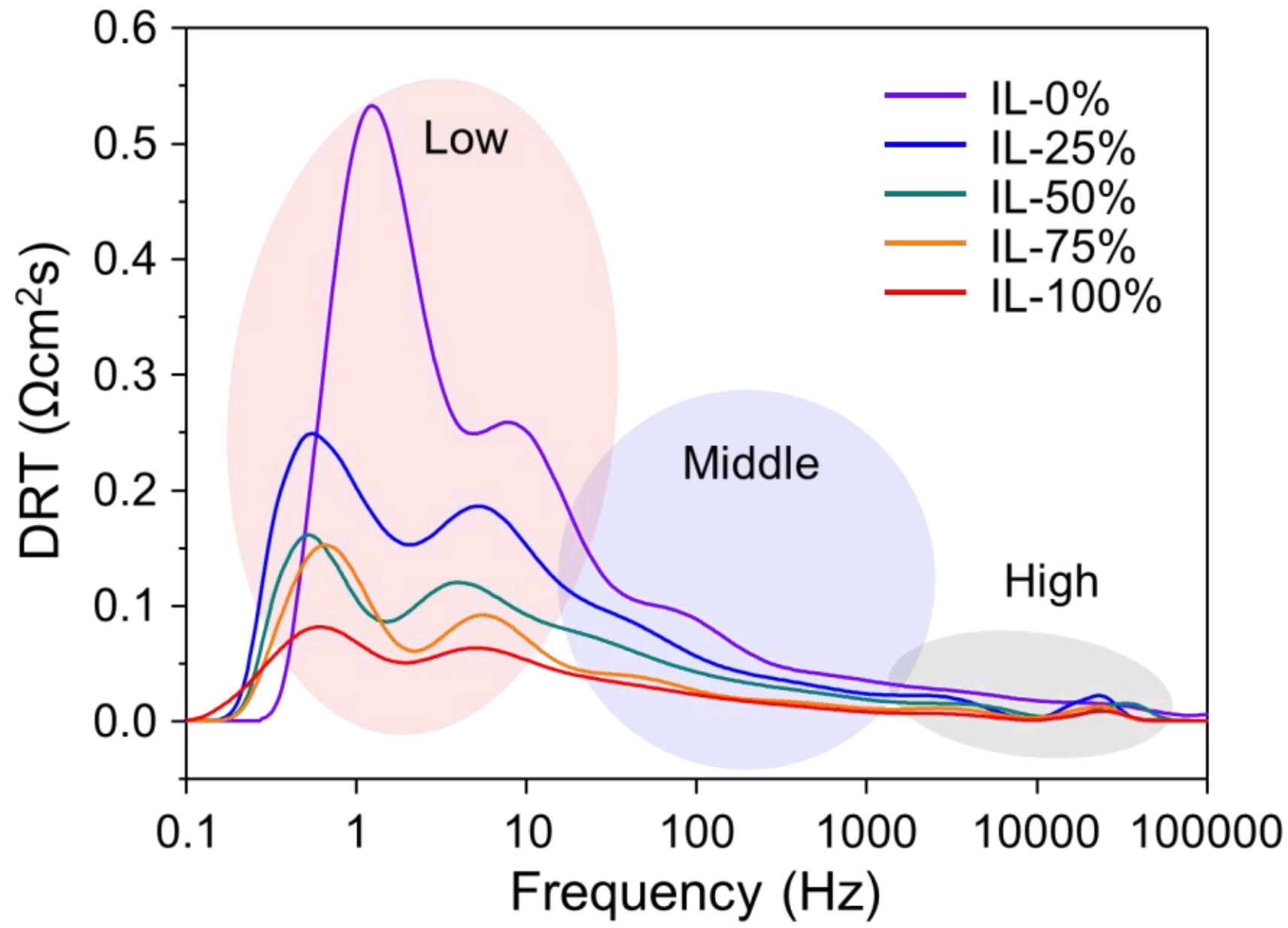

Supplementary Fig. 22. Distributed relaxation time (DRT) analysis of the model samples at 500 °C under OCV conditions. Source data for Supplementary Fig. 22 are provided as a Source Data file.

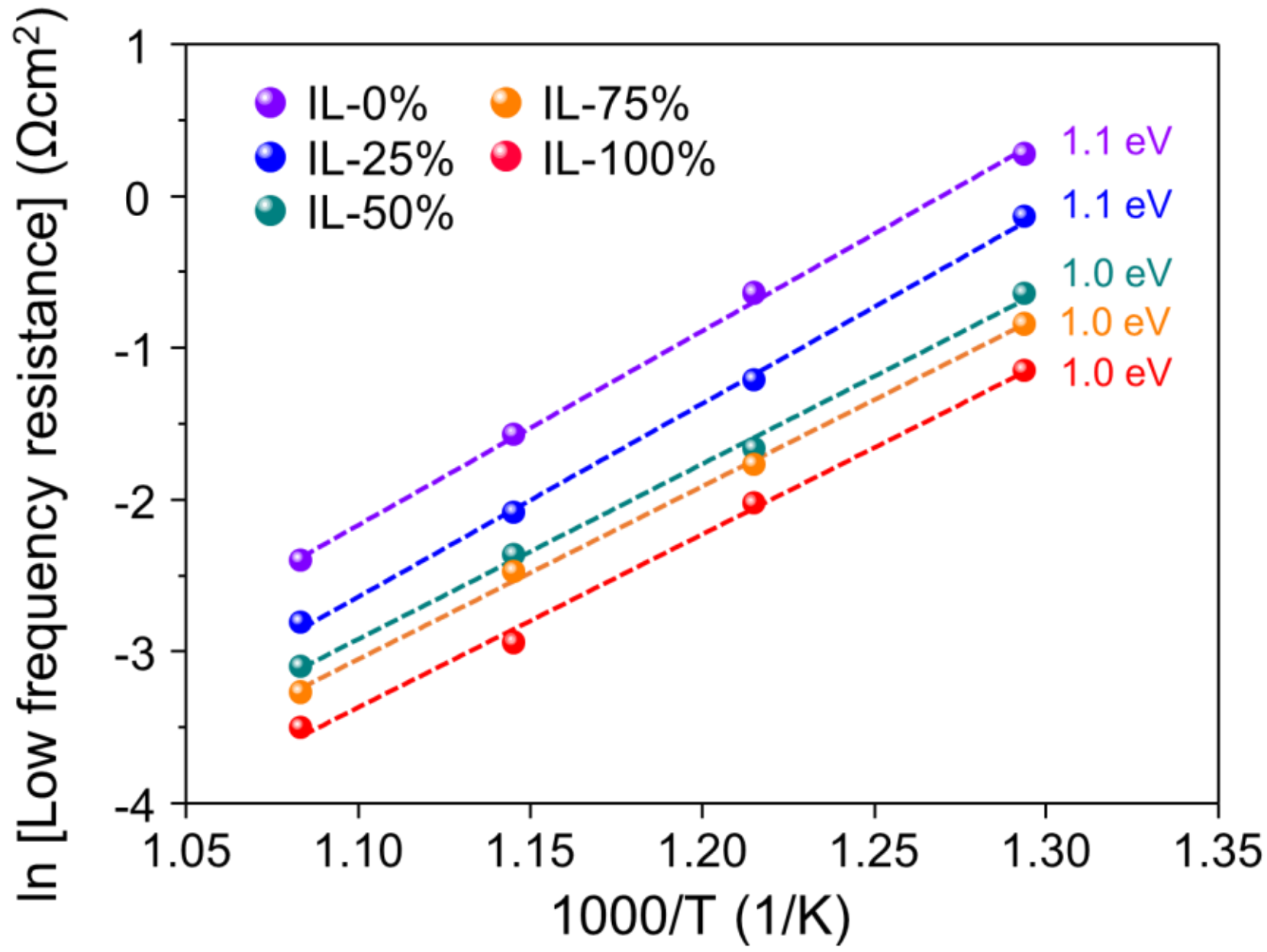

Supplementary Fig. 23. Arrhenius plots of the low-frequency resistances for IL-0%, IL-25%, IL-50%, IL-75%, and IL-100% in the temperature range of 650–500 ℃. Source data for Supplementary Fig. 23 are provided as a Source Data file.

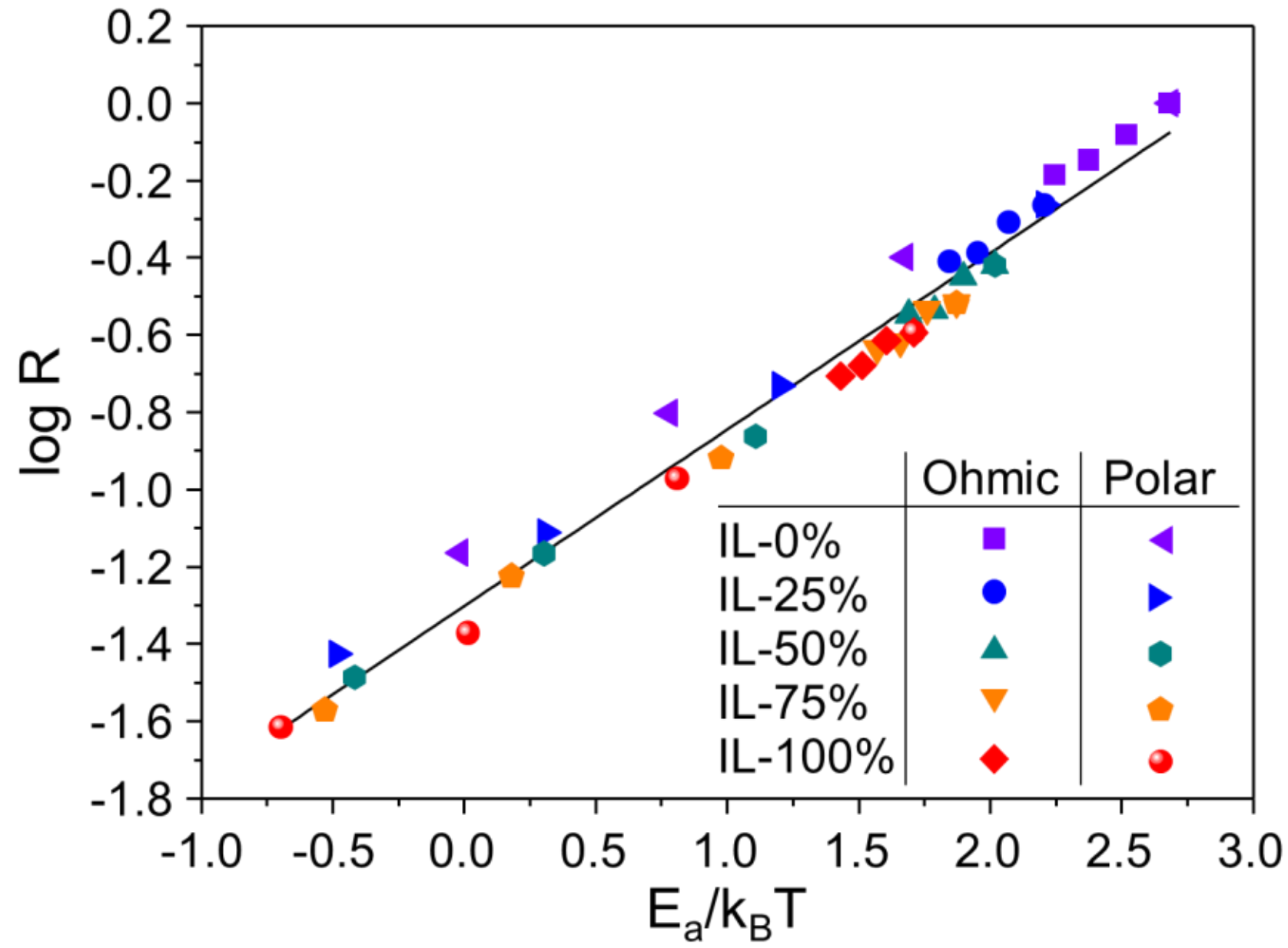

Supplementary Fig. 24. Arrhenius plot of the relative 'interface resistance' R against the inverse of reduced temperature k_BT/E_a[8]. Source data for Supplementary Fig. 24 are provided as a Source Data file.

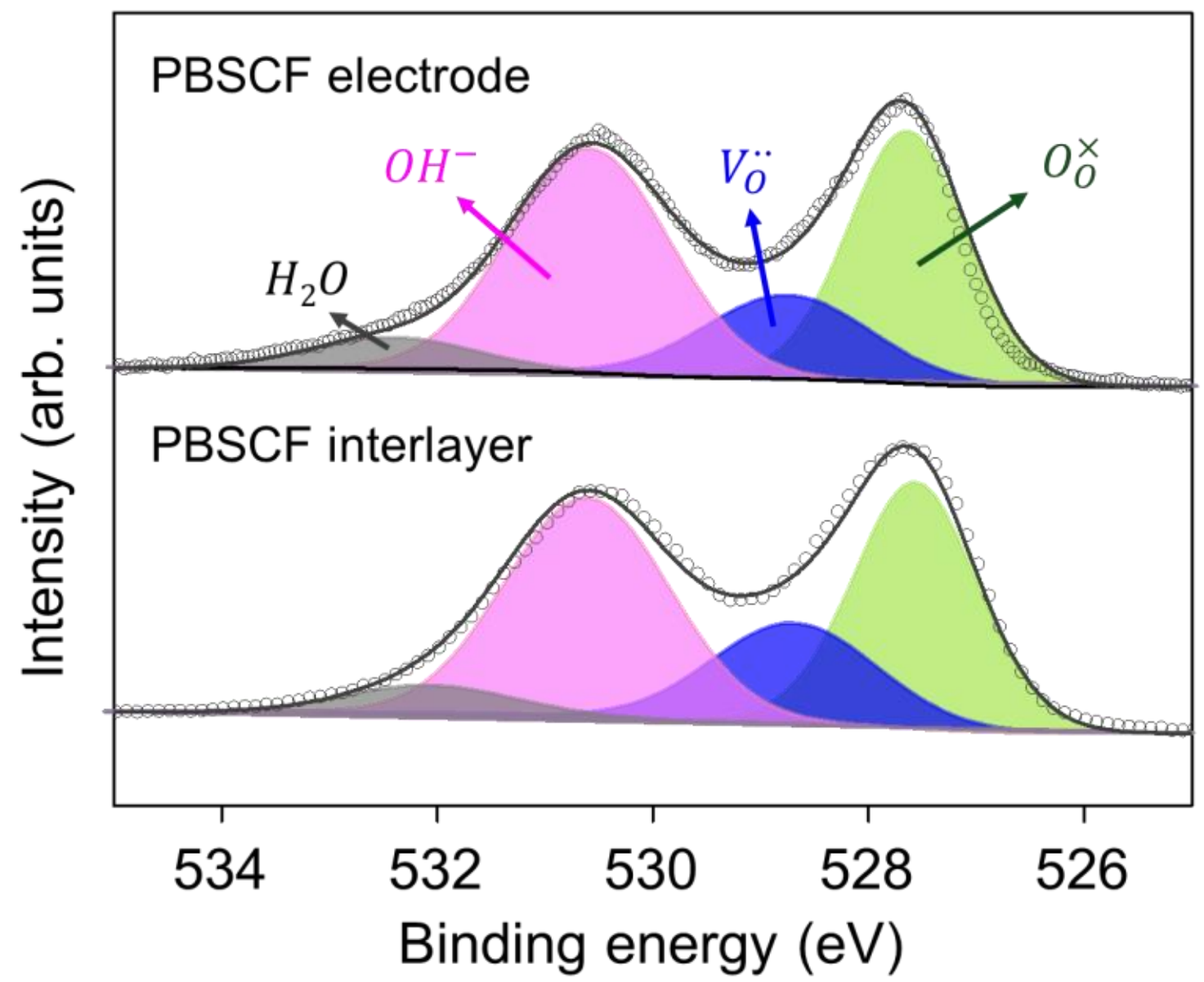

Supplementary Fig. 25. XPS O 1*s* spectra of PBSCF electrode and PBSCF interlayer. Source data for Supplementary Fig. 25 are provided as a Source Data file.

Supplementary Table 8. Relative peak areas of each component in XPS O 1*s* spectra of PBSCF electrode and PBSCF interlayer.

	$O_O^\times$	$V_O^{\cdot\cdot}$	OH^-	H_2O
PBSCF electrode	0.36	0.16	0.42	0.06
PBSCF interlayer	0.35	0.18	0.41	0.06

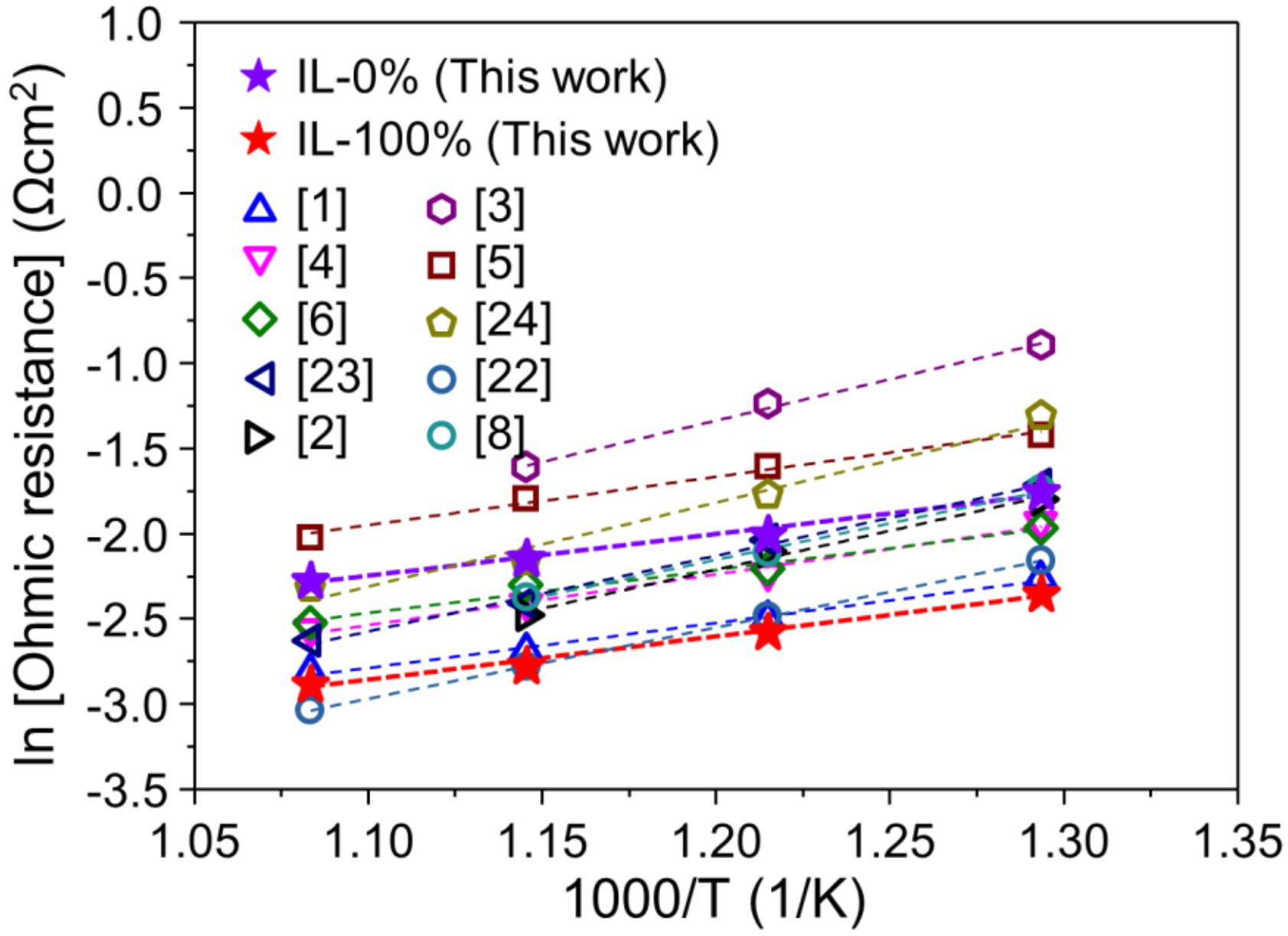

Supplementary Fig. 26. Arrhenius plots of the ohmic resistances for IL-0% and IL-100% with previously reported PCECs in the temperature range of 650−500 °C[1-6,8,22-24].

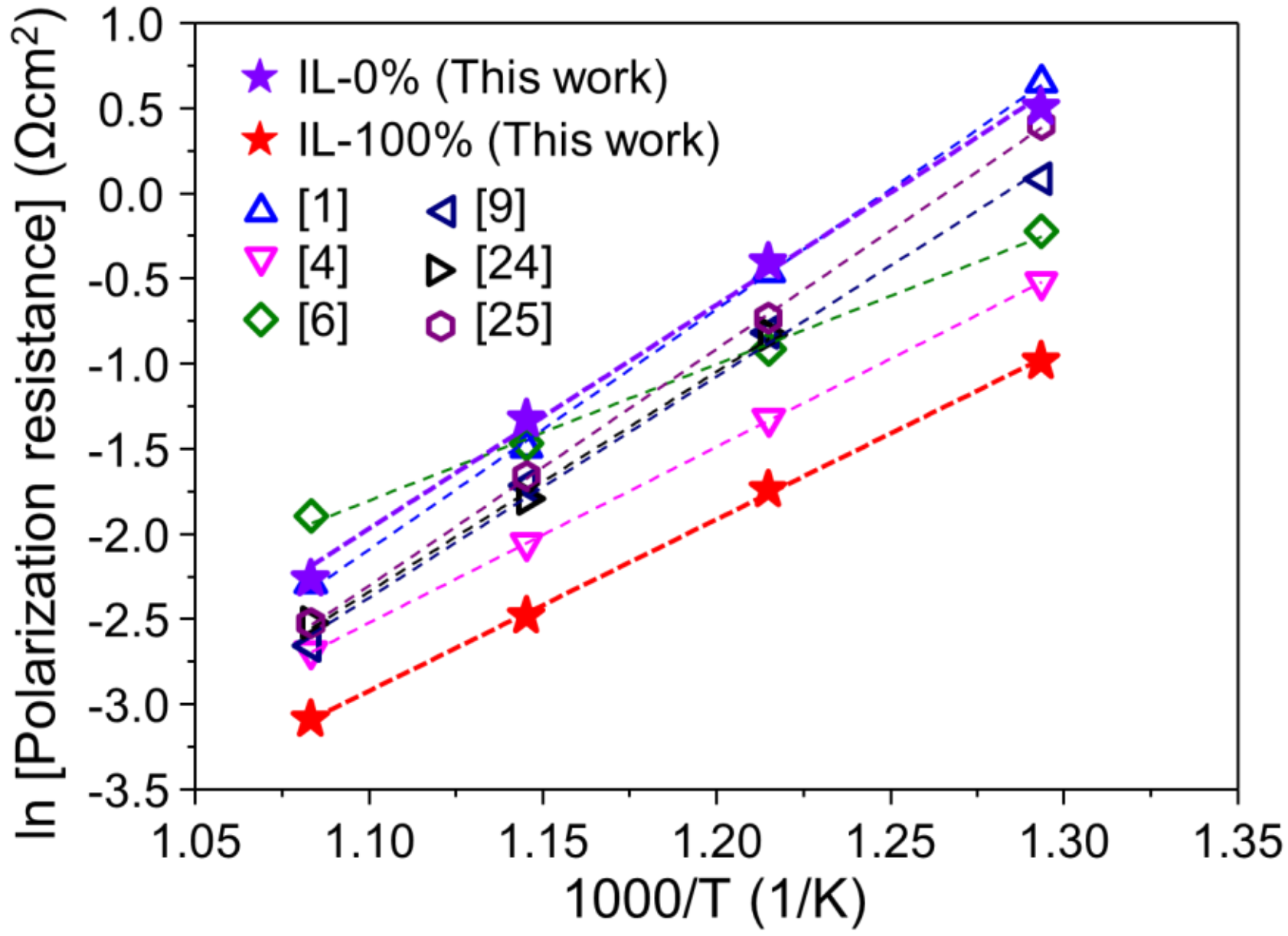

Supplementary Fig. 27. Arrhenius plots of the polarization resistance for IL-0% and IL-100% with recently developed PCECs using PBSCF as an air electrode material in the temperature range of 650–500 °C[1,4,6,9,24,25].

Supplementary Table 9. Comparison of the performance with previously reported PCECs.

Air electrode material	Temperature (°C)	Peak power density (W/cm^2)	Current density at 1.3 V (A/cm^2)	[ref]
PBSCF	500	1.01	1.15	This work (With interlayer)
PBSCF	500	0.48	0.40	This work (Without interlayer)
PBSCF	500	0.69	-	[1]
BSCF	500	0.54	-	[23]
GCCCO+BZCYYb	500	0.48	0.70	[5]
PBSCF	500	0.50	-	[4]
PNC	500	0.31	0.59	[3]
PBSCF	500	0.42	0.21	[24]
BCFZY	500	0.42	-	[26]
PBSCF	500	0.52	0.52	[6]
BCT	500	0.75	0.43	[27]
BCFN	500	0.50	0.30	[28]
BSC+PBSCF	500	0.82	-	[2]
	450	0.53	0.65	
BCFZY	500	-	0.62	[29]
BCFN	500	-	0.52	[30]
NCCO	500	0.48	1.04	[31]

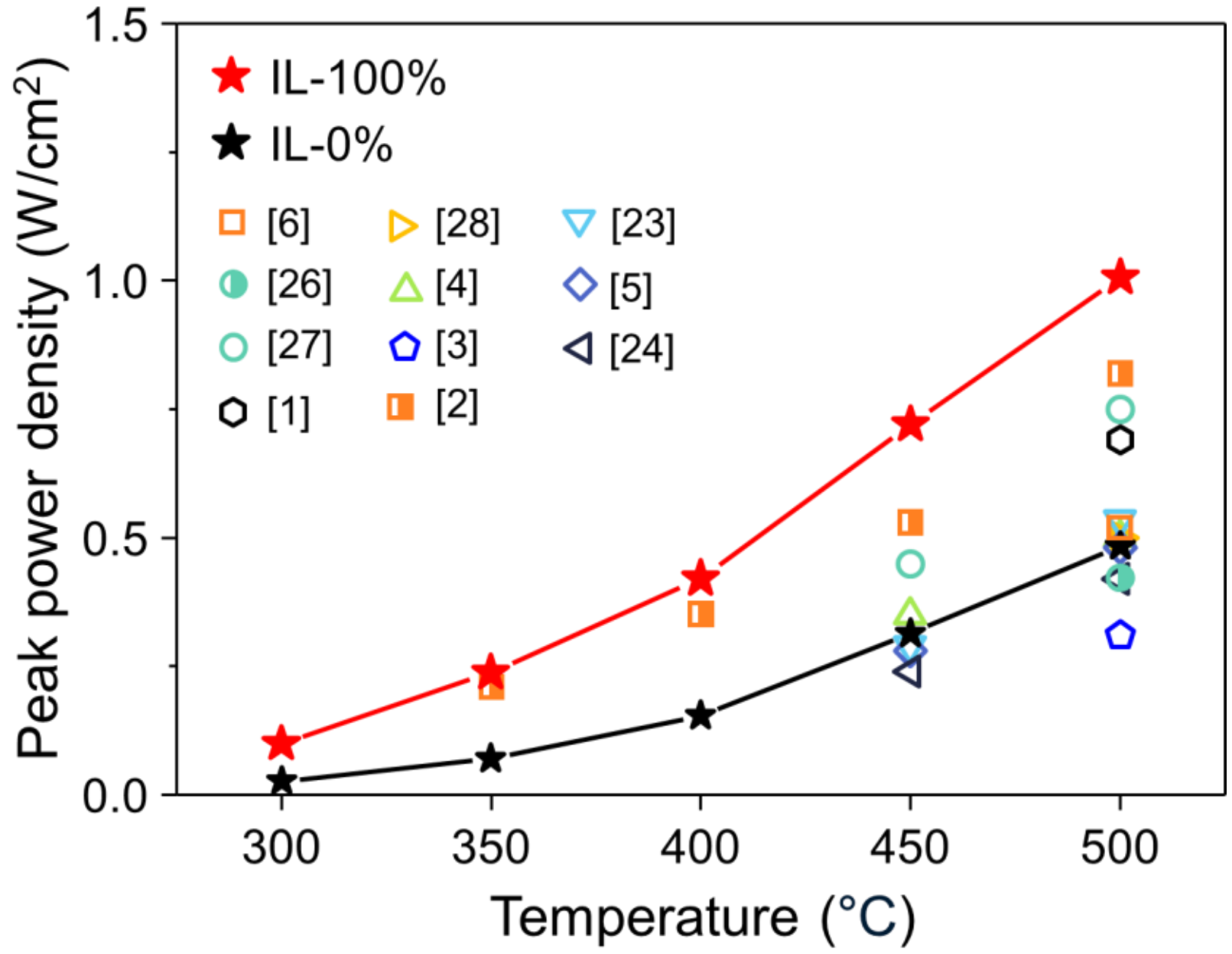

Supplementary Fig. 28. Comparison of peak power densities with previously reported PCECs.[1-6,23,24,26-28]

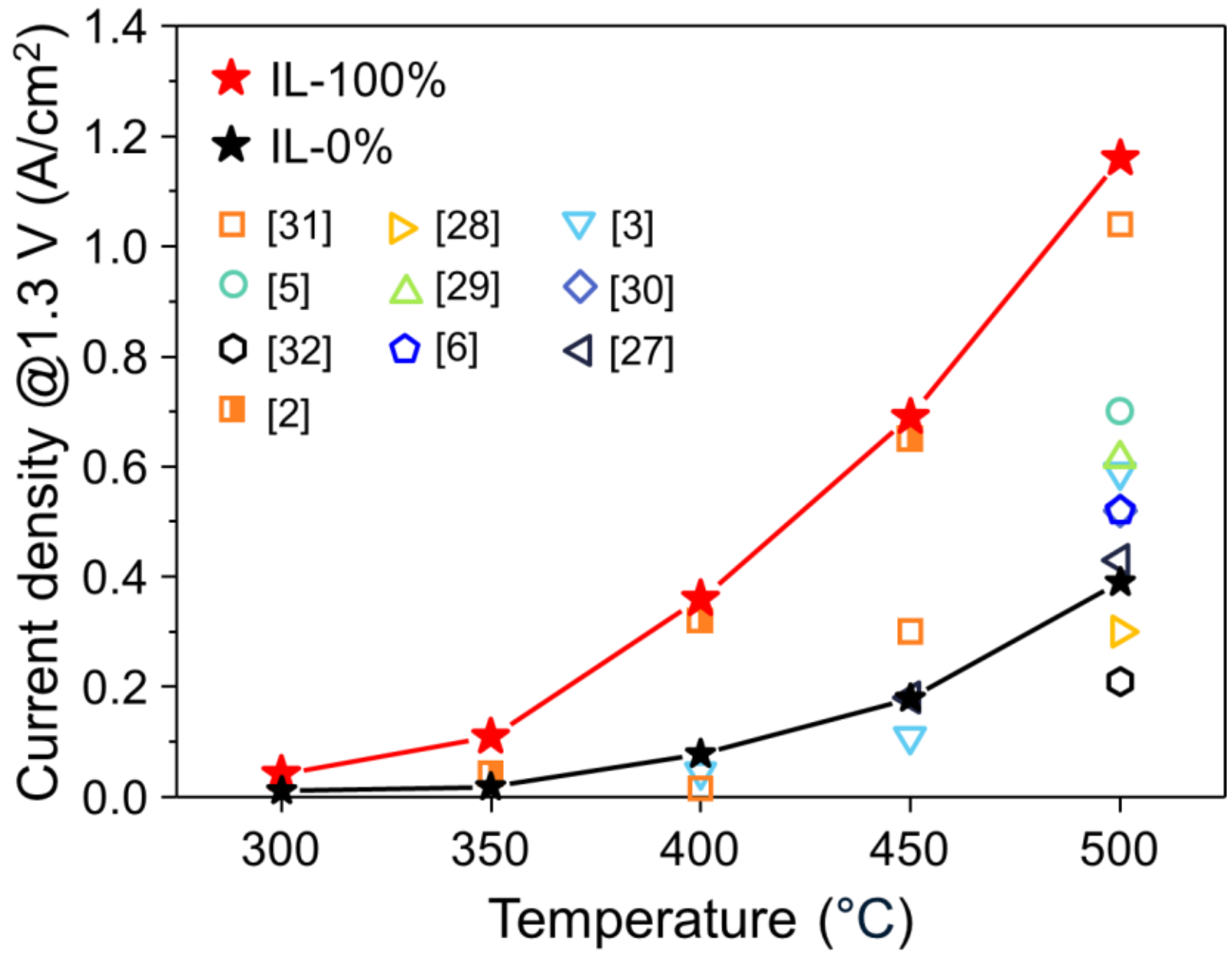

Supplementary Fig. 29. Comparison of the current densities at 1.3 V with previously reported PCECs.[2,3,5,6,27-32]

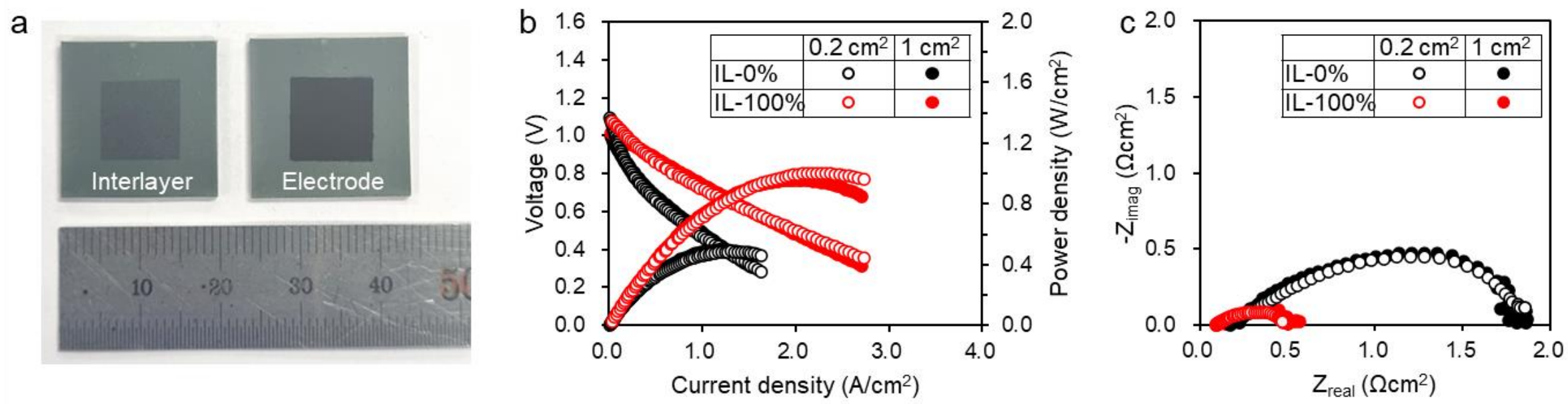

Supplementary Fig. 30. (a) Optical images of PCECs (cell size: 2 × 2 cm^2; active area: 1 × 1 cm^2) after deposition of the PBSCF interlayer and PBSCF electrode. (b) I-V curves of IL-0% and IL-100% at 500 °C and (c) the corresponding Nyquist plots measured under OCV conditions, comparing cells with active areas of 0.2 and 1 cm^2. Source data for Supplementary Fig. 30 are provided as a Source Data file.

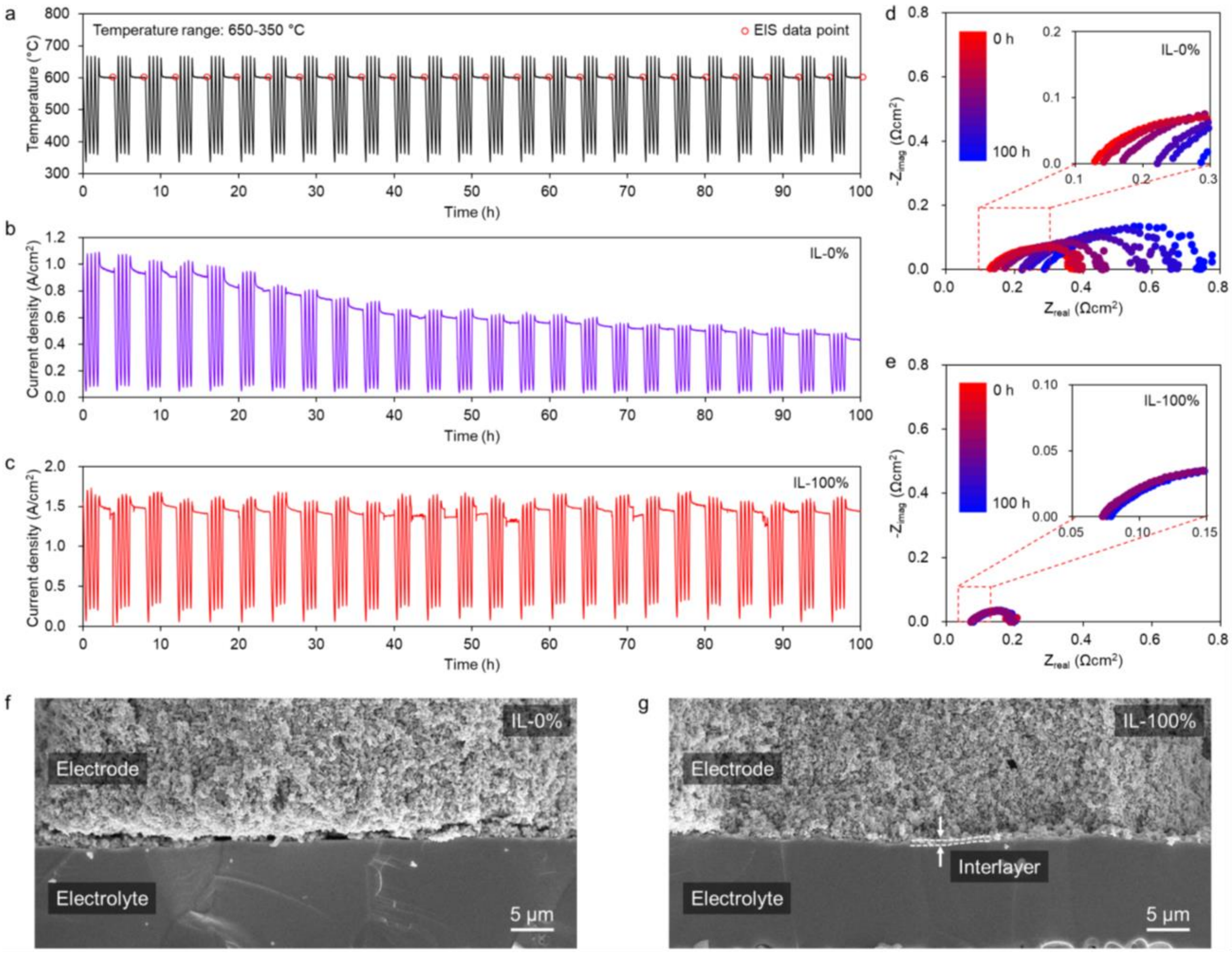

Supplementary Fig. 31. Thermal cycling behavior of larger area PCECs (cell size: 2 × 2 cm^2; effective area: 1 × 1 cm^2). (a) Thermal cycling temperature profile and corresponding output current density under a constant voltage of 0.8 V for (b) IL-0% and (c) IL-100%. Nyquist plots at 600 °C under OCV conditions during thermal cycling for (d) IL-0% and (e) IL-100%. Cross-sectional SEM images at the electrode/electrolyte interface after thermal cycling for (f) IL-0% and (g) IL-100%. Source data for Supplementary Fig. 31 are provided as a Source Data file.

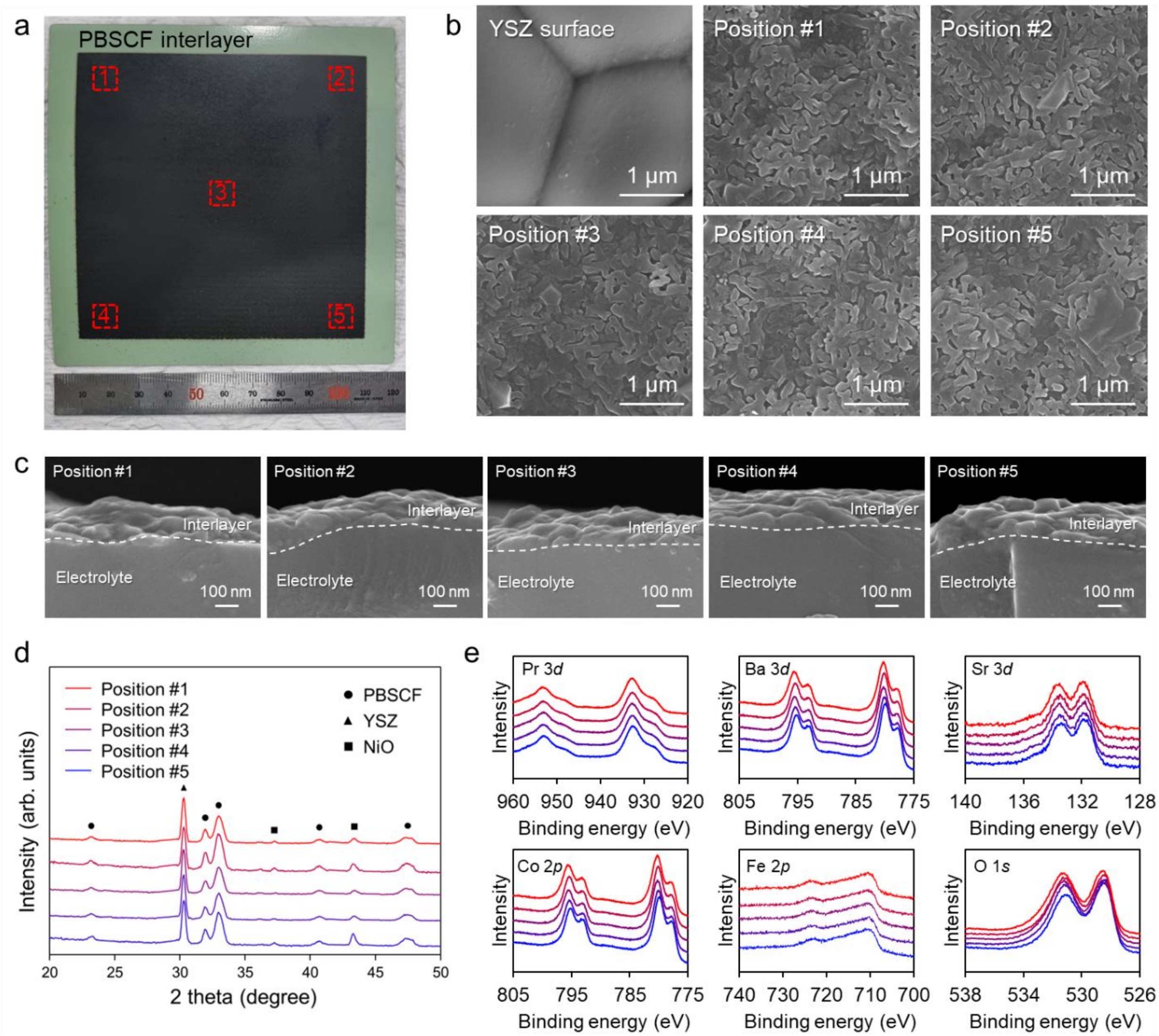

Supplementary Fig. 32. Structural and chemical uniformity of the PBSCF interlayer over an area of 100 cm². (a) Optical image of the PBSCF interlayer. The red dashed boxes indicate the five representative locations selected for the subsequent SEM, XRD, and XPS analyses. (b) Surface SEM images of the YSZ substrate and the PBSCF interlayer. (c) Cross-sectional SEM images of the PBSCF interlayer. (d) XRD patterns of the PBSCF interlayer. (e) XPS spectra of Pr 3*d*, Ba 3*d*, Sr 3*d*, Co 2*p*, Fe 2*p*, and O 1*s* acquired at the PBSCF interlayer. Panels (d) and (e) share the same legend. Source data for Supplementary Fig. 32 are provided as a Source Data file.

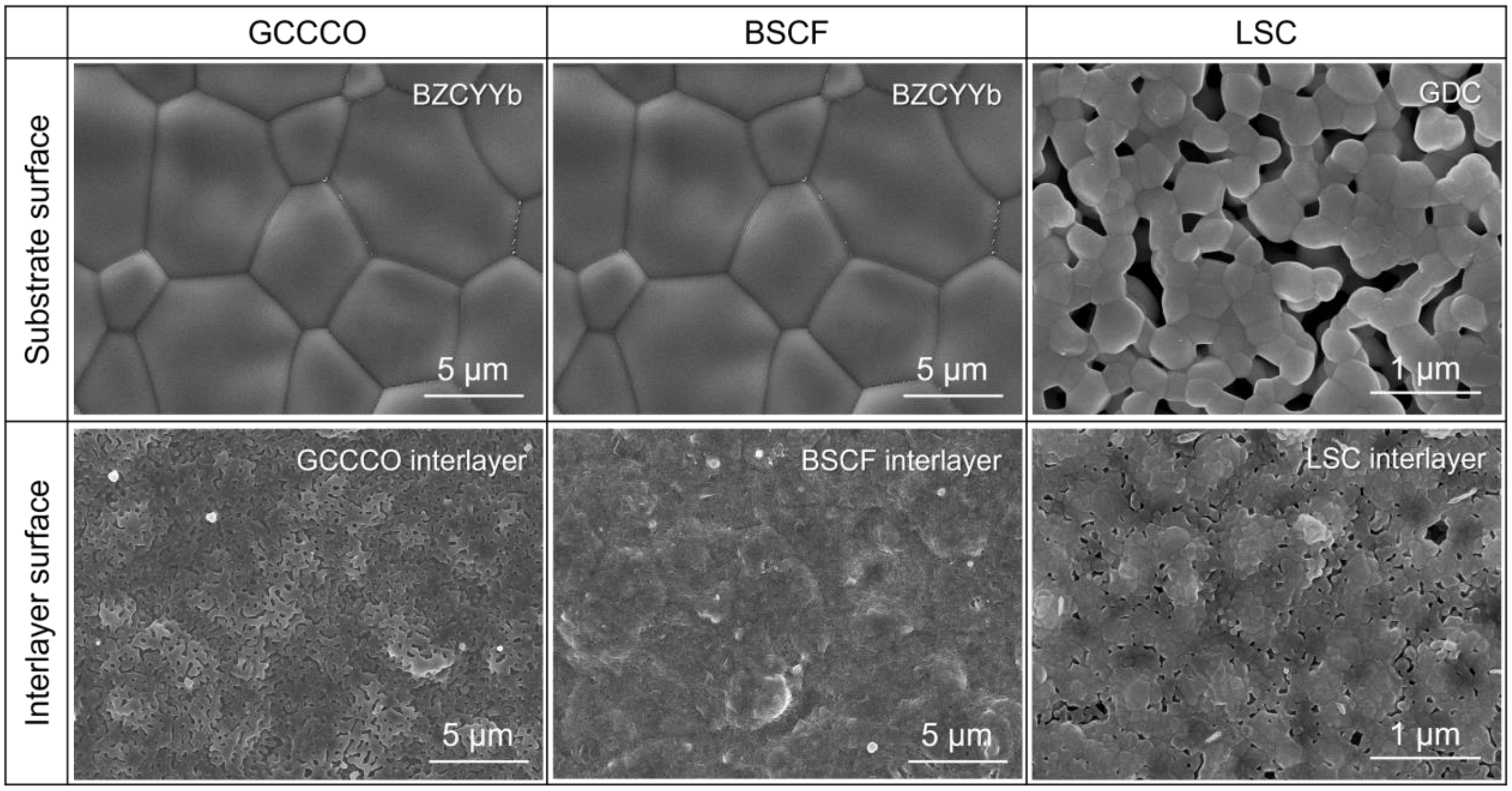

Supplementary Fig. 33. Surface SEM images of the substrates and the precursor-based interlayers.

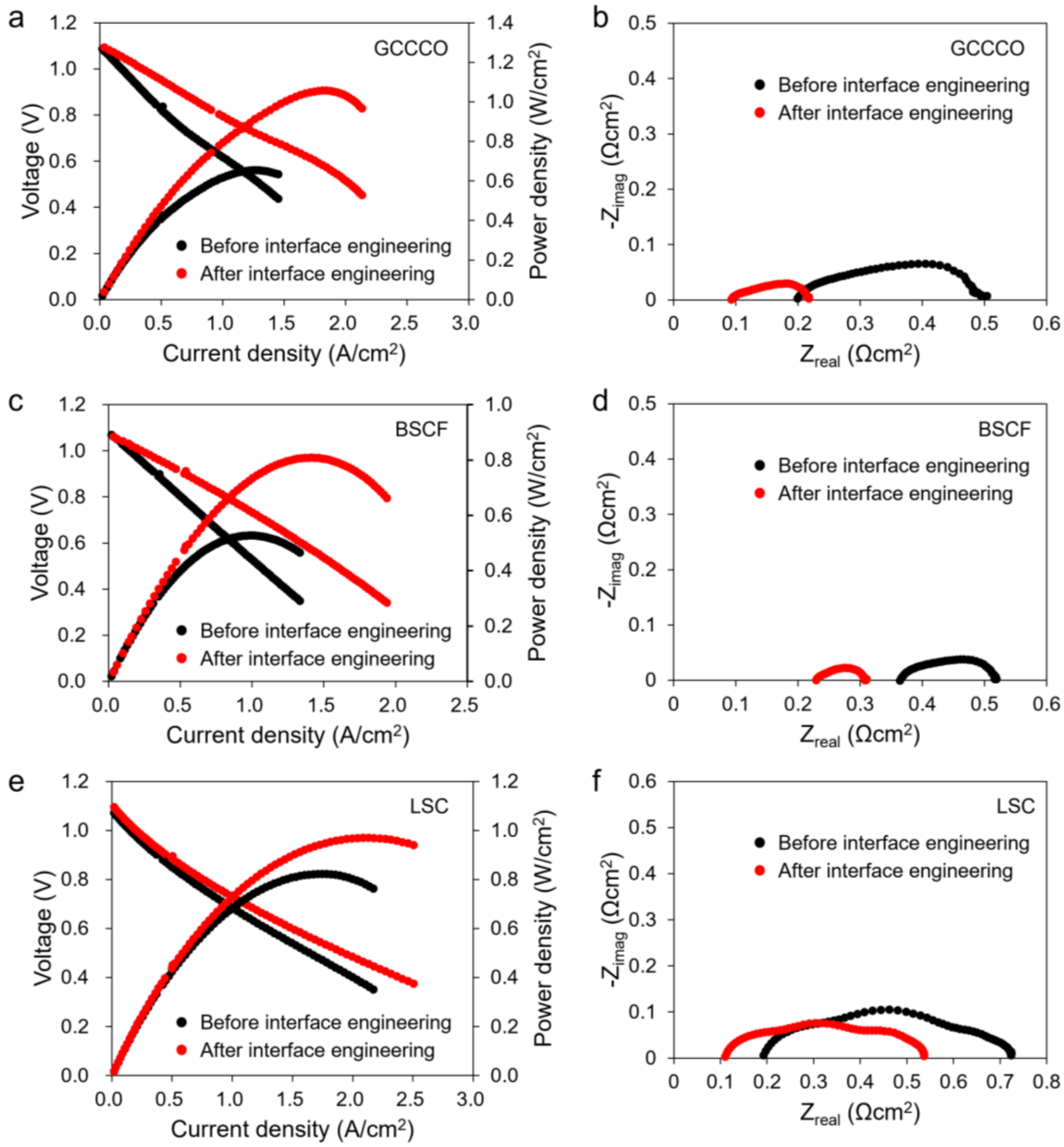

Supplementary Fig. 34. (a) I-V-P curves of the GCCCO interlayer in a Ni-BZCYYb/BZCYYb/GCCCO configuration at 600 °C and (b) Nyquist plots under OCV conditions. (c) I-V-P curves of the BSCF interlayer in a Ni-BZCYYb/BZCYYb/BSCF configuration at 600 °C and (d) Nyquist plots under OCV conditions. (e) I-V-P curves of the LSC interlayer in a Ni-YSZ/YSZ/GDC/LSC configuration at 650 °C and (f) Nyquist plots under OCV conditions. Source data for Supplementary Fig. 34 are provided as a Source Data file.

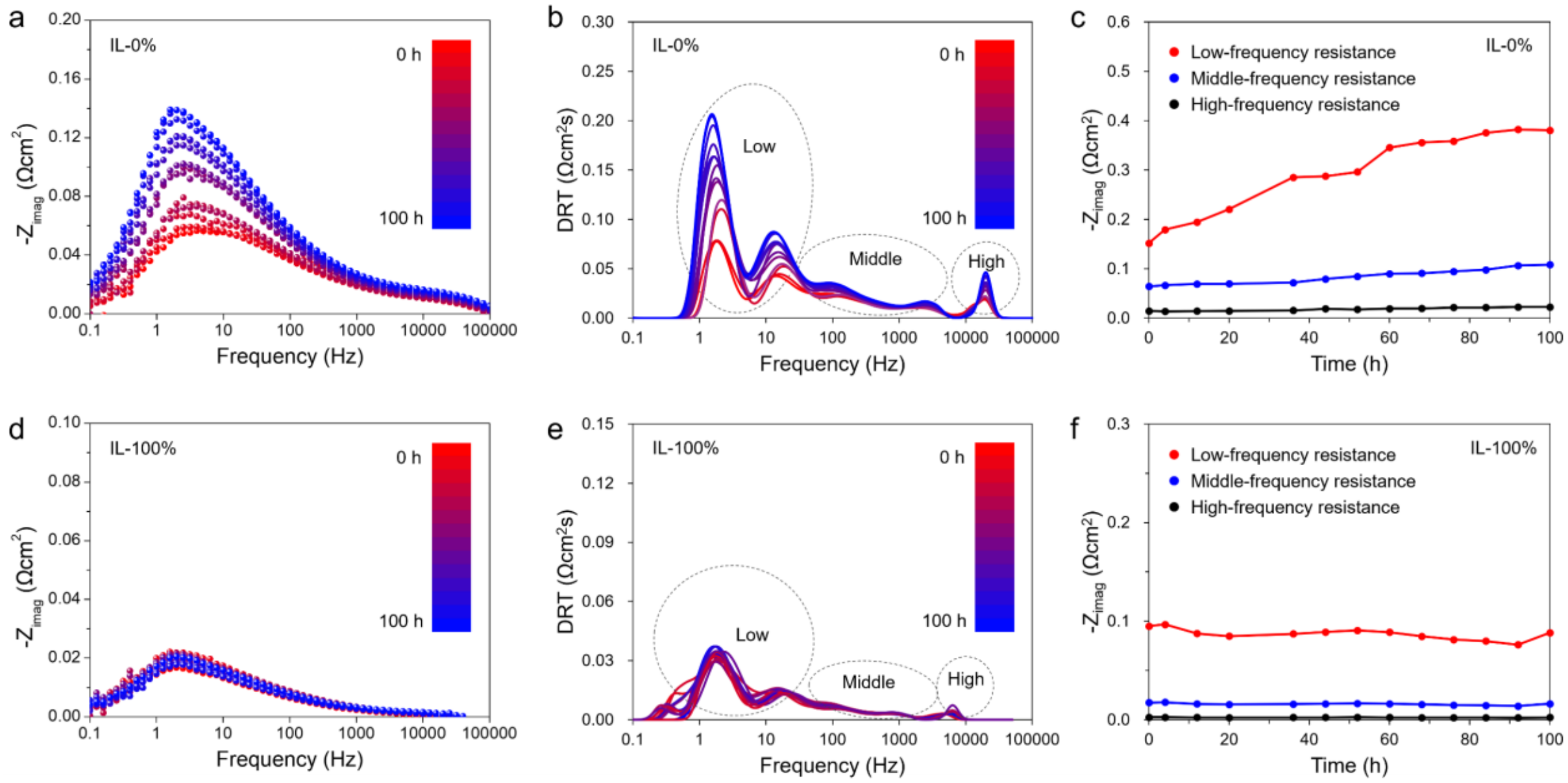

Supplementary Fig. 35. (a) Bode plots, (b) DRT analysis, and (c) the corresponding polarization resistances of low-, middle-, high-frequency regions during thermal cycling at 600 °C under OCV conditions of IL-0%. (d) Bode plots, (e) DRT analysis, and (f) the corresponding polarization resistances of low-, middle-, high-frequency regions during thermal cycling at 600 °C under OCV conditions of IL-100%. Source data for Supplementary Fig. 35 are provided as a Source Data file.

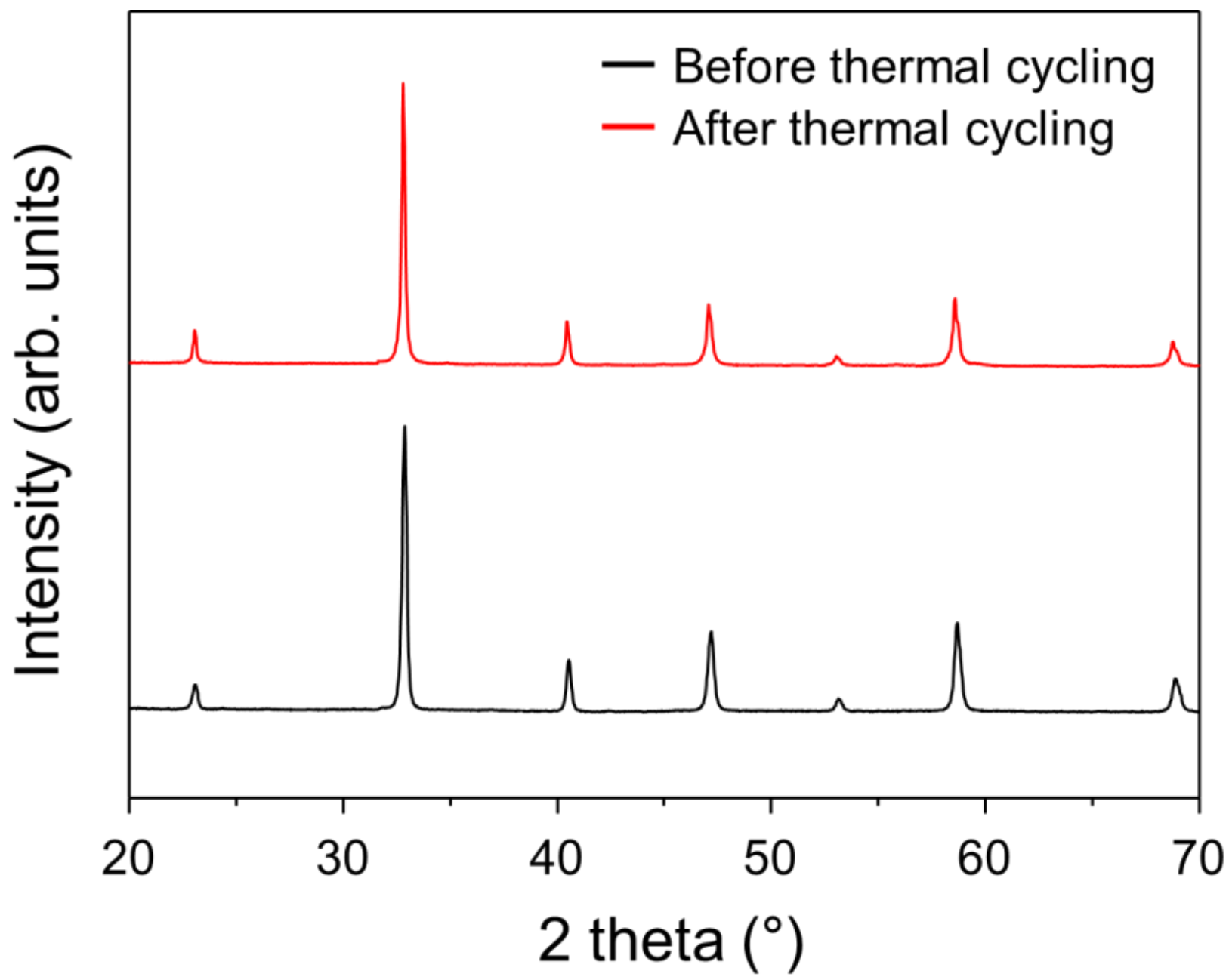

Supplementary Fig. 36. XRD patterns of the PBSCF electrode surfaces before/after thermal cycling tests. Source data for Supplementary Fig. 36 are provided as a Source Data file.

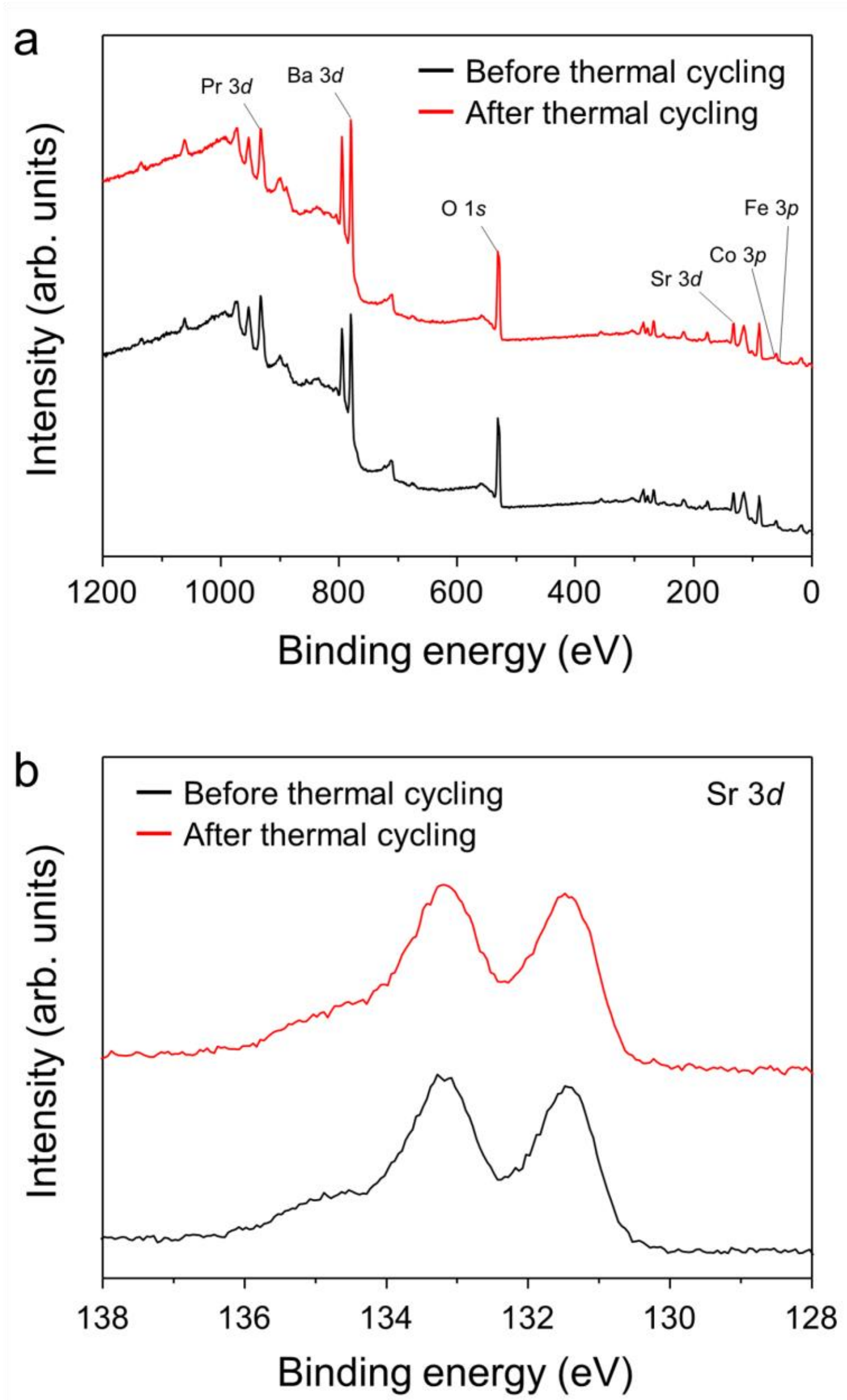

Supplementary Fig. 37. (a) XPS survey spectra and (b) XPS spectra of the Sr 3*d* peaks of the PBSCF electrode surfaces before/after thermal cycling tests. Source data for Supplementary Fig. 37 are provided as a Source Data file.

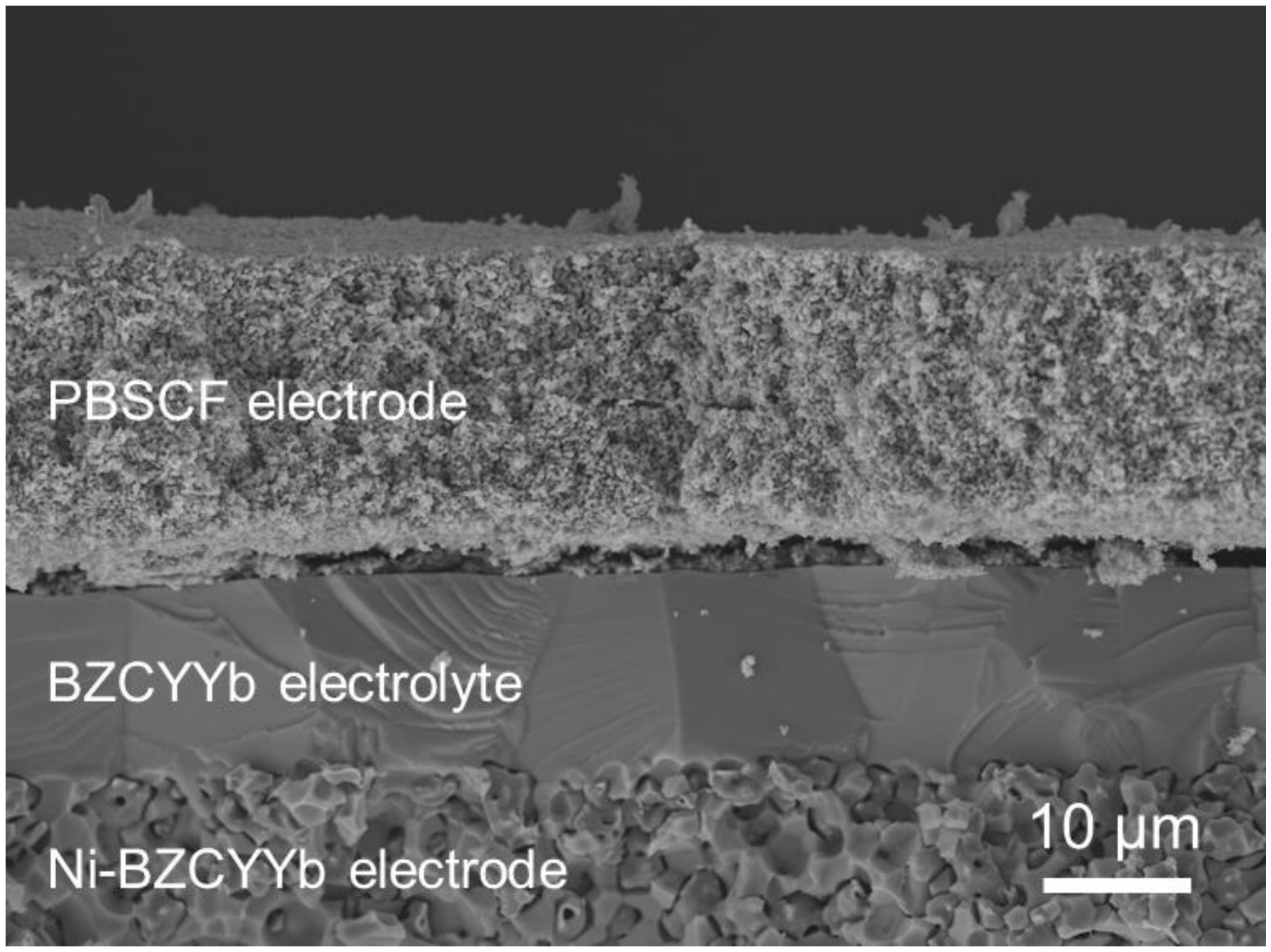

Supplementary Fig. 38. Cross-sectional SEM image of the IL-0% electrolyte after thermal cycling tests.

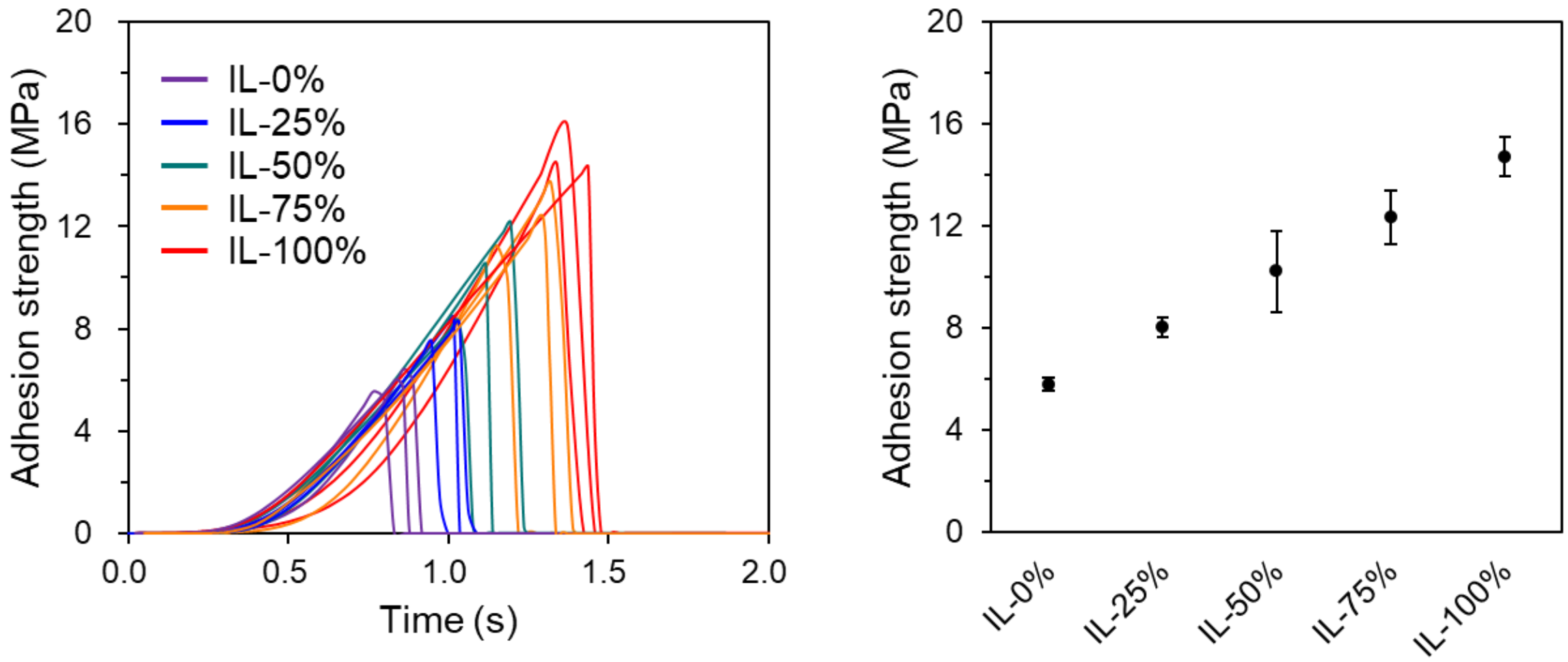

Supplementary Fig. 39. Adhesion strengths at the interfaces of IL-0%, IL-25%, IL-50%, IL-75%, and IL-100% using the mechanical pull-off test. Each sample was tested three times to ensure reliability and reproducibility, and the error bars represent the standard deviation. Source data for Supplementary Fig. 39 are provided as a Source Data file.

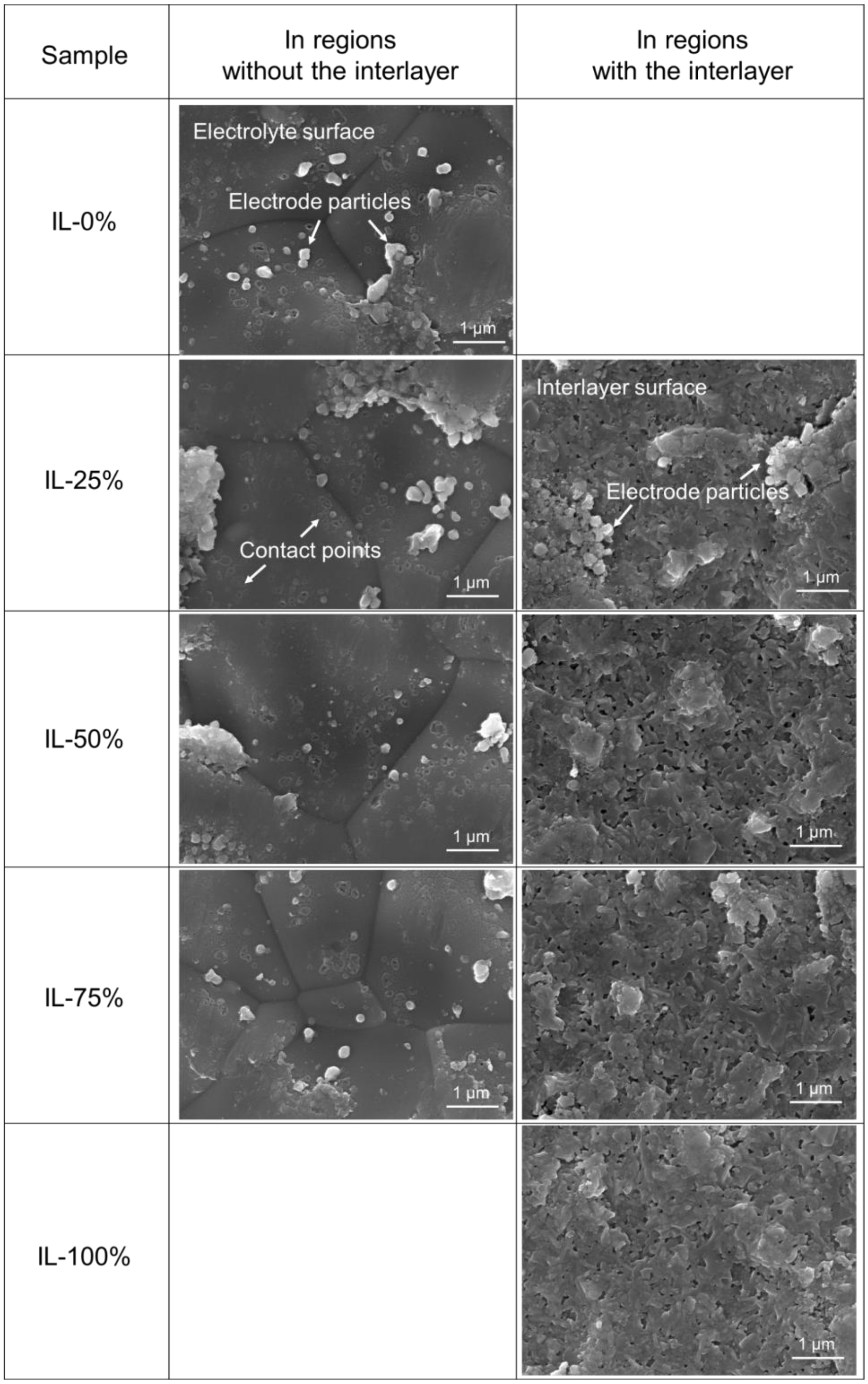

Supplementary Fig. 40. Post-test fracture surface SEM images of IL-0%, IL-25%, IL-50%, IL-75%, and IL-100%.

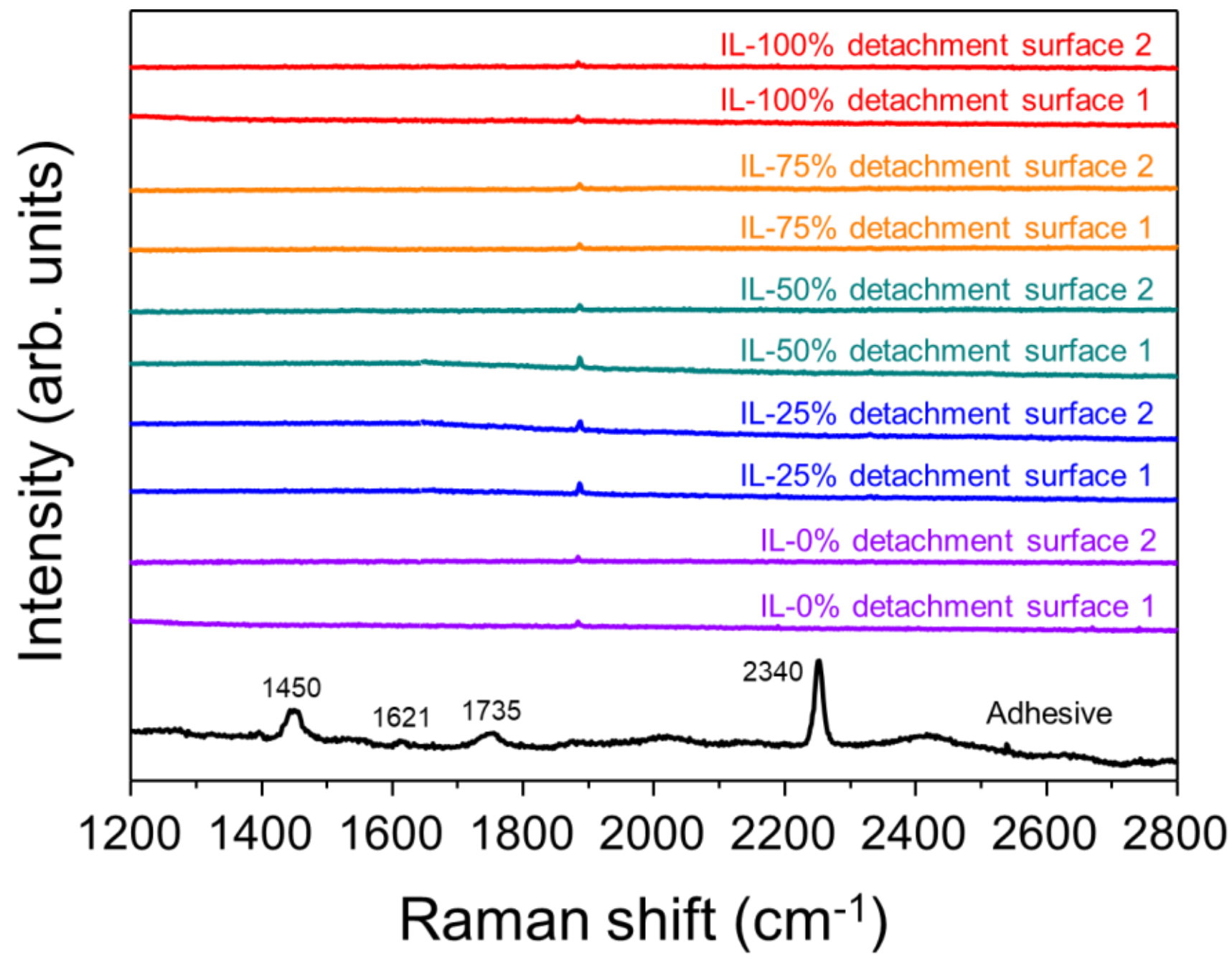

Supplementary Fig. 41. Raman spectroscopy of the detached surfaces after the pull-off test and adhesive. The detachment surfaces on the electrolyte side and the electrode side were designated as detachment surface 1 and 2, respectively. Source data for Supplementary Fig. 41 are provided as a Source Data file.

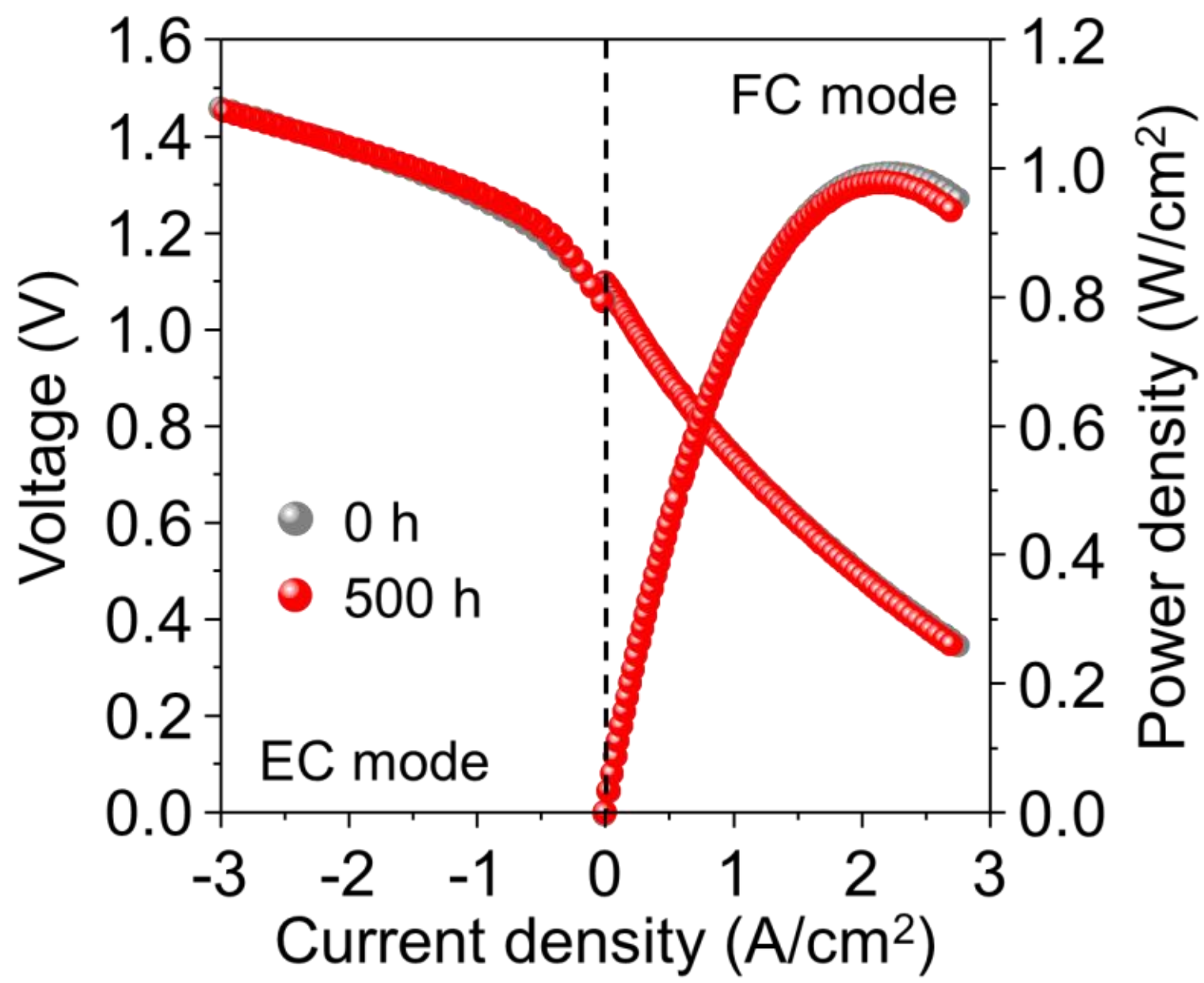

Supplementary Fig. 42. I-V-P curve of the fuel cell and electrolysis cell modes at 500 ˚C before and after the stability test for 500 h. Source data for Supplementary Fig. 42 are provided as a Source Data file.

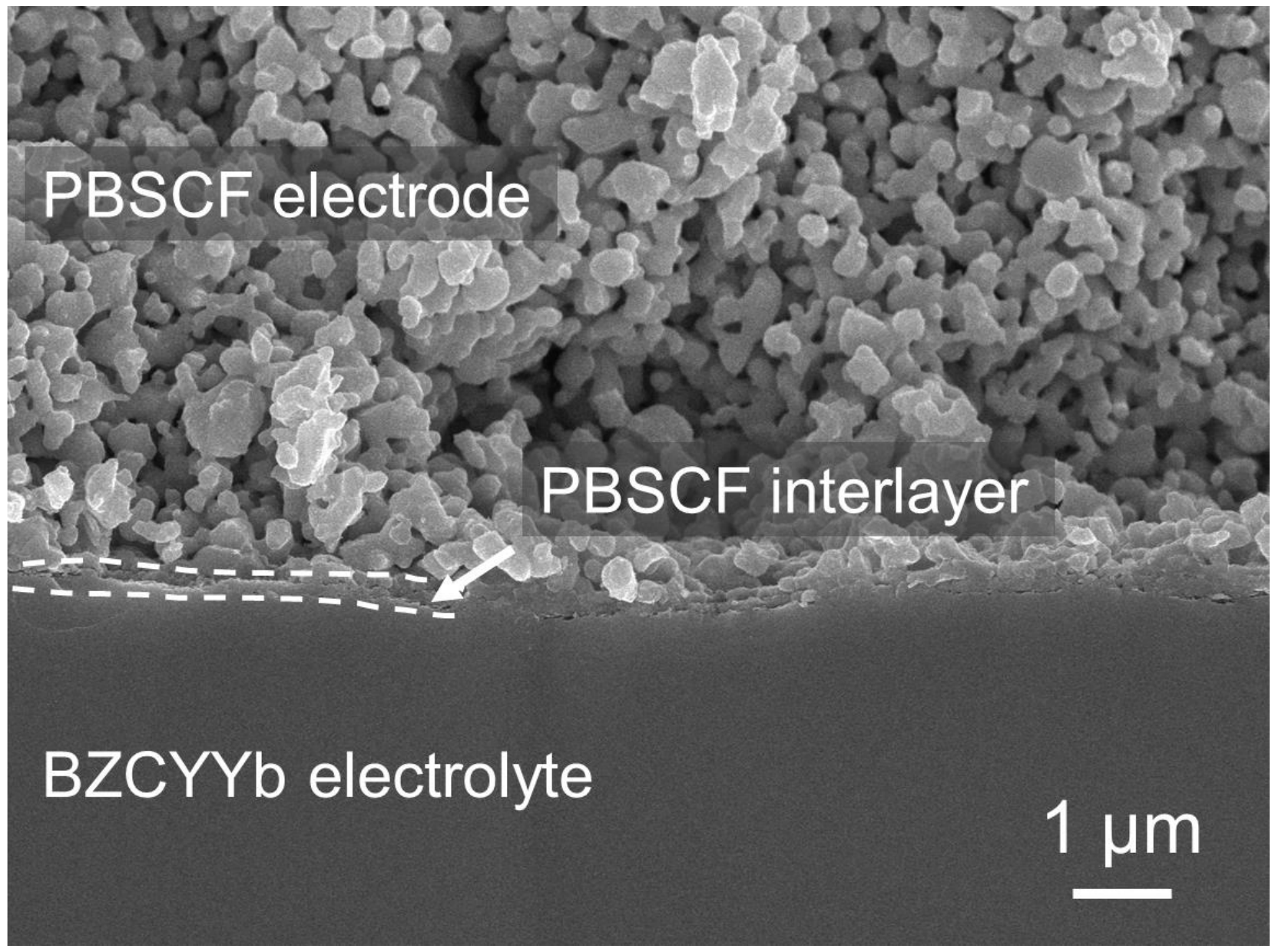

Supplementary Fig. 43. Cross-sectional SEM image of IL-100% after the stability test.

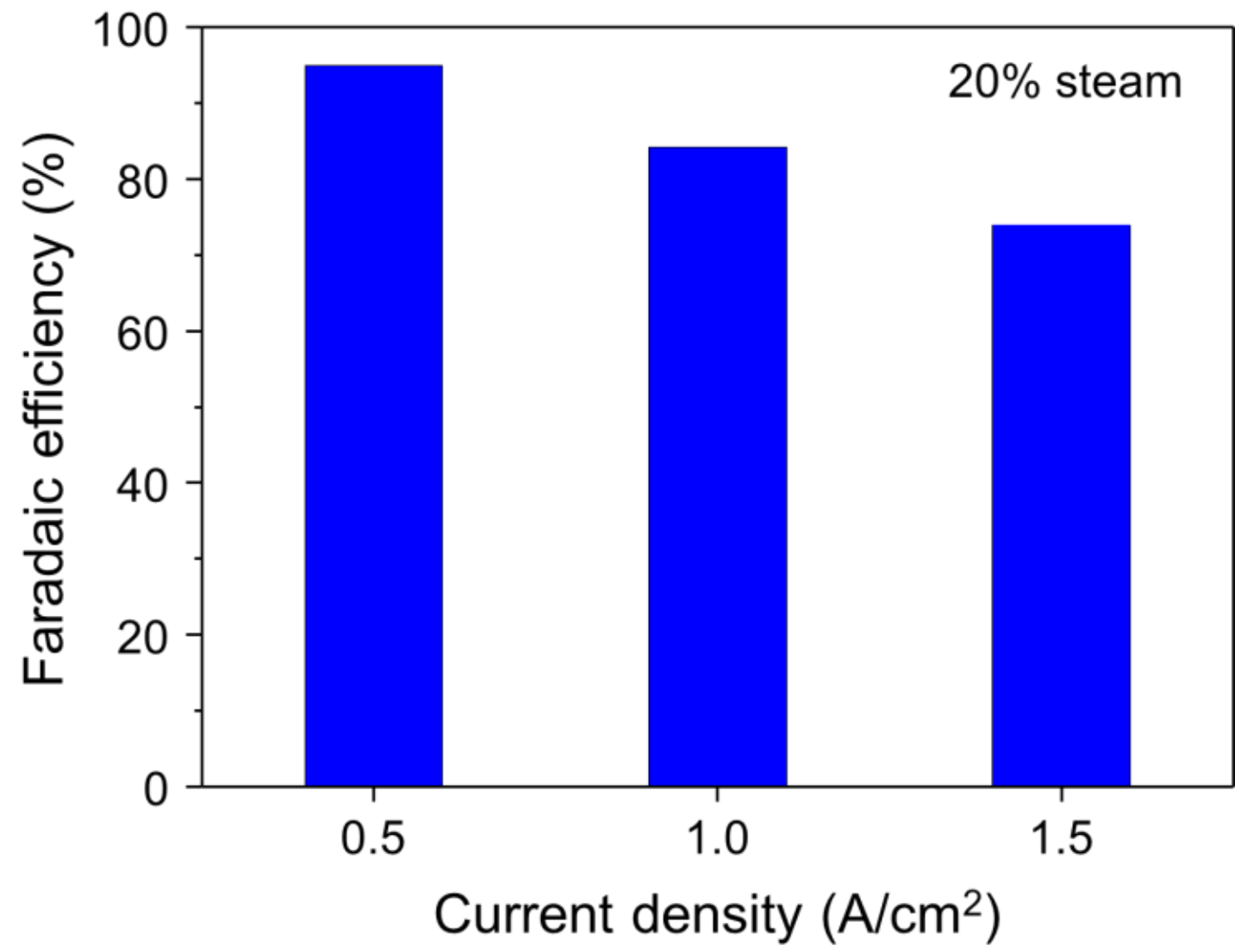

Supplementary Fig. 44. Faradaic efficiency of IL-100% evaluated at different current densities under 20% steam (air electrode) conditions at 500 °C. Source data for Supplementary Fig. 44 are provided as a Source Data file.

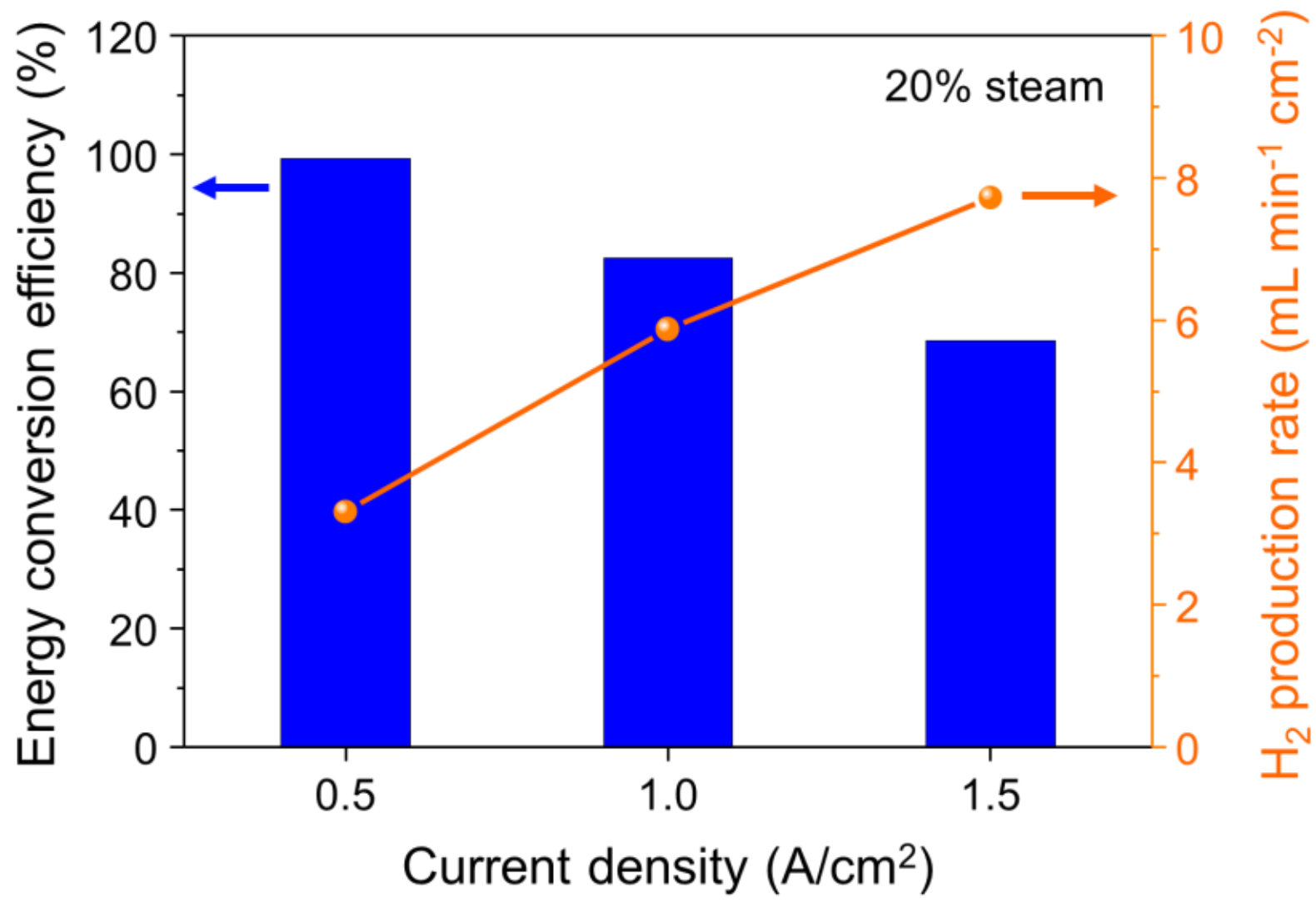

Supplementary Fig. 45. Energy conversion efficiency and hydrogen production rate of IL-100% evaluated at different current densities under 20% steam (air electrode) at 500 °C. Source data for Supplementary Fig. 45 are provided as a Source Data file.

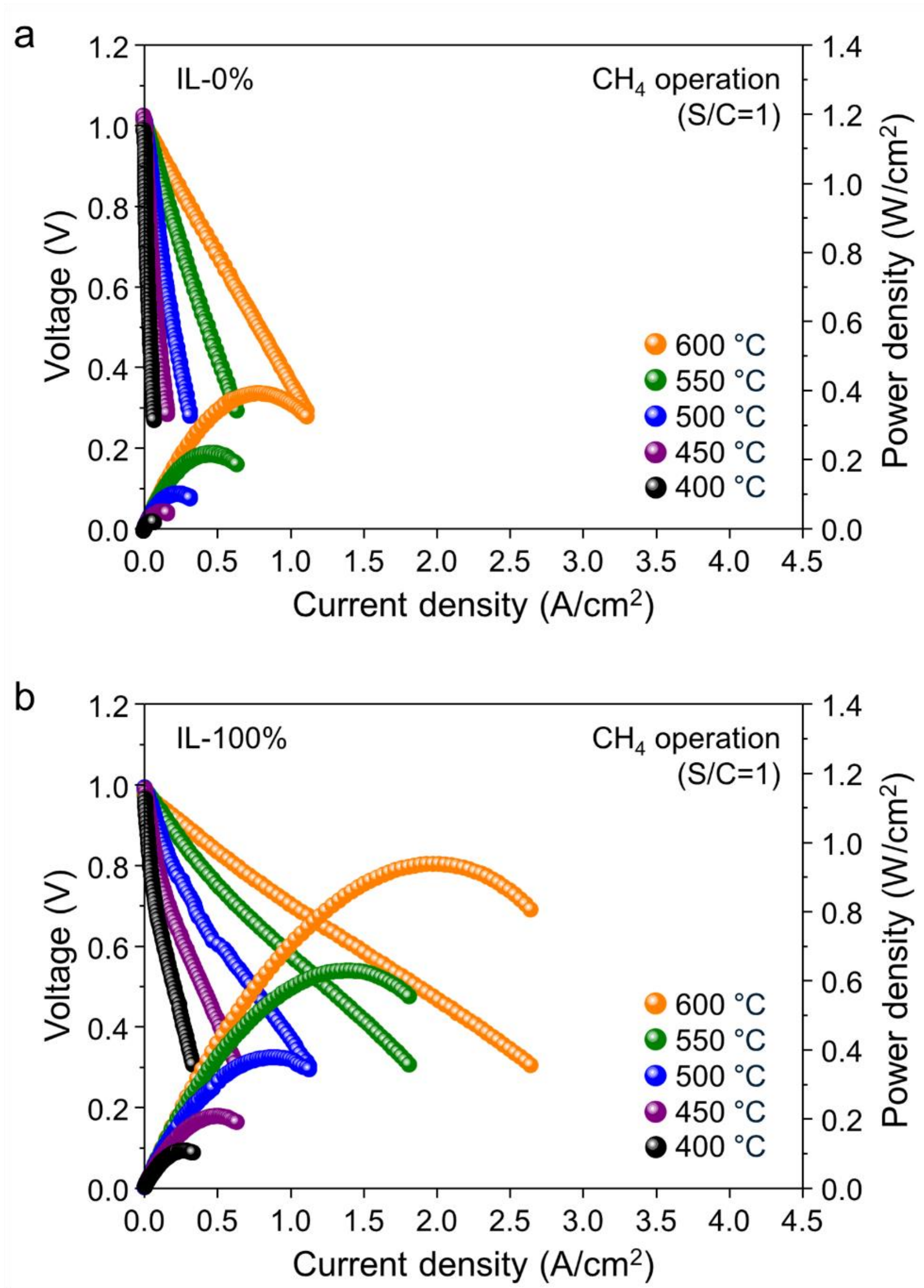

Supplementary Fig. 46. I-V-P curve of the fuel cell mode using methane as a fuel with a steam-to-carbon ratio (S/C) of 1 for (a) IL-0% and (b) IL-100% in the temperature range of 600–400 °C. Source data for Supplementary Fig. 46 are provided as a Source Data file.

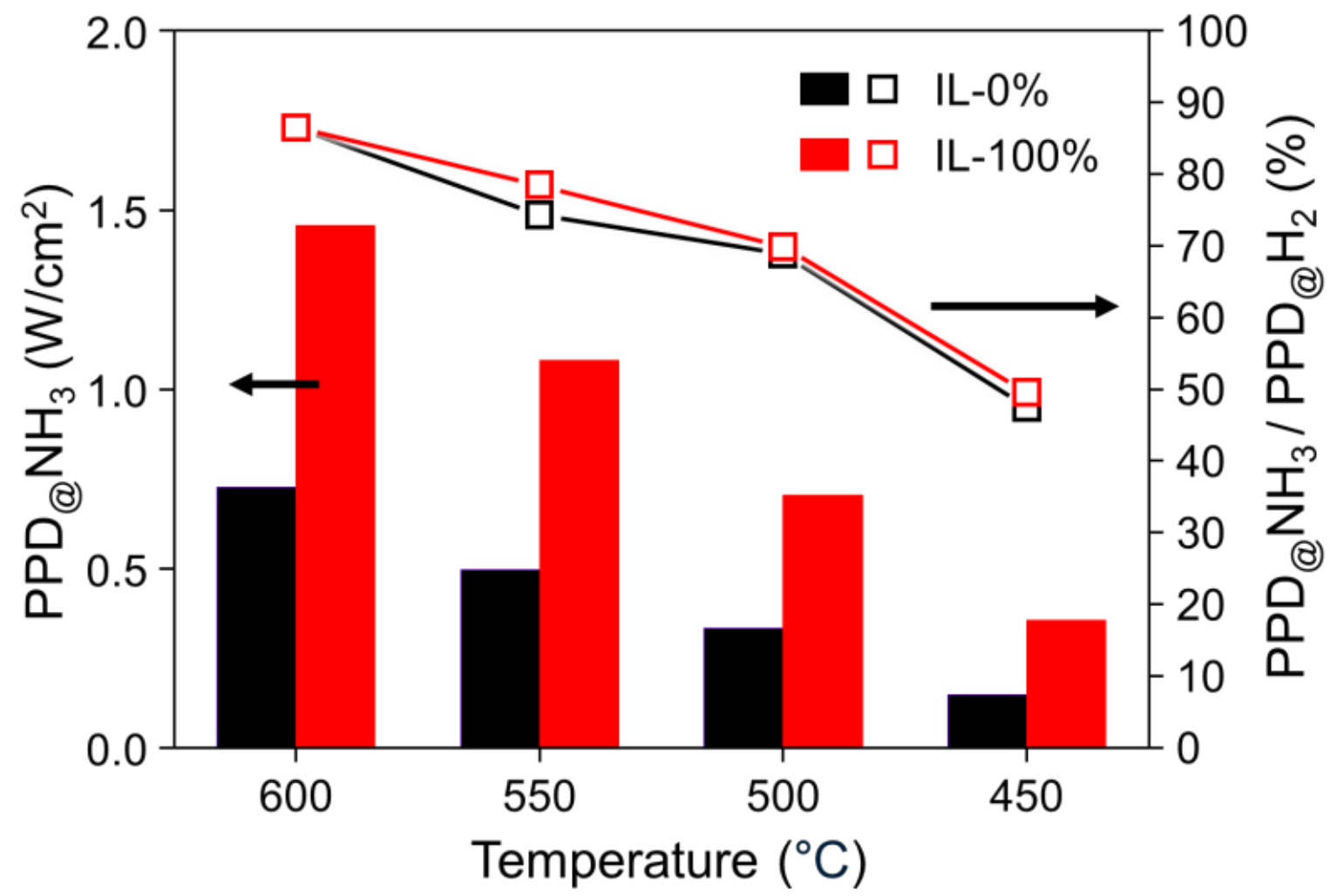

Supplementary Fig. 47. The peak power density at ammonia operation (bar) of IL-0% and IL-100% and the peak power density ratio between ammonia and hydrogen (line) in the temperature range of 600−450 °C. Source data for Supplementary Fig. 47 are provided as a Source Data file.

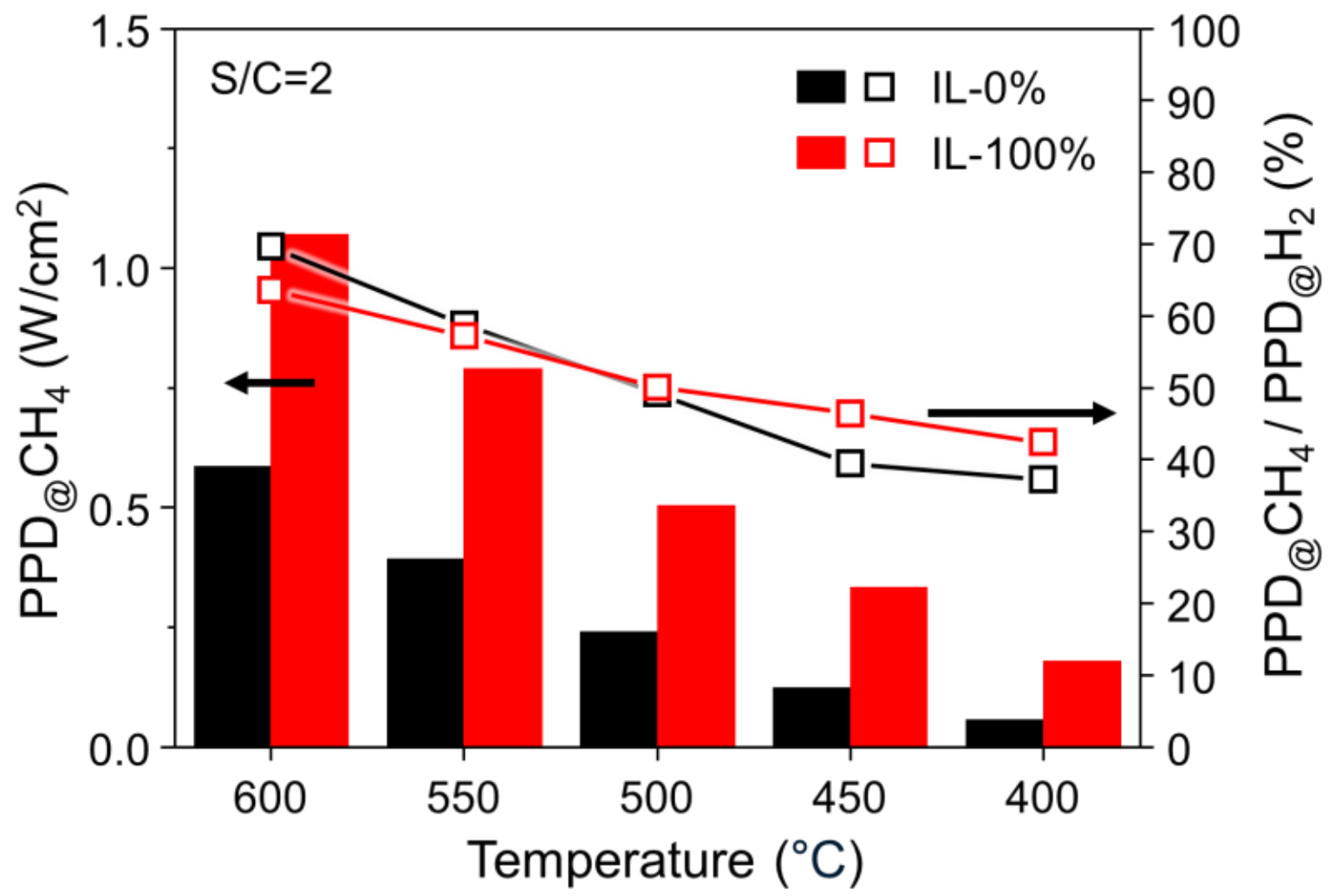

Supplementary Fig. 48. The peak power density at methane operation (bar) of IL-0% and IL-100% and the peak power density ratio between methane and hydrogen (line) in the temperature range of 600–450 °C. Source data for Supplementary Fig. 48 are provided as a Source Data file.

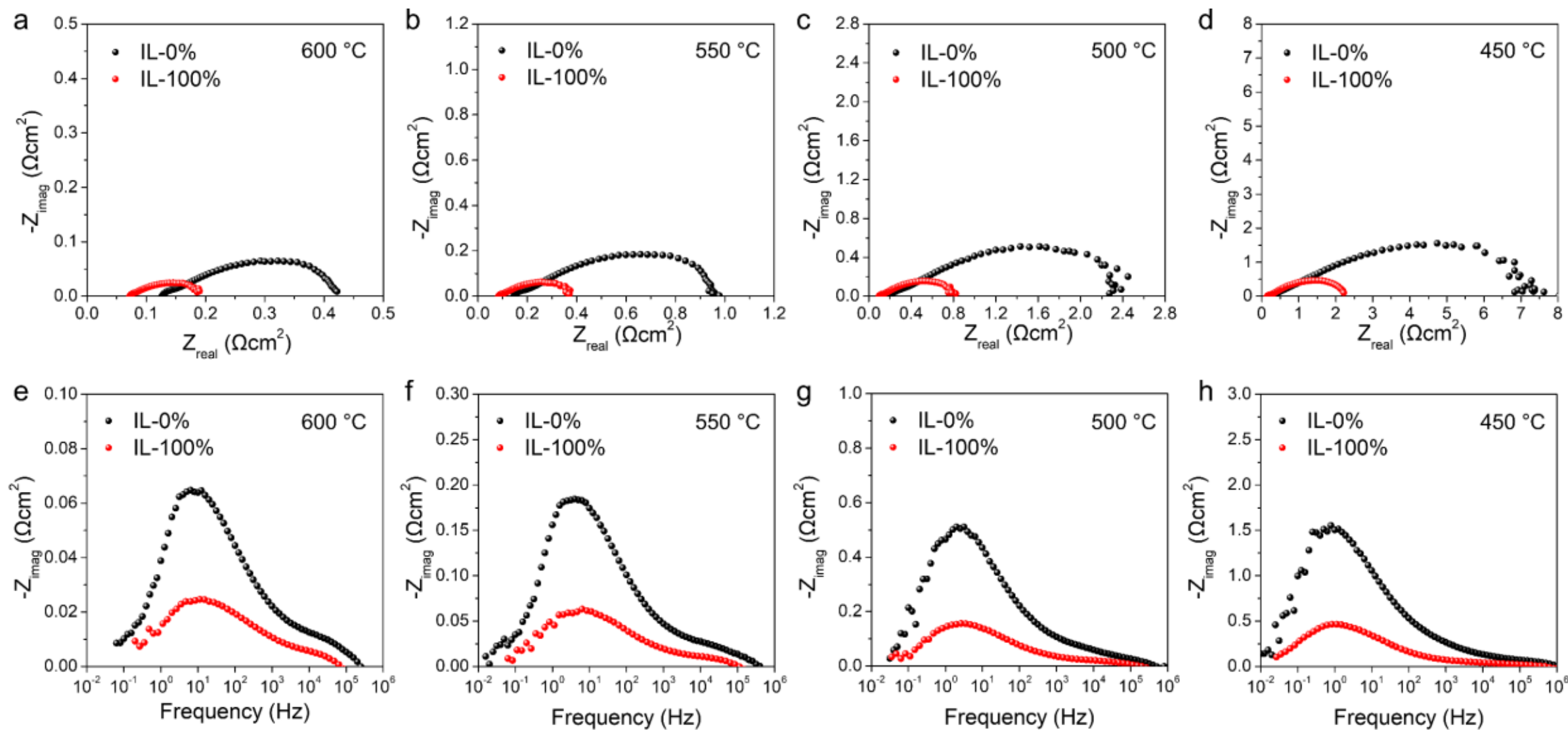

Supplementary Fig. 49. Nyquist plots of IL-0% and IL-100% under NH_3 operation at OCV conditions at (a) 600 °C, (b) 550 °C, (c) 500 °C, and (d) 450 °C. Bode plots of IL-0% and IL-100% under NH_3 operation at OCV conditions at (a) 600 °C, (b) 550 °C, (c) 500 °C, and (d) 450 °C. Source data for Supplementary Fig. 49 are provided as a Source Data file.

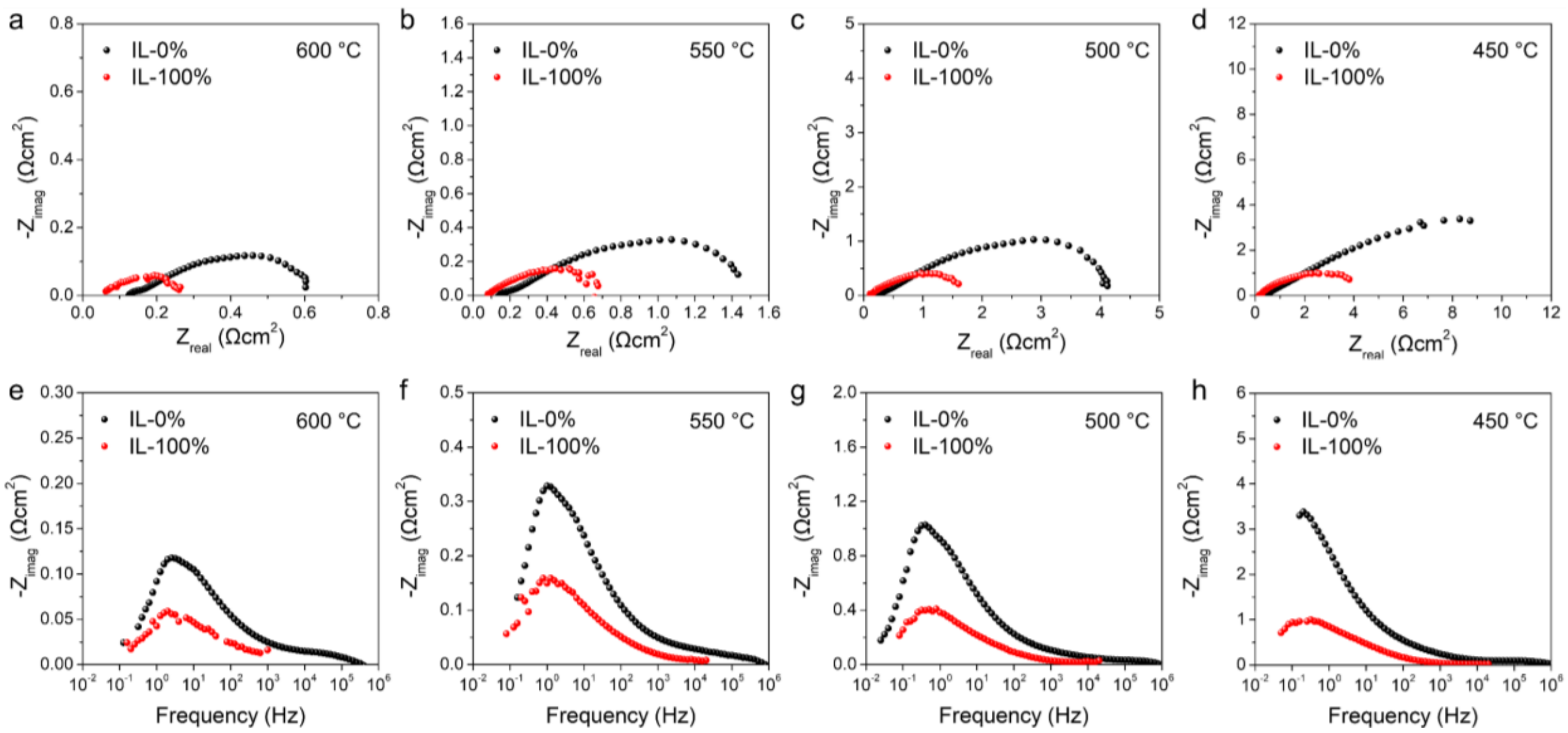

Supplementary Fig. 50. Nyquist plots of IL-0% and IL-100% under CH_4 operation (S/C=2) at OCV conditions at (a) 600 °C, (b) 550 °C, (c) 500 °C, and (d) 450 °C. Bode plots of IL-0% and IL-100% under CH_4 operation (S/C=2) at OCV conditions at (a) 600 °C, (b) 550 °C, (c) 500 °C, and (d) 450 °C. Source data for Supplementary Fig. 50 are provided as a Source Data file.

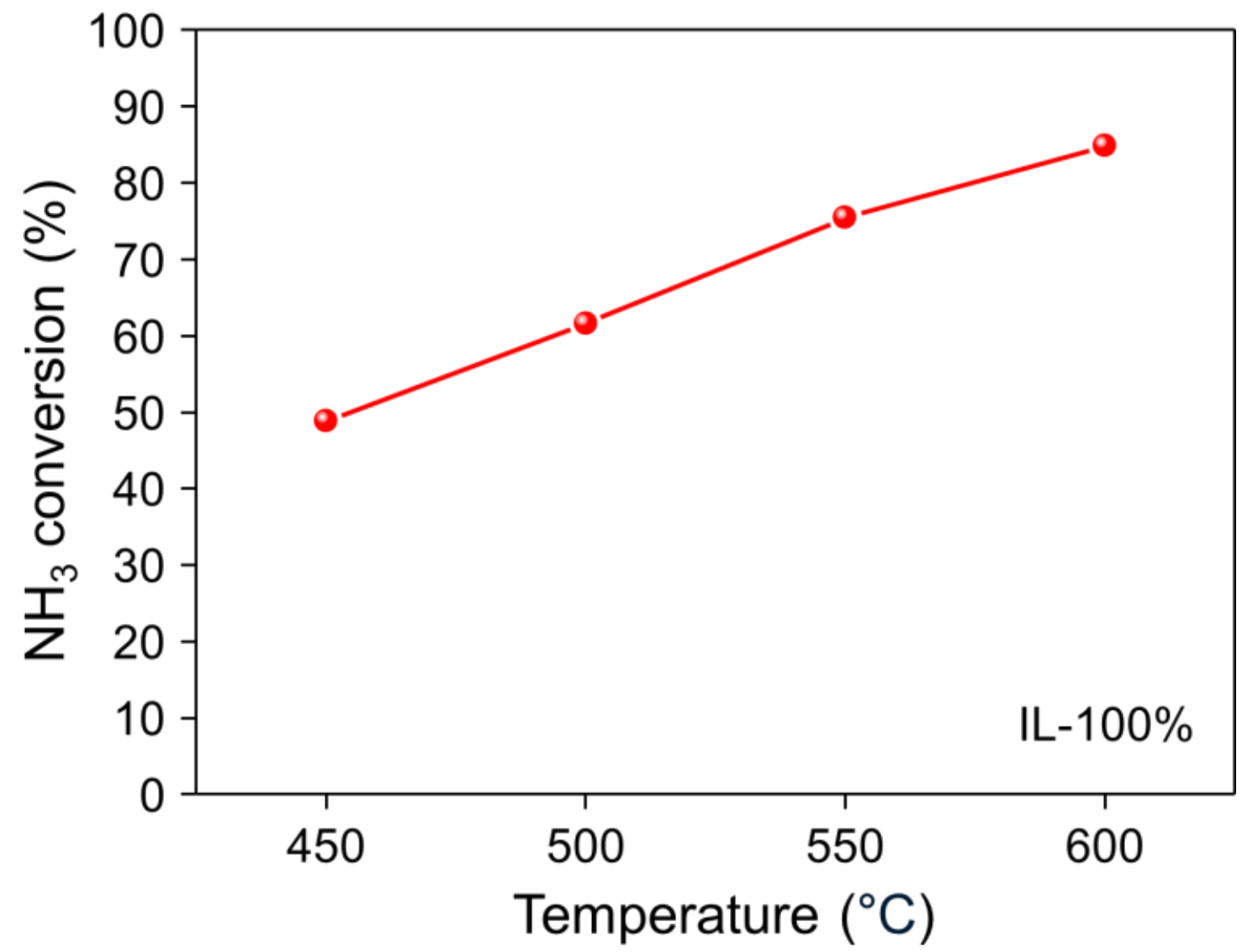

Supplementary Fig. 51. Ammonia conversion of IL-100% in the temperature range of 450–600 °C. Source data for Supplementary Fig. 51 are provided as a Source Data file.

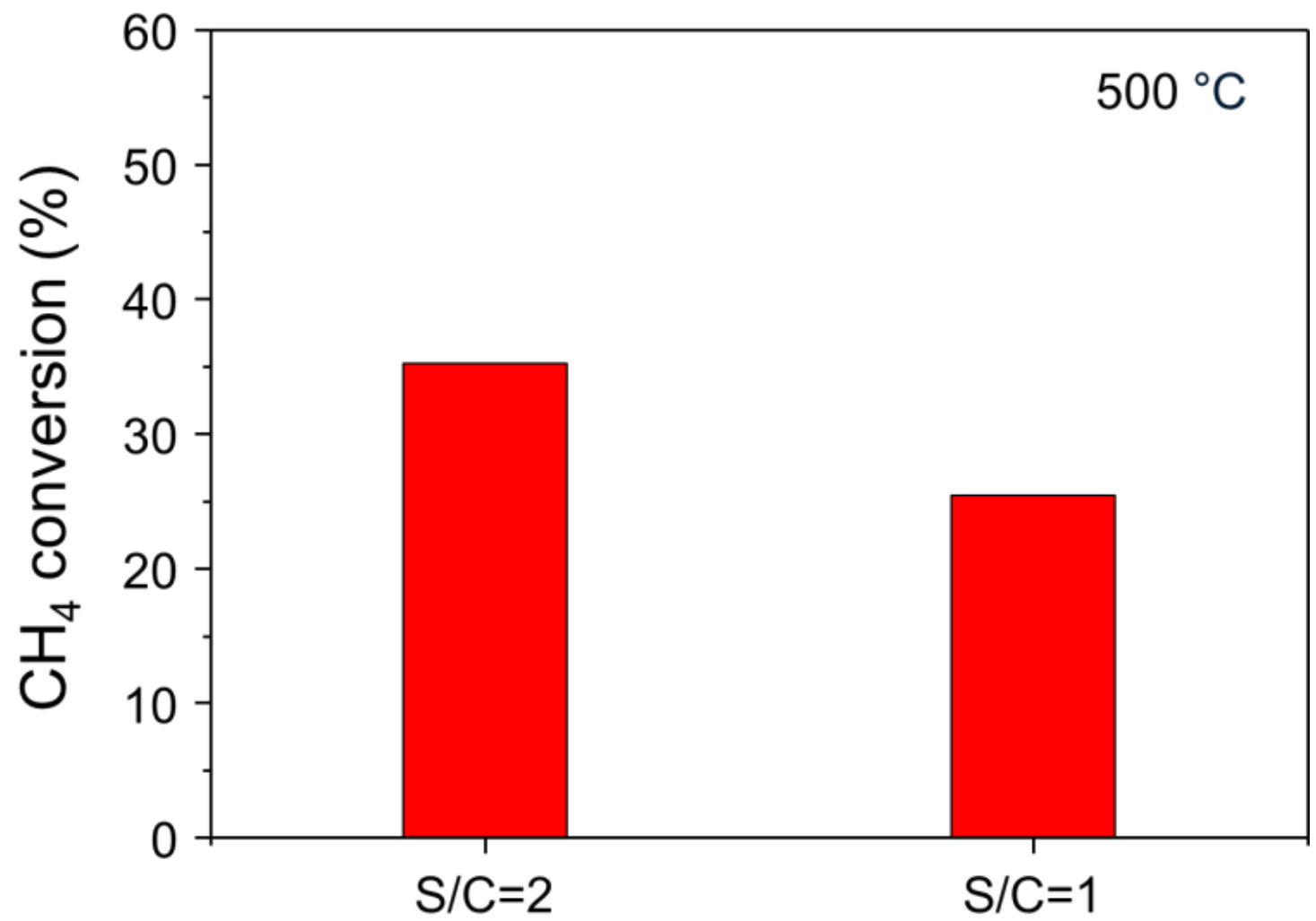

Supplementary Fig. 52. Methane conversion of IL-100% at different S/C ratios. Source data for Supplementary Fig. 52 are provided as a Source Data file.

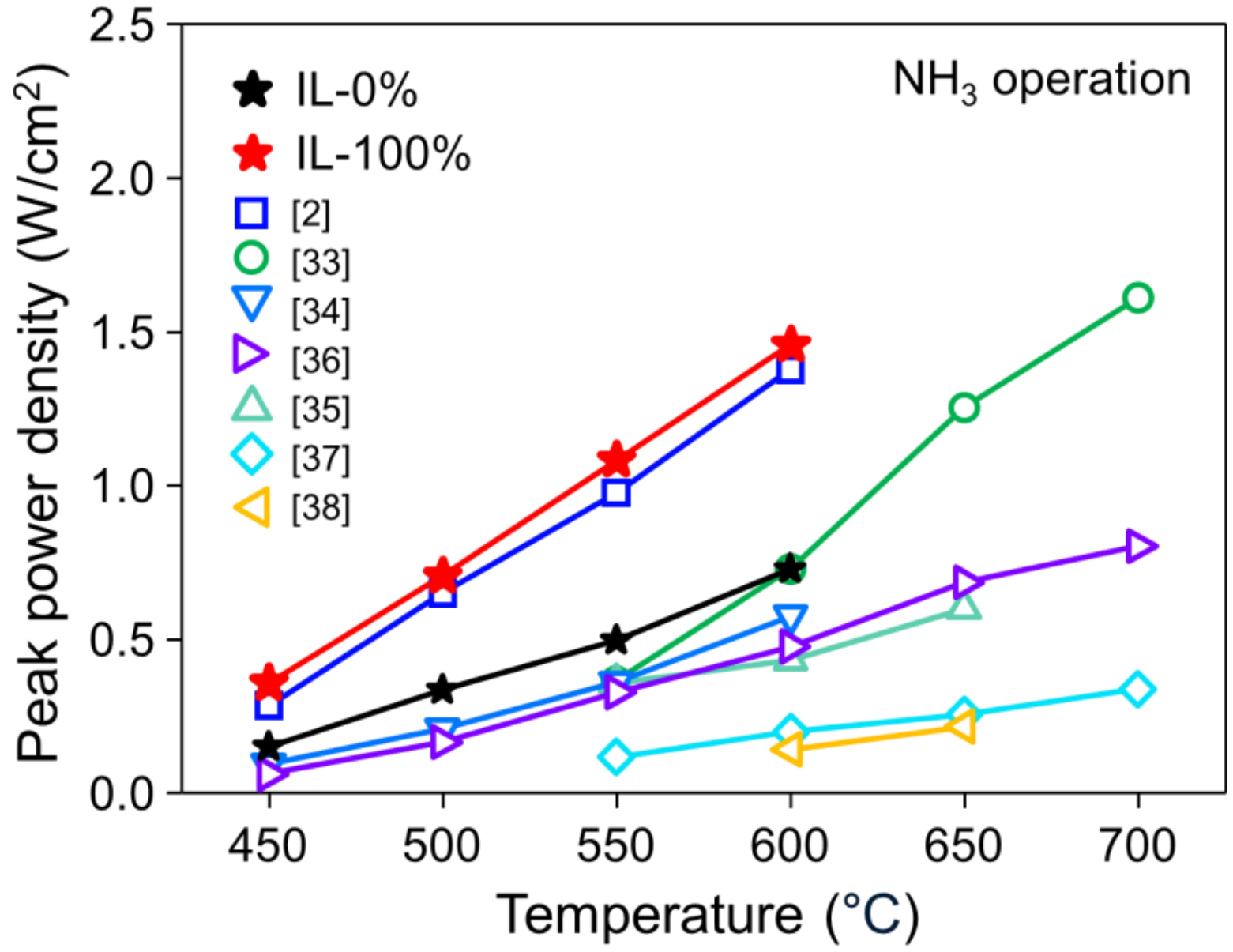

Supplementary Fig. 53. Comparison of peak power densities with previously reported protonic ceramic fuel cells operating with ammonia.[2,33-38]

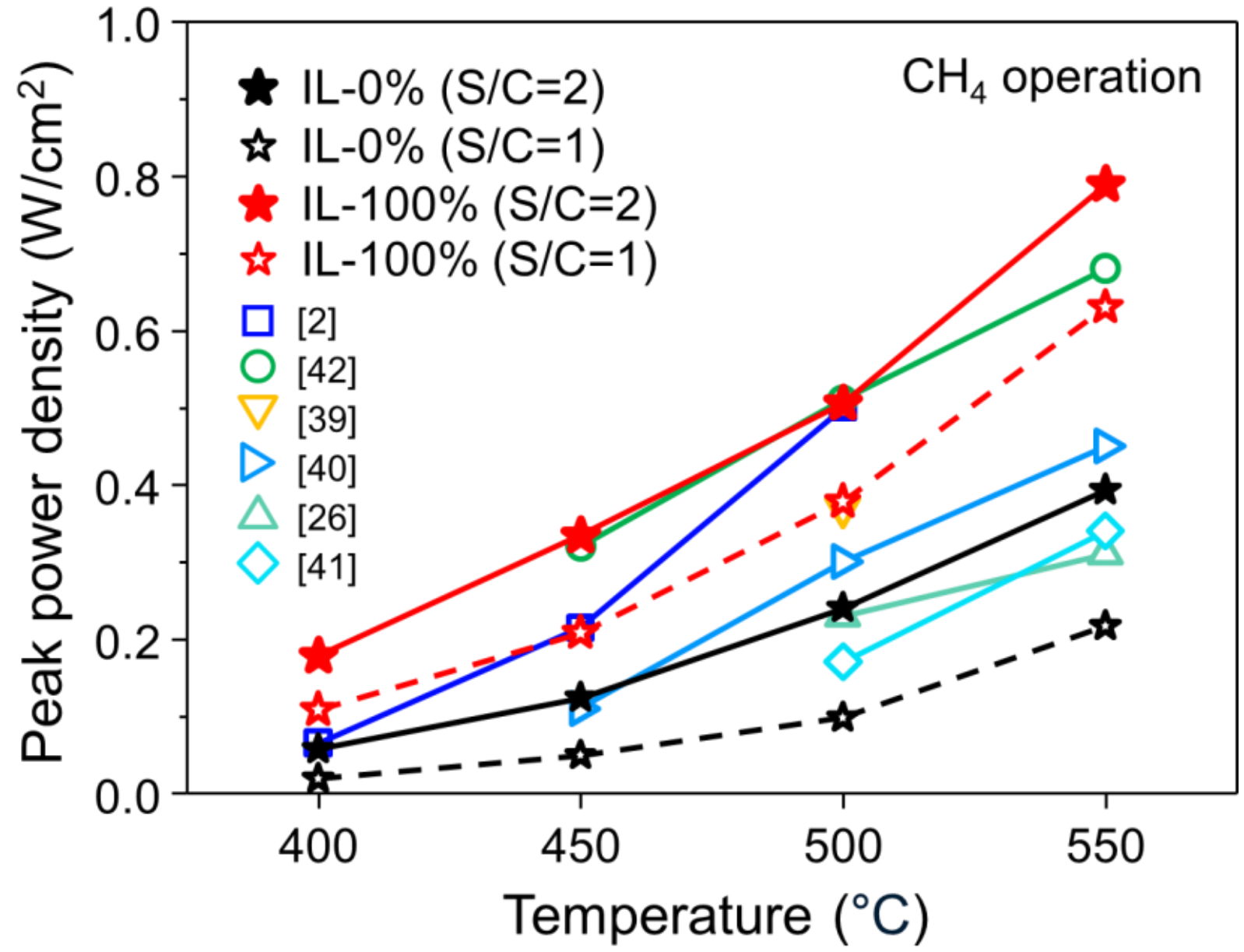

Supplementary Fig. 54. Comparison of peak power densities with previously reported protonic ceramic fuel cells and solid oxide fuel cells operating with methane.[2,26,39-42]

References

1 Choi, M. et al. Exceptionally high performance of protonic ceramic fuel cells with stoichiometric electrolytes. *Energy Environ. Sci.* **14**, 6476-6483 (2021).

2 Liu, F. et al. Lowering the operating temperature of protonic ceramic electrochemical cells to< 450° C. *Nat. Energy* **8**, 1145-1157 (2023).

3 Ding, H. et al. Self-sustainable protonic ceramic electrochemical cells using a triple conducting electrode for hydrogen and power production. *Nat. Commun.* **11**, 1907 (2020).

4 Choi, S. et al. Exceptional power density and stability at intermediate temperatures in protonic ceramic fuel cells. *Nat. Energy* **3**, 202-210 (2018).

5 Saqib, M. et al. Transition from perovskite to misfit-layered structure materials: a highly oxygen deficient and stable oxygen electrode catalyst. *Energy Environ. Sci.* **14**, 2472-2484 (2021).

6 Choi, S., Davenport, T. C. & Haile, S. M. Protonic ceramic electrochemical cells for hydrogen production and electricity generation: exceptional reversibility, stability, and demonstrated faradaic efficiency. *Energy Environ. Sci.* **12**, 206-215 (2019).

7 Zhang, W. et al. A synergistic three-phase, triple-conducting air electrode for reversible proton-conducting solid oxide cells. *ACS Energy Lett.* **8**, 3999-4007 (2023).

8 Bian, W. et al. Revitalizing interface in protonic ceramic cells by acid etch. *Nature* **604**, 479-485 (2022).

9 Park, H. S. et al. High-performance proton ceramic fuel cells using a perovskite oxide cathode surface decorated with CoO_x nanoparticles. *Appl. Surf. Sci.* **612**, 155812 (2023).

10 Dayaghi, A. M. et al. Increasing the thermal expansion of proton conducting Y-doped $BaZrO_3$ by Sr and Ce substitution. *Solid State Ion.* **359**, 115534 (2021).

11 Zhu, Z. et al. High performance and stability enabled by tuning the component thermal expansion coefficients of a proton-conducting solid oxide cell operating at high steam concentration. *ACS Appl. Mater. Interfaces* **15**, 14457-14469 (2023).

12 Zan, J. et al. Characterization and functional application of $PrBa_{0.5}Sr_{0.5}Co_{1.5}Fe_{0.5}O_{5+\delta}$ cathode material for IT-SOFC. *Mater. Res. Bull.* **137**, 111173 (2021).

13 Liu, B. et al. Novel $PrBa_{0.9}Ca_{0.1}Co_{2-x}Zn_xO_{5+\delta}$ double-perovskite as an active cathode material for high-performance proton-conducting solid oxide fuel cells. *Int. J. Hydrogen Energy* **45**, 31009-31016 (2020).

14 Song, Y. et al. Self-assembled triple-conducting nanocomposite as a superior protonic ceramic fuel cell cathode. *Joule* **3**, 2842-2853 (2019).

15 Wan, Y. et al. Thermal cycling durability improved by doping fluorine to $PrBaCo_2O_{5+\delta}$ as oxygen reduction reaction electrocatalyst in intermediate-temperature solid oxide fuel cells. *J. Power Sources* **402**, 363-372 (2018).

16 Tang, W. et al. An Unbalanced Battle in Excellence: Revealing Effect of Ni/Co Occupancy on

Water Splitting and Oxygen Reduction Reactions in Triple-Conducting Oxides for Protonic Ceramic Electrochemical Cells. *Small* **18**, 2201953 (2022).

17 Hung, I.-M. et al. Synthesis and Electrochemical Performance of $PrBa_{0.5}Sr_{0.5}Co_{1.5}Fe_{0.5}O_{5+\delta}$ double perovskite cathode material for Solid Oxide Fuel Cell. *Int. J. Hydrogen Energy* **145**, 371-379 (2025).

18 Kane, N. et al. Durable and high-performance thin-film BHYb-coated BZCYYb bilayer electrolytes for proton-conducting reversible solid oxide cells. *ACS Appl. Mater. Interfaces* **15**, 32395 (2023).

19 Luo, Z. et al. Harnessing high-throughput computational methods to accelerate the discovery of optimal proton conductors for high-performance and durable protonic ceramic electrochemical cells. *Adv. Mater.* **36**, 2311159 (2024).

20 Choi, S. M. et al. Electrochemical analysis of high-performance protonic ceramic fuel cells based on a columnar-structured thin electrolyte. *Appl. Energy* **233**, 29-36 (2019).

21 Choi, M. et al. Interface engineering to operate reversible protonic ceramic electrochemical cells below 500° C. *Adv. Energy Mater.* **15**, 2400124 (2025).

22 He, F. et al. Tailoring the Structural Durability and Proton Conduction of Electrolytes for Highly Fuel-flexible and Reversible Ceramic Cells. *Energy Environ. Sci.* (2025).

23 An, H. et al. A 5×5 cm^2 protonic ceramic fuel cell with a power density of 1.3 W cm^{-2} at 600° C. *Nat. Energy* **3**, 870-875 (2018).

24 Seong, A. et al. Electrokinetic proton transport in triple ($H^+/O^{2-}/e^-$) conducting oxides as a key descriptor for highly efficient protonic ceramic fuel cells. *Adv. Sci.* **8**, 2004099 (2021).

25 Zhou, Y. et al. An active and robust air electrode for reversible protonic ceramic electrochemical cells. *ACS Energy Lett.* **6**, 1511-1520 (2021).

26 Duan, C. et al. Highly durable, coking and sulfur tolerant, fuel-flexible protonic ceramic fuel cells. *Nature* **557**, 217-222 (2018).

27 Kim, J. H. et al. An universal oxygen electrode for reversible solid oxide electrochemical cells at reduced temperatures. *Energy Environ. Sci.* **16**, 3803-3814 (2023).

28 Pei, K. et al. Surface restructuring of a perovskite-type air electrode for reversible protonic ceramic electrochemical cells. *Nat. Commun.* **13**, 2207 (2022).

29 Duan, C. et al. Highly efficient reversible protonic ceramic electrochemical cells for power generation and fuel production. *Nat. Energy* **4**, 230-240 (2019).

30 Luo, Z. et al. Critical role of acceptor dopants in designing highly stable and compatible proton-conducting electrolytes for reversible solid oxide cells. *Energy Environ. Sci.* **15**, 2992-3003 (2022).

31 Park, K. et al. Understanding the highly electrocatalytic active mixed triple conducting $Na_xCa_{3-x}Co_4O_{9-\delta}$ oxygen electrode materials. *Adv. Energy Mater.* **13**, 2202999 (2023).

32 Wu, W. et al. 3D Self-architectured steam electrode enabled efficient and durable hydrogen production in a proton-conducting solid oxide electrolysis cell at temperatures lower than 600

C. *Adv. Sci.* **5**, 1800360 (2018).

33 Zhang, H. et al. An efficient and durable anode for ammonia protonic ceramic fuel cells. *Energy Environ. Sci.* **15**, 287-295 (2022).

34 Aoki, Y. et al. High-efficiency direct ammonia fuel cells based on $BaZr_{0.1}Ce_{0.7}Y_{0.2}O_{3-\delta}$/Pd oxide-metal junctions. *Glob. Chall.* **2**, 1700088 (2018).

35 He, F. et al. A new Pd doped proton conducting perovskite oxide with multiple functionalities for efficient and stable power generation from ammonia at reduced temperatures. *Adv. Energy Mater.* **11**, 2003916 (2021).

36 Lin, Y. et al. Proton-conducting fuel cells operating on hydrogen, ammonia and hydrazine at intermediate temperatures. *Int. J. Hydrogen Energy* **35**, 2637-2642 (2010).

37 Miyazaki, K., Muroyama, H., Matsui, T. & Eguchi, K. Impact of the ammonia decomposition reaction over an anode on direct ammonia-fueled protonic ceramic fuel cells. *Sustain. Energy Fuels* **4**, 5238-5246 (2020).

38 Zhang, L. & Yang, W. Direct ammonia solid oxide fuel cell based on thin proton-conducting electrolyte. *J. Power Sources* **179**, 92-95 (2008).

39 Chen, Y. et al. A robust fuel cell operated on nearly dry methane at 500° C enabled by synergistic thermal catalysis and electrocatalysis. *Nat. Energy* **3**, 1042-1050 (2018).

40 Takagi, Y., Lai, B.-K., Kerman, K. & Ramanathan, S. Low temperature thin film solid oxide fuel cells with nanoporous ruthenium anodes for direct methane operation. *Energy Environ. Sci.* **4**, 3473-3478 (2011).

41 Zha, S., Moore, A., Abernathy, H. & Liu, M. GDC-based low-temperature SOFCs powered by hydrocarbon fuels. *J. Electrochem. Soc.* **151**, A1128-A1133 (2004).

42 Hong, K. et al. Direct methane protonic ceramic fuel cells with self-assembled Ni-Rh bimetallic catalyst. *Nat. Commun.* **14**, 7485 (2023).
