# Supplementary material for: Interface-driven boost in the performance and stability of protonic ceramic electrochemical cells
Source: Nat Commun. 2026 Sep 17;17:10945. doi: 10.1038/s41467-026-77561-6 (PMC13680501; doi:10.1038/s41467-026-77561-6)
Supplement: Supplementary file 2 [file 41467_2026_77561_MOESM2_ESM.pdf]

nature portfolio

Peer Review File

Interface-Driven Boost in the Performance and Stability of Protonic Ceramic Electrochemical Cells

Corresponding Author: Professor Wonyoung Lee

Parts of this Peer Review File have been redacted as indicated to maintain the confidentiality of unpublished data.

Parts of this Peer Review File have been redacted as indicated to remove third-party material.

This file contains all reviewer reports in order by version, followed by all author rebuttals in order by version.

Version 0:

Reviewer comments:

Reviewer #1

(Remarks to the Author)
This manuscript presents a comprehensive and well-executed study that introduces a quantitative interface engineering framework for PCECs, in which interfacial contact coverage is identified and validated as a governing design parameter for both electrochemical performance and durability. By combining controlled model experiments with equivalent-circuit-based impedance analysis, authors systematically correlate contact coverage with interfacial ohmic and polarization resistances. The work is technically strong and addresses an important bottleneck in the development of low-temperature PCECs. The achieved electrochemical performance and durability are impressive, and the experimental investigations are carried out in a systematic manner with appropriate and convincing model validation. However, the following comments need clarification or refinement before the manuscript can be considered for publication.

1. In Figure 1, the optimization process of the precursor-based ESD interlayer is not clearly described. It is unclear which specific conditions were ultimately selected as the optimized ones and why those specific conditions were chosen.

2. The key claim of this manuscript is that contact coverage is the dominant parameter governing both ohmic and polarization resistances at the electrode-electrolyte interface. While the correlation between contact coverage and ohmic resistance is physically intuitive, the extension of the same model to polarization resistance is less straightforward.

3. The log-log relationships in Figure 2f and 2i seem central to authors' argument but its physical meaning is not sufficiently discussed.

4. Authors assume that the specific contact resistance is identical for the electrode-electrolyte and interlayer-electrolyte interfaces. This assumption needs more justification.

5. In Figure S26, the interfacial delamination appears quite severe, but the associated performance degradation seems relatively small. Then, does this imply that a certain degree of interfacial delamination may also have occurred even in samples that do not exhibit noticeable performance degradation?

6. Although authors argue that the approach in the manuscript can be applicable to other electrode materials, no direct validation is provided. Even a limited demonstration using other electrode materials would significantly strengthen the universality of the proposed precursor-based interlayer strategy.

Reviewer #2

(Remarks to the Author)
The manuscript proposes a precursor-based interfacial engineering strategy enabled by ESD to enhance the performance and durability of PCECs. By fabricating an ultrathin, dense PBSCF interlayer, the authors improve interfacial contact coverage and reduce both ohmic and polarization resistances, while enhancing mechanical strength and thermal-cycling robustness. Overall, the study is timely and potentially impactful from both engineering and methodological perspectives.

With substantial revisions and additional supporting evidence, the manuscript may be suitable for publication.
1. Please justify the rationale for employing a co-firing strategy instead of pre-crystallizing the interlayer, specifically clarifying whether interfacial defects arising from precursor decomposition/shrinkage mismatch were present.
2. In Fig. 2c, the most pronounced reduction in resistance occurs in the low-frequency regime, which is typically associated with surface oxygen kinetics or mass transport. Since the interlayer is primarily introduced to improve interfacial physical contact, please clarify the causal link between them.
3. The activation energy should be calculated from the Arrhenius plots and explicitly labeled in Fig. 2f and Fig. 2i. This will allow for a more quantitative comparison of the reaction kinetics.
4. The authors are encouraged to perform a DRT analysis of the post-cycling EIS for IL-0% in Fig. 4b and compare it with the pre-cycling spectrum, to quantitatively identify which frequency-domain process dominates the resistance increase process.
5. It's suggested to discuss whether the adhesion strength exhibits a monotonic relationship with contact coverage and to provide post-test fracture surface morphology.
6. In fig.5(d, e) and fig.5(g, h), it is noted that the IL-100% cells exhibit a slightly lower OCV compared to the IL-0% reference, please explain the underlying reason.
7. The authors are suggested to provide EIS spectra under NH3 and CH4 fuels in the Supplementary Information to further demonstrate the interfacial resilience and mass transport robustness of the ESD-engineered cells in alternative fuel environments.
8. Please provide the cell dimensions and the oxygen-electrode active area in the Methods section, which are important for reproducibility and proper data normalization.

Reviewer #3

(Remarks to the Author)
This manuscript reports a precursor-based electrostatic spray deposition (ESD) method to fabricate PBSCF interlayers in protonic ceramic electrochemical cells, with the central claim that improved contact coverage leads to reduced interfacial resistance. Although the general topic is relevant to the energy conversion community, the manuscript suffers from substantive conceptual and methodological deficiencies that critically undermine the validity of its conclusions. In particular, the work relies heavily on model-dependent EIS fitting without independent experimental validation, employs unverified assumptions regarding contact coverage, and fails to disentangle multiple interfacial effects introduced by the interlayer. As a result, the study does not meet the scientific rigor and evidentiary standards required for publication in Nature Communications.

1. The central premise for this work, that the current characterization techniques for interfacial properties (including contact coverage) are largely qualitative and insufficient, has already been addressed in the authors' recently published work:

Kim, D., Lee, D. G., Kim, H., Jeong, J. S., & Lee, W. (2026). Quantitative interface engineering framework for high-performance and durable Protonic ceramic electrochemical cells. Advanced Energy Materials, e05277. https://doi.org/10.1002/aenm.202505277

2. The manuscript's primary claim, that contact coverage is the dominant factor governing both ohmic and polarization resistances, is derived exclusively from equivalent circuit fitting of EIS data. However, EIS deconvolution is inherently model-dependent and relies on assumptions. The authors did not provide independent, direct experimental evidence (e.g., 3D x-ray tomography, operando transmission electron microscopy, etc.) to validate the fitted parameters or proposed mecanisms. Without corroborating evidence, the mechanistic interpretation remains speculative.
3. The authors assume a linear relationship between interlayer fraction and contact coverage. For the intermediate samples (25%, 50%, 75%), the contact coverage values are not measured but instead assigned through a linear interpolation (Fig. S13, Table S3). The impedance model is then used to validate this assumed linearity, which introduces circular reasoning. The model depends on the assumed coverage values, and the resulting fit is subsequently used to support the assumption itself. This is concerning.
4. Scientifically speaking, the interlayer modifies multiple interfacial properties simultaneously, including chemistry, roughness, defect concentration, wetting behavior, and thermal mechanical compatibility. The work attributes all improvements solely to increased contact coverage, but no experiments are designed to isolate these variables. As a result, the causal link between coverage and performance remains uncertain.
5. The authors claim that the electrode/electrolyte and interlayer/electrolyte interfaces exhibit "nearly identical specific contact resistance," which is surprising given their distinct chemistries, defect structures, and electronic properties. This conclusion is derived solely from equivalent circuit fitting, with no supporting evidence from chemical, electronic, or structural characterization to support.
6. The authors mentnion a same composition PBSCF interlayer without providing mechanistic justification, literature precedent, or comparative controls demonstrating why this choice is necessary, yet it is presented as a systematic strategy. No evidence is offered to show that a chemically identical interlayer is uniquely beneficial or required.
7. The 0–100% interlayer fraction series appears arbitrary, with no conceptual or experimental rationale for why these fractions are meaningful or physically interpretable. Intermediate "coverage" values are not measured but inferred from the model, further weakening the claimed systematicity.
8. Important experimental parameters, such as the fixed interlayer thickness, are not justified, and no optimization or sensitivity analysis is provided. Without a parameter space exploration or sensitivity analysis, the generality and reproducibility of the conclusions remain questionable. Readers cannot assess whether the reported benefits are universal or specific to a narrow set of conditions.

Version 1:

Reviewer comments:

Reviewer #1

(Remarks to the Author)
In this revised version, the authors have adequately addressed my comments. The manuscript can be accepted in its current form.

Reviewer #2

(Remarks to the Author)
The authors have substantially addressed the major concerns raised in the previous review. The revised manuscript is significantly strengthened by additional control experiments, post-cycling DRT analysis, adhesion tests, and EIS measurements under alternative fuel conditions. The central conclusions are now better supported. Publication can be recommended after minor revision.
1. The adhesion-strength data, fracture-surface SEM images, and Raman results for the intermediate samples IL-25%, IL-50%, and IL-75% should be incorporated into the Supplementary Information, rather than only being discussed in the response letter. In addition, the main text should briefly clarify that this relationship represents an experimentally observed correlation under the present testing conditions, rather than a universal mechanical scaling law.
2. Several minor textual and formatting issues remain in the manuscript. For example, “stem-to-carbon ratio” in the caption of Fig. 5 should be corrected to “steam-to-carbon ratio”, and “fundings” in line 444 should be corrected to “findings”. These issues should be corrected before publication.

Reviewer #4

(Remarks to the Author)
The manuscript “Interface-Driven Boost in the Performance and Stability of Protonic Ceramic Electrochemical Cells” reported a precursor-based electrostatic spray deposition method to introduce an interlayer to engineer the interface between the protonic ceramic electrolyte and the triple-conducting oxygen electrode. The ohmic resistance, polarization resistance, and interfacial bonding were improved, resulting in improved fuel cell/electrolysis cell performance and thermal cycle stability. The interfacial coverage was correlated with the ohmic area-specific resistance (ASR) and oxygen polarization resistances. I read through the manuscript and rebuttal letter. Although I acknowledge that it is good work. However, I was not convinced to accept this manuscript for publication because of the following two big concerns.
1) The novelty needs to be further justified. As the manuscript notes, even the authors themselves have reported an interfacial-layer-enhancement effect on ohmic ASR, polarization and thermal stability. The authors claim that the method is scalable, which, however, is supported by the large-area cell performance, ASR, and thermal stability. In addition, there is no clue why this method is better than other methods. The method's operating principles are unclear. Why can the precursor deposition prepare dense thin films? The method is electrostatic deposition. It is not clear whether the method is suitable for other material systems.
2) The authors claim that the quantitively correlated the coverage to specific resistances, which has been mentioned in their previous AEM paper. In addition, the linear relationships between coverage and the interfacial ohmic resistance and the partial polarization resistance were not explained well. It is not clear what the physics are there. Why is the slope 1 rather than some other value? Based on the description, the interface is a dense film; it is unclear whether the interlayer's triple ionic and electronic conductivities and the bulk electrode's conductivities affect the results and fitting conclusions. For example, the authors claim that the number of active sites increased upon interlayer introduction. Will the conclusions be different with an electrode with a different partial conductivity? Therefore, the claimed quantitative correlation needs a deeper understanding. The partial conductivity effect should be included. Will the different tripe conductive electrodes show the same linear relationship and the same fitted slope values? In addition, the low-frequency peak of DRT was related to coverage and attributed to surface reaction sites. More physical explanations of the reaction sites at the interface and the bulk surface should be provided.

Version 2:

Reviewer comments:

Reviewer #4

(Remarks to the Author)
The authors well addressed the concerns what I have raised. Therefore, I suggest the acceptance for publication of the current version.

Open Access This Peer Review File is licensed under a Creative Commons Attribution 4.0 International License, which permits use, sharing, adaptation, distribution and reproduction in any medium or format, as long as you give appropriate credit to the original author(s) and the source, provide a link to the Creative Commons license, and indicate if changes were made.
In cases where reviewers are anonymous, credit should be given to 'Anonymous Referee' and the source.
The images or other third party material in this Peer Review File are included in the article's Creative Commons license, unless indicated otherwise in a credit line to the material. If material is not included in the article's Creative Commons license and your intended use is not permitted by statutory regulation or exceeds the permitted use, you will need to obtain permission directly from the copyright holder.
To view a copy of this license, visit https://creativecommons.org/licenses/by/4.0/

Revision Requested: NCOMMS-25-101349

We thank your consideration of our manuscript for publication in *Nature Communications*. We have revised our manuscript reflecting the reviewers' comments as listed below.

Reviewer #1:

This manuscript presents a comprehensive and well-executed study that introduces a quantitative interface engineering framework for PCECs, in which interfacial contact coverage is identified and validated as a governing design parameter for both electrochemical performance and durability. By combining controlled model experiments with equivalent-circuit-based impedance analysis, authors systematically correlate contact coverage with interfacial ohmic and polarization resistances. The work is technically strong and addresses an important bottleneck in the development of low-temperature PCECs. The achieved electrochemical performance and durability are impressive, and the experimental investigations are carried out in a systematic manner with appropriate and convincing model validation. However, the following comments need clarification or refinement before the manuscript can be considered for publication.

We thank the reviewer for the positive and constructive assessment of our work. We appreciate the reviewer's recognition of the significance of the proposed quantitative interface engineering framework. In response to the comments, we have carefully revised the manuscript, and detailed point-by-point responses are provided below.

1. In Figure 1, the optimization process of the precursor-based ESD interlayer is not clearly described. It is unclear which specific conditions were ultimately selected as the optimized ones and why those specific conditions were chosen.

Thank you for the comment. We agree that the optimization process was not sufficiently discussed in the original manuscript. The desired characteristics for the ideal interlayer include a highly conformal and dense morphology to maximize the interfacial density for increasing both the contact coverage and the electrochemical reaction sites, while remaining sufficiently thin to minimize ion diffusion length. We optimized two key ESD process parameters of the substrate temperature and spray duration. To clarify the optimization process of the precursor-based ESD interlayer, we expanded the parameter space beyond the previously investigated conditions (substrate temperatures of BP50, BP100, BP150, and BP200 and spray durations of 4 h, 6 h, 8 h, and 10 h) to include a higher substrate temperature of BP250 and extended spray durations of 16 h and 24 h. Corresponding electrochemical analyses were also performed for each condition to further support the structural optimization process, as shown in Figs. S5–S8.

To obtain a conformal and dense interlayer, the substrate temperature was optimized to suppress ring-like, non-uniform deposition caused by capillary flow within atomized droplets. In droplet-based deposition processes, substrate temperature strongly affects solvent evaporation behavior and droplet size, thereby regulating capillary flow and the resulting film morphology. Five substrate temperatures (BP50, BP100, BP150, BP200, and BP250), corresponding to 50%, 100%, 150%, 200%, and 250% of the solvent boiling point (190 ℃), were investigated (Fig. S5). At a substrate temperature of BP50 (95 ℃), insufficient solvent evaporation resulted in partially agglomerated structures. Increasing the substrate temperature to BP100 (190 ℃) and BP150 (285 ℃) intensified capillary flow, driving solutes toward

droplet edges and forming non-uniform ring-like structures. At the substrate temperatures of BP200 (380 °C) and BP250 (475 °C), the droplet size decreased below the critical threshold for capillary flow generation, resulting in a highly concentrated and relatively uniform deposition. The corresponding electrochemical analyses (Fig. S6) showed that the peak power density reached its maximum, while both ohmic and polarization resistances were minimized, as the substrate temperature increase up to BP200 (380 °C), and then became saturated at higher temperature. Therefore, BP200 (380 °C) was selected as the optimal substrate temperature.

To maximize the interfacial coverage while maintaining the interlayer sufficiently thin, the spray duration was optimized. SEM images (Fig. S7) revealed that coverage on the BZCYYb electrolyte surface increased with longer spray duration from 4 to 8 h and became saturated with longer spray duration, while the interlayer thickness continuously increased up to 24 h. Electrochemical analyses (Fig. S8) showed a concurrent increase in peak power density and a decrease in both ohmic and polarization resistances with increasing spray duration up to 8 h. Beyond 8 h, further thickening of the interlayer led to a slight increase in the ohmic resistance, which we attribute to an increased ion diffusion length through a thicker interlayer. Accordingly, 8 h was selected as the optimal spray duration.

The final optimization conditions were therefore BP200 with an 8 h spray duration, which yielded a highly conformal, dense, and thin interlayer that maximizes interfacial density while minimizing resistive losses.

The revised manuscript and Supplementary Information are as follows:

[line 19, page 4 in the revised manuscript]

We optimized two key ESD process parameters of the substrate temperature and spray duration, as shown in Supplementary Figs. 5–8. The substrate temperature was optimized to suppress the formation of ring-like structures caused by capillary flow within the droplets (Supplementary Figs. 5 and 6), thereby facilitating the fabrication of the desired dense and uniform interlayer.[25] Subsequently, the spray duration was optimized to achieve the thin and dense interlayer on the electrolyte surface (Supplementary Figs. 7 and 8).

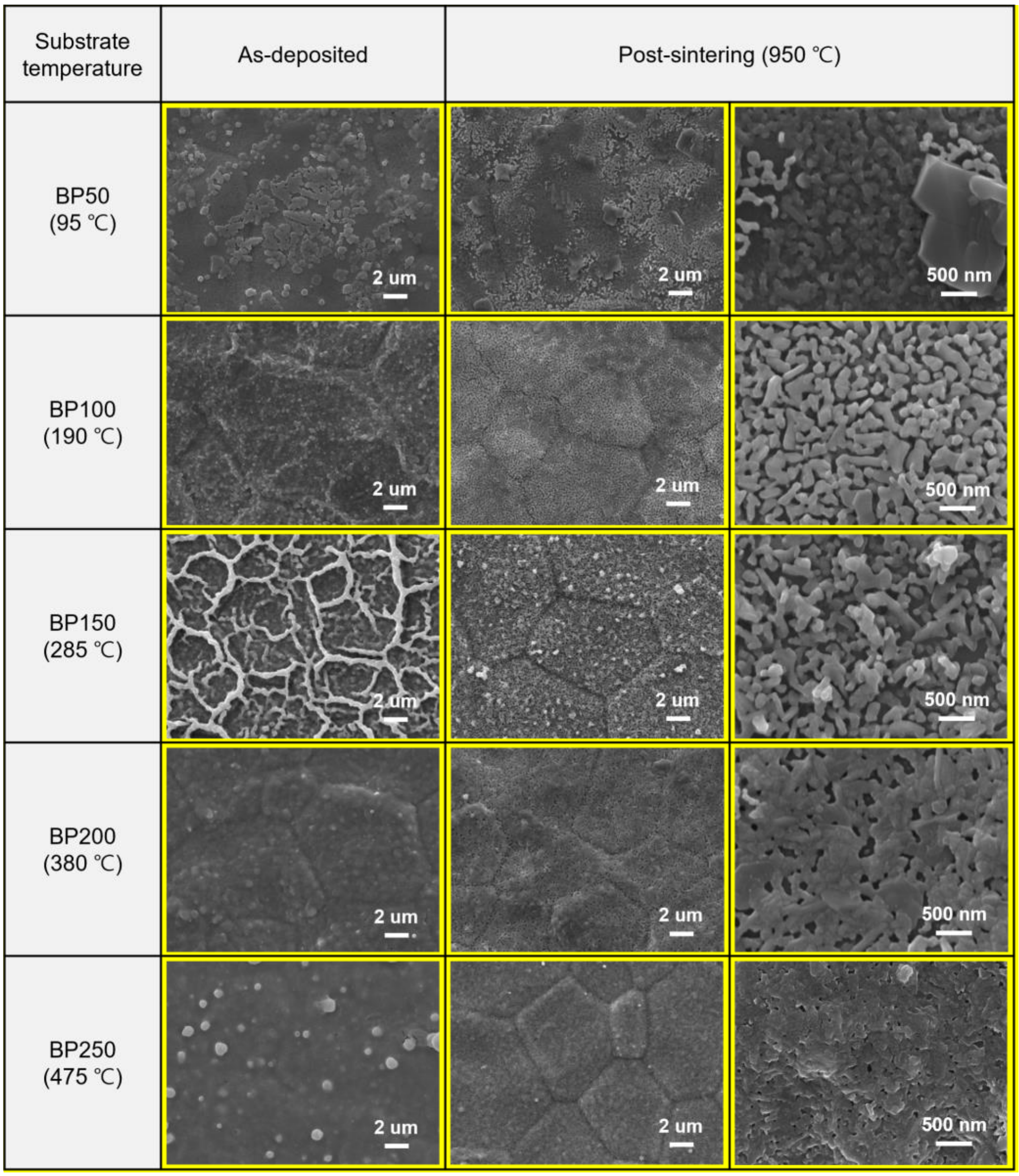

Supplementary Fig. 5. Structural characterization of the precursor-based interlayer was

conducted at five substrate temperatures: BP50, BP100, BP150, BP200, and BP250, corresponding to 50%, 100%, 150%, 200%, and 250% of the solvent boiling point (190 °C), respectively. Surface SEM images were analyzed to evaluate the interlayer morphology in both the as-deposited state and after sintering at 950 °C. In droplet-based deposition processes, substrate temperature strongly affects solvent evaporation behavior and droplet size, thereby regulating capillary flow and the resulting film morphology. Five substrate temperatures (BP50, BP100, BP150, BP200, and BP250), corresponding to 50%, 100%, 150%, 200%, and 250% of the solvent boiling point (190 °C), were investigated. At a substrate temperature of BP50 (95 °C), insufficient solvent evaporation resulted in partially agglomerated structures. Increasing the substrate temperature to BP100 (190 °C) and BP150 (285 °C) intensified capillary flow, driving solutes toward droplet edges and forming non-uniform ring-like structures. At the substrate temperatures of BP200 (380 °C) and BP250 (475 °C), the droplet size decreased below the critical threshold for capillary flow generation, resulting in a highly concentrated and relatively uniform deposition.

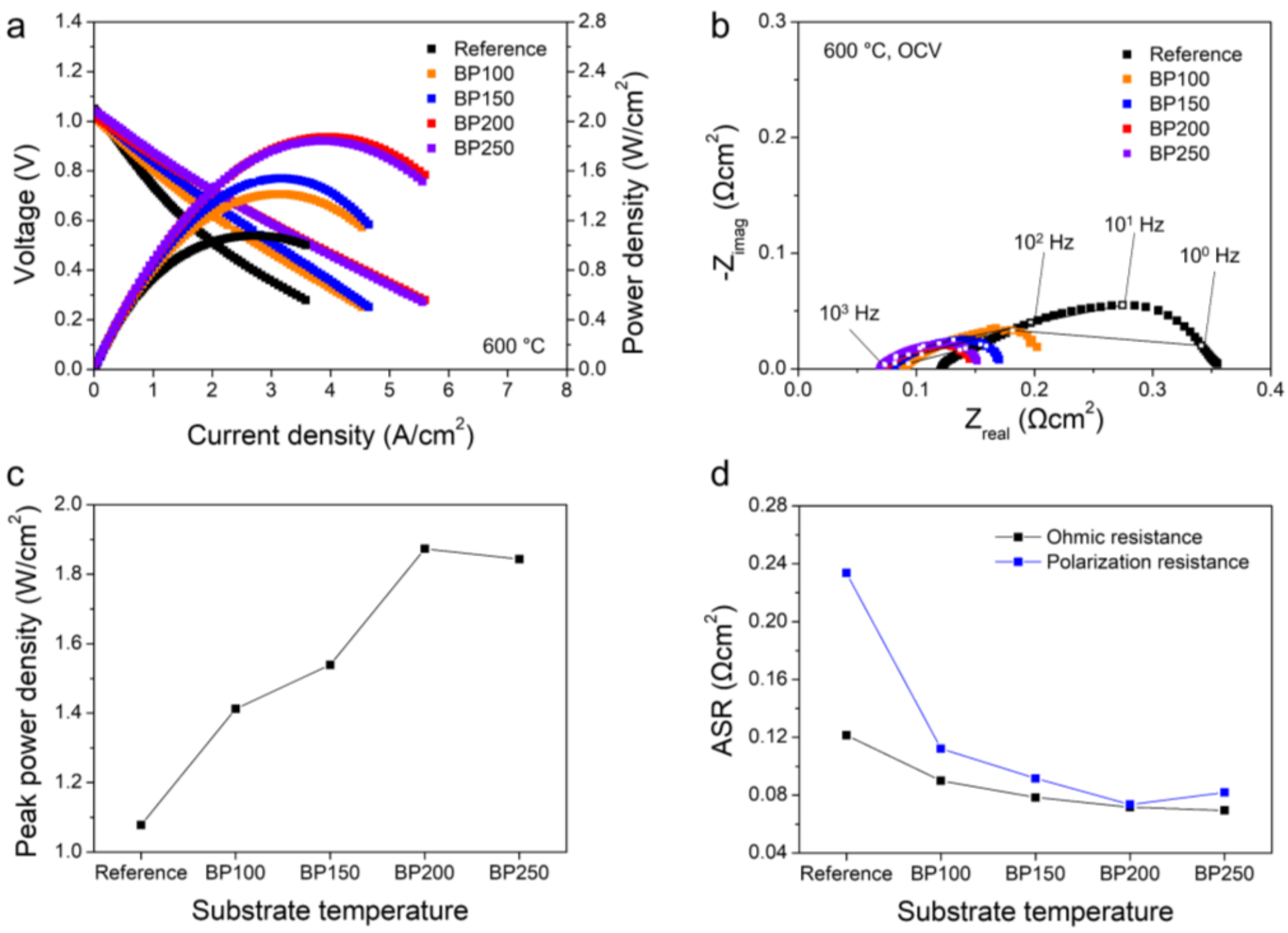

Supplementary Fig. 6. (a) I–V–P curves and (b) Nyquist plots of the reference and interlayer-deposited cells at four substrate temperatures. (c) Peak power density and (d) ohmic and polarization resistances of the corresponding cells measured at 600 °C. The peak power density reached its maximum, while both ohmic and polarization resistances were minimized, as the substrate temperature increase up to BP200 (380 °C), and then became saturated at higher temperature.

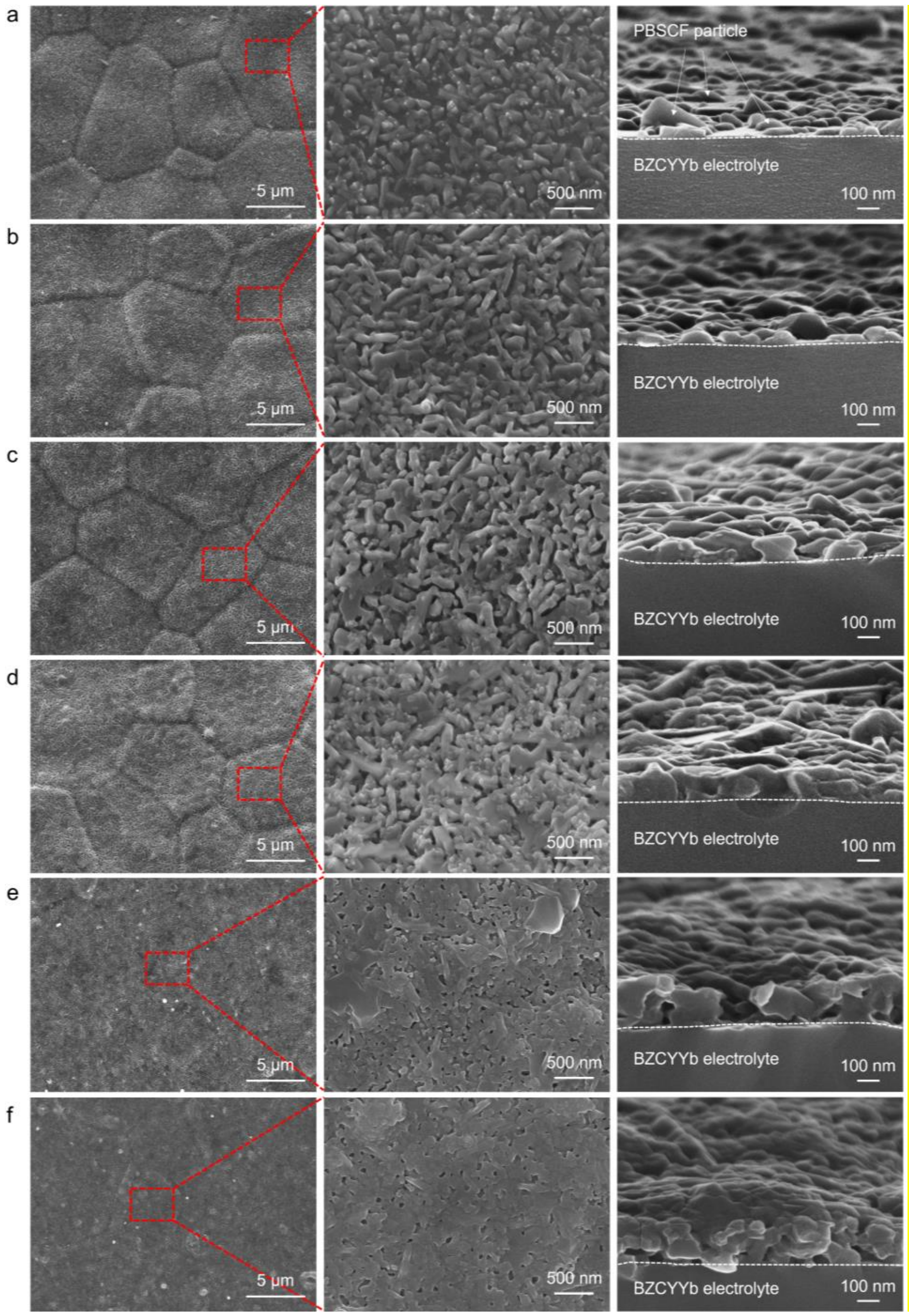

Supplementary Fig. 7. Structural characterization of the precursor-based interlayer deposited with varying spray durations. Surface and tilted SEM images for spray durations of (a) 4 h, (b) 6 h, (c) 8 h, (d) 10 h, (e) 16 h, and (f) 24 h. The coverage on the BZCYYb electrolyte surface increased with longer spray duration from 4 to 8 h and became saturated with longer spray duration, while the interlayer thickness continuously increased up to 24 h. (~150 nm for 8 h and ~400 nm for 24 h).

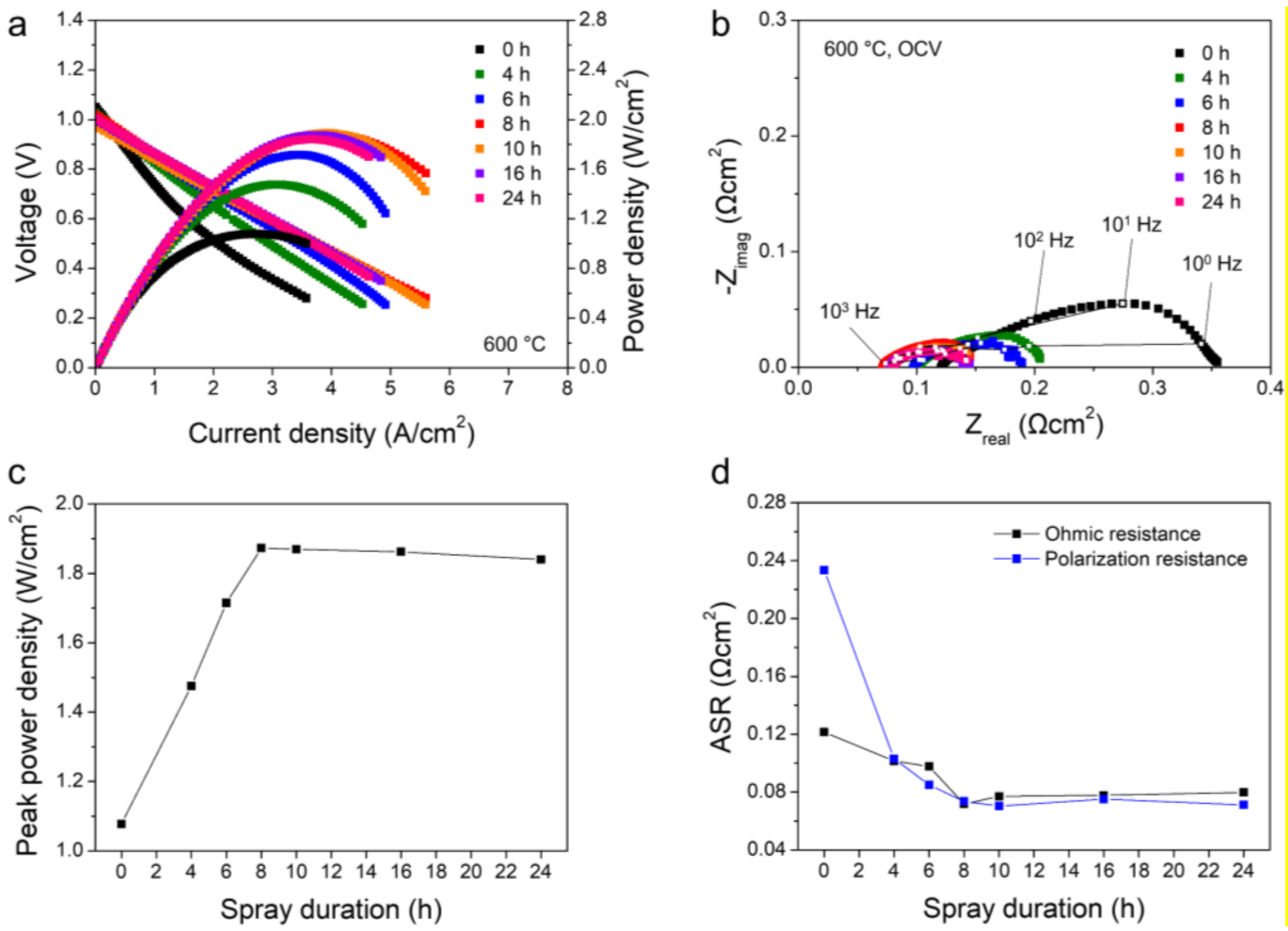

Supplementary Fig. 8. (a) I–V–P curves and (b) Nyquist plots of the reference and interlayer-deposited cells at different spray durations. (c) Peak power density and (d) ohmic and polarization resistances of the corresponding cells measured at 600 °C. Electrochemical analyses showed a concurrent increase in peak power density and a decrease in both ohmic and polarization resistances with increasing spray duration up to 8 h. Beyond 8 h, further thickening of the interlayer led to a slight increase in the ohmic resistance, which we attribute to an increased ion diffusion length through a thicker interlayer. Accordingly, 8 h was selected as the optimal spray duration.

2. The key claim of this manuscript is that contact coverage is the dominant parameter governing both ohmic and polarization resistances at the electrode-electrolyte interface. While the correlation between contact coverage and ohmic resistance is physically intuitive, the

extension of the same model to polarization resistance is less straightforward.

Thank you for raising this important point. In low-temperature PCECs employing triple-conducting oxide (TCO) air electrodes, the dominant contribution to polarization resistance arises primarily from oxygen surface exchange reactions at the air electrode. In our system, the precursor-based interlayer forms a thin, dense, and nearly two-dimensional structure, with a specific surface area of 1.066 ± 0.019 $\mu m^2/\mu m^2$ (Fig. S9), indicating that the interlayer surface area scales approximately with the underlying contact coverage. Therefore, contact coverage can be reasonably used as an effective descriptor of the accessible reaction sites, which explains why polarization resistance can also be expressed as a function of contact coverage. This interpretation is supported by the Bode plots (Fig. R1), which exhibit a dominant impedance response in the 10^{-1}–10^{1} Hz range, characteristic of oxygen surface exchange reactions in low-temperature PCECs with TCO air electrodes. [R1-R2] Similar frequency-domain behavior has also been reported for other state-of-the-art PCECs (Fig. R2). DRT analysis of the model samples (Fig. S22) shows that the most pronounced decrease with increasing interlayer fraction occurs in the low-frequency response (10^{-1}–10^{1} Hz), confirming that the reduction in polarization resistance is dominated by oxygen surface exchange reactions. Lastly, the Arrhenius plots (Fig. S23) reveal a nearly constant activation energy of 1.04 ± 0.06 eV across all model samples, indicating that the reduction in polarization resistance stems primarily from the structural modification (increase in accessible reaction sites), rather than from changes in the intrinsic reaction kinetics.

The related description was provided in the originally submitted manuscript and

Supplementary Information as follows:

[line 13, page 8 in the revised manuscript]

This relationship is consistent with the unique characteristics of PCECs, where the low-temperature operation and use of triple conducting oxide (TCO) electrodes make surface oxygen exchange reactions the rate-determining step (RDS) in polarization resistance.[31-33] This was clearly supported by the Bode plots (Fig. 2(c) and Supplementary Fig. 19), exhibiting a dominant impedance response at a specific frequency at approximately 10^{-1}–10^{1} Hz, typical of surface exchange reactions.[11,30] Therefore, the interlayer contributes to performance enhancement not only by increasing the contact coverage with the underlying electrolyte, but also creating additional surface reaction sites above the interlayer (Supplementary Fig. 20). The 2D morphology of the interlayer, confirmed by a specific surface area of 1.066 ± 0.019 $\mu m^2/\mu m^2$ by AFM analysis (Supplementary Fig. 9), enables these surface reaction sites to scale proportionally with the contact coverage. This structural feature underpins the strong correlation between the contact coverage and polarization resistance, enabling a quantitative assessment of interfacial contribution to electrochemical performance.

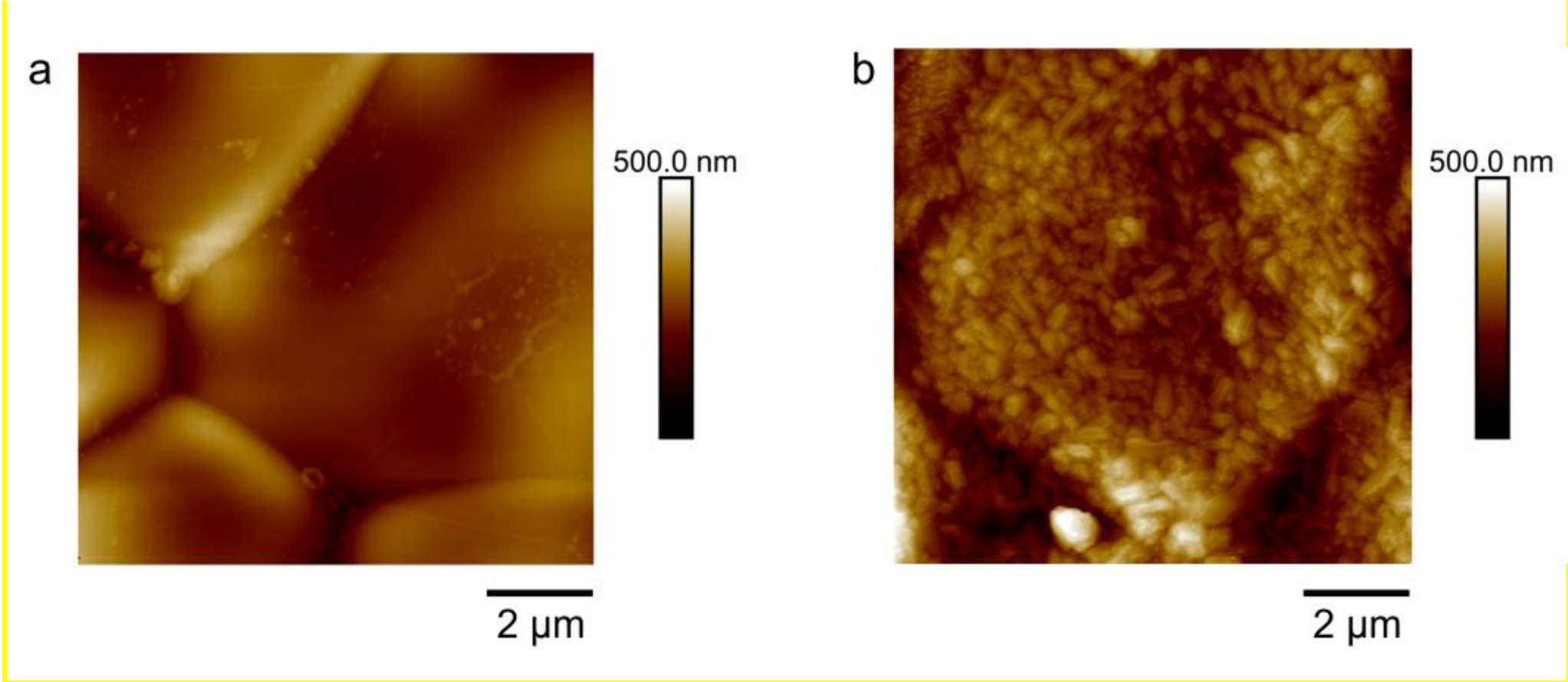

Supplementary Fig. 9. AFM images of (a) the pristine electrolyte surface and (b) the precursor-based interlayer surface. The pristine electrolyte surface exhibited an RMS roughness of 46.7 ± 16.8 nm and a specific surface area of 1.010 ± 0.010 µm^2/µm^2, while the precursor-based interface showed an RMS roughness of 48.6 ± 9.5 nm and a specific surface area of 1.066 ± 0.019 µm^2/µm^2.

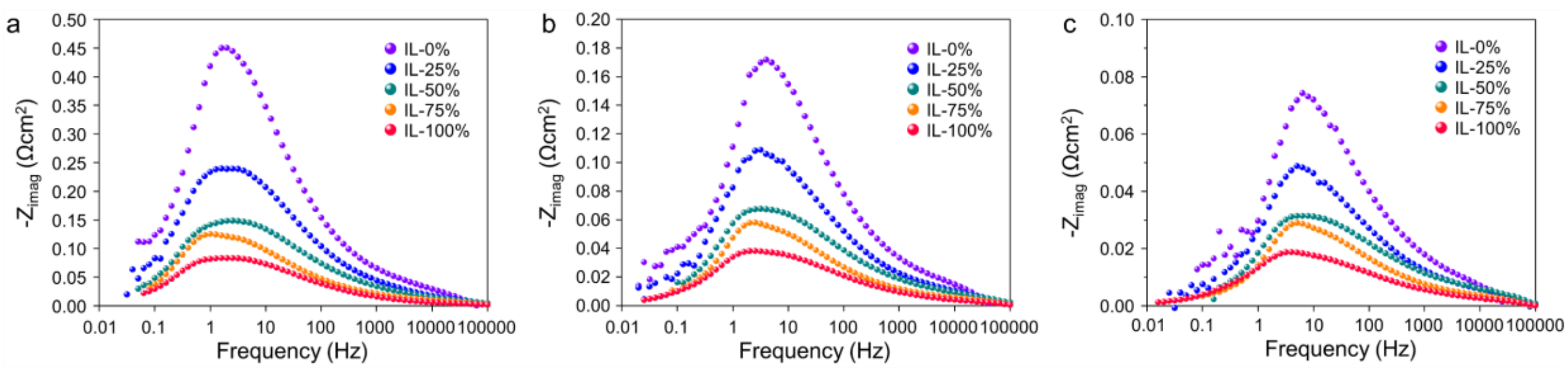

Fig. R1. Bode plots of the model samples under OCV conditions at (a) 500 °C, (b) 550 °C, and (c) 600 °C.

[FIGURE REDACTED]

Fig. R2. (a) DRT analysis and (b) Nyquist plots of previously reported PCECs under OCV conditions. The positions of -log(τ)=0 and -log(τ)=2 s in (a) correspond to 0.159 and 15.9 Hz, respectively. [R3 and R4]

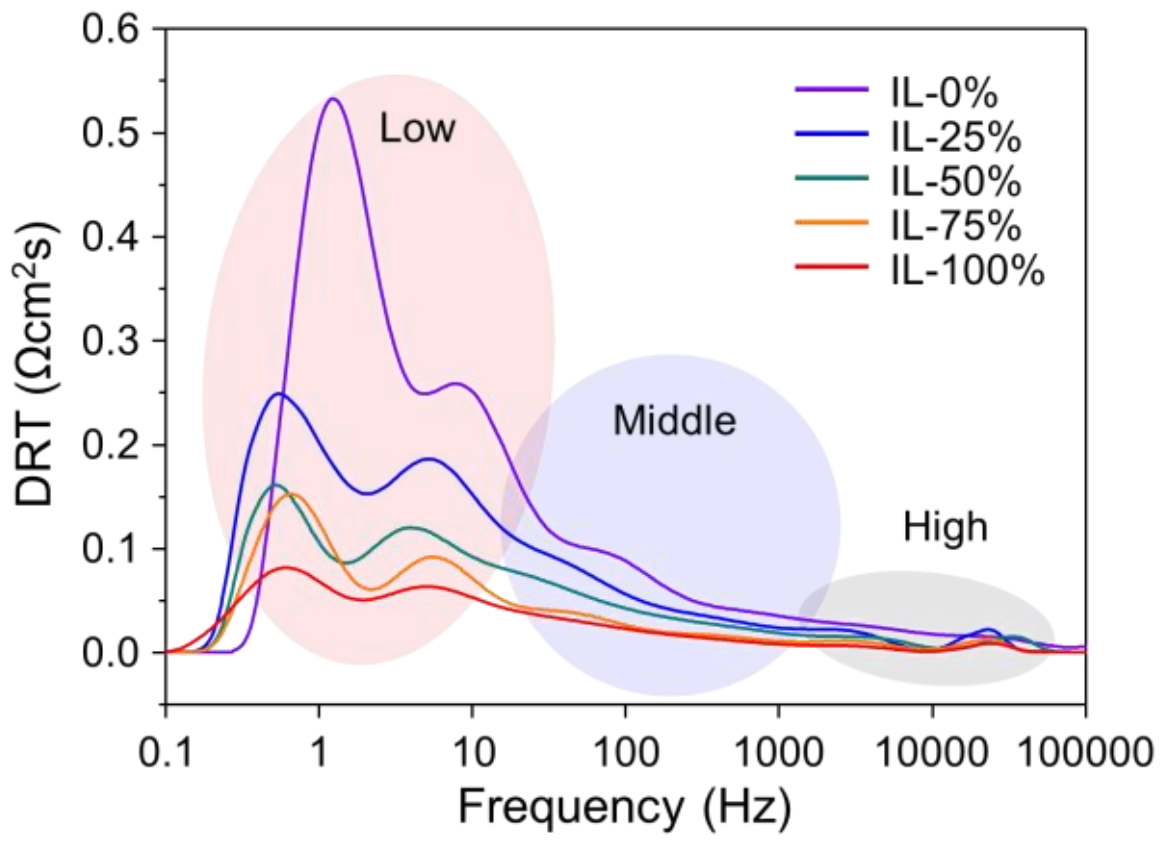

Supplementary Fig. 22. DRT analysis of the model samples at 500 °C under OCV conditions.

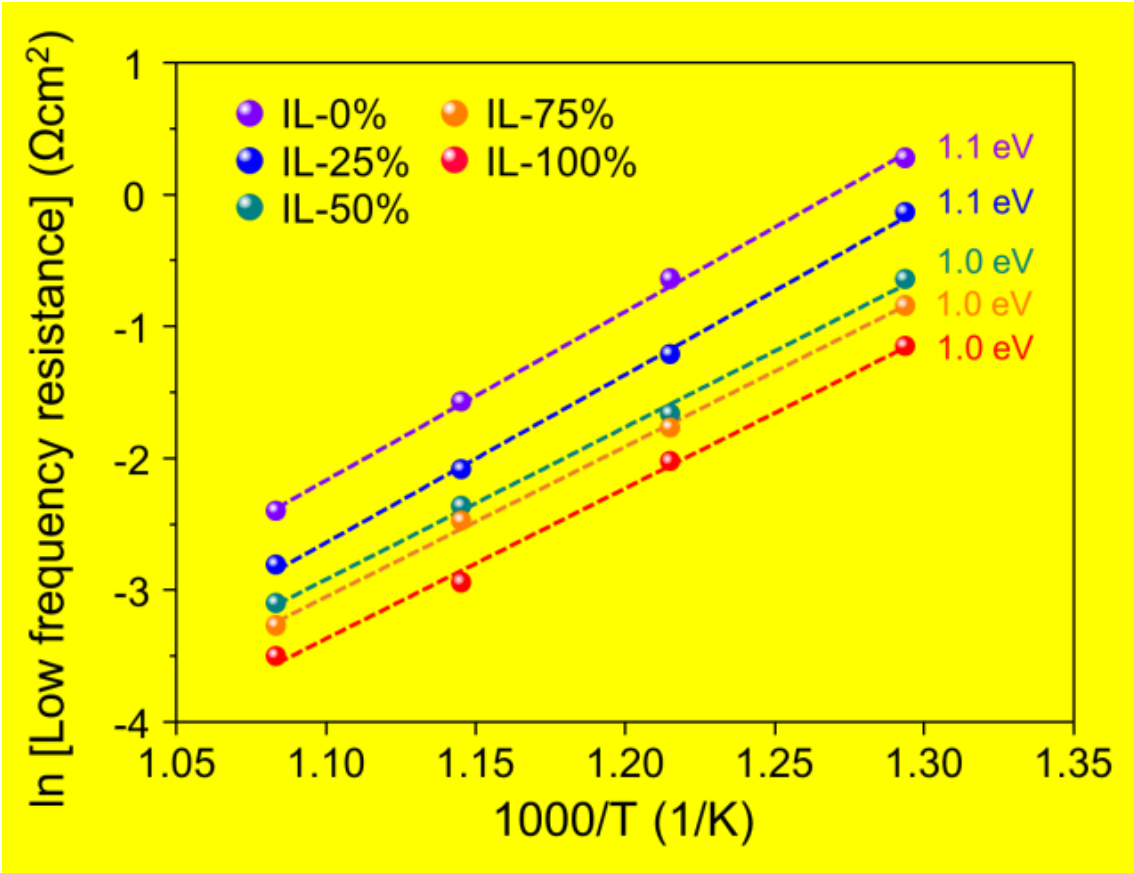

Supplementary Fig. 23. Arrhenius plots of the low-frequency resistances for IL-0%, IL-25%, IL-50%, IL-75%, and IL-100% in the temperature range of 650–500 °C.

[R1] Choi, M. et al. Interface engineering to operate reversible protonic ceramic electrochemical cells below 500 °C. *Adv. Energy Mater.* **15**, 2400124 (2025).

[R2] Choi, S. M. et al. Electrochemical analysis of high-performance protonic ceramic fuel cells based on a columnar-structured thin electrolyte. *Appl. Energy* **233**, 29–36 (2019).

[R3] Yun, J. et al. Synergistic optimization of power output and thermomechanical stability enabled by rational cathode design in protonic ceramic fuel cells. *Adv. Funct. Mater.* e25258 (2025).

[R4] Zhao, S. et al. Reverse atom capture on perovskite surface enabling robust and efficient cathode for protonic ceramic fuel cells. *Adv. Mater.* **36**, 2405052 (2024).

3. The log-log relationships in Figure 2f and 2i seem central to authors' argument but its physical meaning is not sufficiently discussed.

Thank you for the comment. The log-log plots in Figs. 2f and 2i were used to examine whether the interface-related resistances follow a power-law dependence on contact coverage. The observed linear trends with a slope of -1 indicates that both interface-related resistances scale as $R_{interface} \propto \theta^{-1}$ (θ: contact coverage), demonstrating the inverse relationship between the interface resistance and contact coverage. The linear relationships (slopes of -1) remain parallel in the temperature range of 500-650 °C, and the activation energies obtained from the Arrhenius plots are nearly unchanged, indicating that the reduction in the interface-related resistances is governed primarily by structural improvement, rather than by changes in intrinsic reaction kinetics.

The related description was described in the originally submitted manuscript as follows:

[line 34, page 7 in the revised manuscript]

Arrhenius plots (Supplementary Fig. 18) showed consistently lower interface resistances at all temperatures, while the activation energies remained nearly unchanged (0.190 ± 0.009 eV), indicating that the reduction in the interface resistance stems from the structural modifications, particularly the increased contact coverage, rather than changes in reaction kinetics.[12,29,30] Fig. 2(f) shows a clear correlation between the contact coverage and the interface resistance at all temperatures. The interface resistances exhibited a linear trend in double logarithmic scale with a slope of -1, demonstrating an inverse proportionality to the contact coverage. The linear parallel slopes at different temperatures suggest a consistent underlying mechanism driving the reduction in the interface resistance.

[line 4, page 9 in the revised manuscript]

Arrhenius plots (Supplementary Fig. 23) and their correlation with the contact coverage (Fig. 2(i)) revealed a consistent activation energy (1.04 ± 0.06 eV) and parallel trends in all contact coverages, confirming that increased contact coverage is the dominant factor reducing polarization resistance.

4. Authors assume that the specific contact resistance is identical for the electrode-electrolyte and interlayer-electrolyte interfaces. This assumption needs more justification.

Thank you for the important comment. To clarify, the similarity in specific contact resistance between the electrode/electrolyte and interlayer/electrolyte interfaces was not assumed, but rather derived from a combination of independently measured structural quantities and experimentally obtained resistance values.

The calculated specific contact resistances for two interfaces are comparable for all temperature range (Table S6). While the powder-based PBSCF and precursor-based PBSCF may exhibit differences in chemistries, defect structures, and electronic properties, these differences are relatively limited in the present system. As a result, two interfaces exhibit similar intrinsic interfacial resistivity, which is reflected in the comparable specific contact resistance values. Therefore, the observed similarity in specific contact resistance is physically reasonable. Instead, it supports the conclusion that the reduction in the interfacial resistance is governed

primarily by the increase in contact coverage, rather than by changes in intrinsic interfacial properties.

This interpretation is further supported by the fact that other interfacial variables, aside from contact coverage, were carefully controlled and exhibited minimal variation in the present system. Accordingly, the present study was designed to systematically evaluate the role of contact coverage in electrochemical performance while minimizing or constraining other contributing factors, including chemistry, roughness, microstructure, defect concentration, and physical contact characteristics.

To isolate the effect of material chemistry, the interlayer was fabricated using PBSCF with same composition as the electrode. TEM point EDS analysis (Fig. S10, Table S1) and XPS analysis (Table R1) confirm identical atomic ratios between the PBSCF electrode and PBSCF interlayer, thereby excluding differences in chemical composition as a dominant factor. In addition, AFM analysis (Fig. S9) shows that the interlayer surface exhibits a roughness of 48.6 ± 9.5 nm and a specific surface area of 1.066 ± 0.019 $\mu m^2/\mu m^2$, comparable to those of the pristine electrolyte surface, 46.7 ± 16.8 nm and 1.010 ± 0.010 $\mu m^2/\mu m^2$, respectively, indicating a nearly 2D morphology. This suggests that variations in surface roughness and associated surface area are minimal. Although the powder-based PBSCF electrode and the precursor-based PBSCF interlayer may exhibit differences in crystallite size and the associated defect chemistry due to their distinct fabrication methods, these differences are limited under the present conditions. XRD analysis (Fig. S11) shows that the crystallite sizes of the electrode (25.1 ± 2.7 nm) and the interlayer (21.1 ± 1.9 nm) are comparable. Furthermore, XPS O 1s analysis (Fig. S25 and Table S7) indicates similar oxygen vacancy concentrations for the electrode (relative area of defect-related peaks, 0.16) and the interlayer (relative area of defect-related peaks, 0.18). These results indicate that differences in microstructure and defect

concentration exist but are limited, and therefore are unlikely to be the dominant factors governing the observed performance changes.

This interpretation is further supported by nearly unchanged activation energies for both ohmic and polarization resistances. The Arrhenius plots of the ohmic and polarization resistances for the model samples (Fig. R3) exhibit parallel trends with similar activation energies (0.190 ± 0.009 and 1.04 ± 0.06 eV, respectively), indicating that the underlying reaction mechanisms remain unchanged. Since variations in chemistry or defect structure typically manifest as changes in activation energy, the absence of such changes suggests that the observed improvements do not originate from intrinsic kinetic improvement. Furthermore, the contact coverage-based model shows strong agreement with experimental data, with high R^2 values (0.997 ± 0.001 for ohmic resistance and 0.974 ± 0.015 for polarization resistance) (Fig. R4). The clear linear scaling behavior observed in the power-law relationship (Fig. R5) further supports that the variation in both ohmic and polarization resistances is correlated with contact coverage.

Therefore, while multiple interfacial factors may exist, the combination of identical materials composition, comparable surface morphology, similar microstructure, similar defect concentration, and unchanged activation energies provides consistent evidence that the observed performance improvement is governed primarily by structural modification, that is the increase in interfacial contact coverage, rather than by changes in intrinsic interfacial properties.

We have revised the manuscript and Supplementary Information as follows:

[line 26, page 7 in the revised manuscript]

Fitting results revealed that the specific contact resistance, defined as the interface resistance normalized by the contact coverage, was nearly identical for both interface types: the electrode/electrolyte interface and the interlayer/electrolyte interface, consistent with the values independently derived from measured structural quantities and experimentally obtained resistance values.[20] Detailed calculations are provided in Supplementary Table 6. This similarity further substantiates that contact coverage is the primary factor governing interface resistance.

Supplementary Table 6. Calculated specific contact resistance.

	Temperature (°C)	IL-0%	IL-100%
Total ohmic resistance (Ωcm^2)	650	0.1030	0.0563
	600	0.1176	0.0626
	550	0.1354	0.0754
	500	0.1739	0.0965
Non-interface resistance (Ωcm^2)	650	0.0367	
	600	0.0407	
	550	0.0483	
	500	0.0652	
Interface resistance (Ωcm^2)	650	0.0663	0.0196
	600	0.0769	0.0219
	550	0.0871	0.0271
	500	0.1087	0.0313
Contact coverage (%)	650	26.7 ± 4.1	92.6 ± 0.6
	600		
	550		
	500		
Specific contact resistance (Ωcm^2)	650	0.0177 ± 0.0027	0.0181 ± 0.0001
	600	0.0205 ± 0.0032	0.0203 ± 0.0001
	550	0.0233 ± 0.0036	0.0251 ± 0.0002
	500	0.0290 ± 0.0045	0.0290 ± 0.0002

[Interface resistance] = [Total ohmic resistance] - [Non-interface resistance]

[Specific contact resistance] = [Interface resistance] × [Contact coverage/100]

[line 10, page 9 in the revised manuscript]

This conclusion was further supported by the strong linear correlation with an R^2 of ~0.978 observed in the normalized Arrhenius plots of all interface resistance values (Supplementary Fig. 24).[12] Importantly, this interpretation does not imply that the contact coverage is the only interfacial variable governing electrochemical performance. Therefore, compositionally identical PBSCF was used for the electrode and interlayer to provide a controlled model system that minimizes variations in other material-related factors, including chemistry, microstructure, and defect concentration. This design is supported by TEM point EDS analysis confirming similar chemical composition (Supplementary Fig. 10 and Supplementary Table 1), AFM showing comparable surface roughness (Supplementary Fig. 9), XRD indicating similar crystallite sizes (Supplementary Fig. 11), XPS O 1s analysis showing comparable oxygen vacancy-related features (Supplementary Fig. 25 and Supplementary Table 7), and the nearly unchanged activation energies (Supplementary Figs. 18 and 23).

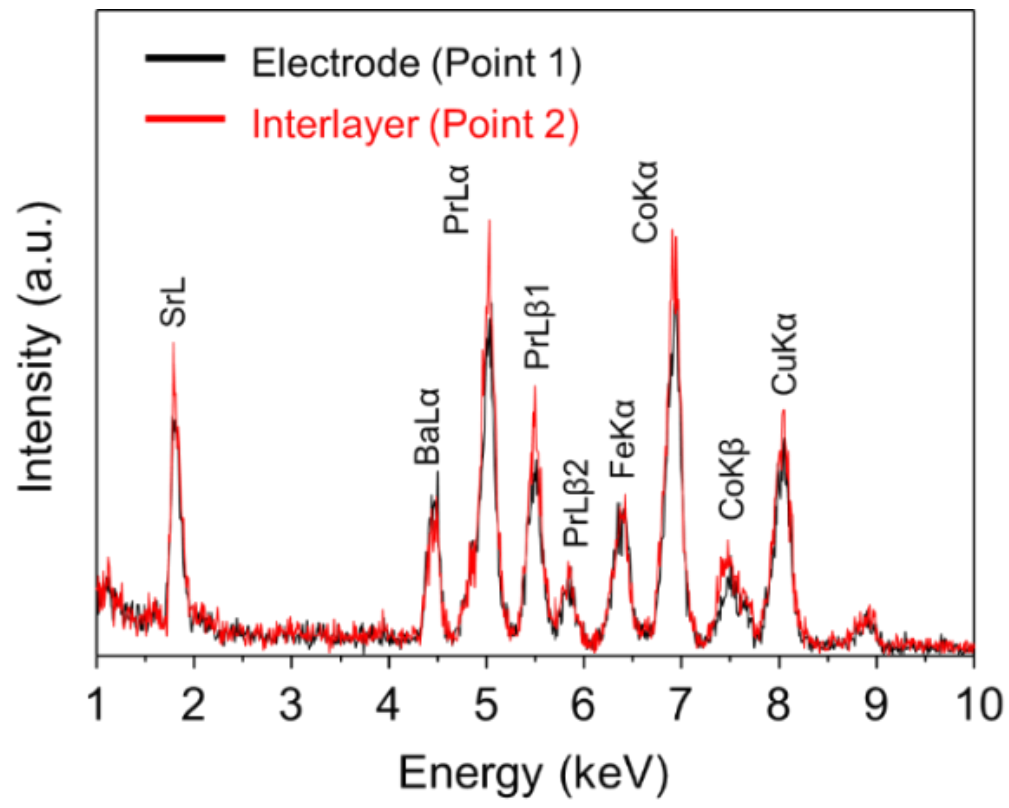

Supplementary Fig. 10. EDS spectra acquired at the powder electrode (Point 1) and the precursor-based interlayer (Point 2).

Supplementary Table 1. Atomic ratios acquired at the powder electrode (Point 1) and the precursor-based interlayer (Point 2) from EDS spectra in Supplementary Fig. 10.

	Atomic ratio over (Pr+Ba+Sr+Co+Fe)				
	Pr	Ba	Sr	Co	Fe
Electrode (Point 1)	0.220	0.082	0.180	0.434	0.083
Interlayer (Point 2)	0.225	0.084	0.171	0.445	0.075

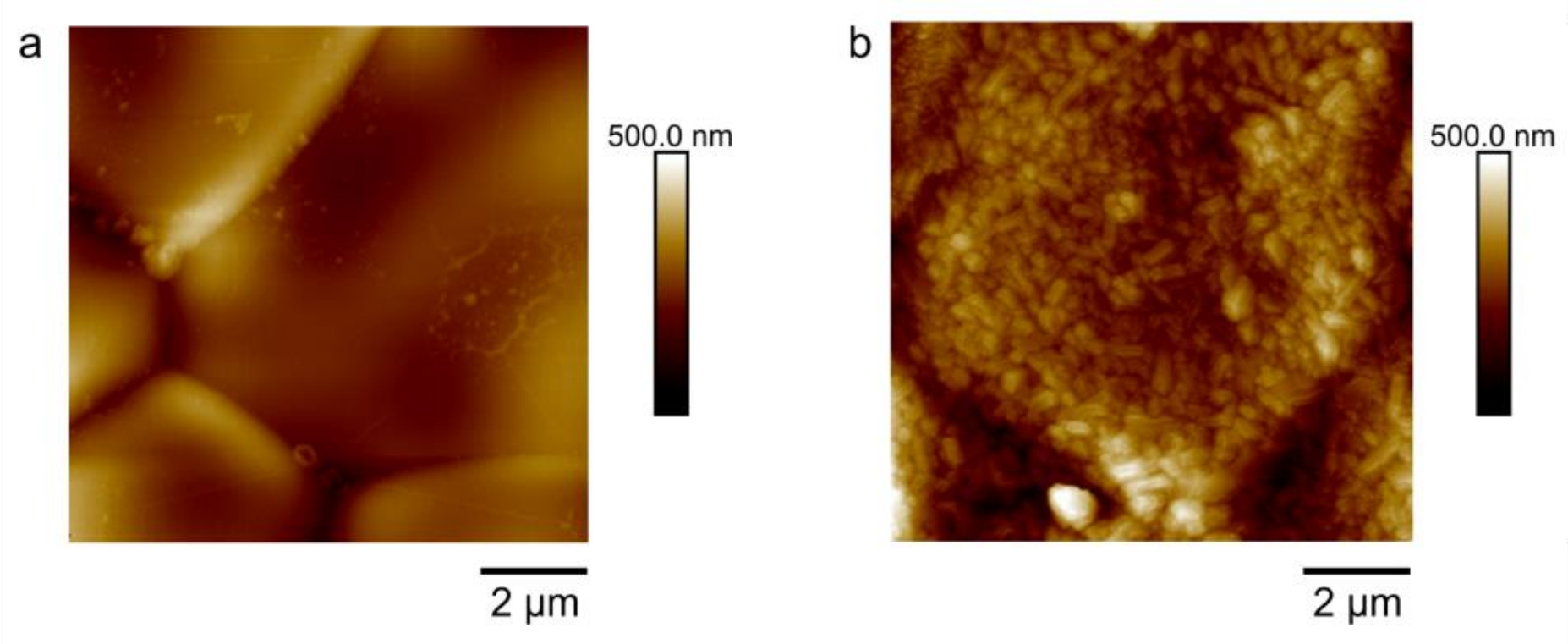

Supplementary Fig. 9. AFM images of (a) the surface of the pristine electrolyte and (b) the surface of the precursor-based interlayer. The electrolyte surface exhibited an RMS roughness

of 46.7 ± 16.8 nm and a surface area of 1.010 ± 0.010 μm^2/μm^2, while the interface surface showed an RMS roughness of 48.6 ± 9.5 nm and a surface area of 1.066 ± 0.019 μm^2/μm^2.

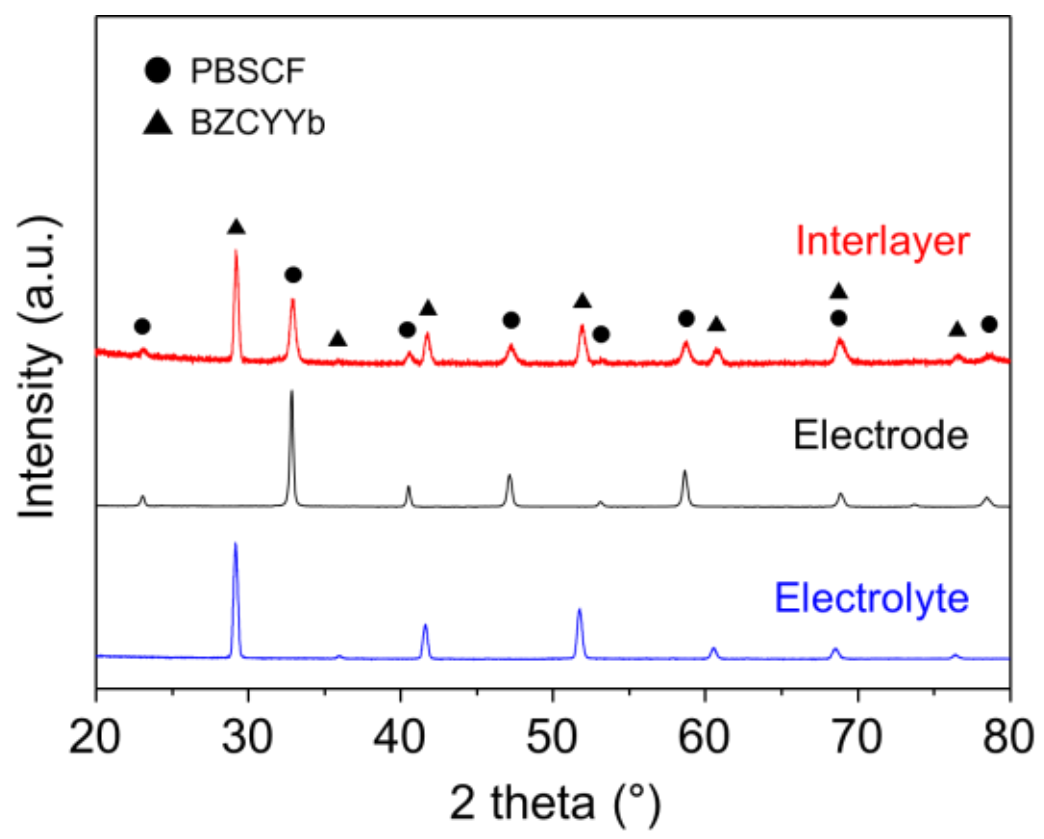

Supplementary Fig. 11. XRD patterns of the precursor-based interlayer, powder electrode, and electrolyte. The crystallite sizes of the electrode and interlayer are 25.1 ± 2.7 nm and 21.1 ± 1.9 nm, respectively.

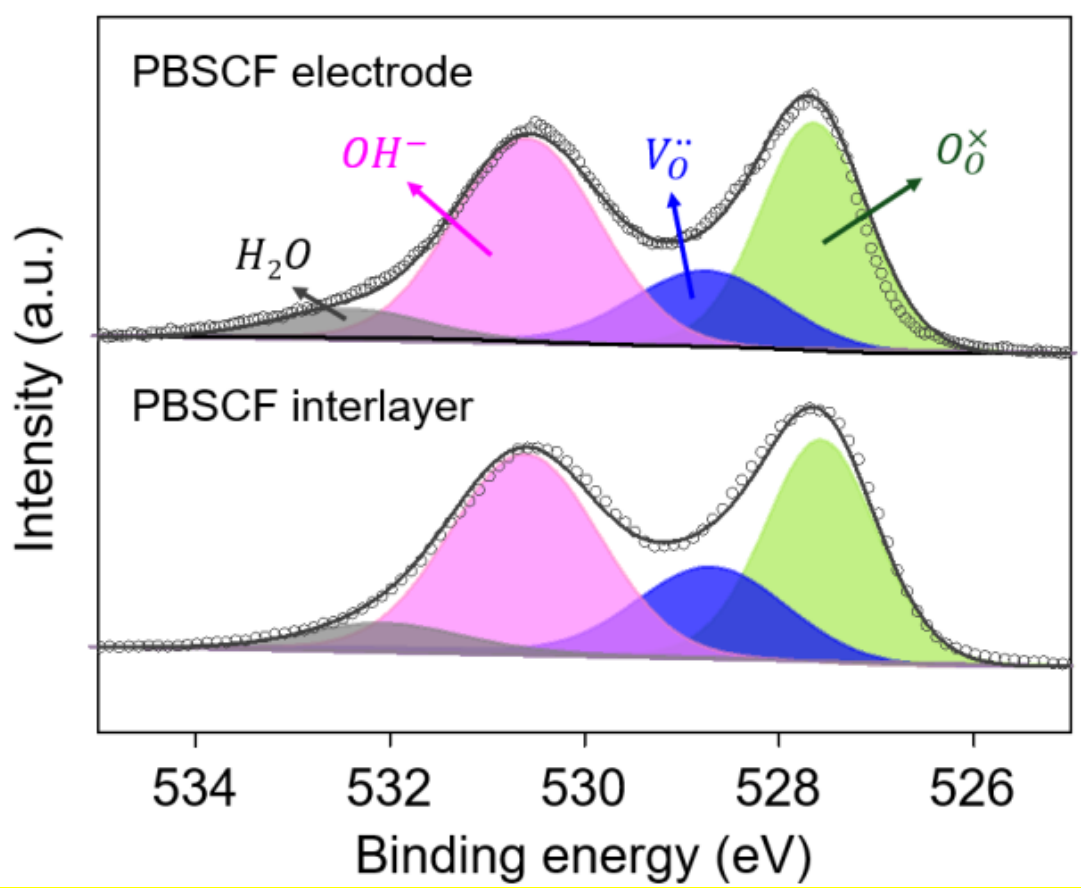

Supplementary Fig. 25. XPS O 1s spectra of PBSCF electrode and PBSCF interlayer.

Supplementary Table 7. Relative peak areas of each component in XPS O 1s spectra of PBSCF electrode and PBSCF interlayer.

	$O_O^\times$	$V_O^{\cdot\cdot}$	OH^-	H_2O
PBSCF electrode	0.36	0.16	0.42	0.06
PBSCF interlayer	0.35	0.18	0.41	0.06

Table R1. Atomic ratios of PBSCF electrode and PBSCF interlayer from XPS.

	Atomic ratio over (Pr+Ba+Sr+Co+Fe)				
	Pr	Ba	Sr	Co	Fe
PBSCF electrode	0.260	0.307	0.154	0.153	0.126
PBSCF interlayer	0.257	0.315	0.145	0.149	0.133

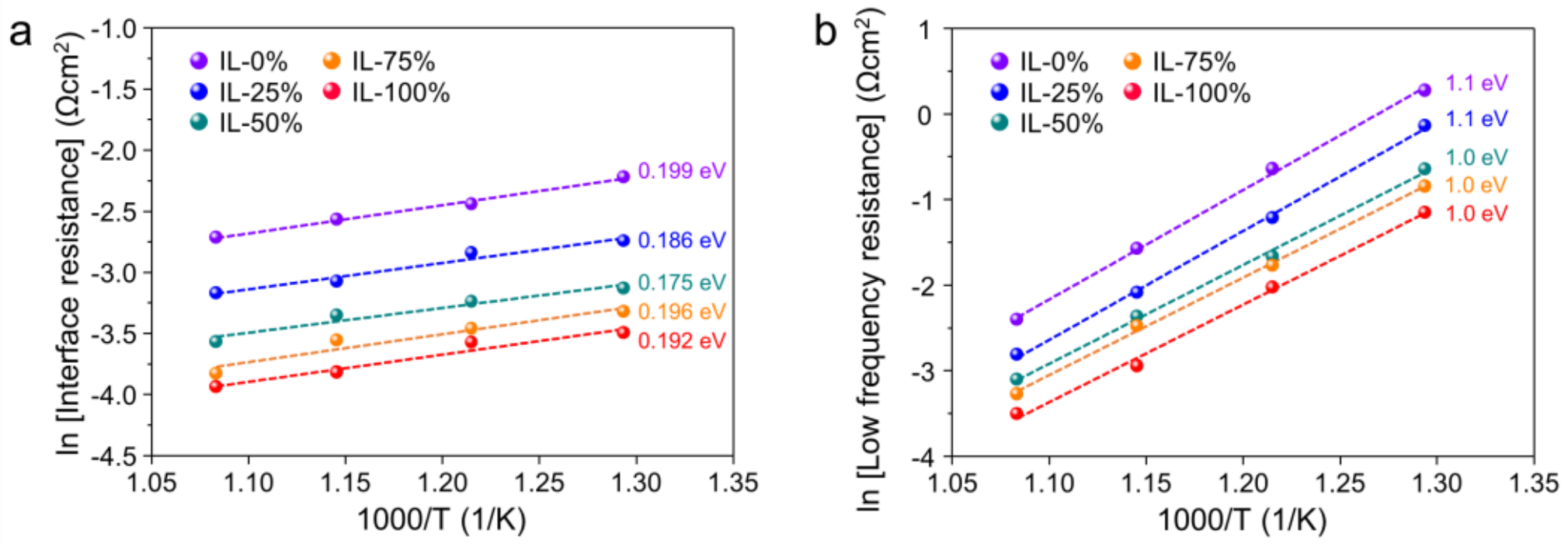

Fig. R3. Arrhenius plots of the (a) ohmic interface resistance and (b) polarization interface resistance for IL-0%, IL-25%, IL-50%, IL-75%, and IL-100% in the temperature range of 650–500 °C.

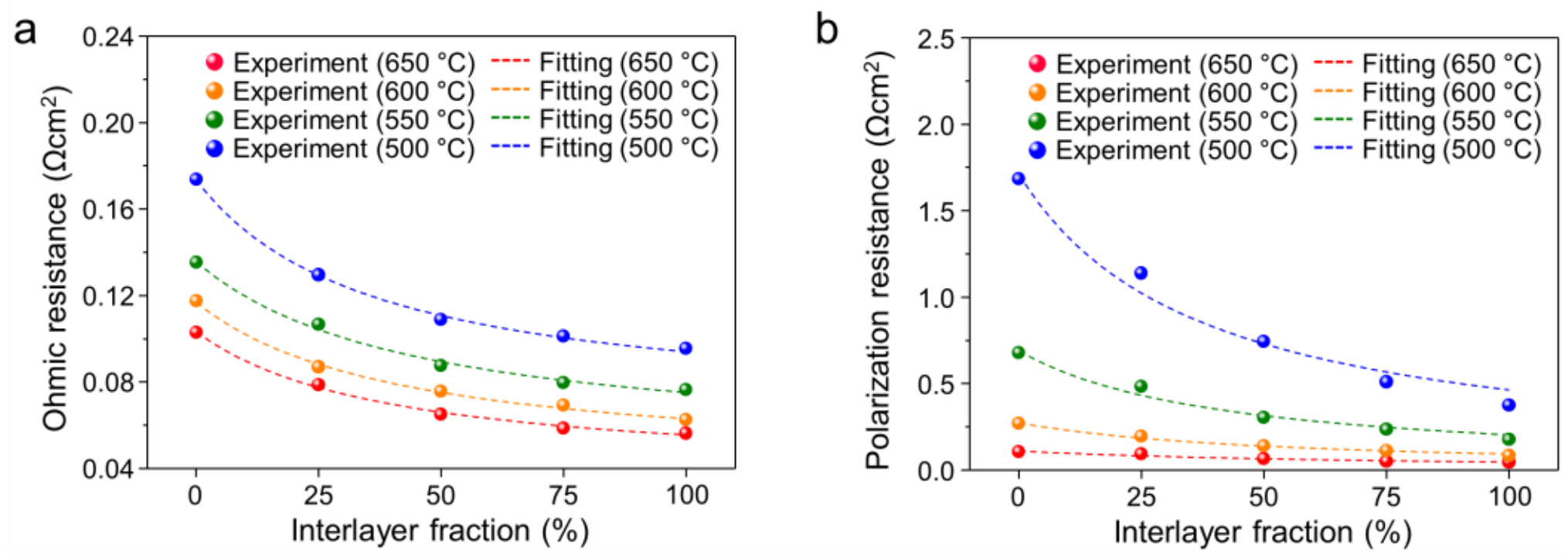

Fig. R4. (a) Ohmic resistance and (b) polarization resistance as a function of the interlayer fraction with corresponding fitting results.

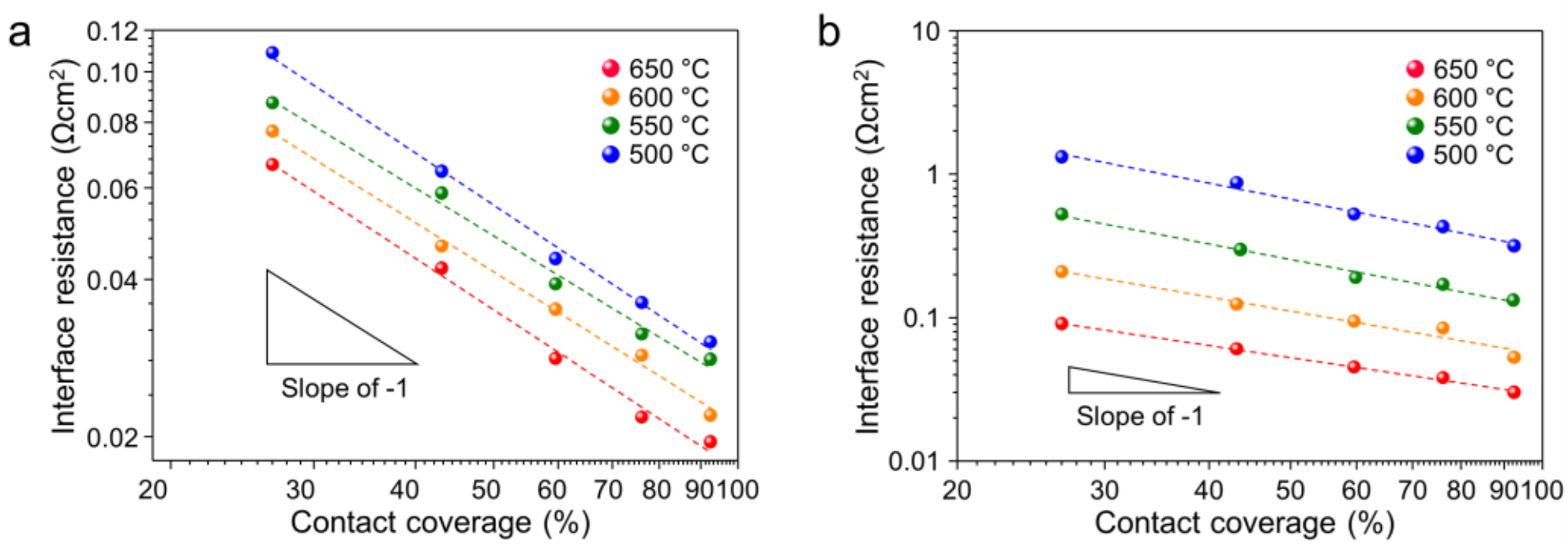

Fig. R5. Correlation between contact coverage and the interface components of (a) ohmic resistance and (b) polarization resistance in double logarithmic coordinates.

5. In Figure S26, the interfacial delamination appears quite severe, but the associated performance degradation seems relatively small. Then, does this imply that a certain degree of interfacial delamination may also have occurred even in samples that do not exhibit noticeable performance degradation?

Thank you for the comment. We agree that the interfacial delamination observed in Figure S33 (Figure S26 in the originally submitted Supplementary Information) may appear more severe than the corresponding electrochemical degradation. As a reviewer noted, a certain degree of microscale and localized delamination may exist even in samples without obvious performance degradation. However, if the effective contact network remains largely preserved, such localized delamination may not lead to pronounced performance degradation. In addition, the

apparent extent of delamination in the reference sample may be amplified during SEM sample preparation, because weakly bonded interfaces are more vulnerable to additional damage during cutting or polishing. In contrast, the interlayer-engineered samples remained largely intact under the same preparation conditions, indicating substantially greater resistance to the interfacial delamination. Similar apparent discrepancies between post-mortem delamination morphology and electrochemical degradation have also been reported in previous studies (Fig. R6).

[FIGURE REDACTED]

Fig. R6. (a,b) Nyquist plots during the thermal cycling test and the corresponding cross-sectional SEM image of the air electrode–electrolyte interface after the test. (c,d) The corresponding results for the other sample. [R5 and R6]

[R5] Yang, H. et al. Hydration-induced stiffness enabling robust thermal cycling of high-temperature fuel cell cathodes. *Nat. Commun.* **16**, 3154 (2025).

[R6] Shi, K. et al. A core–shell perovskite composite air electrode with thermal-expansion offset and mechanical support functions for highly durable reversible protonic ceramic cells.

Adv. Mater. **37**, 2419224 (2025).

6. Although authors argue that the approach in the manuscript can be applicable to other electrode materials, no direct validation is provided. Even a limited demonstration using other electrode materials would significantly strengthen the universality of the proposed precursor-based interlayer strategy.

Thank you for the important comment. We agree that demonstrating applicability beyond a single electrode material is necessary to support and strengthen the generality of the proposed interlayer strategy.

To address this point, we have performed additional experiments using three representative electrode materials with distinct characteristics: a triple conducting oxide (TCO), $Gd_{0.3}Ca_{2.7}Co_{3.82}Cu_{0.18}O_{9-\delta}$ (GCCCO), and a mixed ionic and electronic conductor (MIEC), $Ba_{0.5}Sr_{0.5}Co_{0.8}Fe_{0.2}O_{3-\delta}$ (BSCF), as an air electrode in protonic ceramic fuel cells (Ni-BZCYYb/BZCYYb/air electrode), and $La_{0.6}Sr_{0.4}CoO_{3-\delta}$ (LSC) as an air electrode in a conventional solid oxide fuel cell (Ni-YSZ/YSZ/GDC/air electrode) [R7-R9]. In all cases, a precursor-based interlayer was deposited using the corresponding electrode precursors, forming a conformal, dense, and thin layer similar to PBSCF-based interlayer in the manuscript, confirming that the fabrication approach is not limited to the specific material. (Fig. S28).

Electrochemical measurements show that the introduction of the interlayer consistently enhances the peak power density in all electrode materials. (Fig. S29). In particular, the ohmic resistance exhibits a comparable reduction for all electrode materials, indicating that improved

interfacial contact coverage consistently reduces ohmic resistance regardless of the electrode material (Fig. R7a). The polarization resistance also decreases for all electrode materials, although the magnitude of reduction varies, reflecting differences in intrinsic reaction kinetics (Fig. R7b). These results demonstrate that the proposed interlayer strategy is not limited to a specific electrode material, but is broadly applicable to different electrode materials, supporting the generality of the proposed interface engineering approach.

We have revised the manuscript and Supplementary Information as follows:

[line 2, page 11 in the revised manuscript]

These values represent the highest reported values for state-of-the-art PCECs, especially those operating below 500 °C (Figs. 3(c and f) and Supplementary Table 8).[1,5-10,36-43] To further examine the generality of the interface-engineering strategy, the precursor-based interlayer strategy was further tested with additional air electrode materials, including $Gd_{0.3}Ca_{2.7}Co_{3.82}Cu_{0.18}O_{9-\delta}$ (GCCCO)[9], $Ba_{0.5}Sr_{0.5}Co_{0.8}Fe_{0.2}O_{3-\delta}$ (BSCF)[44], and $La_{0.6}Sr_{0.4}CoO_{3-\delta}$ (LSC)[45] (Supplementary Figs. 28 and 29). The consistent performance improvements observed in these systems indicate that the interlayer strategy is not limited to a specific electrode material and support its broad applicability. This generality is particularly important because, as PCECs increasingly adopt thinner electrolytes to enhance performance, the interfacial resistance becomes more critical due to its independence from the electrolyte thickness. In addition, at lower temperatures, polarization resistance, primarily governed by interfacial processes, becomes the major performance-limiting factor. Therefore, the interlayer-based

approach offers a scalable and effective strategy to overcome the key challenges in advancing high-performance, low-temperature PCECs.

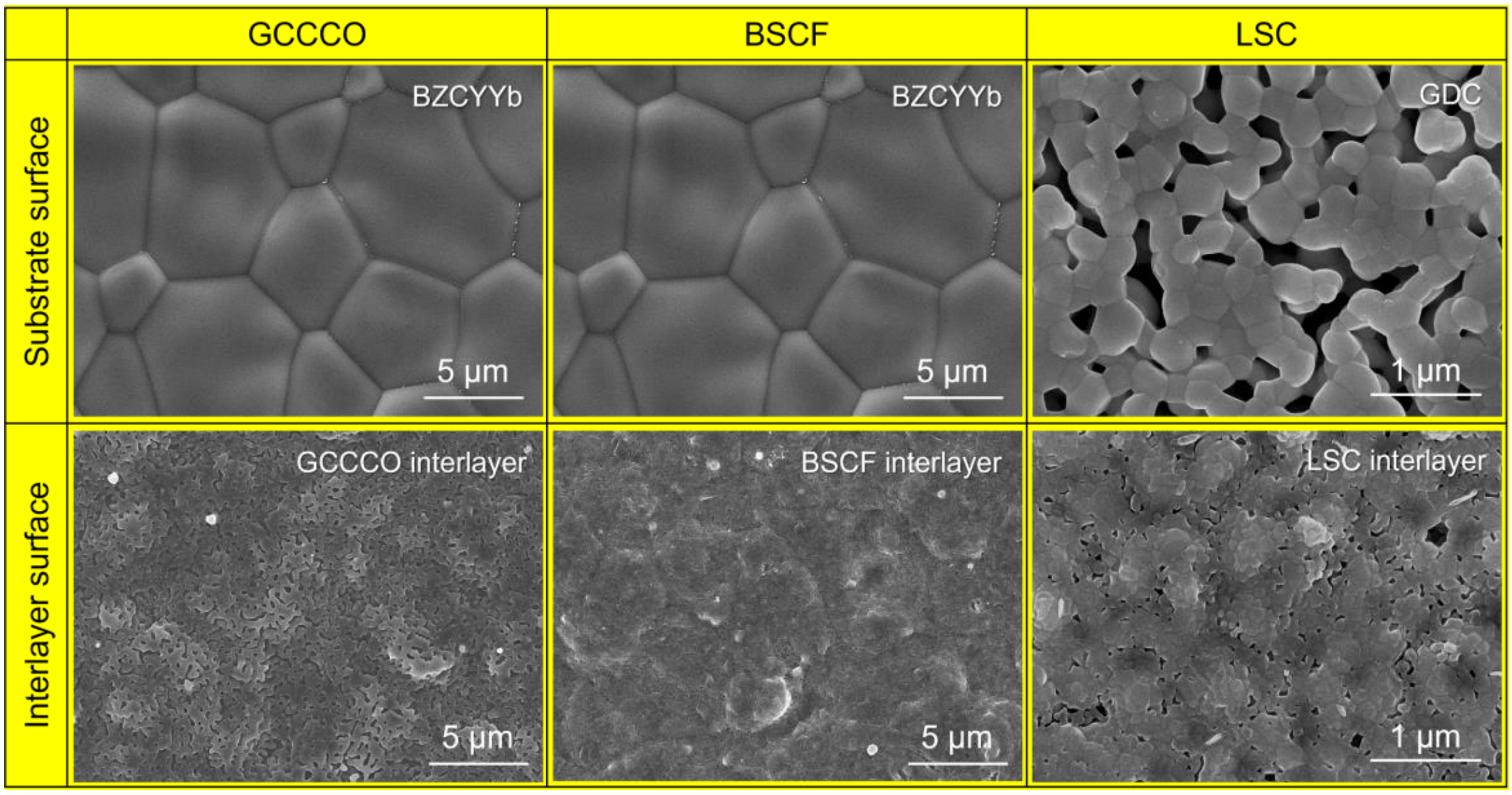

Supplementary Fig. 28. Surface SEM images of the substrates and the precursor-based interlayers.

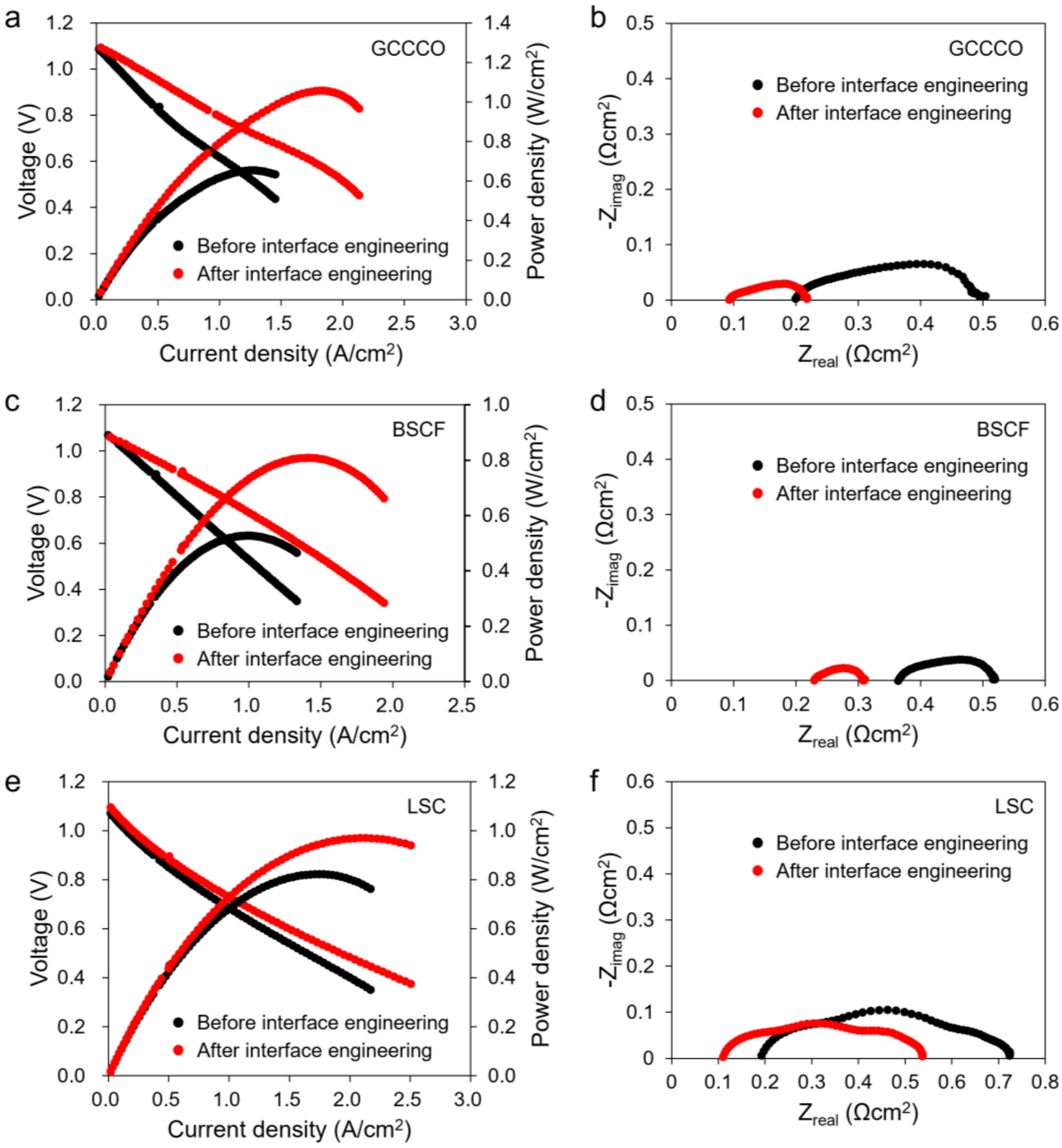

Supplementary Fig. 29. (a) I-V-P curves of the GCCCO interlayer in a Ni-BZCYYb/BZCYYb/GCCCO configuration at 600 °C and (b) Nyquist plots under OCV conditions. (c) I-V-P curves of the BSCF interlayer in a Ni-BZCYYb/BZCYYb/BSCF configuration at 600 °C and (d) Nyquist plots under OCV conditions. (e) I-V-P curves of the LSC interlayer in a Ni-YSZ/YSZ/GDC/LSC configuration at 650 °C and (f) Nyquist plots under OCV conditions.

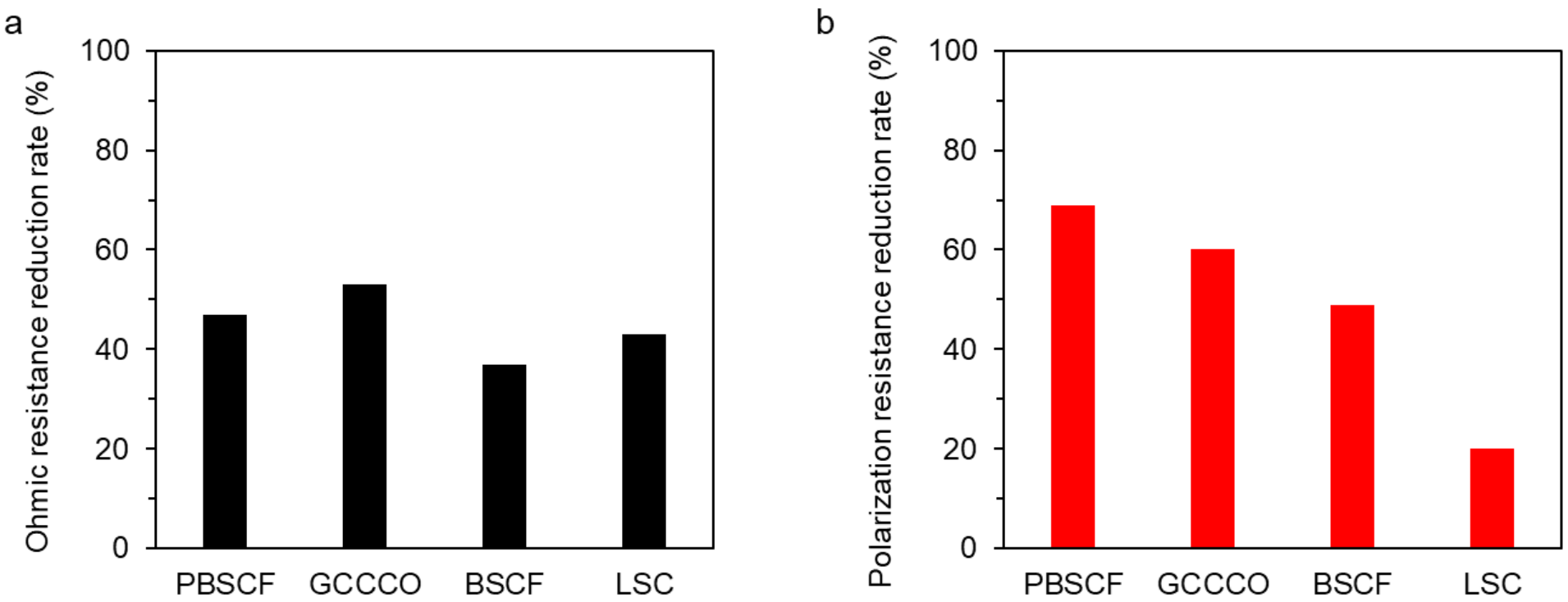

Fig. R7. Reduction rates of (a) ohmic and (b) polarization resistances upon introduction of the interlayer for various electrode materials.

[R7] Saqib, M. et al. Transition from perovskite to misfit-layered structure materials: a highly oxygen-deficient and stable oxygen electrode catalyst. *Energy Environ. Sci.* **14**, 2472–2484 (2021).

[R8] Liu, Z. et al. Strategic atomic trapping at heterointerfaces for protonic ceramic cells. *Nat. Commun.* **16**, 10405 (2025).

[R9] Rehman, S. U. et al. Designing the nano-scale architecture of the air electrode for high-performance and robust reversible solid oxide cells. *Appl. Catal. B* Environ. Energy **333**, 122784 (2023).

Reviewer #2:

The manuscript proposes a precursor-based interfacial engineering strategy enabled by ESD to enhance the performance and durability of PCECs. By fabricating an ultrathin, dense PBSCF interlayer, the authors improve interfacial contact coverage and reduce both ohmic and polarization resistances, while enhancing mechanical strength and thermal-cycling robustness. Overall, the study is timely and potentially impactful from both engineering and methodological perspectives. With substantial revisions and additional supporting evidence, the manuscript may be suitable for publication.

We thank the reviewer for the positive and constructive assessment of our work. We appreciate the reviewer's recognition of its significance and potential. In response to the comments, we have carefully revised the manuscript. Additional experiments and analyses were also performed to address the concerns and strengthen the supporting evidence. Detailed point-by-point responses are provided below.

1. Please justify the rationale for employing a co-firing strategy instead of pre-crystallizing the interlayer, specifically clarifying whether interfacial defects arising from precursor decomposition/shrinkage mismatch were present.

Thank you for the comment. To justify the co-firing strategy, we fabricated a pre-crystallized interlayer and compared structural and electrochemical characteristics with a co-fired interlayer. The pre-crystallized interlayer was prepared in two sintering steps; the interlayer was first sintered at 950 °C for 4 h, followed by air electrode deposition and a second sintering at 950 °C

for 4 h. In contrast, the co-fired interlayer was prepared in a single sintering step at 950 °C for 4 h. The pre-crystallized and co-fired interlayers show no significant differences in their structural and electrochemical characteristics. Cross-sectional SEM images (Fig. R8a and b) revealed no noticeable interfacial defects, such as cracks or voids, in both interlayers, indicating that precursor decomposition and shrinkage mismatch did not introduce detectable interfacial damage under the present conditions. This comparable structural integrity was also reflected in the electrochemical performance, where both interlayers show nearly identical peak power density, ohmic resistance, and polarization resistance (Fig. R8c and d). These results demonstrate that the co-firing strategy preserves interfacial quality and electrochemical performance, while offering a simpler and more practical processing approach.

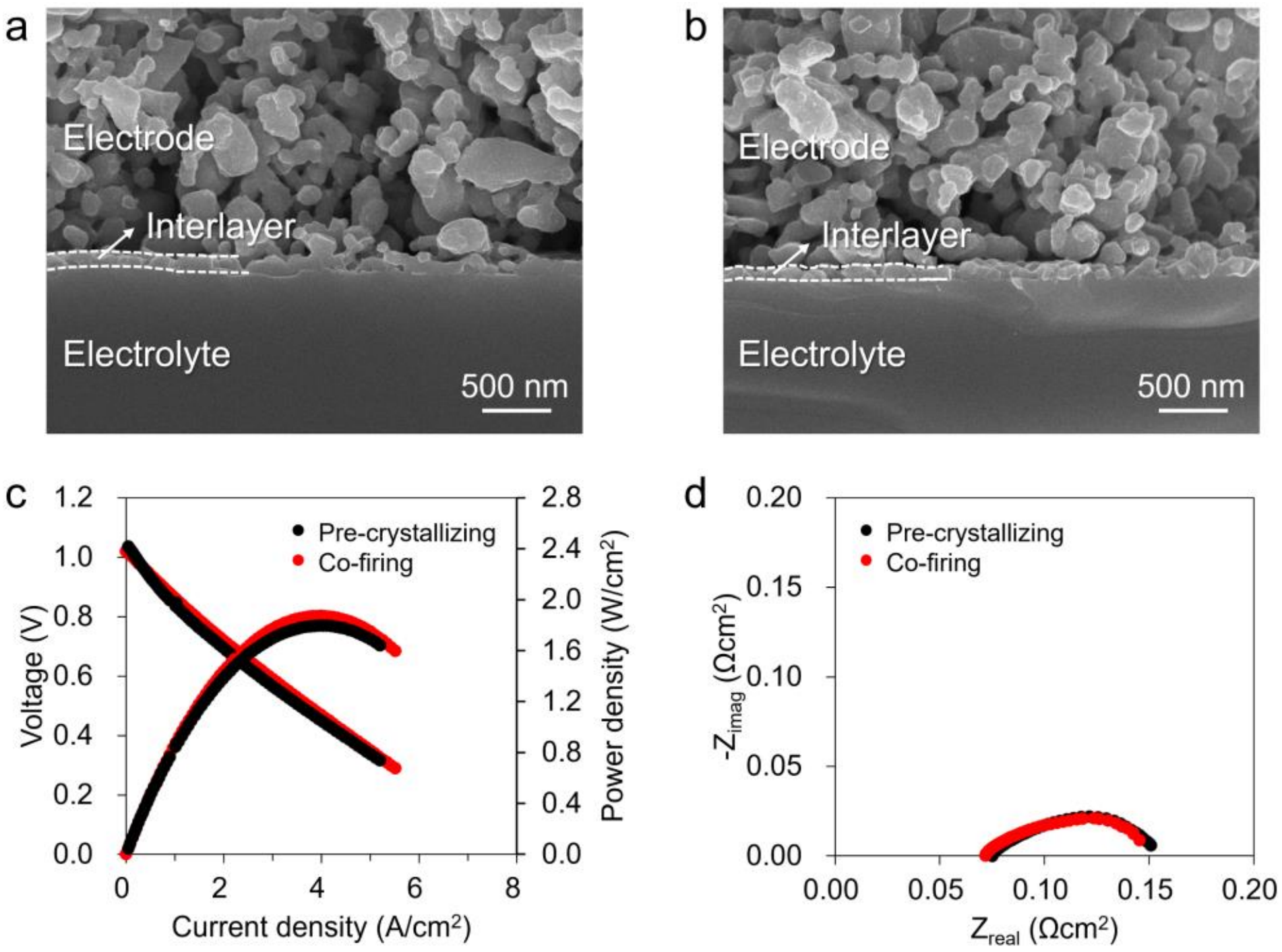

Fig. R8. Cross-sectional SEM images of (a) the co-fired interlayer and (b) the pre-crystallized interlayer. (c) I-V-P curves of the corresponding samples at 600 °C, and (d) Nyquist plots under OCV conditions.

2. In Fig. 2c, the most pronounced reduction in resistance occurs in the low-frequency regime, which is typically associated with surface oxygen kinetics or mass transport. Since the interlayer is primarily introduced to improve interfacial physical contact, please clarify the causal link between them.

Thank you for the comment. As the reviewer noted, in low-temperature PCECs employing triple-conducting oxide (TCO) air electrodes, the dominant contribution to polarization resistance arises primarily from oxygen surface exchange reactions at the air electrode. In our system, the role of the interlayer is therefore not limited to improving interfacial physical contact with the electrolyte, but also includes providing an additional surface that can participate in oxygen surface exchange reactions, thereby expanding the effective reaction area.

In our system, the precursor-based interlayer forms a thin, dense, and nearly two-dimensional structure, with a specific surface area of 1.066 ± 0.019 $\mu m^2/\mu m^2$ (Fig. S9), indicating that the interlayer surface area scales approximately with the underlying contact coverage. Therefore, contact coverage can be reasonably used as an effective descriptor of the accessible reaction sites, which explains why polarization resistance can also be expressed as a function of contact coverage. This interpretation is supported by the Bode plots (Fig. R9), which exhibit a dominant impedance response in the 10^{-1}–10^{1} Hz range, characteristic of oxygen surface exchange reactions in low-temperature PCECs with TCO air electrodes. [R10-R11] Similar frequency-domain behavior has also been reported for other state-of-the-art PCECs (Fig. R10). DRT analysis of the model samples (Fig. S22) shows that the most pronounced decrease with increasing interlayer fraction occurs in the low-frequency response (10^{-1}–10^{1} Hz), confirming that the reduction in polarization resistance is dominated by oxygen surface exchange reactions. Lastly, the Arrhenius plots (Fig. S23) reveal a nearly constant activation energy of 1.04 ± 0.06

eV across all model samples, indicating that the reduction in polarization resistance stems primarily from the structural modification (increase in accessible reaction sites), rather than from changes in the intrinsic reaction kinetics.

The related description was provided in the originally submitted manuscript and Supplementary Information as follows:

[line 13, page 8 in the revised manuscript]

This relationship is consistent with the unique characteristics of PCECs, where the low-temperature operation and use of triple conducting oxide (TCO) electrodes make surface oxygen exchange reactions the rate-determining step (RDS) in polarization resistance.[31-33] This was clearly supported by the Bode plots (Fig. 2(c) and Supplementary Fig. 19), exhibiting a dominant impedance response at a specific frequency at approximately 10^{-1}–10^{1} Hz, typical of surface exchange reactions.[11,30] Therefore, the interlayer contributes to performance enhancement not only by increasing the contact coverage with the underlying electrolyte, but also creating additional surface reaction sites above the interlayer (Supplementary Fig. 20). The 2D morphology of the interlayer, confirmed by a specific surface area of 1.066 ± 0.019 $\mu m^2/\mu m^2$ by AFM analysis (Supplementary Fig. 9), enables these surface reaction sites to scale proportionally with the contact coverage. This structural feature underpins the strong correlation between the contact coverage and polarization resistance, enabling a quantitative assessment of interfacial contribution to electrochemical performance.

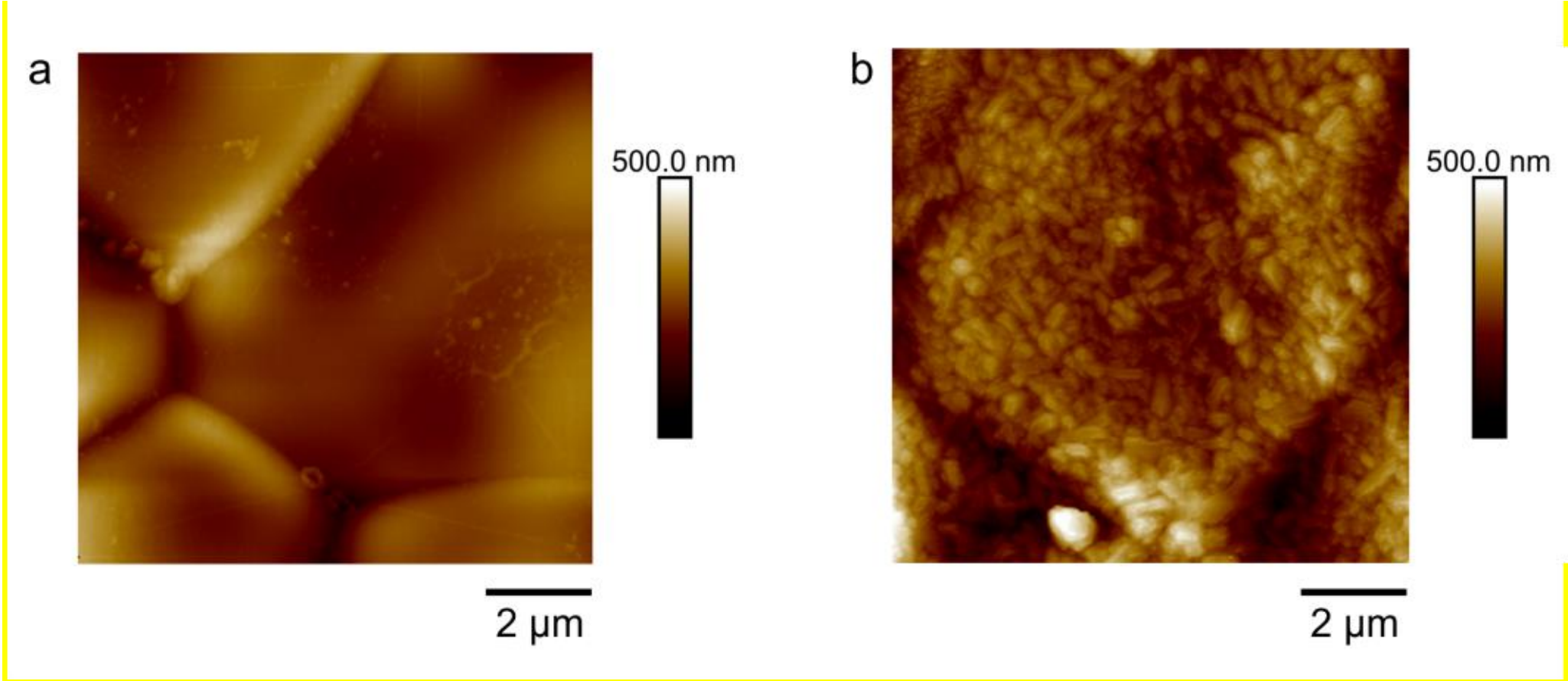

Supplementary Fig. 9. AFM images of (a) the pristine electrolyte surface and (b) the precursor-based interlayer surface. The pristine electrolyte surface exhibited an RMS roughness of 46.7 ± 16.8 nm and a specific surface area of 1.010 ± 0.010 $\mu m^2/\mu m^2$, while the precursor-based interface showed an RMS roughness of 48.6 ± 9.5 nm and a specific surface area of 1.066 ± 0.019 $\mu m^2/\mu m^2$.

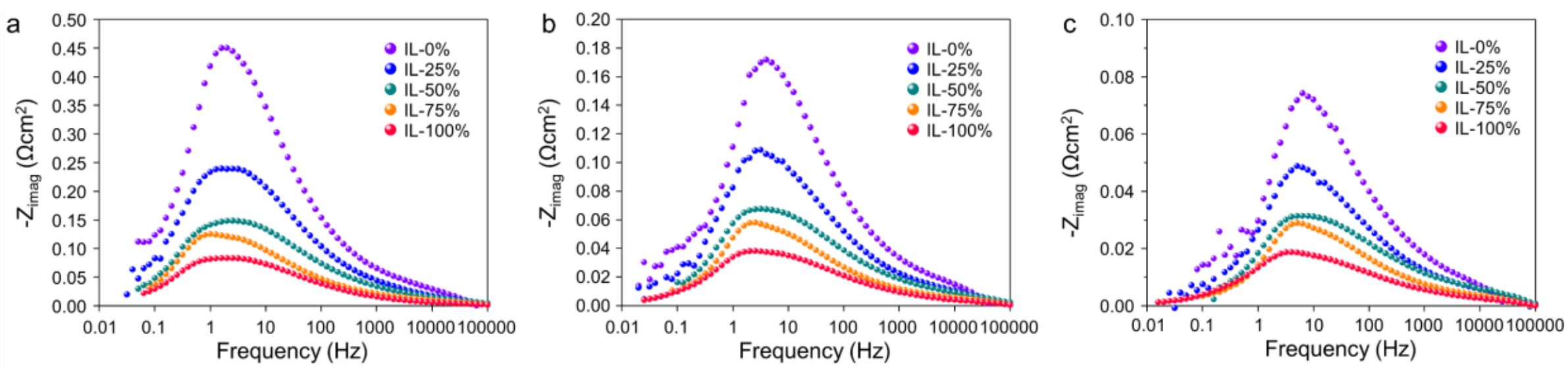

Fig. R9. Bode plots of the model samples under OCV conditions at (a) 500 °C, (b) 550 °C, and (c) 600 °C.

[FIGURE REDACTED]

Fig. R10. (a) DRT analysis and (b) Nyquist plots of previously reported PCECs under OCV conditions. The positions of -log(τ)=0 and -log(τ)=2 s in (a) correspond to 0.159 and 15.9 Hz, respectively. [R12 and R13]

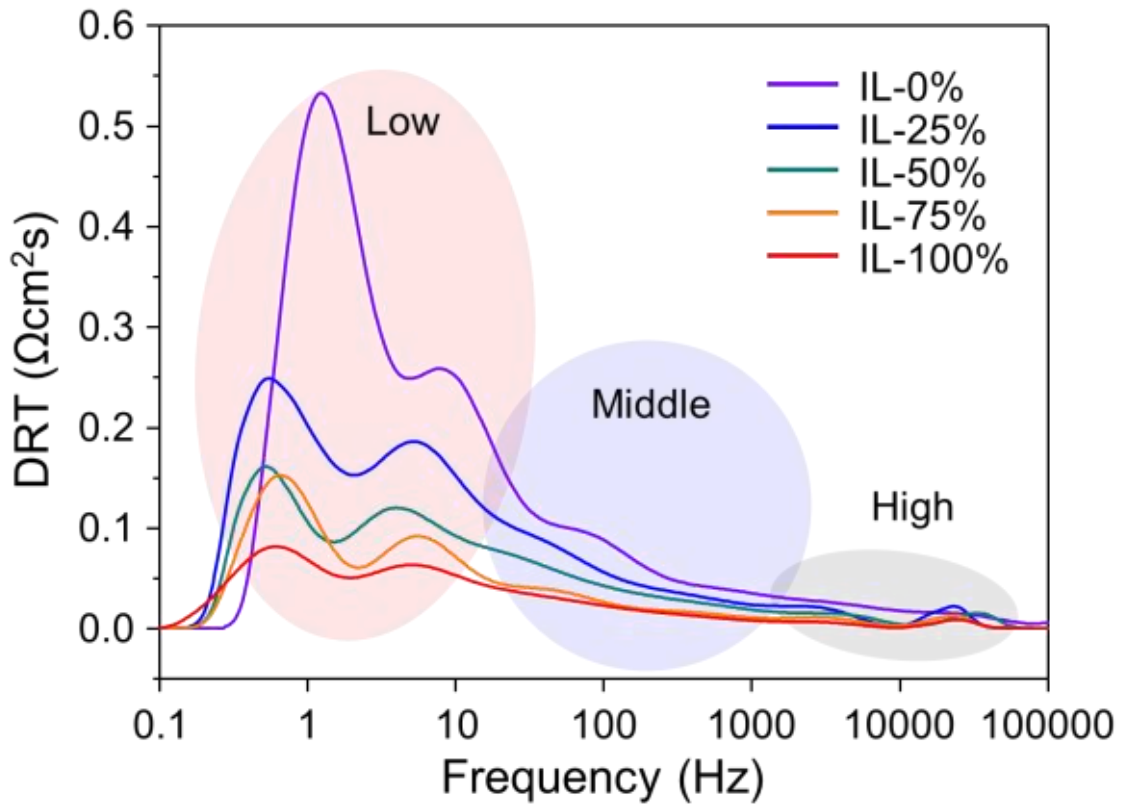

Supplementary Fig. 22. DRT analysis of the model samples at 500 °C under OCV conditions.

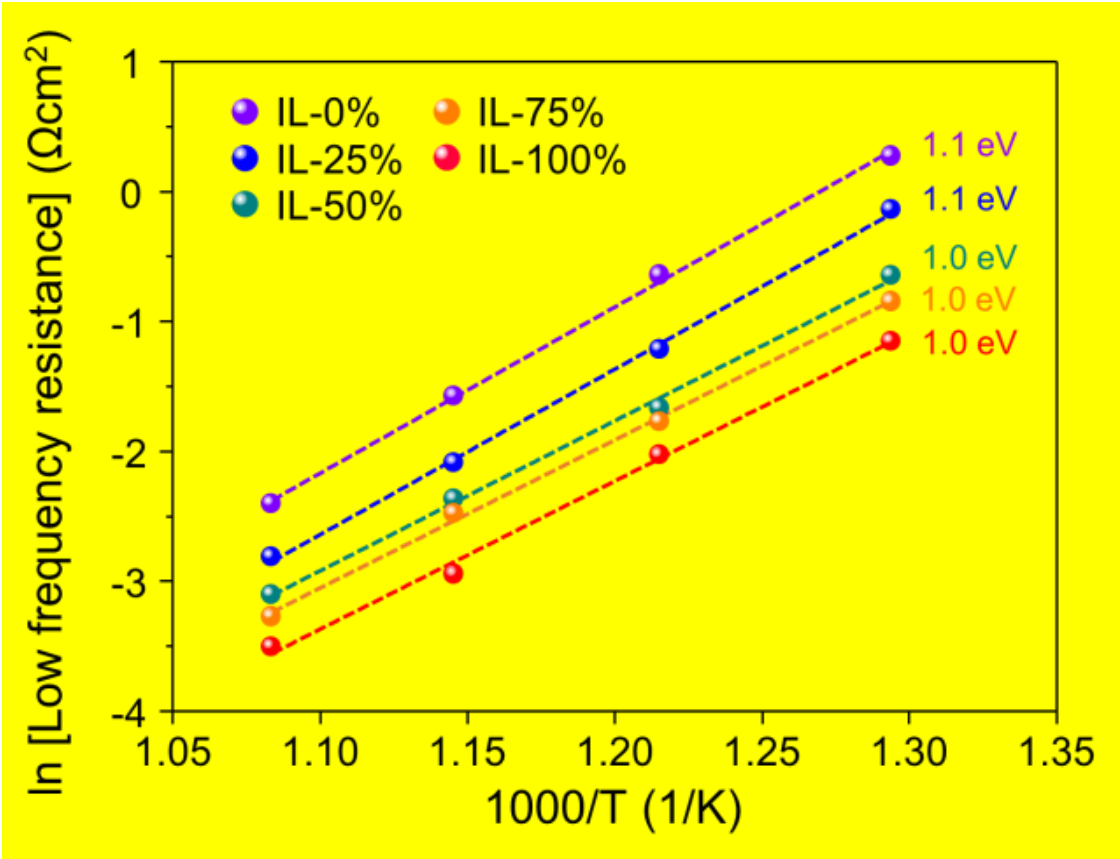

Supplementary Fig. 23. Arrhenius plots of the low-frequency resistances for IL-0%, IL-25%, IL-50%, IL-75%, and IL-100% in the temperature range of 650–500 °C.

[R10] Choi, M. et al. Interface engineering to operate reversible protonic ceramic electrochemical cells below 500 °C. *Adv. Energy Mater.* **15**, 2400124 (2025).

[R11] Choi, S. M. et al. Electrochemical analysis of high-performance protonic ceramic fuel cells based on a columnar-structured thin electrolyte. *Appl. Energy* **233**, 29–36 (2019).

[R12] Yun, J. et al. Synergistic optimization of power output and thermomechanical stability enabled by rational cathode design in protonic ceramic fuel cells. *Adv. Funct. Mater.* e25258 (2025).

[R13] Zhao, S. et al. Reverse atom capture on perovskite surface enabling robust and efficient cathode for protonic ceramic fuel cells. *Adv. Mater.* **36**, 2405052 (2024).

3. The activation energy should be calculated from the Arrhenius plots and explicitly labeled in Fig. 2f and Fig. 2i. This will allow for a more quantitative comparison of the reaction kinetics.

Thank you for the comment. We note that Figs. 2f and 2i are not Arrhenius plots, but log-log plots showing the relationship between contact coverage and the interface resistances at each temperature. These figures are used to show the coverage-dependent scaling behavior of the resistances, rather than their temperature dependence. As the reviewer suggested, we therefore calculated activation energies from the corresponding Arrhenius plots and explicitly added the values to Supplementary Information (Figs. S18 and S23). Both interfacial ohmic and polarization resistances exhibit nearly constant activation energies of 0.190 ± 0.009 eV and 1.04 ± 0.06 eV, respectively, across all samples. These results further support that the decrease in the interfacial resistances originates primarily from structural improvement through increased contact coverage, rather than from changes in intrinsic reaction kinetics.

We have revised the Supplementary Information as follows:

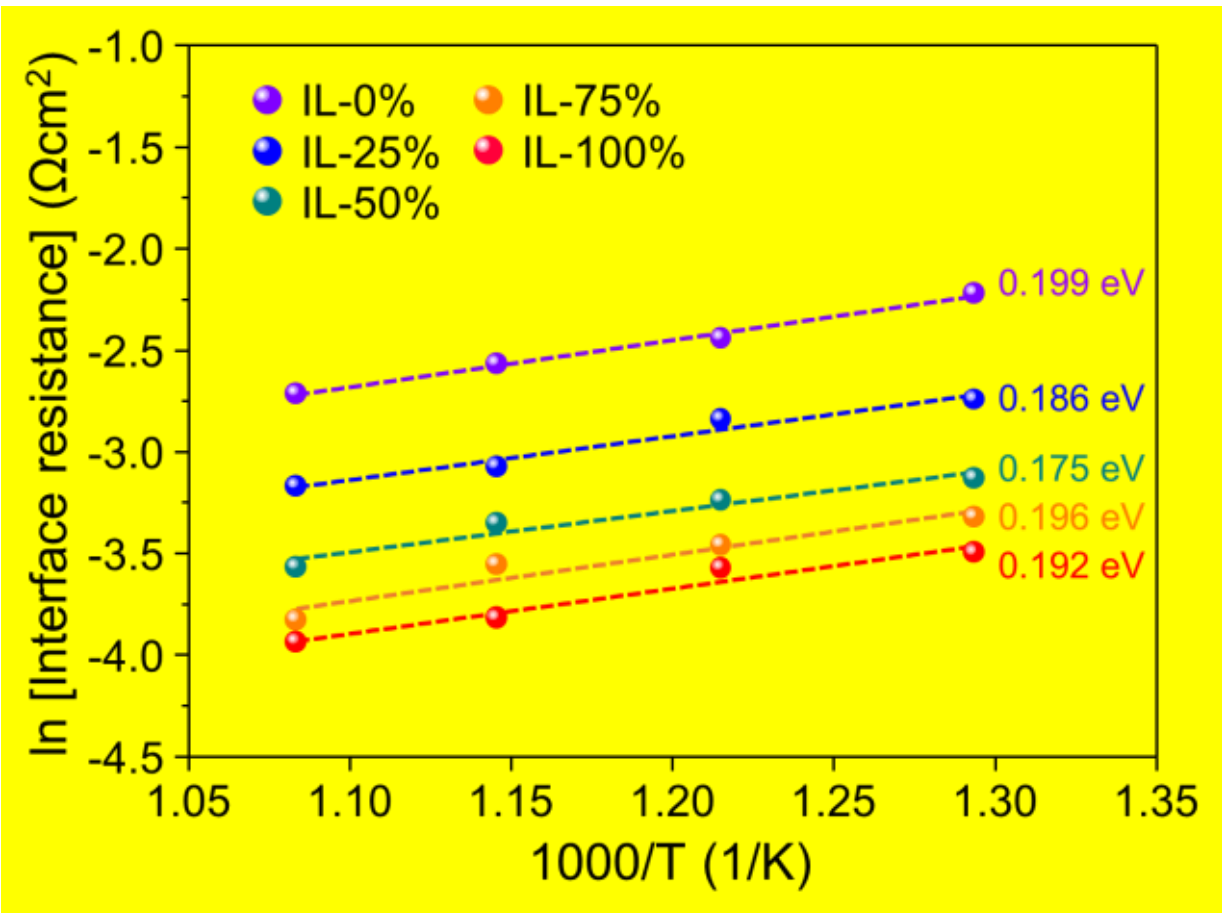

Supplementary Fig. 18. Arrhenius plots of the interfacial ohmic resistances for IL-0%, IL-25%, IL-50%, IL-75%, and IL-100% in the temperature range of 650–500 °C.

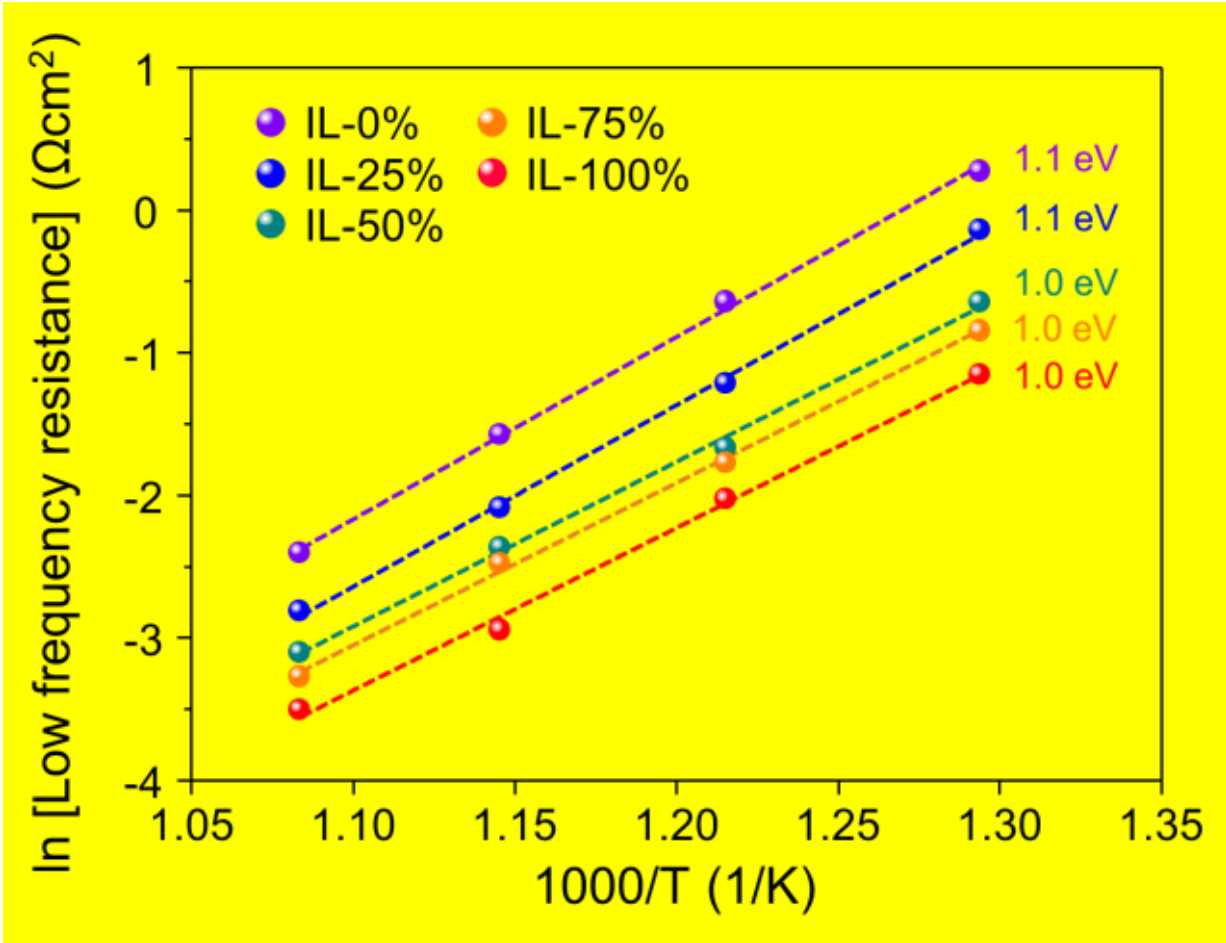

Supplementary Fig. 23. Arrhenius plots of the low-frequency resistances for IL-0%, IL-25%, IL-50%, IL-75%, and IL-100% in the temperature range of 650–500 °C.

4. The authors are encouraged to perform a DRT analysis of the post-cycling EIS for IL-0% in Fig. 4b and compare it with the pre-cycling spectrum, to quantitatively identify which frequency-domain process dominates the resistance increase process.

Thank you for the comment. As the reviewer suggested, we performed DRT analysis of the post-cycling EIS of IL-0% (Fig. 4b) to quantitatively identify which frequency-domain process dominates the resistance increase after thermal cycling. We also analyzed the corresponding post-cycling EIS of IL-100% for comparison. The resulting Bode plots, DRT spectra, and evolution of each frequency response are added in the revised Supplementary Information (Fig. S30).

For IL-0%, the most pronounced increase occurs in the low-frequency region, where the resistance increases from 0.15 Ωcm^2 before cycling to 0.38 Ωcm^2 after cycling, corresponding

to an increase of ~150%. As described in the original manuscript and Supplementary Information (line 24, page 8 in the revised manuscript), this frequency region was assigned to the surface charge transfer process based on the O_2 partial pressure-controlled DRT analysis (Fig. S21). The substantial increase in the low-frequency response therefore indicates that the dominant degradation after cycling is associated with the loss of electrochemically active reaction area, consistent with interfacial delamination. In contrast, IL-100% exhibits negligible changes in all frequency responses, confirming that the interlayer effectively stabilizes the interface during thermal cycling.

We have revised the manuscript and Supplementary Information as follows:

[line 6, page 13 in the revised manuscript]

Nyquist plots (Figs. 4(b and c)) revealed that IL-0% exhibited 113% and 106% increases in ohmic and polarization resistances after 100 h, respectively, whereas IL-100% maintained its initial resistances. Post-cycling DRT analysis showed that the degradation in IL-0% was dominated by a pronounced increase in the low frequency resistance, which is associated with the loss of electrochemically active reaction area (Supplementary Fig. 30). Commonly reported degradation mechanisms in PCECs include cation segregation toward the electrode surface[46-49], grain boundary weakening in the electrolyte[12,50,51], and electrode-electrolyte delamination[12,45].

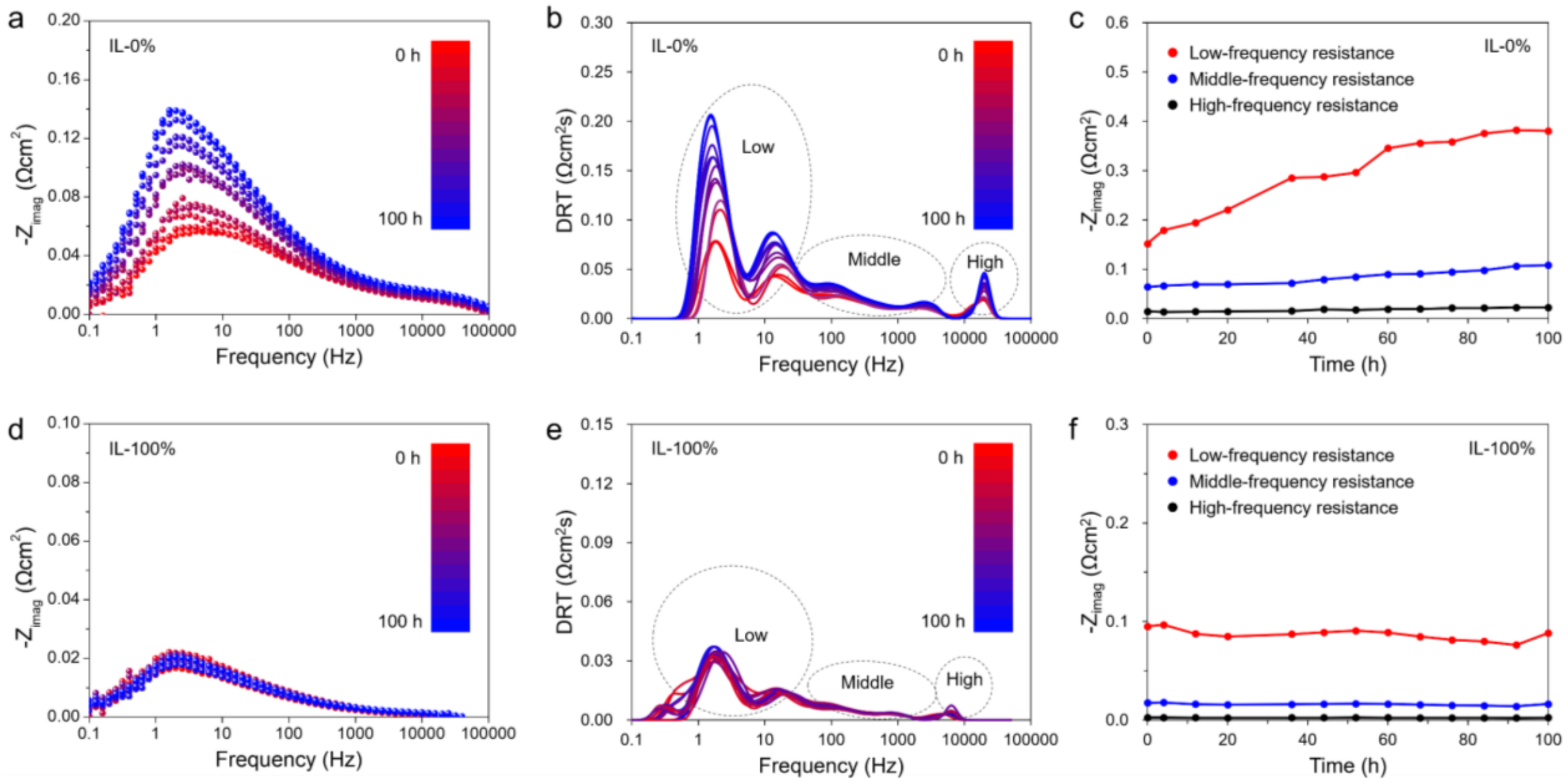

Supplementary Fig. 30. (a) Bode plots, (b) DRT analysis, and (c) the corresponding polarization resistances of low-, middle-, high-frequency regions during thermal cycling at 600 °C under OCV conditions of IL-0%. (d) Bode plots, (e) DRT analysis, and (f) the corresponding polarization resistances of low-, middle-, high-frequency regions during thermal cycling at 600 °C under OCV conditions of IL-100%.

5. It's suggested to discuss whether the adhesion strength exhibits a monotonic relationship with contact coverage and to provide post-test fracture surface morphology.

Thank you for the comment. In the originally submitted manuscript, adhesion strength measurements were performed only for IL-0% and IL-100%, as the intermediate samples (IL-25%, IL-50%, and IL-75%) were initially designed for model experiments to provide controlled variations in interfacial contact coverage for electrochemical evaluation.

Following the reviewer's suggestion, we have conducted additional mechanical pull-off tests for the intermediate samples to evaluate their adhesion strength. Optical images obtained after the pull-off tests confirm the complete detachment of the PBSCF electrode layer (Fig. R11). The post-test fracture surface morphology reveals that the detachment occurred at the electrode/electrolyte interface in regions without the interlayer, and at the electrode/interlayer interface in regions with the interlayer (Fig. R12). Raman spectroscopy confirms the clearly detached surfaces without the residual adhesive, validating the reliability of the characterization method (Fig. R13).

The measured adhesion strength exhibits a monotonic increase with increasing interlayer fraction, which is consistent with the increase in interfacial contact coverage (Fig. R14). This trend can be attributed to the increase in the load-bearing interface area with contact coverage, resulting in greater resistance to interfacial delamination. At the same time, we note that the intermediate samples (IL-25%, IL-50%, and IL-75%) represent multi-interface systems, where fracture behavior is governed by competing failure pathways between different interfaces. Accordingly, the measured adhesion strength may be influenced not only by the contact coverage but also by additional mechanical factors, such as local stress distribution and transition of fracture location between interfaces. [R14-R15] Therefore, although a clear monotonic trend is observed in the present data, this relationship should be interpreted as an experimentally observed correlation under the current conditions, rather than as a universally deterministic scaling law.

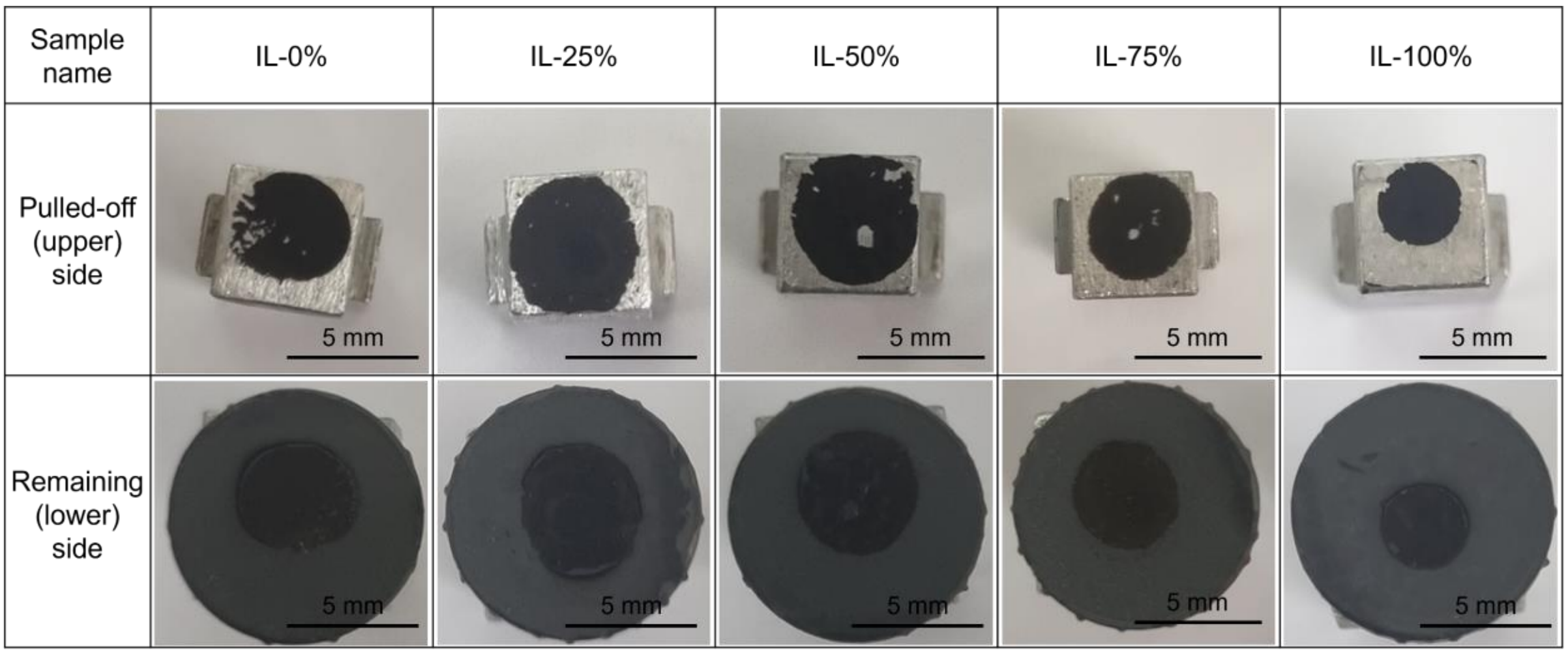

Fig. R11. Optical images obtained after the pull-off tests.

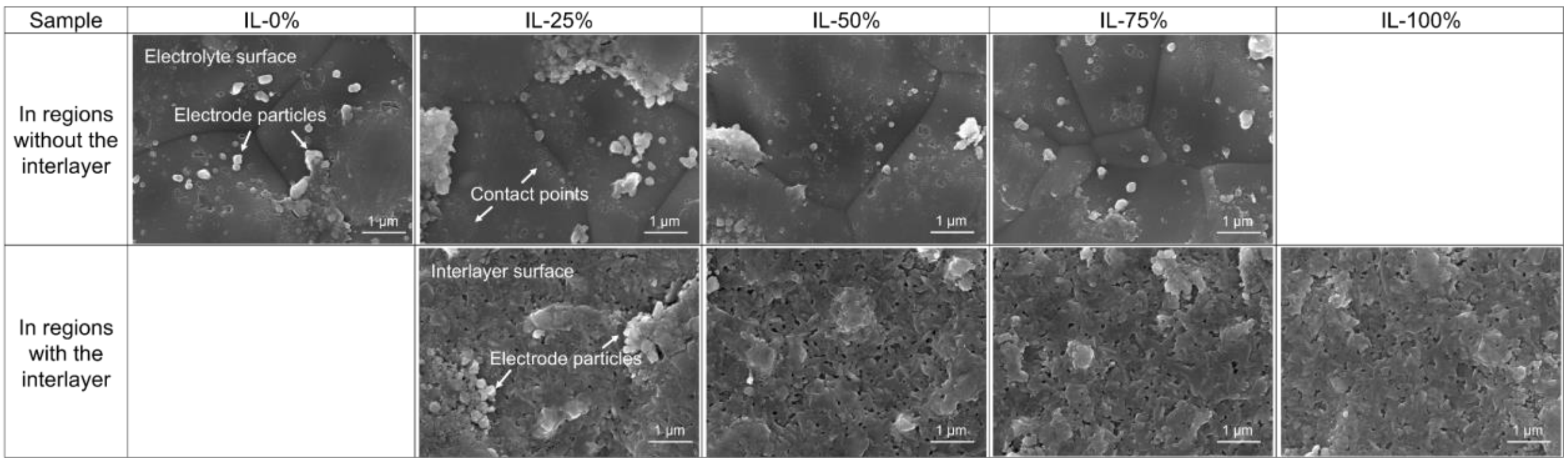

Fig. R12. Post-test fracture surface SEM images of IL-0%, IL-25%, IL-50%, IL-75%, and IL-100%.

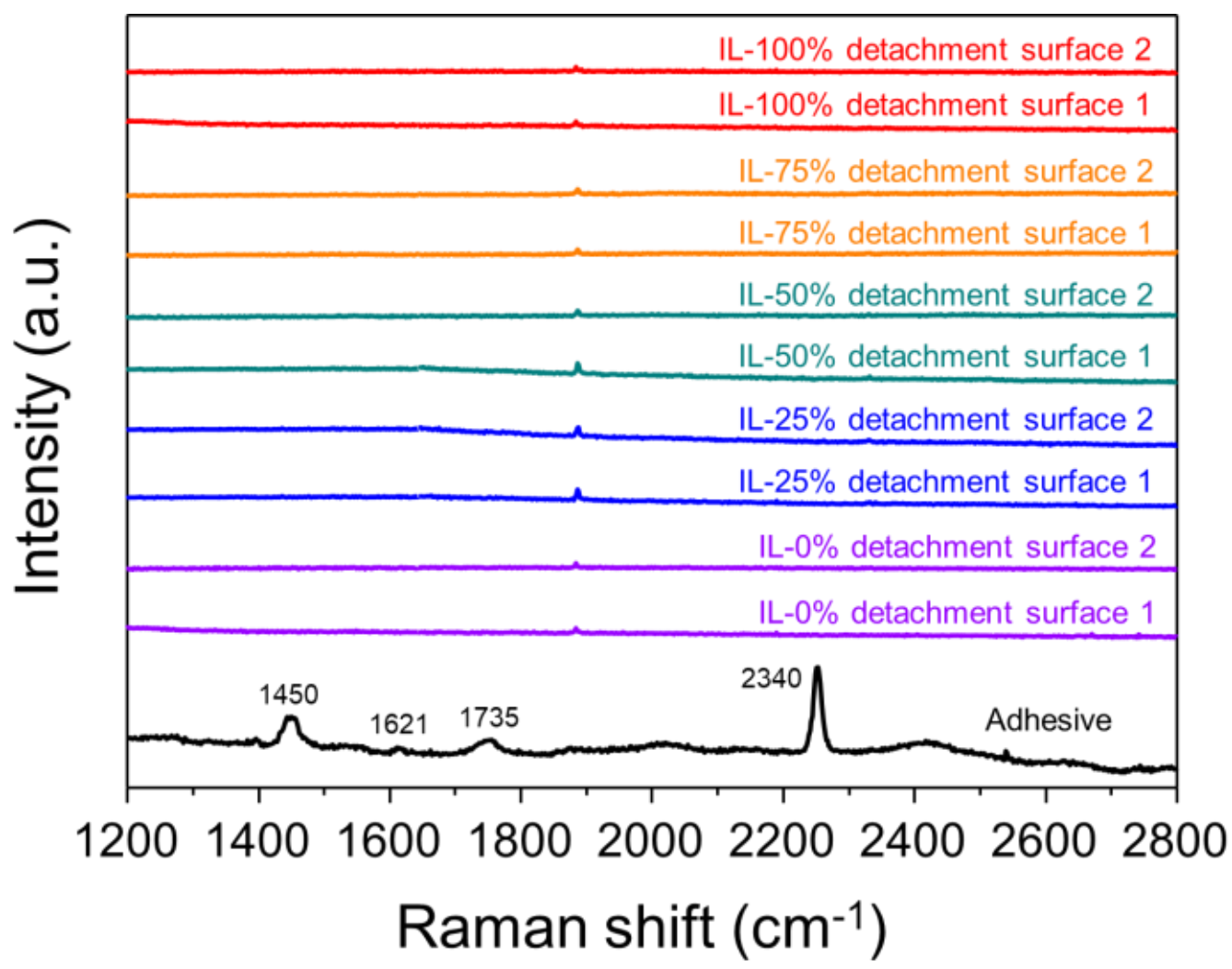

Fig. R13. Raman spectroscopy of the detached surfaces after the pull-off test and adhesive. The detachment surfaces on the electrolyte side and the electrode side were designated as detachment surface 1 and 2, respectively.

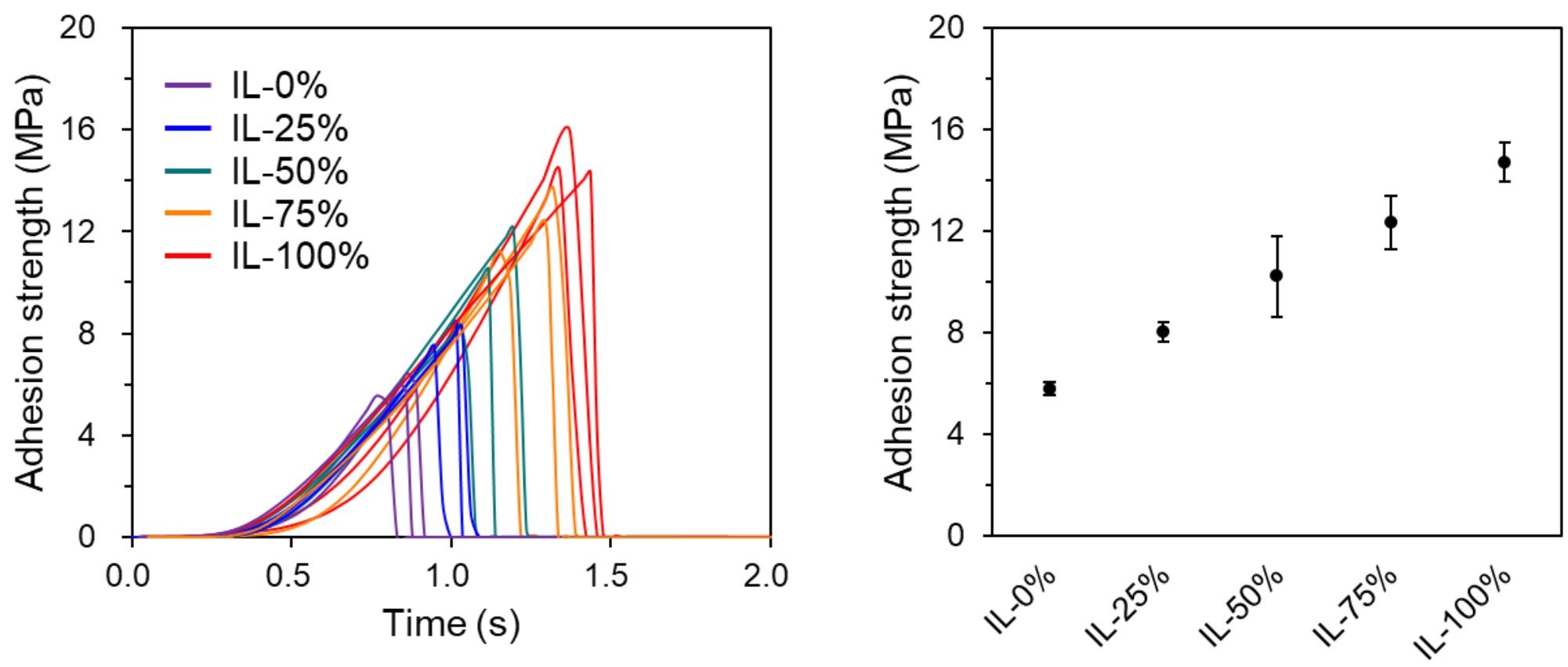

Fig. R14. Adhesion strengths at the interfaces of IL-0%, IL-25%, IL-50%, IL-75%, and IL-100% using the mechanical pull-off test.

[R14] Lassnig, A. et al. Nanoscale stress and microstructure gradients across a buckled Mo–Cu bilayer: Cu self-annealing triggered by interface delamination. *Acta Mater.* **283**, 120465

(2025).

[R15] Wei, Z. et al. Interfacial fracture of solid-state diffusion bonded Cr–Zircaloy bilayer materials by tensile test and FEM simulation. *J. Nucl. Sci. Technol.* **62**, 1023–1037 (2025).

6. In fig.5(d, e) and fig.5(g, h), it is noted that the IL-100% cells exhibit a slightly lower OCV compared to the IL-0% reference, please explain the underlying reason.

Thank you for the comment. The slight difference in OCV is most likely due to minor variations in cell fabrication and/or gas sealing, rather than the intrinsic difference between cells. To avoid any misunderstanding, we repeated the measurements using newly prepared cells, and the results are nearly identical to the original data.

We have revised the manuscript and the related Supplementary Information as follows:

[line 11, page 15 in the revised manuscript]

The PCEC with the interlayer demonstrated excellent fuel flexibility, operating effectively with alternative fuels such as ammonia and methane, both considered promising hydrogen carriers due to their higher volumetric energy density and convenient storage and transportation.[39,52-55] Without additional cracking or reforming catalysts, IL-100% achieved a peak power density of

0.76 W/cm^2 with direct ammonia fuel at 500 °C, which was 2.2 times higher compared to that of IL-0% (0.34 W/cm^2) (Figs. 5(d and e)). A similar enhancement was observed with methane: at a steam-to-carbon ratio (S/C) of 2 without additional reforming catalysts, IL-100% achieved a peak power density of 0.50 W/cm^2 at 500 °C, which was 2.1 times higher compared to that of IL-0% (0.24 W/cm^2) (Figs. 5(g and h)).

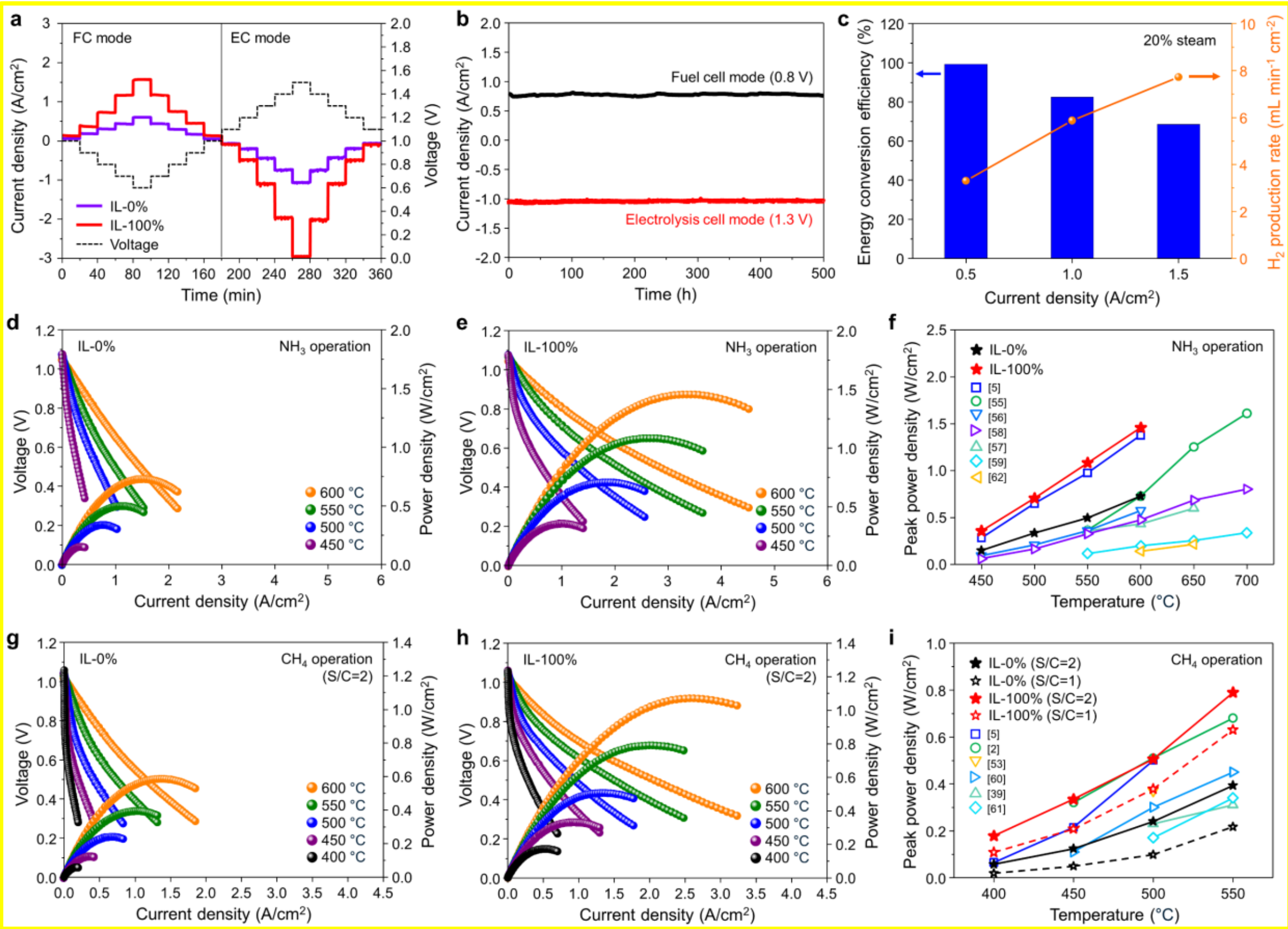

Fig. 5: Exceptionally high-performance and robust PCEC platform demonstrating a versatile energy conversion capability. (a) Current densities under voltage ranges of 0.6–1.0 V (FC mode) and 1.1–1.5 V (EC mode) at 500 ˚C. (b) Long-term stability test at 500 °C under constant voltages of 0.8 V (FC mode) and 1.3 V (EC mode). (c) Energy conversion efficiency and hydrogen production rate of IL-100% evaluated at different current densities under 20%

steam (air electrode) at 500 °C. I-V-P curves using ammonia fuel for (d) IL-0% and (e) IL-100% in the temperature range of 600–450 °C. (f) Comparison of peak power densities with previously reported protonic ceramic fuel cells operating with ammonia. I-V-P curves using methane fuel with a stem-to-carbon ratio of 2 for (g) IL-0% and (h) IL-100% in the temperature range of 600–400 °C. (i) Comparison of peak power densities with previously reported protonic ceramic fuel cells and solid oxide fuel cells operating with methane.

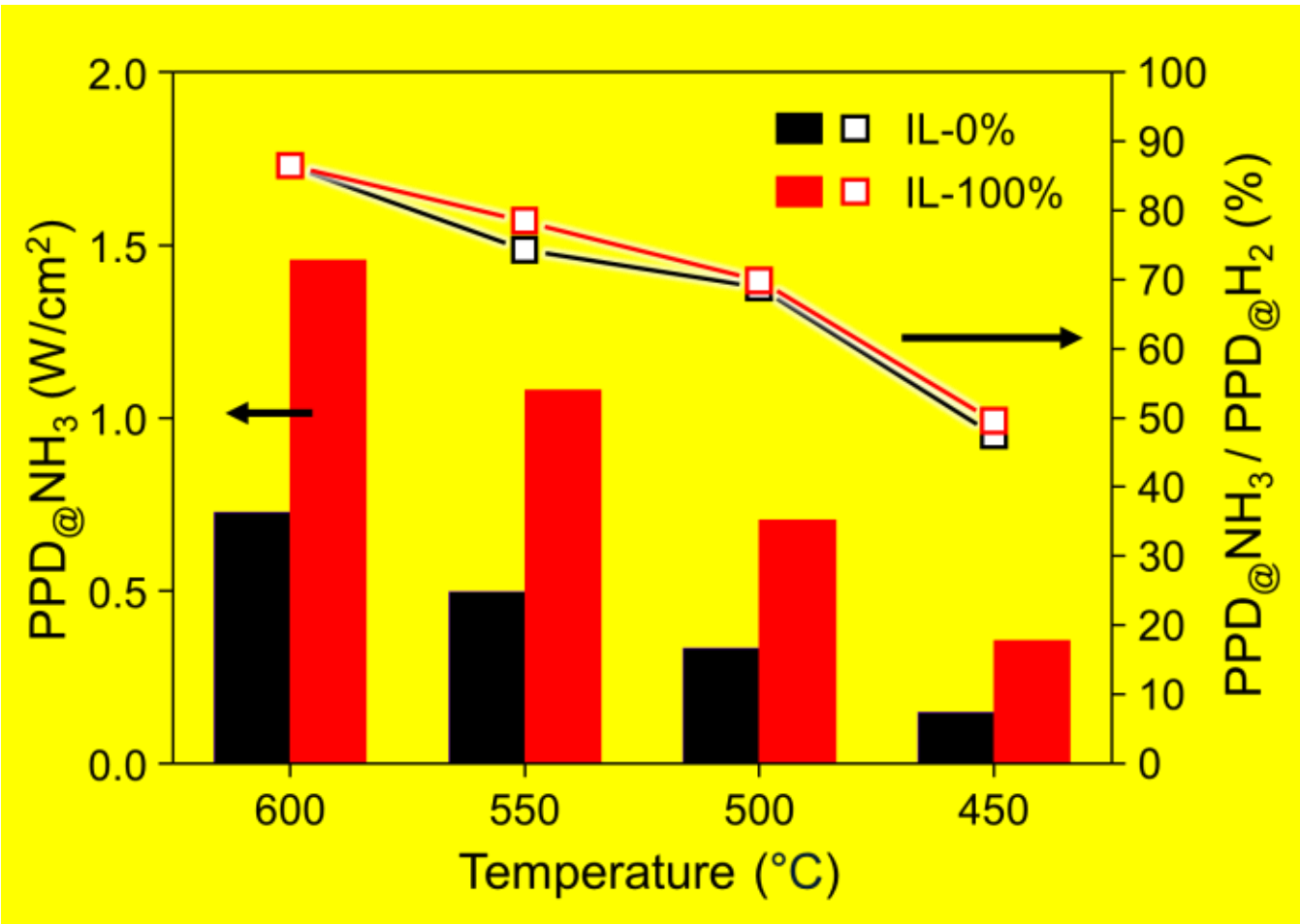

Supplementary Fig. 40. The peak power density at ammonia operation (bar) of IL-0% and IL-100% and the peak power density ratio between ammonia and hydrogen (line) in the temperature range of 600–450 °C.

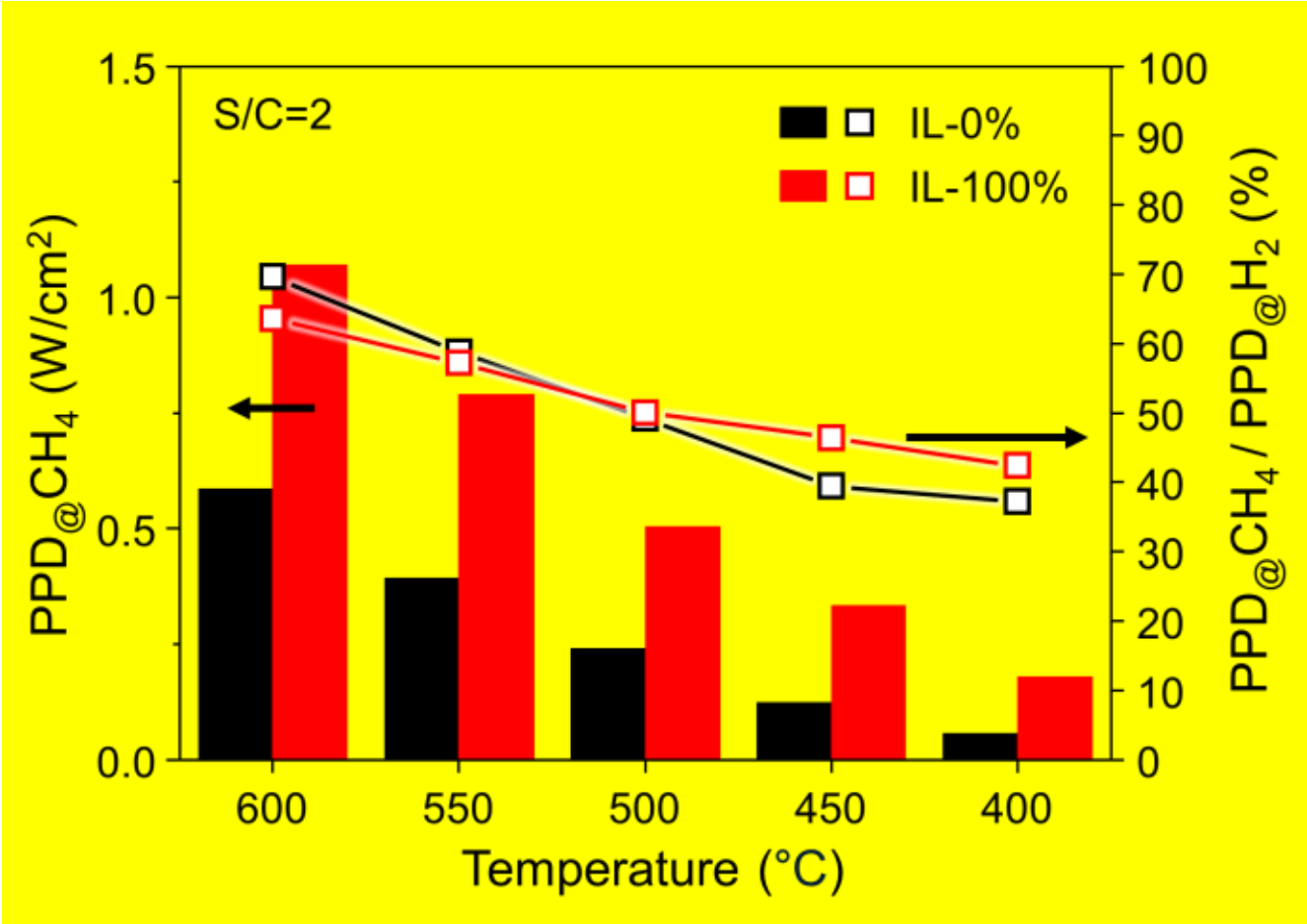

Supplementary Fig. 41. The peak power density at methane operation (bar) of IL-0% and IL-100% and the peak power density ratio between methane and hydrogen (line) in the temperature range of 600–450 °C.

7. The authors are suggested to provide EIS spectra under NH3 and CH4 fuels in the Supplementary Information to further demonstrate the interfacial resilience and mass transport robustness of the ESD-engineered cells in alternative fuel environments.

Thank you for the comment. Since the precursor-based interlayer is introduced to improve the air electrode/electrolyte interface, rather than the fuel electrode/electrolyte interface, its beneficial effect is expected to remain valid regardless of the fuel type. As the reviewer suggested, we have added the EIS spectra measured under NH_3 and CH_4 fuels to Supplementary Information to further demonstrate the interfacial resilience of the ESD-engineered cells under alternative fuel environment.

As shown in the Nyquist plots (Figs. S42 and S43), both the ohmic and polarization resistances of IL-100% are reduced relative to IL-0% in the temperature range of 600–450 °C under both NH_3 and CH_4 operation. The corresponding Bode plots show a pronounced decrease in the low-frequency region (<10 Hz) for IL-100%, indicating enhanced surface charge transfer kinetics at the air electrode, consistent with the behavior observed under H_2 operation. In particular, at 600 °C, the extent of reduction in both ohmic and polarization resistances for IL-100% relative to IL-0% during NH_3 and CH_4 operation is nearly identical to that observed under H_2 operation (Fig. R15). These results confirm that the beneficial effect of the precursor-based interlayer on the air electrode/electrolyte interfacial properties remains effective under alternative fuel conditions.

We have revised the manuscript and the related Supplementary Information as follows:

[line 19, page 15 in the revised manuscript]

In both ammonia and methane fuels, the ratio of peak power densities relative to hydrogen fuel remained similar for IL-0% and IL-100% (Supplementary Figs. 40 and 41), indicating that the observed performance enhancements stem from the same interfacial improvements that enabled the high performance with hydrogen. Consistently, EIS spectra obtained under ammonia and methane operation showed that IL-100% maintained lower ohmic and polarization resistances than IL-0%, with resistances reduction comparable to those observed under hydrogen operation (Supplementary Figs. 42 and 43). This confirms that the beneficial effect of the precursor-based interlayer remains effective under alternative fuel conditions.

These results suggest that further performance improvements could be achieved through ammonia (or methane)-specific strategies to increase their conversion, especially considering the moderate ammonia and methane conversions of ~60% and ~35% at 500 °C, respectively (Supplementary Figs. 44 and 45).

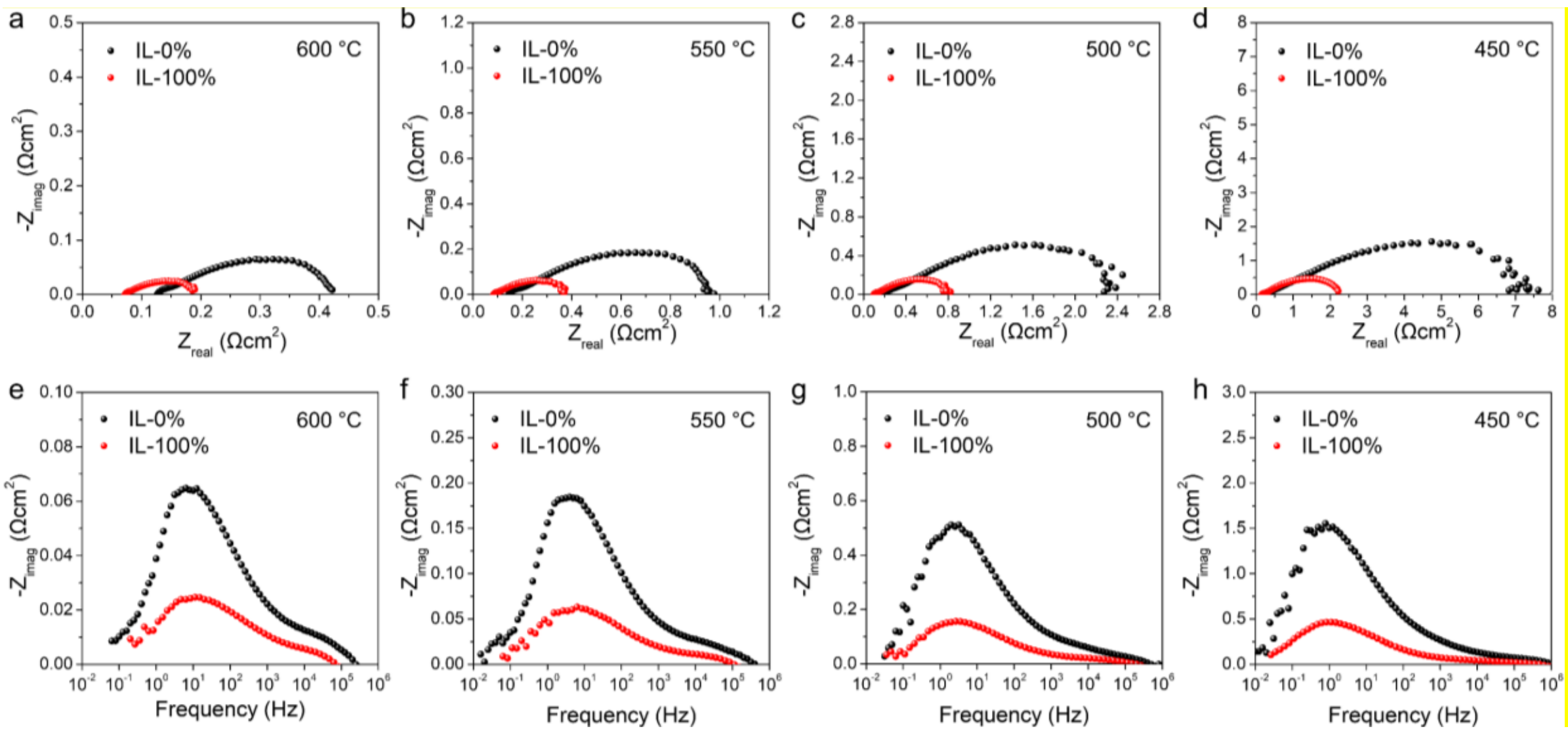

Supplementary Fig. 42. Nyquist plots of IL-0% and IL-100% under NH_3 operation at OCV conditions at (a) 600 °C, (b) 550 °C, (c) 500 °C, and (d) 450 °C. Bode plots of IL-0% and IL-100% under NH_3 operation at OCV conditions at (a) 600 °C, (b) 550 °C, (c) 500 °C, and (d) 450 °C.

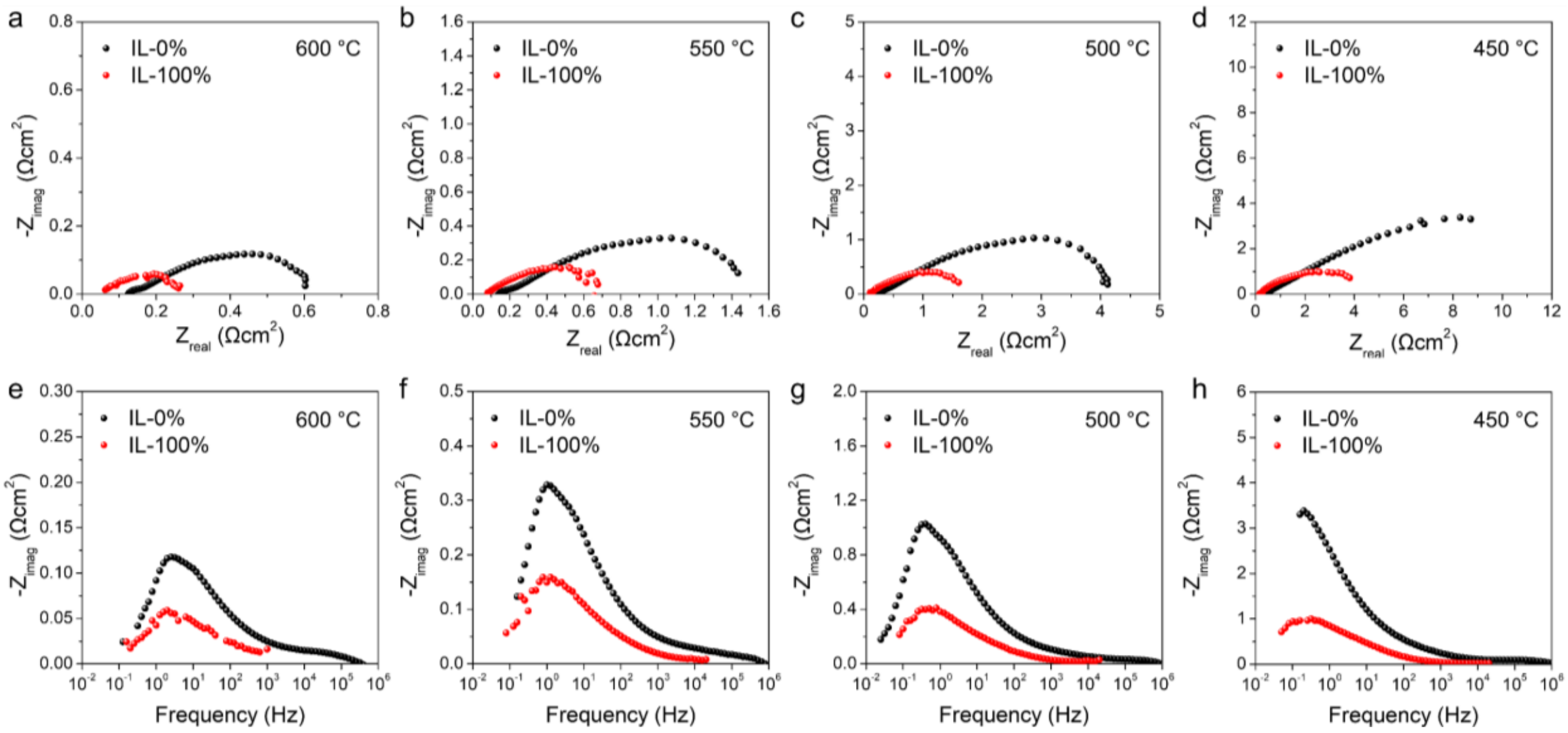

Supplementary Fig. 43. Nyquist plots of IL-0% and IL-100% under CH_4 operation (S/C=2) at OCV conditions at (a) 600 °C, (b) 550 °C, (c) 500 °C, and (d) 450 °C. Bode plots of IL-0% and IL-100% under CH_4 operation (S/C=2) at OCV conditions at (a) 600 °C, (b) 550 °C, (c) 500 °C, and (d) 450 °C.

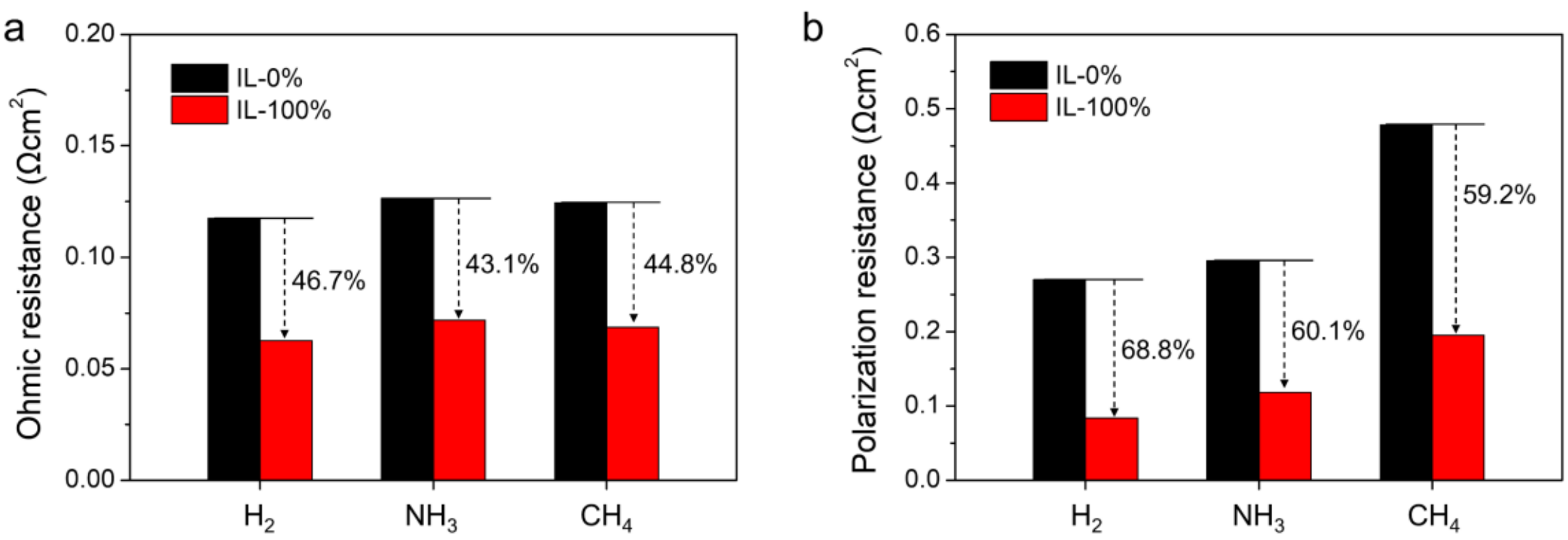

Fig. R15. Comparison of (a) ohmic and (b) polarization resistances for IL-0% and IL-100% under H_2, NH_3, and CH_4 operation at 600 °C.

8. Please provide the cell dimensions and the oxygen-electrode active area in the Methods section, which are important for reproducibility and proper data normalization.

Thank you for the comment. The cell area is 1 cm^2, and the active area of the air electrode is 0.2 cm^2. We have added this information to the Method section as follows:

[line 29, page 16 in the revised manuscript]

After deposition of the air electrode layer, all samples were sintered at 950 °C for 4 h. The cell area was 1 cm^2, and the active area of the air electrode was 0.2 cm^2.

Reviewer #3:

This manuscript reports a precursor-based electrostatic spray deposition (ESD) method to fabricate PBSCF interlayers in protonic ceramic electrochemical cells, with the central claim that improved contact coverage leads to reduced interfacial resistance. Although the general topic is relevant to the energy conversion community, the manuscript suffers from substantive conceptual and methodological deficiencies that critically undermine the validity of its conclusions. In particular, the work relies heavily on model-dependent EIS fitting without independent experimental validation, employs unverified assumptions regarding contact coverage, and fails to disentangle multiple interfacial effects introduced by the interlayer. As a result, the study does not meet the scientific rigor and evidentiary standards required for publication in Nature Communications.

We thank the reviewer for the thorough and critical evaluation. We appreciate the concerns regarding the conceptual and methodological limitations of the original manuscript, particularly the reliance on model-dependent EIS fitting, the unverified assumptions associated with contact coverage, and the lack of clear separation among the multiple interfacial effects introduced by the interlayer. In response, we have substantially revised the manuscript to address these issues by removing unverified assumptions regarding contact coverage, providing independent experimental validation beyond model-dependent EIS fitting, and explicitly disentangling the multiple interfacial effects introduced by the interlayer. As a result, the conclusions are now supported by consistent and complementary experimental evidence rather than by model fitting alone. We believe that these revisions directly address the reviewer's concerns and strengthen the scientific rigor and evidentiary basis of the study. Detailed point-by-point responses are provided below.

1. The central premise for this work, that the current characterization techniques for interfacial properties (including contact coverage) are largely qualitative and insufficient, has already been addressed in the authors' recently published work:

Kim, D., Lee, D. G., Kim, H., Jeong, J. S., & Lee, W. (2026). Quantitative interface engineering framework for high-performance and durable Protonic ceramic electrochemical cells. Advanced Energy Materials, e05277. https://doi.org/10.1002/aenm.202505277

Thank you for this important comment. We acknowledge that our recent work established a quantitative framework for interface engineering in PCECs, including the use of contact coverage as a key descriptor of interfacial properties. While our previous work established a quantitative framework for interface engineering, the present study focuses on its experimental realization and validation through a scalable interlayer design.

More specifically, the present study goes beyond the prior work in two important aspects. First, from a processing perspective, it introduces a precursor-based ESD strategy that enables the formation of a thin, dense, and highly conformal interlayer, providing a practical and scalable route to realize the proposed interface engineering framework. In contrast to the powder-based process in the previous work, which inherently limited the achievable contact coverage to ~50%, the present study extends the contact coverage range to nearly complete contact coverage (~92.6%), enabling validation of the framework in a regime that was not accessible in the previous study and leads to substantially greater interfacial improvement.

Second, from a modeling and validation perspective, the present study extends the framework beyond the treatment of a single powder electrode/electrolyte interface. Specifically, it considers structurally distinct interfacial configurations, including the conventional powder

electrode/electrolyte interface and the precursor-derived interlayer/electrolyte interface, and introduces an expanded equivalent-circuit framework to analyze and compare their interfacial properties in a unified manner. The significance of this extension is not just the inclusion of two interfaces, but the ability to quantitatively evaluate how different interfacial architectures with different contact characteristics contribute to the overall electrochemical behavior. This is particularly important because direct quantification of interfacial properties such as contact coverage, and their systematic correlation with electrochemical performance, remains experimentally challenging and is still rarely demonstrated in a rigorous manner. In this study, we strengthen this aspect through broader structural and electrochemical validation of the contact coverage-resistance relationship.

Therefore, while the conceptual basis was introduced in our previous work, the present work provides its scalable experimental realization, expanded quantitative validation, and implementation across structurally distinct interfaces.

2. The manuscript's primary claim, that contact coverage is the dominant factor governing both ohmic and polarization resistances, is derived exclusively from equivalent circuit fitting of EIS data. However, EIS deconvolution is inherently model-dependent and relies on assumptions. The authors did not provide independent, direct experimental evidence (e.g., 3D x-ray tomography, operando transmission electron microscopy, etc.) to validate the fitted parameters or proposed mechanisms. Without corroborating evidence, the mechanistic interpretation remains speculative.

Thank you for this important comment. We acknowledge that the previous version of the model contained a logical inconsistency in how the key parameter, contact coverage, was incorporated into the analysis, as the reviewer noted.

Even in the original model, independently measured contact coverage values were available from structural and image-based analyses, i.e., model-independent experimental measurements. However, in the previous version of the manuscript, these experimental values were used only to validate the fitted contact coverage values obtained from EIS-based model, rather than being directly incorporated into the fitting procedure. As a result, the subsequent analysis relied on the fitted contact coverage values, which introduced circular reasoning and compromised the validity of the mechanistic interpretation.

To address this issue, we have revised the model accordingly (Table R2). In the original model, all five parameters were determined through fitting. In the revised model, however, three parameters, non-interface resistance ($R_{non\text{-}interface}$) and the contact coverages at each interface (θ_1 and θ_2), are fixed using independently measured experimental values, and only the remaining two parameters, specific contact resistances ($R_{contact1}$ and $R_{contact2}$), are fitted. This revision directly addresses the reviewer's concern, because contact coverage is no longer derived exclusively from equivalent circuit fitting of EIS data, but is instead treated as an independently measured parameter obtained from model-independent experiments.

Table R2. Comparison of fitting parameter between the original and revised models.

	Original model	Revised model
$R_{non\text{-}interface}$ (Ωcm^2)	Fitted	Measured
θ_1 (%)	Fitted	Measured
θ_2 (%)	Fitted	Measured
$R_{contact1}$ (Ωcm^2)	Fitted	Fitted
$R_{contact2}$ (Ωcm^2)	Fitted	Fitted

The detailed modifications to the model are described as follows.

The construction of the equivalent circuit model and the definitions of the parameters ($R_{non-interface}$, $R_{contact1}$, $R_{contact2}$, θ_1, θ_2) are described in detail in Fig. S13.

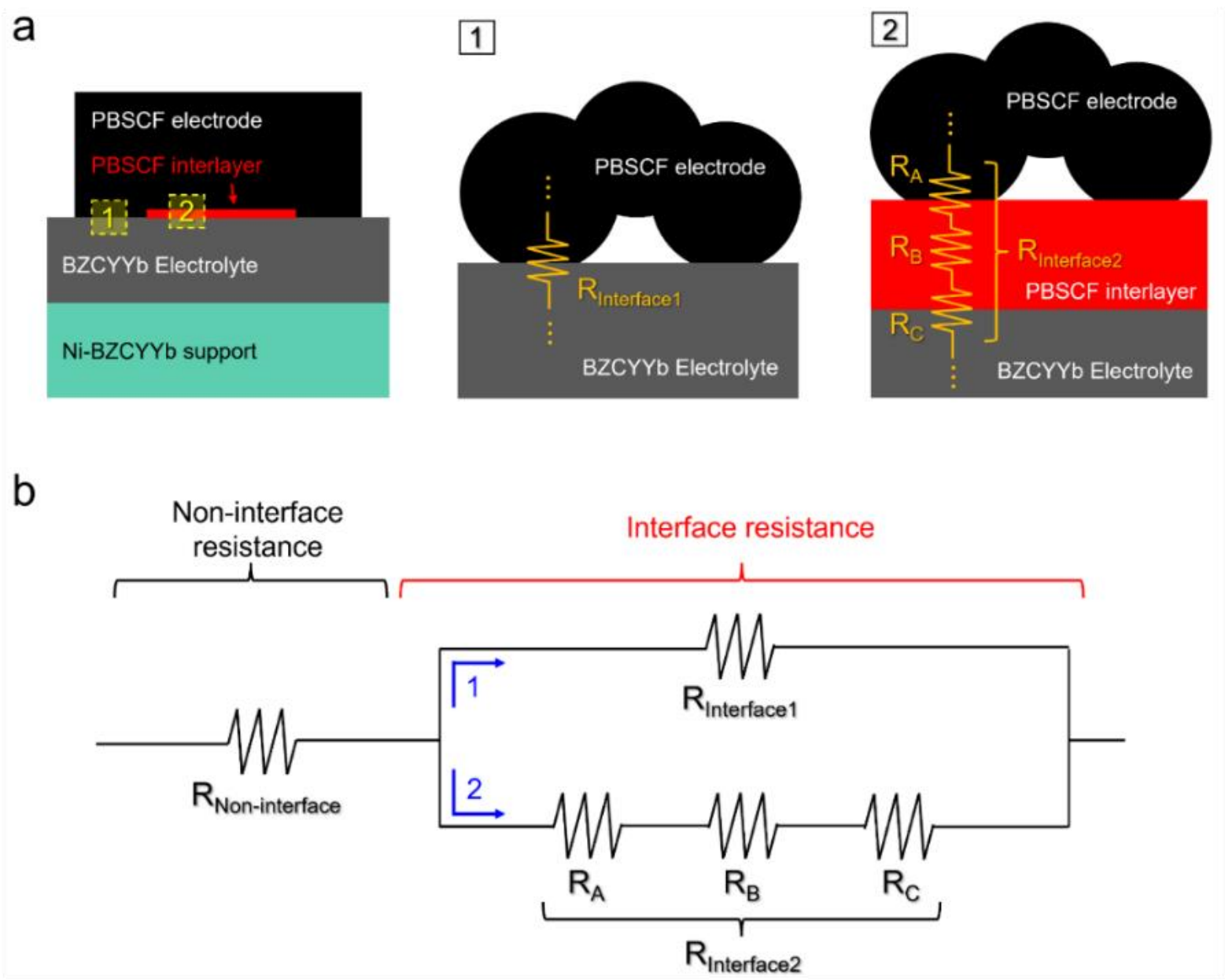

Supplementary Fig. 13. (a) Schematic of the model sample with the corresponding equivalent circuit model for the ohmic resistance at two types of interfaces, with and without the PBSCF interlayer. (b) Equivalent circuit model used to describe the total ohmic resistance.

The resistance terms are defined as follows:

R_{total}: Area-specific total ohmic resistance

$R_{non-interface}$: Area-specific resistance from all components except the PBSCF electrode/BZCYYb electrolyte interface

$R_{interface}$: Area-specific resistance from the PBSCF electrode/BZCYYb electrolyte interface

$R_{interface1}$: $R_{interface}$ without the PBSCF interlayer

$R_{interface2}$: $R_{interface}$ with the PBSCF interlayer

For the interlayer-containing configuration, $R_{interface2}$ is further decomposed into:

R_A: Area-specific resistance from the PBSCF electrode/PBSCF interlayer interface

R_B: Area-specific resistance from ionic and electronic transport within the PBSCF interlayer

R_C: Area-specific resistance from the PBSCF interlayer/BZCYYb electrolyte interface

The corresponding ohmic resistance model can be expressed as:

$$R_{total} = R_{non-interface} + R_{interface}$$

Considering the PBSCF interlayer fraction, *a* (%), defined as the area fraction of the PBSCF interlayer relative to the total electrode area, the interface resistance can be written as:

$$R_{total} = R_{non-interface} + \left(\frac{1 - \frac{a}{100}}{R_{interface1}} + \frac{\frac{a}{100}}{R_{interface2}} \right)^{-1}$$

Here, $R_{interface1}$ and $R_{interface2}$ correspond to the interface resistances without and with the PBSCF interlayer, respectively. For the interlayer-containing configuration, $R_{interface2}$ is composed of three contributions:

$$R_{interface2} = R_A + R_B + R_C$$

where R_A, R_B, and R_C represent the resistances at the PBSCF electrode/PBSCF interlayer

interface, within the PBSCF interlayer, and at the PBSCF interlayer/BZCYYb electrolyte interface, respectively.

However, the ohmic contributions from R_A and R_B are negligible under the present conditions. R_A is expected to be small because the PBSCF electrode and the PBSCF interlayer are co-sintered, promoting strong interfacial integration between identical PBSCF phases. R_B is also minimized by the ultrathin interlayer thickness (~150 nm) and the substantially higher conductivity of PBSCF (~700 S/cm at 500 °C) [R16] compared to BZCYYb (~0.018 S/cm at 500 °C) [R17]. Accordingly, $R_{interface2}$ can be approximated as:

$$R_{interface2} \approx R_C$$

The interface resistance is then expressed in terms of two key parameters: contact resistance and contact coverage, defined as follows:

$R_{contact1}$: Area-specific contact resistance at the PBSCF electrode/BZCYYb electrolyte interface

$R_{contact2}$: Area-specific contact resistance at the PBSCF interlayer/BZCYYb electrolyte interface

θ_1: Contact coverage at the PBSCF electrode/BZCYYb electrolyte interface

θ_2: Contact coverage at the PBSCF interlayer/BZCYYb electrolyte interface

Based on these definitions, the total resistance can be written as:

$$R_{total} = R_{non-interface} + \left(\frac{1 - \frac{a}{100}}{R_{contact1} \cdot \frac{100}{\theta_1}} + \frac{\frac{a}{100}}{R_{contact2} \cdot \frac{100}{\theta_2}} \right)^{-1}$$

[R16] Hung, I.-M. et al. Synthesis and electrochemical performance of $PrBa_{0.5}Sr_{0.5}Co_{1.5}Fe_{0.5}O_{5+\delta}$ double perovskite cathode material for solid oxide fuel cells. *Int. J. Hydrogen Energy* **145**, 371–379 (2025).

[R17] Choi, S. et al. Exceptional power density and stability at intermediate temperatures in protonic ceramic fuel cells. *Nat. Energy* **3**, 202–210 (2018).

In the revised model, three parameters ($R_{non\text{-}interface}$, θ_1, and θ_2) are fixed using values obtained from model-independent experiments, while the remaining two parameters ($R_{contact1}$ and $R_{contact2}$) are fitted. The PBSCF interlayer fraction, *a*, is also defined independently as the area fraction of the PBSCF interlayer relative to the total electrode area. The model-independent experiments to determine each parameter is described as follows.

$R_{non\text{-}interface}$, which primarily originates from ionic conduction through the electrolyte, was independently determined from electrolyte thickness-dependent experiments (Fig. S14). As described in the main manuscript, the calculated electrolyte conductivity (~0.015 S/cm) was in good agreement with the reported values for BZCYYb (~0.01–0.02 S/cm, at 500 °C), confirming the reliability of the measurements. More specifically, linear fitting of the area-specific ohmic resistance as a function of electrolyte thickness yielded the electrolyte conductivity from the inverse slope, and the corresponding activation energy (0.24 eV) was also consistent with literature values (0.23-0.33 eV). Using the measured electrolyte thickness of ~10 μm), the electrolyte resistance was calculated to be 0.0652, 0.0483, 0.0407, and 0.0367 Ωcm^2 at 500, 550, 600, and 650 °C, respectively. These values were then fixed as $R_{non\text{-}interface}$ in the revised model.

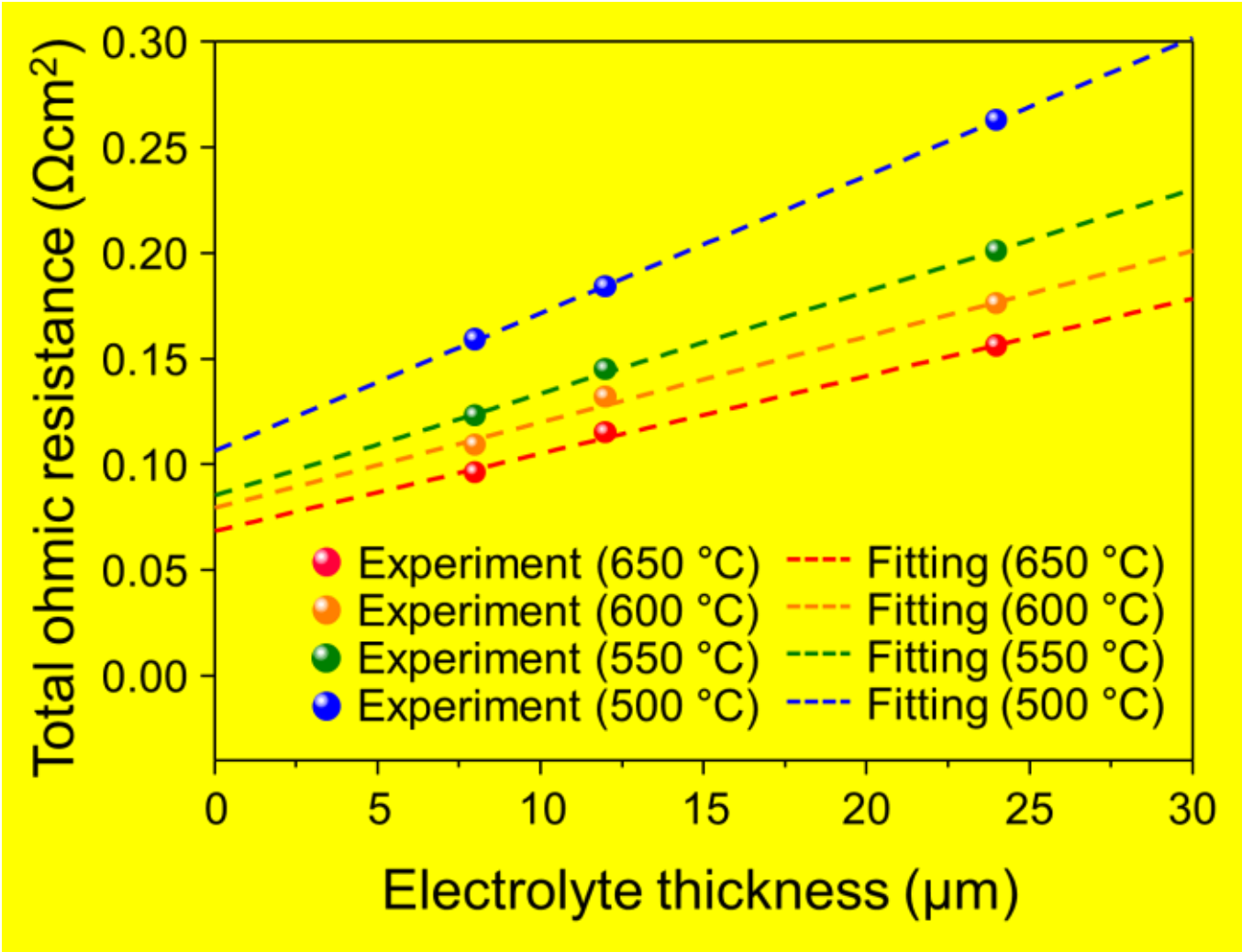

Supplementary Fig. 14. Total ohmic resistance as a function of electrolyte thickness. The electrolyte thickness was controlled by varying the number of spin-coating processes.

Supplementary Table 2. Comparison of the electrolyte conductivity and corresponding activation energy with reported values.

	Conductivity (S/cm)				Activation energy (eV)
	650 °C	600 °C	550 °C	500 °C	
This work (Electrolyte-thickness dependent experiment)	0.027	0.025	0.021	0.015	0.24
Previous work (R18)	0.033	0.027	0.024	0.018	0.23
Previous work (R19)	0.018	0.014	0.011	0.008	0.33
Previous work (R20)	0.014	0.012	0.010	0.007	0.27

[R18] Choi, S. et al. Exceptional power density and stability at intermediate temperatures in protonic ceramic fuel cells. *Nat. Energy* **3**, 202–210 (2018).

[R19] Kane, N. et al. Durable and high-performance thin-film BHYb-coated BZCYYb bilayer

electrolytes for proton-conducting reversible solid oxide cells. *ACS Appl. Mater. Interfaces* **15**, 32395–32403 (2023).

[R20] Luo, Z. et al. Harnessing high-throughput computational methods to accelerate the discovery of optimal proton conductors for high-performance and durable protonic ceramic electrochemical cells. *Adv. Mater.* **36**, 2311159 (2024).

θ_1 and θ_2, representing the contact coverages at the PBSCF electrode/BZCYYb electrolyte interface and the PBSCF interlayer/BZCYYb electrolyte interface, respectively, were independently determined by model-independent structural analyses. For θ_1, the electrode was removed from the electrolyte surface, and the fraction of contact traces remaining on the electrolyte surface was quantified by image analysis. To ensure reliability, the analysis was performed on a total of four SEM images (Fig. S15 and Table S3), yielding an experimental θ_1 value of 26.7 ± 4.1%. This value is in good agreement with previously reported values obtained using powder-based electrode deposition methods (17.9-41.1%) [R21-R24]. For θ_2, the value was measured by image analysis of SEM images of the precursor-based interlayer surface (Fig. S16 and Table S4), yielding 92.6 ± 0.6%. Thess values were then fixed as θ_1 and θ_2 in the revised model.

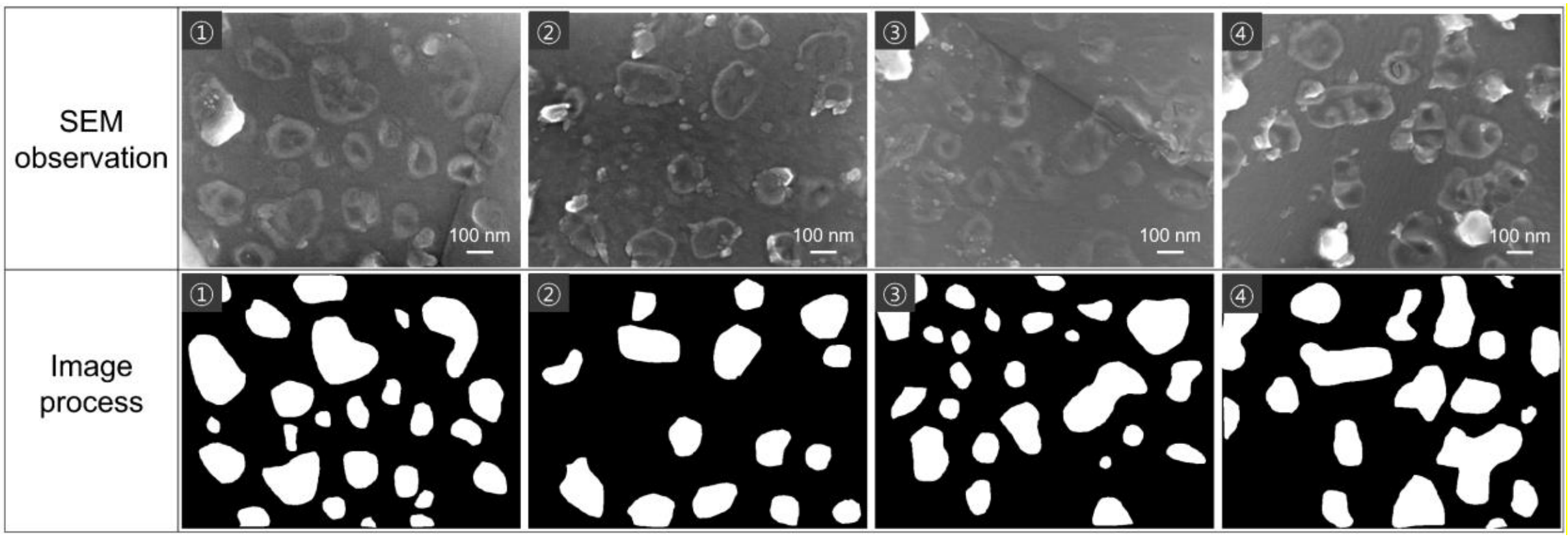

Supplementary Fig. 15. Image processing of the BZCYYb electrolyte surface after detaching the PBSCF electrode particles of IL-0%. Using the image processing software (Image J), the contact areas (crater-like traces) were identified as white regions. Then, the contact coverage was calculated using the equation shown below.

$$Contact\ coverage\ (\%) = \frac{number\ of\ pixels\ for\ contact\ area}{number\ of\ pixels\ for\ electrolyte\ surface} \times 100$$

Supplementary Table. 3. Measured contact coverage of IL-0% derived from the analysis in Supplementary Fig. 15.

Location	1	2	3	4
Number of pixels for electrolyte surface	428652	428652	428652	428652
Number of pixels for contact area	120691	78773	98440	125119
Contact coverage (%)	28.156	18.377	22.965	29.189

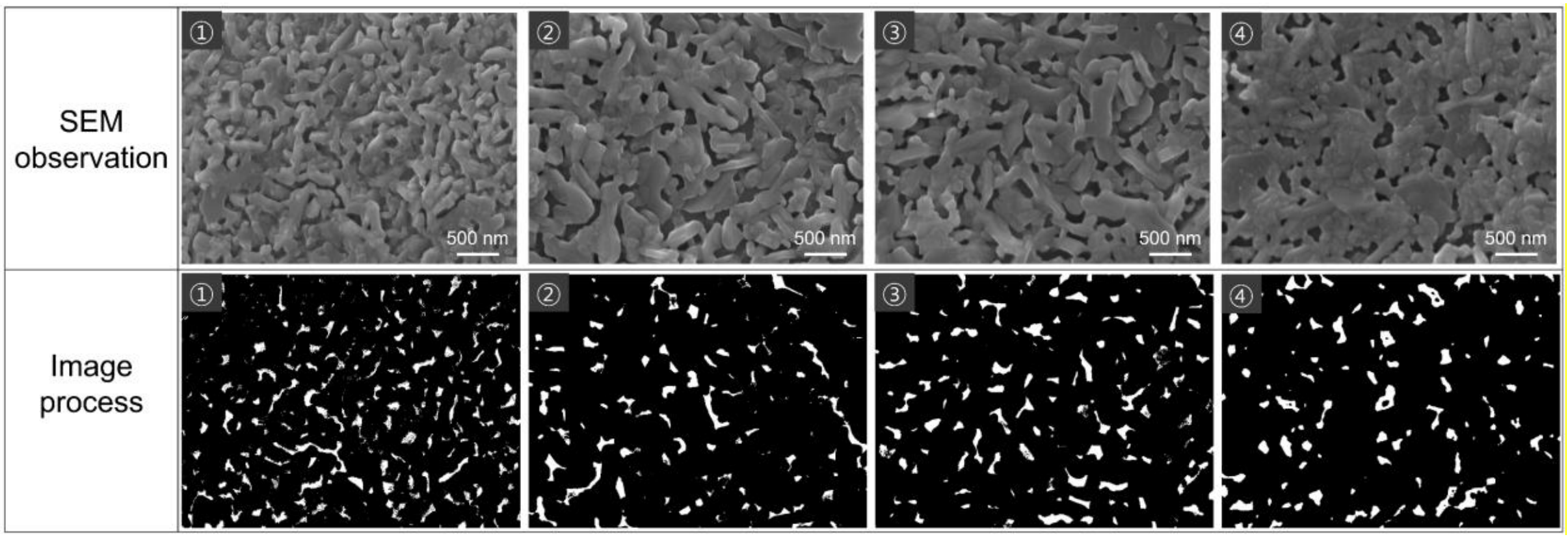

Supplementary Fig. 16. Image processing of the PBSCF interlayer surface. Using the image processing software (Image J), the contact coverage was calculated using the equation shown below.

$$Contact\ coverage\ (\%) = \frac{number\ of\ pixels\ for\ interlayer\ surface}{number\ of\ pixels\ for\ entire\ image} \times 100$$

Supplementary Table. 4. Measured contact coverage of IL-100% derived from the analysis in Supplementary Fig. 16.

Location	1	2	3	4
Number of pixels for entire image	418691	418691	418691	418691
Number of pixels for interlayer surface	384928	391614	386021	388160
Contact coverage (%)	91.936	93.533	92.197	92.708

[R21] Yoo, H. S. et al. Interface engineering to improve electrochemical performance of intermediate-temperature solid oxide fuel cells. *Appl. Surf. Sci.* **639**, 158188 (2023).

[R22] Choi, M. et al. Interface engineering to operate reversible protonic ceramic electrochemical cells below 500 °C. *Adv. Energy Mater.* **15**, 2400124 (2025).

[R23] Kim, D. et al. Quantitative interface engineering framework for high-performance and

durable protonic ceramic electrochemical cells. *Adv. Energy Mater.* e05277 (2026).

[R24] Im, S. et al. $PrBa_{0.5}Sr_{0.5}Co_{1.5}Fe_{0.5}O_{5+\delta}$ composite cathode in protonic ceramic fuel cells. *J. Korean Ceram. Soc.* **58**, 351–358 (2021).

After fixing these three parameters using independently measured values, fitting was conducted only for the remaining two parameters, the specific contact resistances ($R_{contact1}$ and $R_{contact2}$). This reduced the number of free fitting parameters from five to two, thereby substantially decreasing the degree of model dependence. The revised model reproduced the experimental data with an R^2 value of 0.997 ± 0.001 (Fig. R16). The fitted values of $R_{contact1}$ and $R_{contact2}$ were comparable to those obtained from the original model (Tables R3 and R4), and the overall relationship between contact coverage and ohmic resistance was preserved. These results indicate that correcting the previous logical inconsistency does not alter the central conclusion that increased contact coverage is the primary factor reducing interfacial resistance.

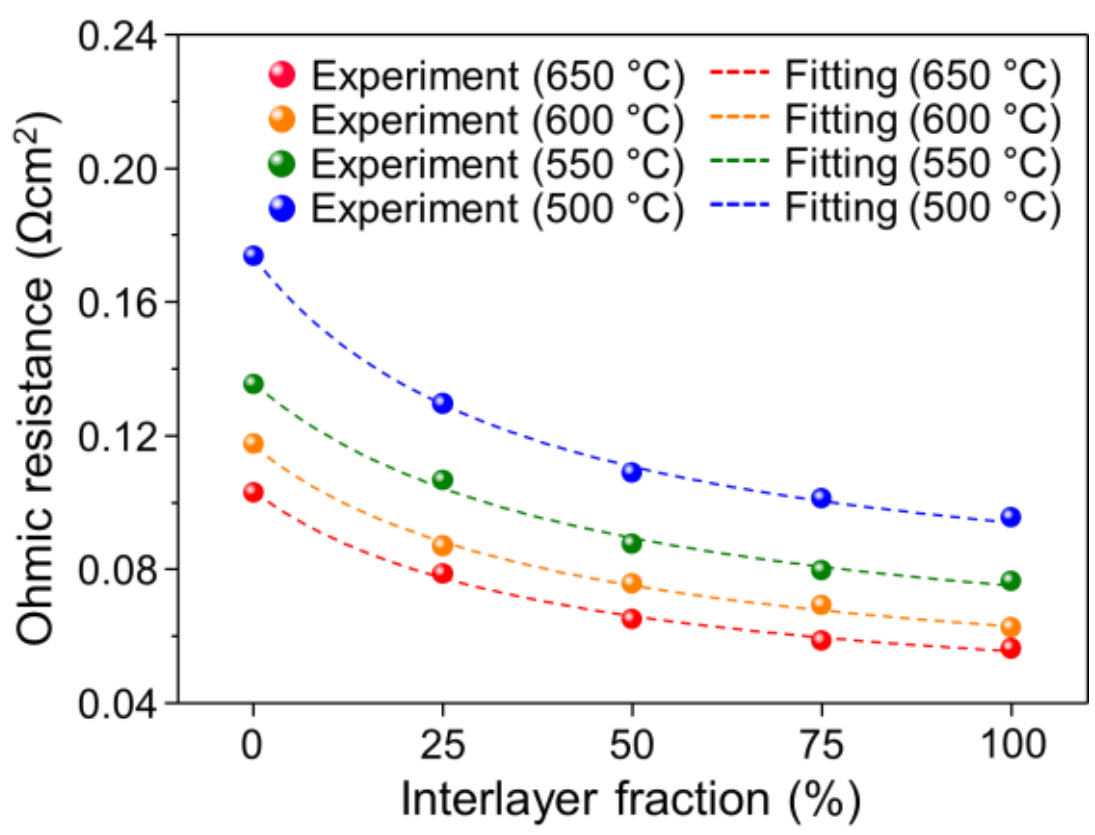

Fig. R16. Total ohmic resistance as a function of interlayer fraction with corresponding fitting results in the temperature range of 650–500 °C.

Table R3. Parameters obtained from the original model.

	Temperature (°C)			
	650	600	550	500
$R_{non\text{-}interface}$ (Ωcm^2) (Fitted)	0.0351	0.0439	0.0458	0.0691
θ_1 (%) (Fitted)	27.41	28.39	28.54	25.09
θ_2 (%) (Fitted)	91.12	91.99	94.26	92.29
$R_{contact1}$ (Ωcm^2) (Fitted)	0.0187	0.0209	0.0257	0.0263
$R_{contact2}$ (Ωcm^2) (Fitted)	0.0182	0.0184	0.0235	0.0239

Table R4. Parameters obtained from the modified model.

	Temperature (°C)			
	650	600	550	500
$R_{non\text{-}interface}$ (Ωcm^2) (Measured)	0.0367	0.0407	0.0483	0.0652
θ_1 (%) (Measured)	26.7			
θ_2 (%) (Measured)	92.6			
$R_{contact1}$ (Ωcm^2) (Fitted)	0.0178	0.0204	0.0234	0.0290
$R_{contact2}$ (Ωcm^2) (Fitted)	0.0174	0.0207	0.0250	0.0268

We have revised the manuscript and supporting information as follows:

[line 4, page 7 in the revised manuscript]

To further evaluate the specific contributions of the interlayer to the interface-related components in ohmic resistance, we developed an equivalent circuit model comprising two

components: non-interface resistance and interface resistance (Supplementary Fig. 13).[20] The non-interface resistance ($R_{non\text{-}interface}$), originated from the electrolyte, electrodes, and measurement setup, remained constant for all samples due to the identical cell configurations. $R_{non\text{-}interface}$, which primarily originates from ionic conduction through the electrolyte, was independently determined from electrolyte thickness-dependent experiments (Supplementary Fig. 14 and Supplementary Table 2). The calculated electrolyte conductivity (~0.015 S/cm at 500 °C) and activation energy (~0.24 eV) agreed well with reported values for BZCYYb (~0.01–0.02 S/cm at 500 °C and ~0.23–0.33 eV)[6,27,28], confirming the reliability of the measurements. In contrast, the interface resistance varied with the interlayer fraction and contact properties, including contact coverage and specific contact resistance. The contact coverages of the electrode/electrolyte interface in IL-0% and the interlayer/electrolyte interface in IL-100%, denoted as θ_1 and θ_2, respectively, were independently measured by structural analysis of the electrolyte surface after detaching the electrode and of the interlayer surface (Supplementary Figs. 15-16 and Supplementary Tables 3-4). In particular, the interlayer/electrolyte interface exhibited a significantly higher contact coverage of 92.6 ± 0.6% (θ_2), which is ~3.5 times higher than that of the electrode/electrolyte interface (26.7 ± 4.1%, θ_1), consistent with previously reported values.[11,20] For the intermediate samples (IL-25%, IL-50%, and IL-75%), which were physically constructed as composite interfaces with defined area fractions of two configurations, the overall contact coverage was geometrically defined from θ_1, θ_2, and the interlayer fraction (Supplementary Fig. 17). After fixing three parameters ($R_{non\text{-}interface}$, θ_1, and θ_2), fitting was conducted for the remaining two parameters, the specific contact resistances ($R_{contact1}$ and $R_{contact2}$). The model accurately reproduced the measured ohmic resistances for all interlayer fractions in the temperature range of 650–500 °C, with an R^2 value of 0.997 ± 0.001 as shown in Fig. 2(d). The detailed fitting results are listed in Supplementary Table 5.

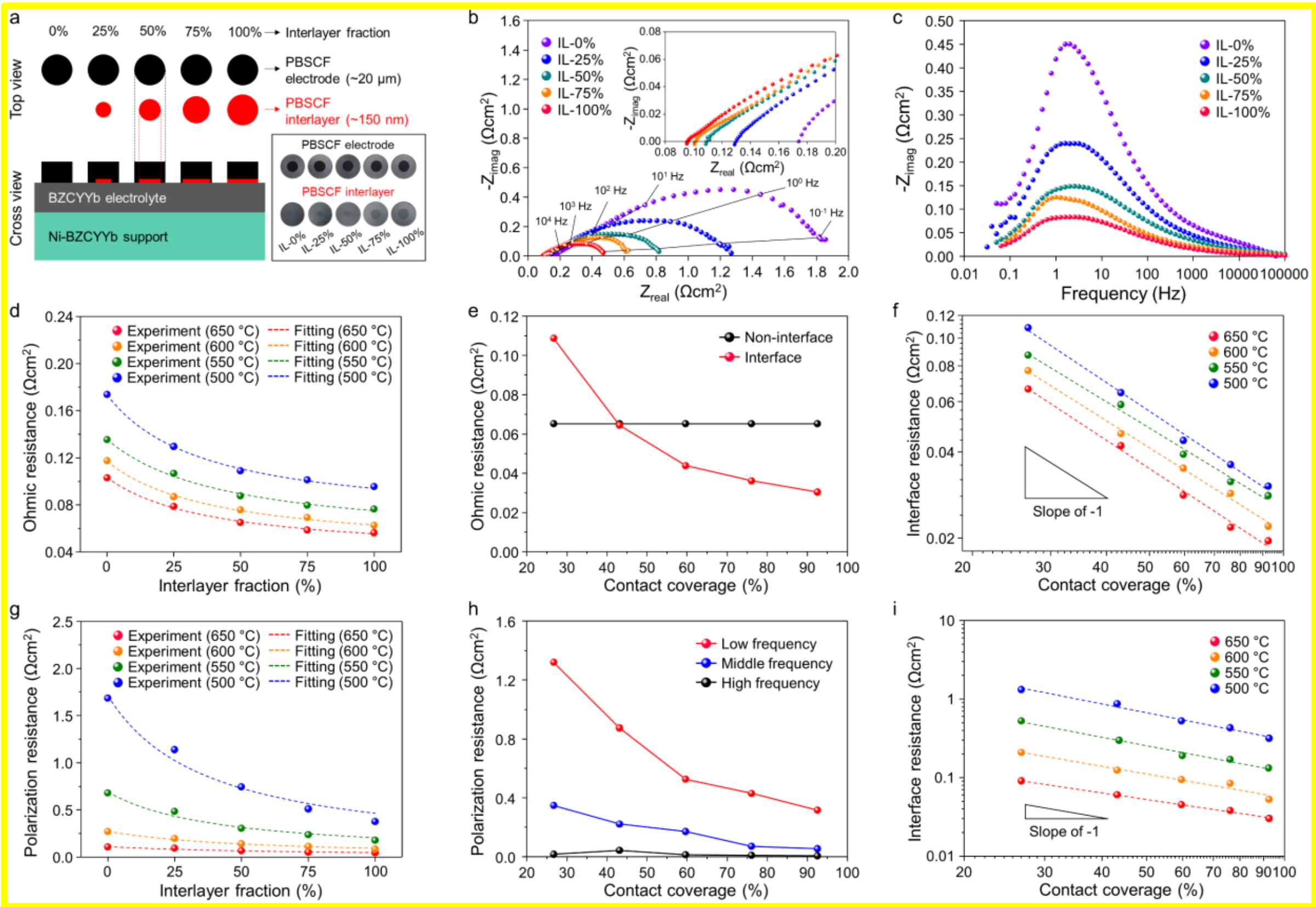

Fig. 2: Impedance model analysis: Correlation between the contact coverage and the interface resistances. (a) Schematic illustration of the model sample configuration with optical images. (b) Nyquist plots and (c) Bode plots of the model samples at 500 °C under OCV conditions. (d) Total ohmic resistance as a function of the interlayer fraction with corresponding fitting results in the temperature range of 650–500 °C. (e) Decoupled ohmic resistance of the interface and non-interface resistances at 500 °C as a function of the contact coverage. (f) Correlation between the interface component of the ohmic resistance and contact coverage in double logarithmic coordinates. (g) Total polarization resistance as a function of the interlayer fraction with corresponding fitting results in the temperature range of 650–500 °C. (h) Decoupled polarization resistance of the low-, middle-, and high-frequency resistances at 500 °C as a function of the contact coverage. (i) Correlation between the interface component

of the polarization resistance and contact coverage in double logarithmic coordinates.

Supplementary Table 5. The model fitting results at 500–650 °C.

	Temperature (°C)			
	650	600	550	500
$R_{contact1}$ (Ωcm^2)	0.0178	0.0204	0.0234	0.0290
$R_{contact2}$ (Ωcm^2)	0.0174	0.0207	0.0250	0.0268

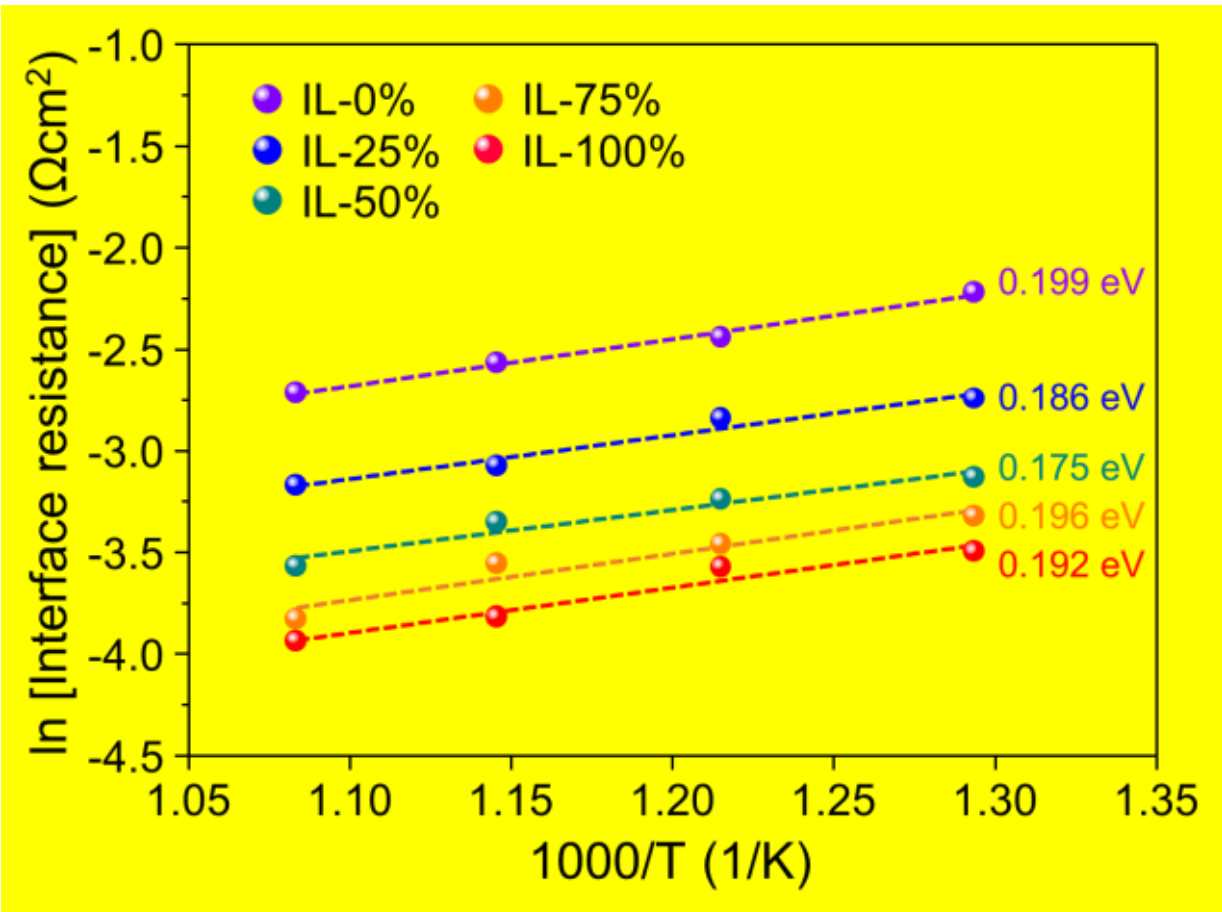

Supplementary Fig. 18. Arrhenius plots of the interfacial ohmic resistances for IL-0%, IL-25%, IL-50%, IL-75%, and IL-100% in the temperature range of 650–500 °C.

3. The authors assume a linear relationship between interlayer fraction and contact coverage. For the intermediate samples (25%, 50%, 75%), the contact coverage values are not measured but instead assigned through a linear interpolation (Fig. S13, Table S3). The impedance model is then used to validate this assumed linearity, which introduces circular reasoning. The model depends on the assumed coverage values, and the resulting fit is subsequently used to support the assumption itself. This is concerning.

Thank you for the important comment. We agree that using fitted or assumed contact coverage values as model inputs would introduce circular reasoning, and we acknowledge that the original model contained such a logical inconsistency, as discussed in our response to Comment 2. To address this issue, we have revised the model by directly incorporating experimentally measured contact coverage values, thereby eliminating the logical inconsistency. We would also like to clarify that the relationship between interlayer fraction and contact coverage in the intermediate samples is not an arbitrary linear interpolation. In the present study, the intermediate samples (IL-25%, IL-50%, and IL-75%) were physically constructed as composite interfaces consisting of defined area fractions of two distinct configurations (Fig. R17): the powder-based electrode/electrolyte interface (IL-0%) and the precursor-based interlayer/electrolyte interface (IL-100%). The contact coverage values for these two distinct configurations were independently measured through model-independent structural analyses, rather than obtained from fitting. Accordingly, the overall contact coverage of each intermediate sample is geometrically defined as the area-weighted combination of the experimentally measured two distinct configurations.

$$\theta = \left(1 - \frac{a}{100}\right) \times \theta_1 + \frac{a}{100} \times \theta_2$$

where a is the interlayer fraction, θ_1 and θ_2 are the experimentally measured contact coverage values for IL-0% and IL-100%, respectively. Therefore, the contact coverage values used in revised model are not derived from fitting, but are determined from the known sample geometry and independently measured quantity. In this regard, the circular reasoning identified by the reviewer has been removed.

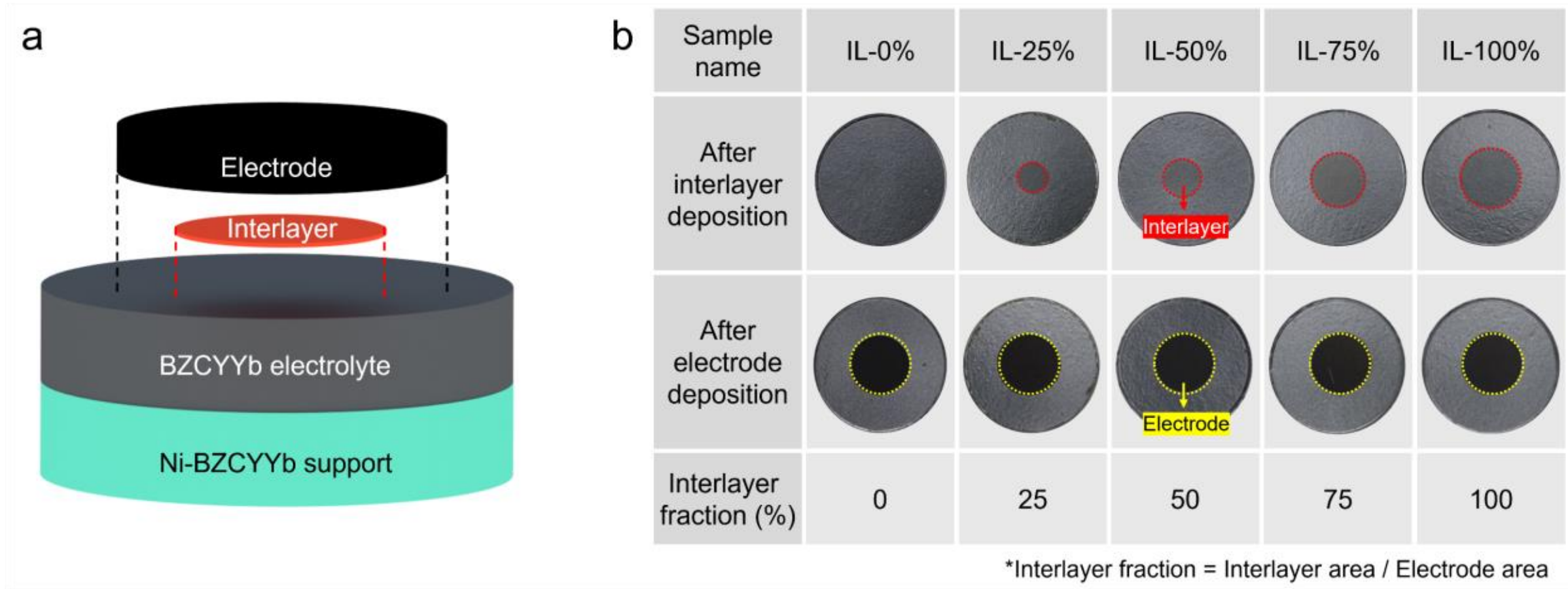

Fig. R17. (a) Schematic illustration of the model sample configuration and (b) the corresponding optical images of the samples with different interlayer fractions.

Accordingly, we have revised the manuscript and Supplementary Information as follows:

[line 17, page 7 in the revised manuscript]

The contact coverages of the electrode/electrolyte interface in IL-0% and the interlayer/electrolyte interface in IL-100%, denoted as θ_1 and θ_2, respectively, were independently measured by structural analysis of the electrolyte surface after detaching the electrode and of the interlayer surface (Supplementary Figs. 15-16 and Supplementary Tables 3-4). In particular, the interlayer/electrolyte interface exhibited a significantly higher contact coverage of 92.6 ± 0.6% (θ_2), which is ~3.5 times higher than that of the electrode/electrolyte interface (26.7 ± 4.1%, θ_1), consistent with previously reported values.[11,20] For the intermediate samples (IL-25%, IL-50%, and IL-75%), which were physically constructed as composite interfaces with defined area fractions of two configurations, the overall contact coverage was

geometrically defined from θ_1, θ_2, and the interlayer fraction (Supplementary Fig. 17).

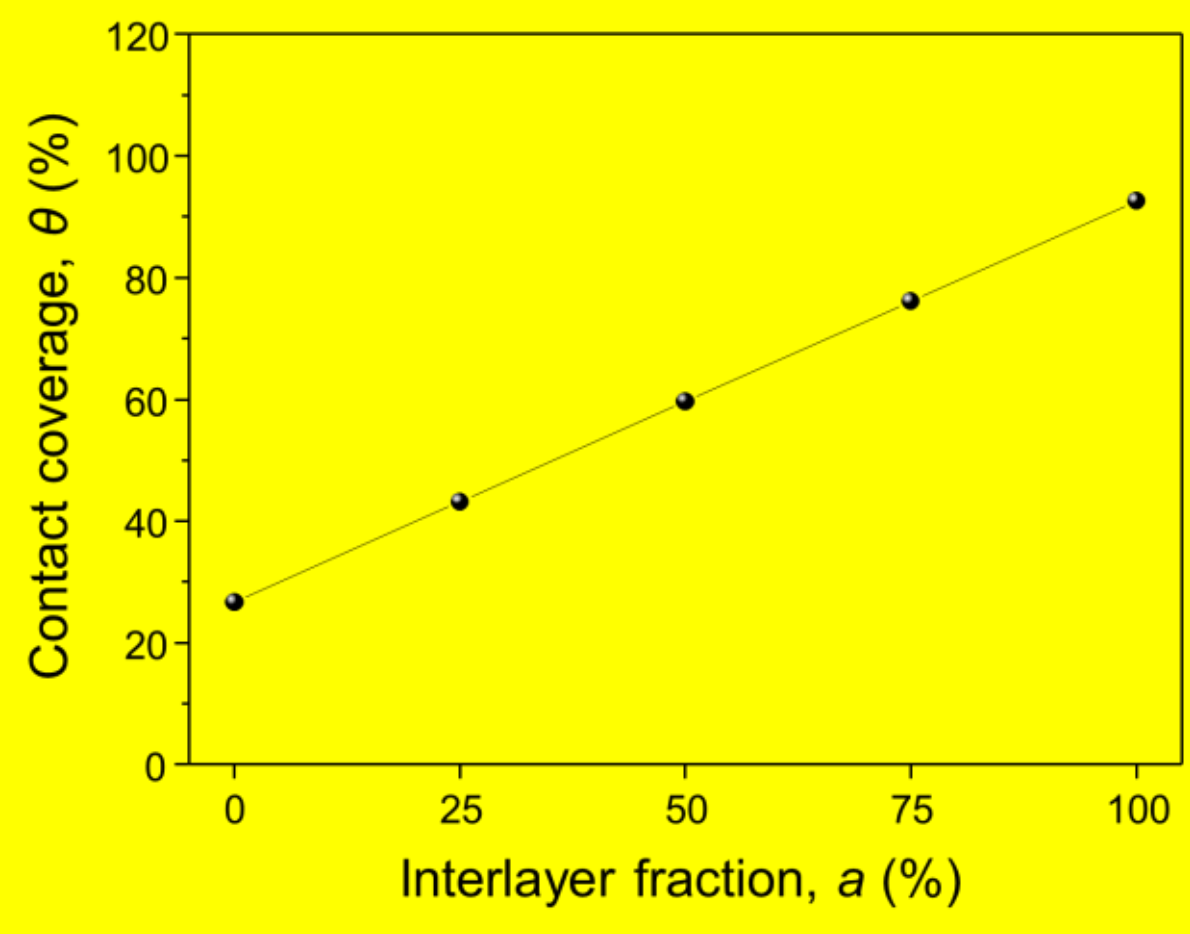

Supplementary Fig. 17. The linear correlation between the interlayer fraction and the contact coverage, determined from the area-weighted combination of the experimentally measured contact coverages of IL-0% and IL-100%.

4. Scientifically speaking, the interlayer modifies multiple interfacial properties simultaneously, including chemistry, roughness, defect concentration, wetting behavior, and thermal mechanical compatibility. The work attributes all improvements solely to increased contact coverage, but no experiments are designed to isolate these variables. As a result, the causal link between coverage and performance remains uncertain.

Thank you for the important comment. We agree that electrochemical performance at the electrode/electrolyte interface can be influenced by multiple interfacial properties, including

chemistry, roughness, microstructure, defect concentration, and physical contact characteristics. Accordingly, the present study does not assume that contact coverage is the only variable, but instead designs to systematically evaluate its role while minimizing or constraining other contributing factors.

To isolate the effect of material chemistry, the interlayer was fabricated using PBSCF with same composition as the electrode. TEM point EDS analysis (Fig. S10, Table S1) and XPS analysis (Table R5) confirm identical atomic ratios between the PBSCF electrode and PBSCF interlayer, thereby excluding differences in chemical composition as a dominant factor.

In addition, AFM analysis (Fig. S9) shows that the interlayer surface exhibits a roughness of 48.6 ± 9.5 nm and a specific surface area of 1.066 ± 0.019 $\mu m^2/\mu m^2$, comparable to those of the pristine electrolyte surface, 46.7 ± 16.8 nm and 1.010 ± 0.010 $\mu m^2/\mu m^2$, respectively, indicating a nearly 2D morphology. This suggests that variations in surface roughness and associated surface area are minimal.

Although the powder-based PBSCF electrode and the precursor-based PBSCF interlayer may exhibit differences in crystallite size and the associated defect chemistry due to their distinct fabrication methods, these differences are limited under the present conditions. XRD analysis (Fig. S11) shows that the crystallite sizes of the electrode (25.1 ± 2.7 nm) and the interlayer (21.1 ± 1.9 nm) are comparable. Furthermore, XPS O 1s analysis (Fig. S25 and Table S7) indicates similar oxygen vacancy concentrations for the electrode (relative area of defect-related peaks, 0.16) and the interlayer (relative area of defect-related peaks, 0.18). These results indicated that differences in microstructure and defect concentration exist but are limited, and therefore are unlikely to be the dominant factors governing the observed performance changes.

This interpretation is further supported by nearly unchanged activation energies for both ohmic

and polarization resistances. The Arrhenius plots of the ohmic and polarization resistances for the model samples (Fig. R18) exhibit parallel trends with similar activation energies (0.190 ± 0.009 and 1.04 ± 0.06 eV, respectively), indicating that the underlying reaction mechanisms remain unchanged. Since variations in chemistry or defect structure typically manifest as changes in activation energy, the absence of such changes suggests that the observed improvements do not originate from intrinsic kinetic improvement. Furthermore, the contact coverage-based model shows strong agreement with experimental data, with high R^2 values (0.997 ± 0.001 for ohmic resistance and 0.974 ± 0.015 for polarization resistance) (Fig. R19). The clear linear scaling behavior observed in the power-law relationship (Fig. R20) further supports that the variation in both ohmic and polarization resistances is correlated with contact coverage.

Therefore, while multiple interfacial factors may exist, the combination of identical chemical composition, comparable surface morphology, similar microstructure, similar defect concentration, and unchanged activation energies provides consistent evidence that the observed performance improvement is governed primarily by structural modification, that is the increase in interfacial contact coverage, rather than by changes in intrinsic interfacial properties.

We acknowledge that this aspect was not sufficiently discussed in the originally submitted manuscript. Accordingly, we have revised the manuscript and Supplementary Information as follows:

[line 10, page 9 in the revised manuscript]

This conclusion was further supported by the strong linear correlation with an R^2 of ~0.978 observed in the normalized Arrhenius plots of all interface resistance values (Supplementary Fig. 24).[12] Importantly, this interpretation does not imply that the contact coverage is the only interfacial variable governing electrochemical performance. Therefore, compositionally identical PBSCF was used for the electrode and interlayer to provide a controlled model system that minimizes variations in other material-related factors, including chemistry, microstructure, and defect concentration. This design is supported by TEM point EDS analysis confirming similar chemical composition (Supplementary Fig. 10 and Supplementary Table 1), AFM showing comparable surface roughness (Supplementary Fig. 9), XRD indicating similar crystallite sizes (Supplementary Fig. 11), XPS O 1s analysis showing comparable oxygen vacancy-related features (Supplementary Fig. 25 and Supplementary Table 7), and the nearly unchanged activation energies (Supplementary Figs. 18 and 23).

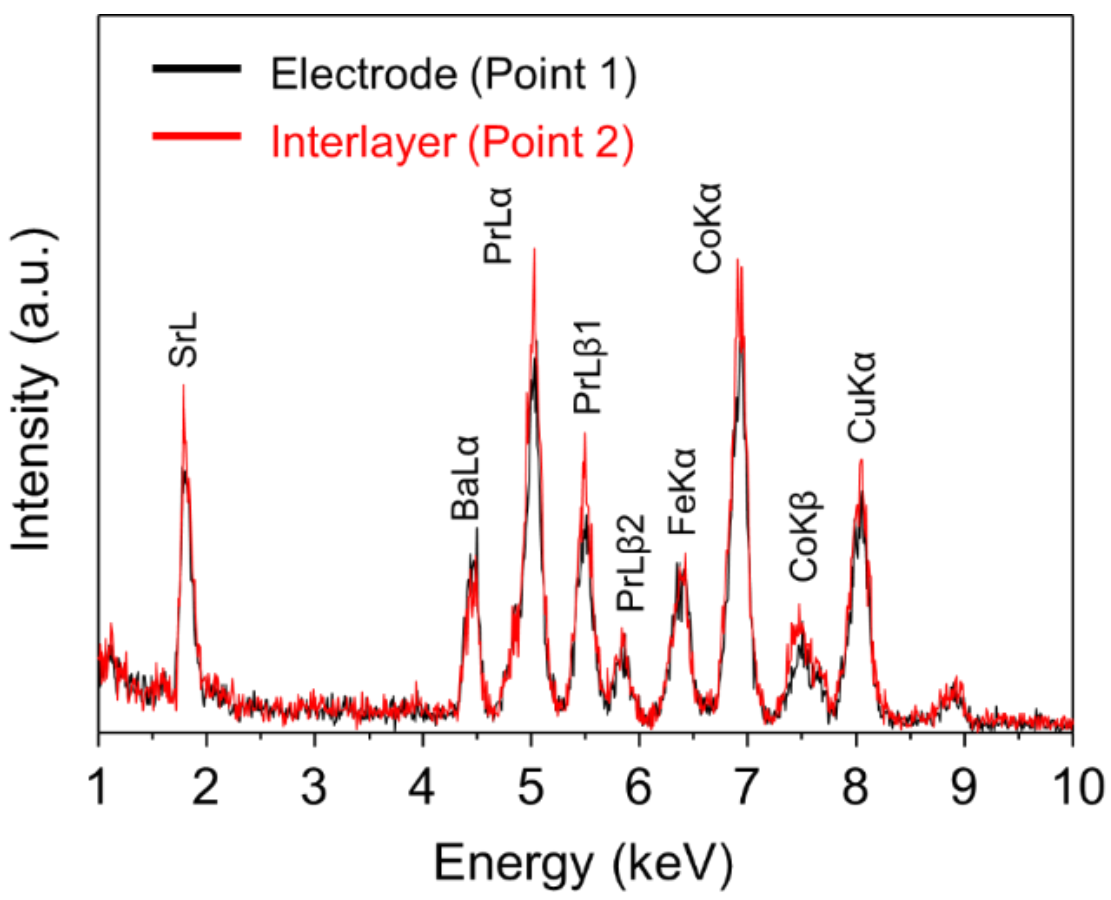

Supplementary Fig. 10. EDS spectra acquired at the powder electrode (Point 1) and the precursor-based interlayer (Point 2).

Supplementary Table 1. Atomic ratios acquired at the powder electrode (Point 1) and the

precursor-based interlayer (Point 2) from EDS spectra in Supplementary Fig. 10.

	Atomic ratio over (Pr+Ba+Sr+Co+Fe)				
	Pr	Ba	Sr	Co	Fe
Electrode (Point 1)	0.220	0.082	0.180	0.434	0.083
Interlayer (Point 2)	0.225	0.084	0.171	0.445	0.075

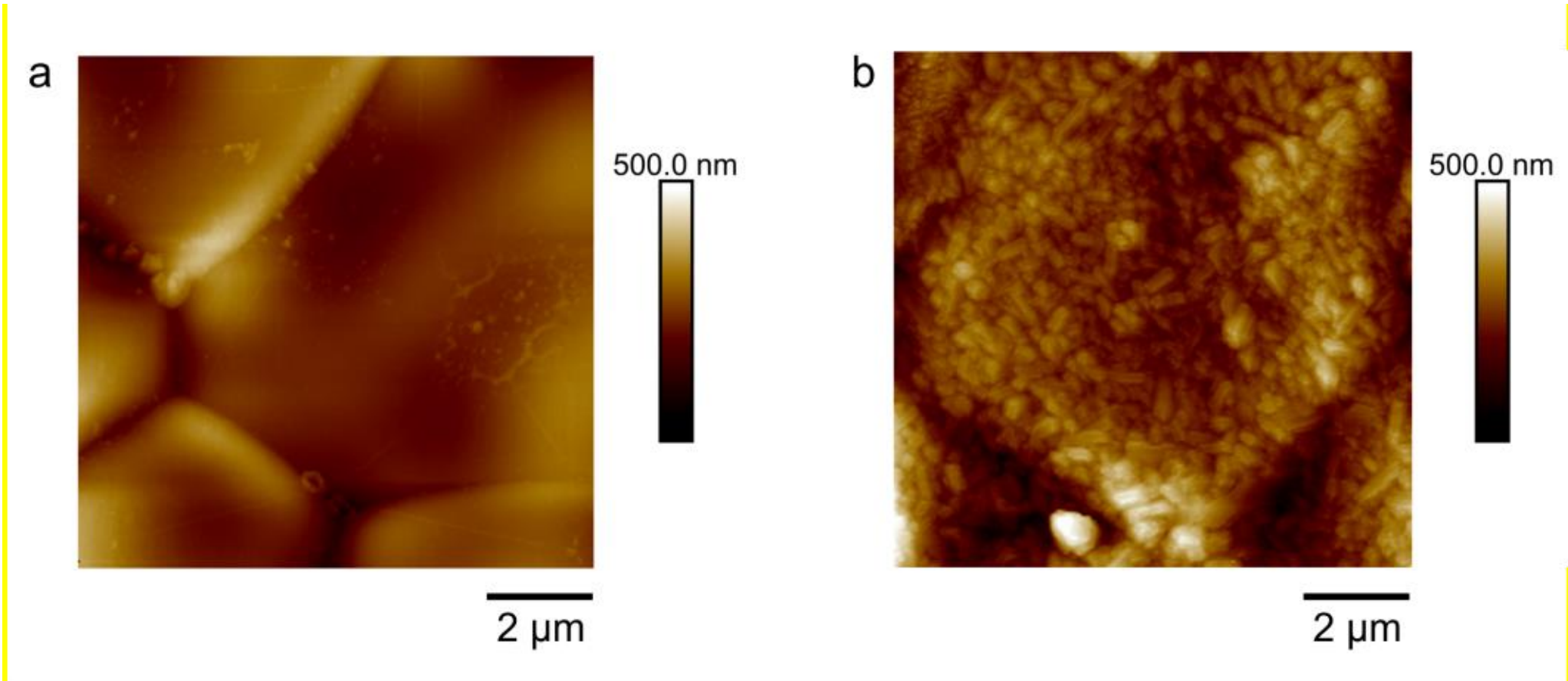

Supplementary Fig. 9. AFM images of (a) the surface of the pristine electrolyte and (b) the surface of the precursor-based interlayer. The electrolyte surface exhibited an RMS roughness of 46.7 ± 16.8 nm and a surface area of 1.010 ± 0.010 μm²/μm², while the interface surface showed an RMS roughness of 48.6 ± 9.5 nm and a surface area of 1.066 ± 0.019 μm²/μm².

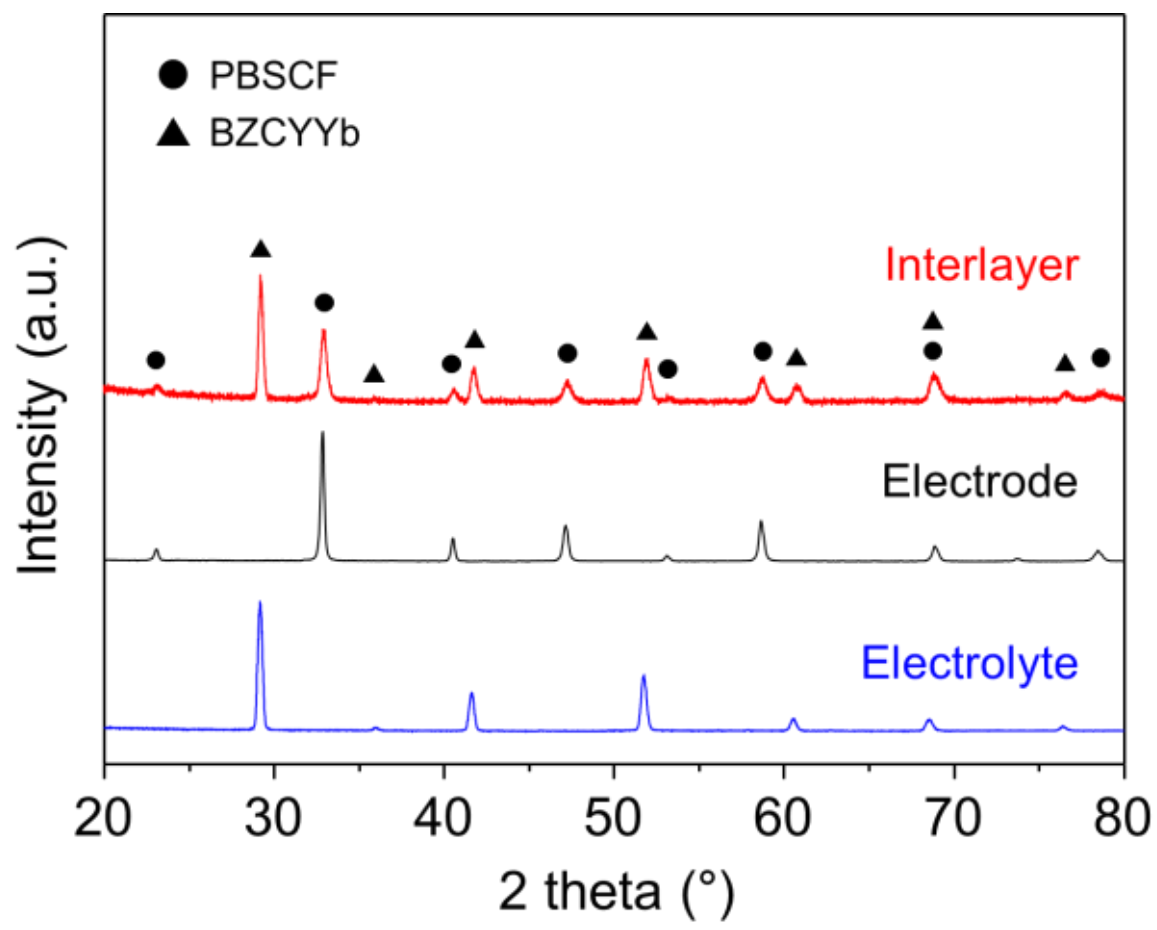

Supplementary Fig. 11. XRD patterns of the precursor-based interlayer, powder electrode, and electrolyte. The crystallite sizes of the electrode and interlayer are 25.1 ± 2.7 nm and 21.1 ± 1.9 nm, respectively.

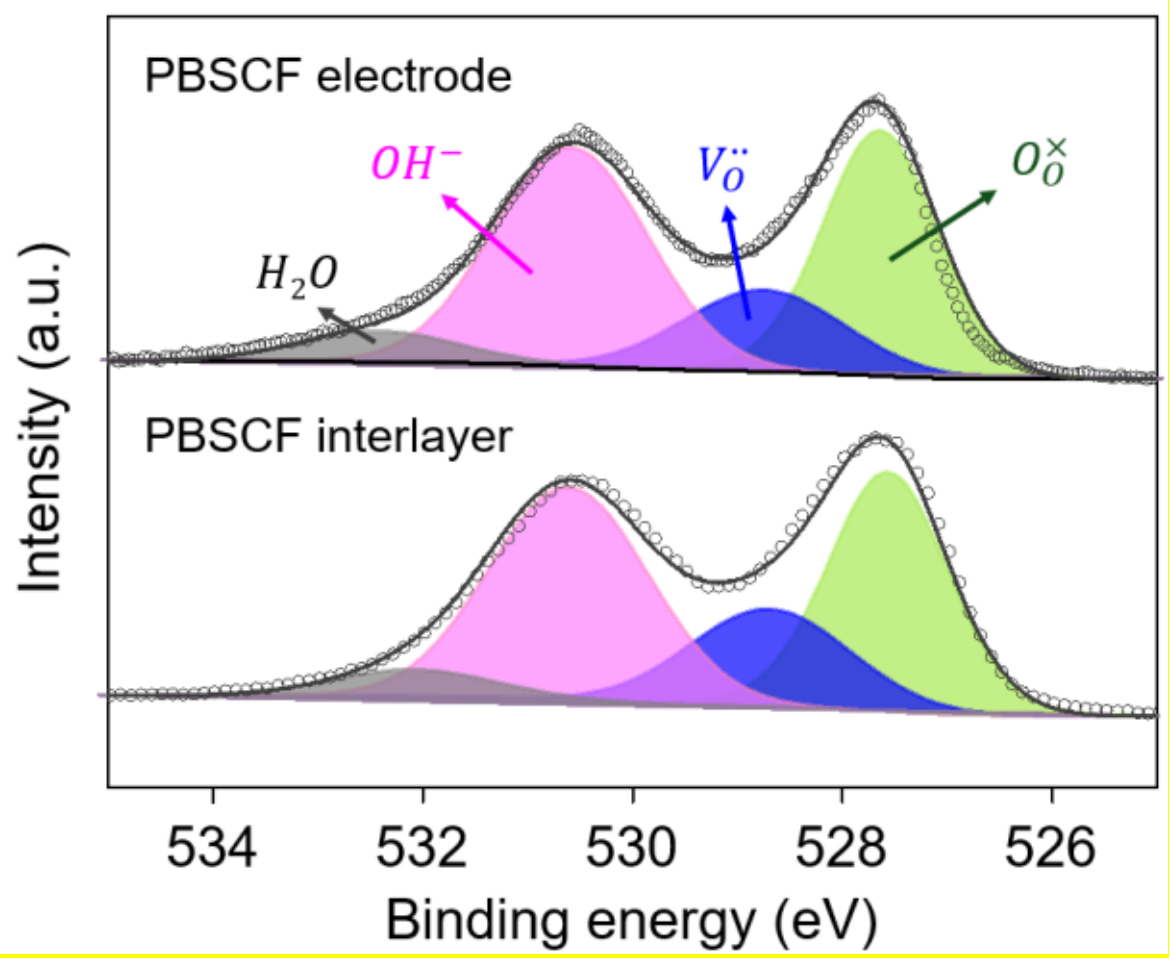

Supplementary Fig. 25. XPS O 1s spectra of PBSCF electrode and PBSCF interlayer.

Supplementary Table 7. Relative peak areas of each component in XPS O 1s spectra of PBSCF electrode and PBSCF interlayer.

	$O_O^{\times}$	$V_O^{\cdot\cdot}$	OH^-	H_2O
PBSCF electrode	0.36	0.16	0.42	0.06
PBSCF interlayer	0.35	0.18	0.41	0.06

Table R5. Atomic ratios of PBSCF electrode and PBSCF interlayer from XPS.

	Atomic ratio over (Pr+Ba+Sr+Co+Fe)				
	Pr	Ba	Sr	Co	Fe
PBSCF electrode	0.260	0.307	0.154	0.153	0.126
PBSCF interlayer	0.257	0.315	0.145	0.149	0.133

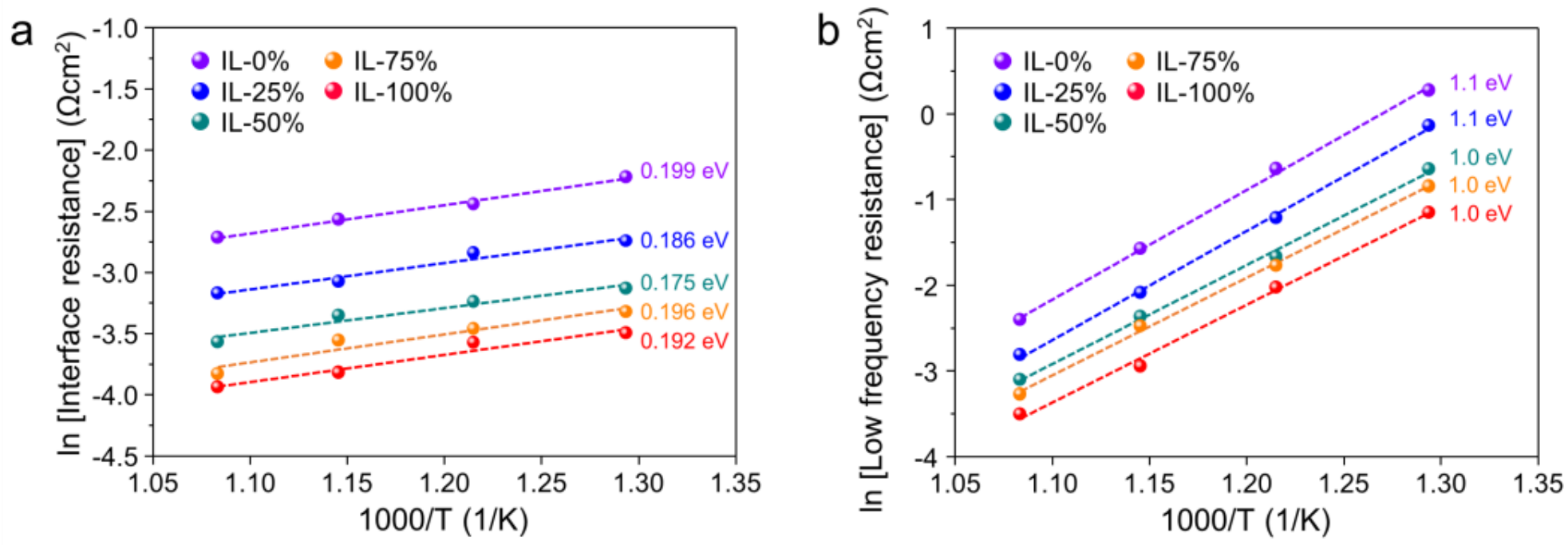

Fig. R18. Arrhenius plots of the (a) ohmic interface resistance and (b) polarization interface resistance for IL-0%, IL-25%, IL-50%, IL-75%, and IL-100% in the temperature range of 650–500 °C.

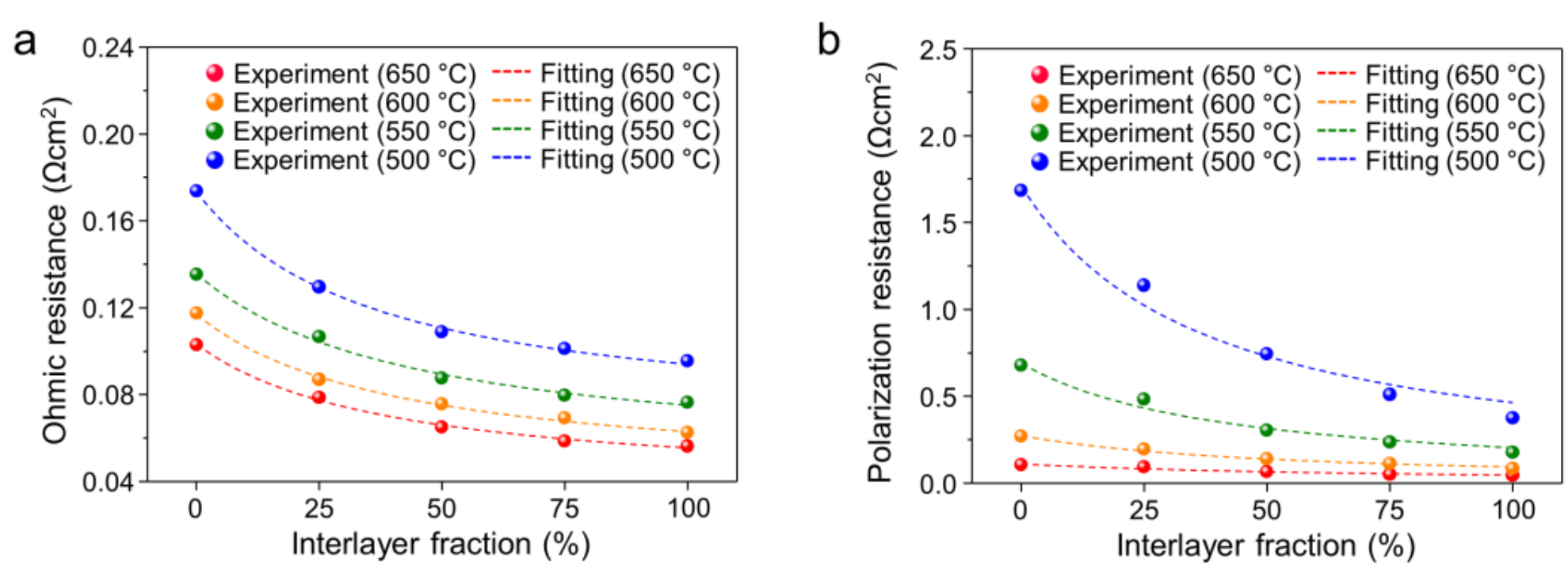

Fig. R19. (a) Ohmic resistance and (b) polarization resistance as a function of the interlayer fraction with corresponding fitting results.

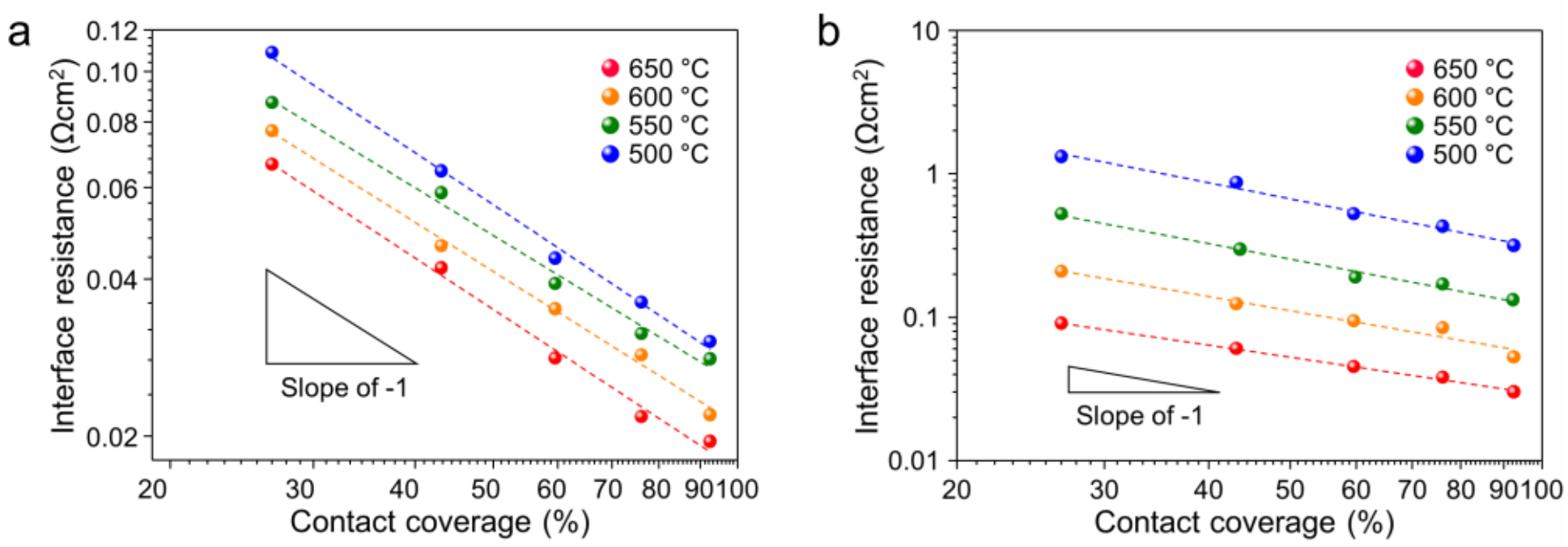

Fig. R20. Correlation between contact coverage and the interface components of (a) ohmic resistance and (b) polarization resistance in double logarithmic coordinates.

5. The authors claim that the electrode/electrolyte and interlayer/electrolyte interfaces exhibit "nearly identical specific contact resistance," which is surprising given their distinct chemistries, defect structures, and electronic properties. This conclusion is derived solely from equivalent circuit fitting, with no supporting evidence from chemical, electronic, or structural characterization to support.

Thank you for the important comment. We agree that, in the originally submitted manuscript, the conclusion regarding similar specific contact resistance was primarily derived from equivalent circuit fitting, and lacked independent supporting evidence.

In the revised manuscript, this issue has been addressed by introducing independently measured parameters into the analysis. Specifically, the contact coverage values for both interfaces are now obtained from model-independent structural and image-based measurements. The non-interface resistance was independently determined from electrolyte thickness-dependent experiments. The interface resistance was then calculated by subtracting the non-interface resistance from the experimentally measured total ohmic resistance. Subsequently, the specific contact resistance was obtained by normalizing the interface resistance by the corresponding contact coverage. Therefore, the specific contact resistance is no longer a purely fitted parameter, but is derived from a combination of independently measured structural quantities and experimentally obtained resistance values.

The calculated specific contact resistances for two interfaces are comparable for all temperature range (Table S6). While the powder-based PBSCF and precursor-based PBSCF may exhibit differences in chemistries, defect structures, and electronic properties, these differences are relatively small in the present system, as discussed in our response to Comment 4. As a result,

two interfaces exhibit similar intrinsic interfacial resistivity, which is reflected in the comparable specific contact resistance values. Therefore, the observed similarity in specific contact resistance is reasonable and not an artifact of model fitting. Instead, it supports the conclusion that the reduction in the interfacial resistance is governed primarily by the increase in contact coverage, rather than by changes in intrinsic interfacial properties.

We have revised the manuscript and Supplementary Information as follows:

[line 26, page 7 in the revised manuscript]

Fitting results revealed that the specific contact resistance, defined as the interface resistance normalized by the contact coverage, was nearly identical for both interface types: the electrode/electrolyte interface and the interlayer/electrolyte interface, consistent with the values independently derived from measured structural quantities and experimentally obtained resistance values.[20] Detailed calculations are provided in Supplementary Table 6. This similarity further substantiates that contact coverage is the primary factor governing interface resistance.

Supplementary Table 6. Calculated specific contact resistance.

	Temperature (°C)	IL-0%	IL-100%
Total ohmic resistance (Ωcm^2)	650	0.1030	0.0563
	600	0.1176	0.0626
	550	0.1354	0.0754
	500	0.1739	0.0965
Non-interface resistance (Ωcm^2)	650	0.0367	
	600	0.0407	
	550	0.0483	
	500	0.0652	
Interface resistance (Ωcm^2)	650	0.0663	0.0196
	600	0.0769	0.0219
	550	0.0871	0.0271
	500	0.1087	0.0313
Contact coverage (%)	650	26.7 ± 4.1	92.6 ± 0.6
	600		
	550		
	500		
Specific contact resistance (Ωcm^2)	650	0.0177 ± 0.0027	0.0181 ± 0.0001
	600	0.0205 ± 0.0032	0.0203 ± 0.0001
	550	0.0233 ± 0.0036	0.0251 ± 0.0002
	500	0.0290 ± 0.0045	0.0290 ± 0.0002

[Interface resistance] = [Total ohmic resistance] - [Non-interface resistance]

[Specific contact resistance] = [Interface resistance] × [Contact coverage/100]

6. The authors mention a same composition PBSCF interlayer without providing mechanistic justification, literature precedent, or comparative controls demonstrating why this choice is necessary, yet it is presented as a systematic strategy. No evidence is offered to show that a chemically identical interlayer is uniquely beneficial or required.

Thank you for the comment. In this study, the use of a PBSCF interlayer with the identical composition to the electrode was not intended to claim that such configuration is uniquely beneficial or required. Instead, it was deliberately chosen as a controlled model system to isolate the effect of interfacial contact coverage from other variables, particularly those related to differences in chemistry, microstructure, defect concentration, and intrinsic catalytic activity. By maintaining identical composition between the electrode and the interlayer, the present system minimizes variations in intrinsic material properties, allowing the observed changes in electrochemical performance to be attributed primarily to structural factors, that is the increase in interfacial contact coverage. Accordingly, the chemically identical interlayer serves as a reference configuration to decouple structural effects from chemical effects.

Furthermore, as described in our response to Comment 8, the interlayer strategy is not limited to the PBSCF system. Additional experiments using different electrode materials, including $Gd_{0.3}Ca_{2.7}Co_{3.82}Cu_{0.18}O_{9-\delta}$ (GCCCO), $Ba_{0.5}Sr_{0.5}Co_{0.8}Fe_{0.2}O_{3-\delta}$ (BSCF), and $La_{0.6}Sr_{0.4}CoO_{3-\delta}$ (LSC), show consistent improvements in electrochemical performance, indicating that the beneficial effect of the interlayer is not dependent on a specific material composition. Therefore, the use of a chemically identical interlayer is not presented as a uniquely beneficial or required condition, but as a model system that enables a clear interpretation of the underlying mechanism, while the broader applicability of the approach is supported by addition experimental validation.

We have revised the manuscript as follows:

[line 10, page 9 in the revised manuscript]

This conclusion was further supported by the strong linear correlation with an R^2 of ~0.978 observed in the normalized Arrhenius plots of all interface resistance values (Fig. S24).[12] Importantly, this interpretation does not imply that the contact coverage is the only interfacial variable governing electrochemical performance. Therefore, compositionally identical PBSCF was used for the electrode and interlayer to provide a controlled model system that minimizes variations in other material-related factors, including chemistry, microstructure, and defect concentration. This design is supported by TEM point EDS analysis confirming similar chemical composition (Fig. S10 and Table S1), AFM showing comparable surface roughness (Fig. S9), XRD indicating similar crystallite sizes (Fig. S11), XPS O 1s analysis showing comparable oxygen vacancy-related features (Fig. S25 and Table S7), and the nearly unchanged activation energies (Figs. S18 and S23).

[line 2, page 11 in the revised manuscript]

These values represent the highest reported values for state-of-the-art PCECs, especially those operating below 500 °C (Figs. 3(c and f) and Supplementary Table 8).[1,5-10,36-43] To further examine the generality of the interface-engineering strategy, the precursor-based interlayer strategy was further tested with additional air electrode materials, including $Gd_{0.3}Ca_{2.7}Co_{3.82}Cu_{0.18}O_{9-\delta}$ (GCCCO)[9], $Ba_{0.5}Sr_{0.5}Co_{0.8}Fe_{0.2}O_{3-\delta}$ (BSCF)[44], and $La_{0.6}Sr_{0.4}CoO_{3-\delta}$ (LSC)[45] (Supplementary Figs. 28 and 29). The consistent performance improvements observed in these systems indicate that the interlayer strategy is not limited to a specific electrode material and support its broad applicability. This generality is particularly important because,

as PCECs increasingly adopt thinner electrolytes to enhance performance, the interfacial resistance becomes more critical due to its independence from the electrolyte thickness. In addition, at lower temperatures, polarization resistance, primarily governed by interfacial processes, becomes the major performance-limiting factor. Therefore, the interlayer-based approach offers a scalable and effective strategy to overcome the key challenges in advancing high-performance, low-temperature PCECs.

7. The 0–100% interlayer fraction series appears arbitrary, with no conceptual or experimental rationale for why these fractions are meaningful or physically interpretable. Intermediate “coverage” values are not measured but inferred from the model, further weakening the claimed systematicity.

Thank you for this important comment. This point is closely related to concern raised in Comment 3. However, the issue here is more about the rationale and systematicity of the interlayer fraction series itself.

The interlayer fractions of 0%, 25%, 50%, 75%, and 100% were intentionally designed to span two structurally distinct interfacial configurations: the powder-based electrode/electrolyte interface (IL-0%) and the precursor-based interlayer/electrolyte interface (IL-100%). The intermediate samples therefore represent controlled composite interfaces with defined area fractions of these two configurations, allowing the contact coverage to vary systematically and monotonically, rather than being as arbitrary interpolation points. This design enables a systematic evaluation of electrochemical performance as a function of contact coverage, while

simultaneously considering the characteristics of two structurally distinct interfaces. In addition, as clarified in our response to Comment 3, in the modified model, the contact coverage values for the intermediate samples are not inferred from the model, but are determined from the known sample geometry and the independently measured values of two distinct configurations (Table R6).

Table R6. Interlayer fractions and corresponding contact coverage of model samples.

Sample name	IL-0%	IL-25%	IL-50%	IL-75%	IL-100%
Interlayer fraction (%)	0	25	50	75	100
Contact coverage (%)	26.7	43.2	59.7	76.1	92.6

8. Important experimental parameters, such as the fixed interlayer thickness, are not justified, and no optimization or sensitivity analysis is provided. Without a parameter space exploration or sensitivity analysis, the generality and reproducibility of the conclusions remain questionable. Readers cannot assess whether the reported benefits are universal or specific to a narrow set of conditions.

Thank you for the important comment. We agree that the optimization process and the generality of the proposed interlayer strategy were not sufficiently discussed in the original manuscript. We address these points separately as below.

Regarding the optimization process:

The desired characteristics for the ideal interlayer include a highly conformal and dense morphology to maximize the interfacial density for increasing both the contact coverage and the electrochemical reaction sites, while remaining sufficiently thin to minimize ion diffusion length. We optimized two key ESD process parameters of the substrate temperature and spray duration. To clarify the optimization process of the precursor-based ESD interlayer, we expanded the parameter space beyond the previously investigated conditions (substrate temperatures of BP50, BP100, BP150, and BP200 and spray durations of 4 h, 6 h, 8 h, and 10 h) to include a higher substrate temperature of BP250 and extended spray durations of 16 h and 24 h. Corresponding electrochemical analyses were also performed for each condition to further support the structural optimization process, as shown in Figs. S5–S8.

To obtain a conformal and dense interlayer, the substrate temperature was optimized to suppress ring-like, non-uniform deposition caused by capillary flow within atomized droplets. In droplet-based deposition processes, substrate temperature strongly affects solvent evaporation behavior and droplet size, thereby regulating capillary flow and the resulting film morphology. Five substrate temperatures (BP50, BP100, BP150, BP200, and BP250), corresponding to 50%, 100%, 150%, 200%, and 250% of the solvent boiling point (190 °C), were investigated (Fig. S5). At a substrate temperature of BP50 (95 °C), insufficient solvent evaporation resulted in partially agglomerated structures. Increasing the substrate temperature to BP100 (190 °C) and BP150 (285 °C) intensified capillary flow, driving solutes toward droplet edges and forming non-uniform ring-like structures. At the substrate temperatures of BP200 (380 °C) and BP250 (475 °C), the droplet size decreased below the critical threshold for capillary flow generation, resulting in a highly concentrated and relatively uniform deposition. The corresponding electrochemical analyses (Fig. S6) showed that the peak power density reached its maximum, while both ohmic and polarization resistances were minimized,

as the substrate temperature increase up to BP200 (380 °C), and then became saturated at higher temperature. Therefore, BP200 (380 °C) was selected as the optimal substrate temperature.

To maximize the interfacial coverage while maintaining the interlayer sufficiently thin, the spray duration was optimized. SEM images (Fig. S7) revealed that coverage on the BZCYYb electrolyte surface increased with longer spray duration from 4 to 8 h and became saturated with longer spray duration, while the interlayer thickness continuously increased up to 24 h. Electrochemical analyses (Fig. S8) showed a concurrent increase in peak power density and a decrease in both ohmic and polarization resistances with increasing spray duration up to 8 h. Beyond 8 h, further thickening of the interlayer led to a slight increase in the ohmic resistance, which we attribute to an increased ion diffusion length through a thicker interlayer. Accordingly, 8 h was selected as the optimal spray duration.

The final optimization conditions were therefore BP200 with an 8 h spray duration, which yielded a highly conformal, dense, and thin interlayer that maximizes interfacial density while minimizing resistive losses.

The revised manuscript and Supplementary Information are as follows:

[line 19, page 4 in the revised manuscript]

We optimized two key ESD process parameters of the substrate temperature and spray duration, as shown in Supplementary Figs. 5–8. The substrate temperature was optimized to suppress the formation of ring-like structures caused by capillary flow within the droplets (Supplementary

Figs. 5 and 6), thereby facilitating the fabrication of the desired dense and uniform interlayer.[25] Subsequently, the spray duration was optimized to achieve the thin and dense interlayer on the electrolyte surface (Supplementary Figs. 7 and 8).

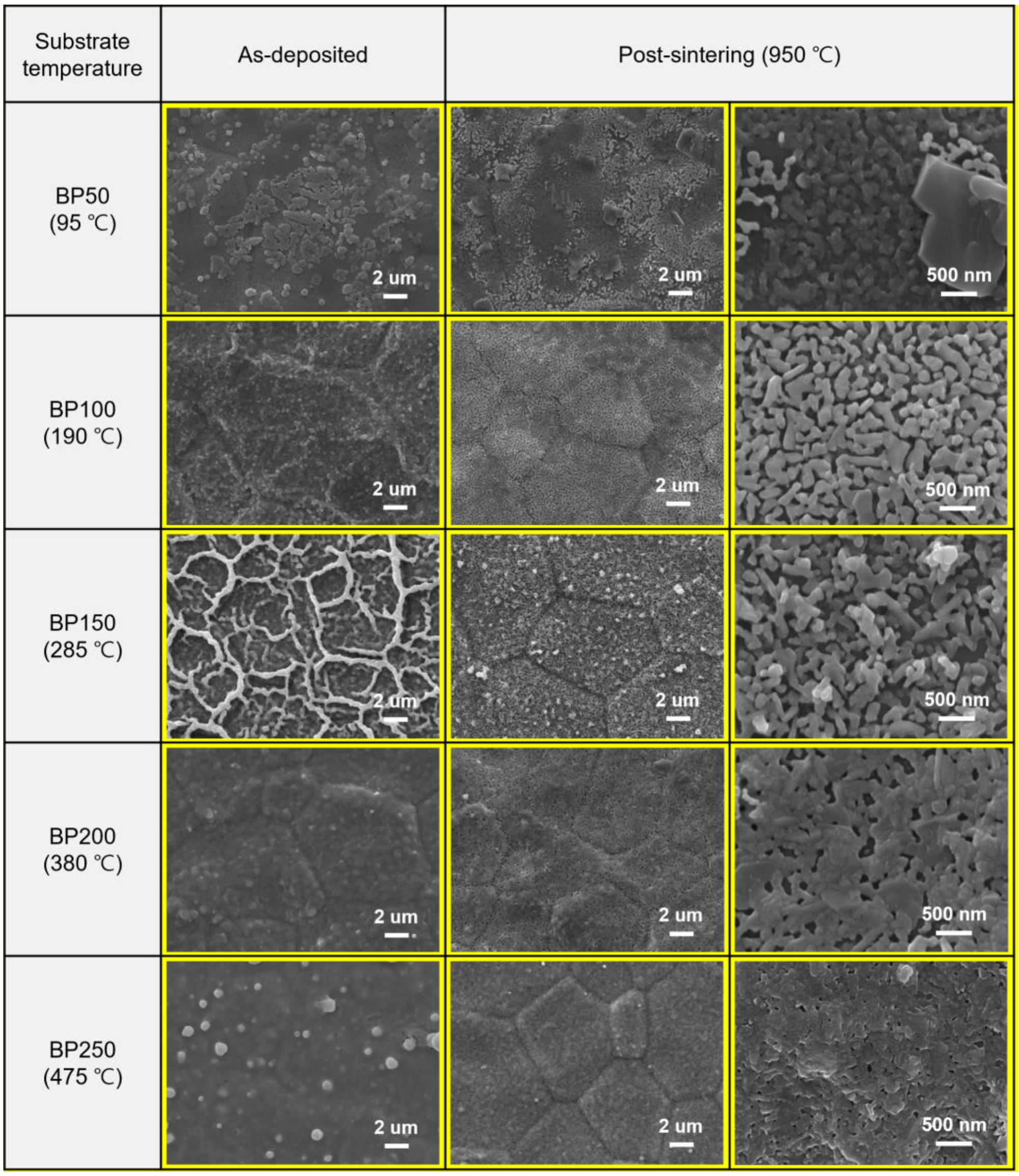

Supplementary Fig. 5. Structural characterization of the precursor-based interlayer was conducted at five substrate temperatures: BP50, BP100, BP150, BP200, and BP250, corresponding to 50%, 100%, 150%, 200%, and 250% of the solvent boiling point (190 °C), respectively. Surface SEM images were analyzed to evaluate the interlayer morphology in both

the as-deposited state and after sintering at 950 °C. In droplet-based deposition processes, substrate temperature strongly affects solvent evaporation behavior and droplet size, thereby regulating capillary flow and the resulting film morphology. Five substrate temperatures (BP50, BP100, BP150, BP200, and BP250), corresponding to 50%, 100%, 150%, 200%, and 250% of the solvent boiling point (190 °C), were investigated. At a substrate temperature of BP50 (95 °C), insufficient solvent evaporation resulted in partially agglomerated structures. Increasing the substrate temperature to BP100 (190 °C) and BP150 (285 °C) intensified capillary flow, driving solutes toward droplet edges and forming non-uniform ring-like structures. At the substrate temperatures of BP200 (380 °C) and BP250 (475 °C), the droplet size decreased below the critical threshold for capillary flow generation, resulting in a highly concentrated and relatively uniform deposition.

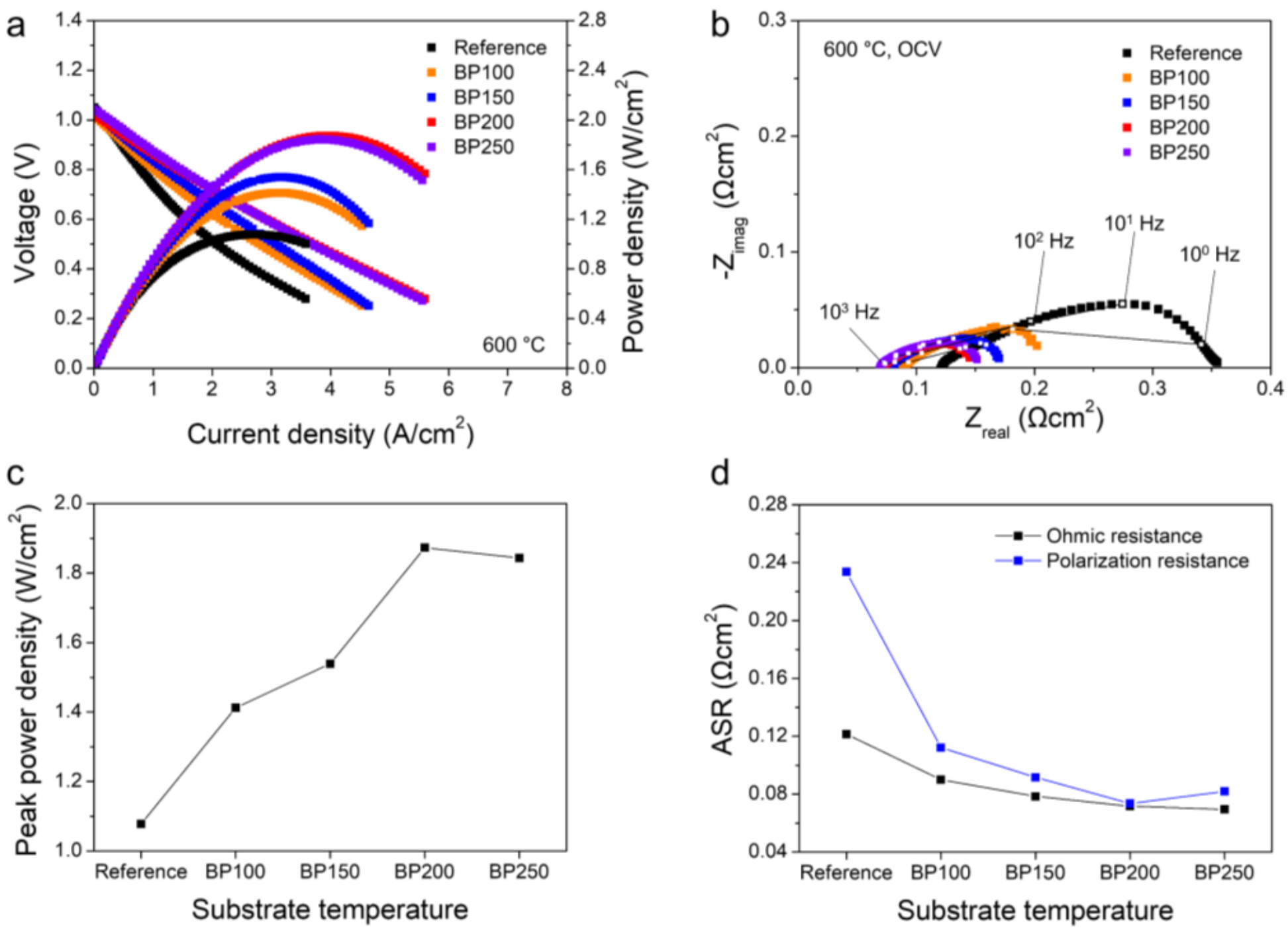

Supplementary Fig. 6. (a) I–V–P curves and (b) Nyquist plots of the reference and interlayer-deposited cells at four substrate temperatures. (c) Peak power density and (d) ohmic and

polarization resistances of the corresponding cells measured at 600 °C. The peak power density reached its maximum, while both ohmic and polarization resistances were minimized, as the substrate temperature increase up to BP200 (380 ℃), and then became saturated at higher temperature.

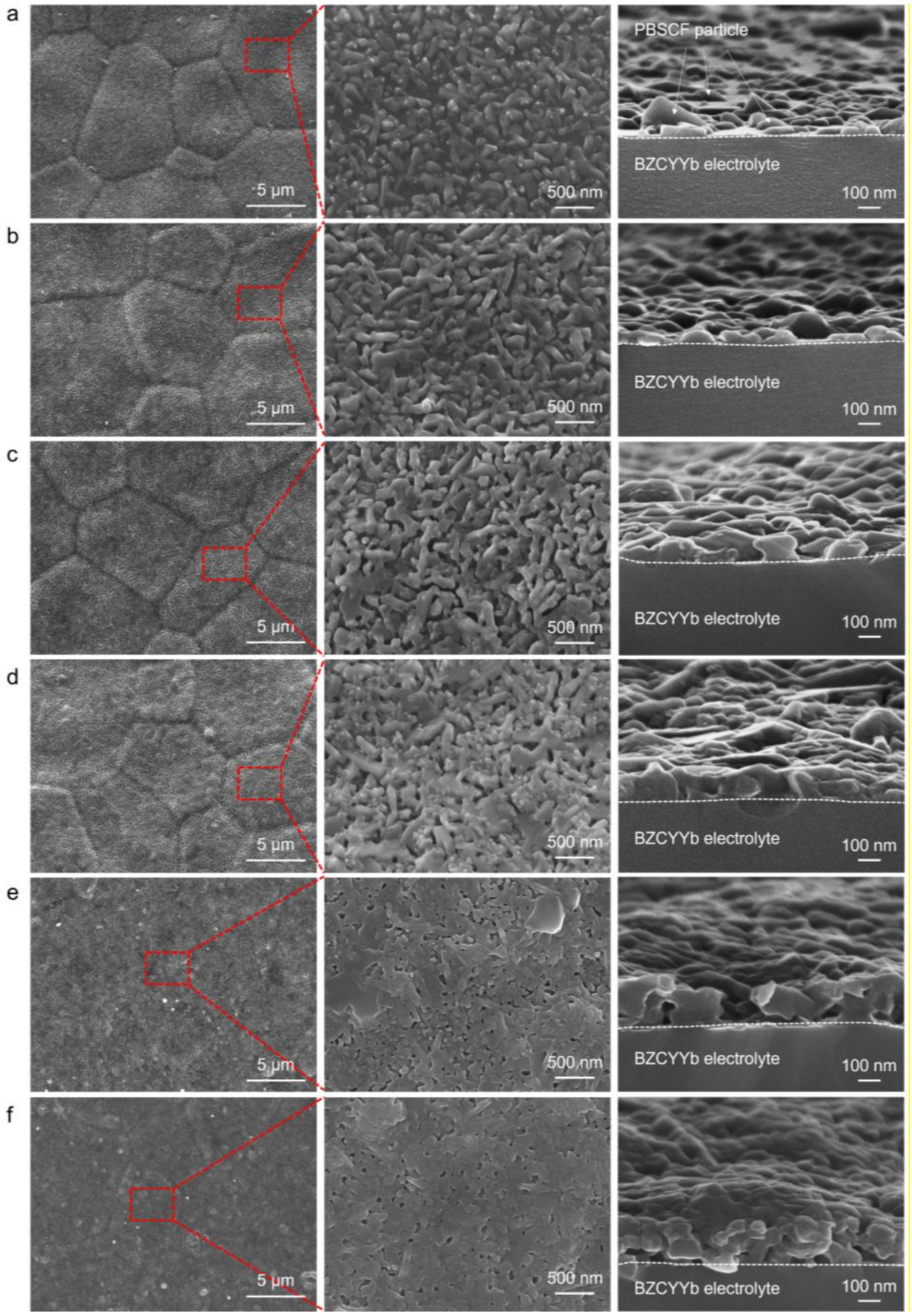

Supplementary Fig. 7. Structural characterization of the precursor-based interlayer deposited

with varying spray durations. Surface and tilted SEM images for spray durations of (a) 4 h, (b) 6 h, (c) 8 h, (d) 10 h, (e) 16 h, and (f) 24 h. The coverage on the BZCYYb electrolyte surface increased with longer spray duration from 4 to 8 h and became saturated with longer spray duration, while the interlayer thickness continuously increased up to 24 h. (~150 nm for 8 h and ~400 nm for 24 h).

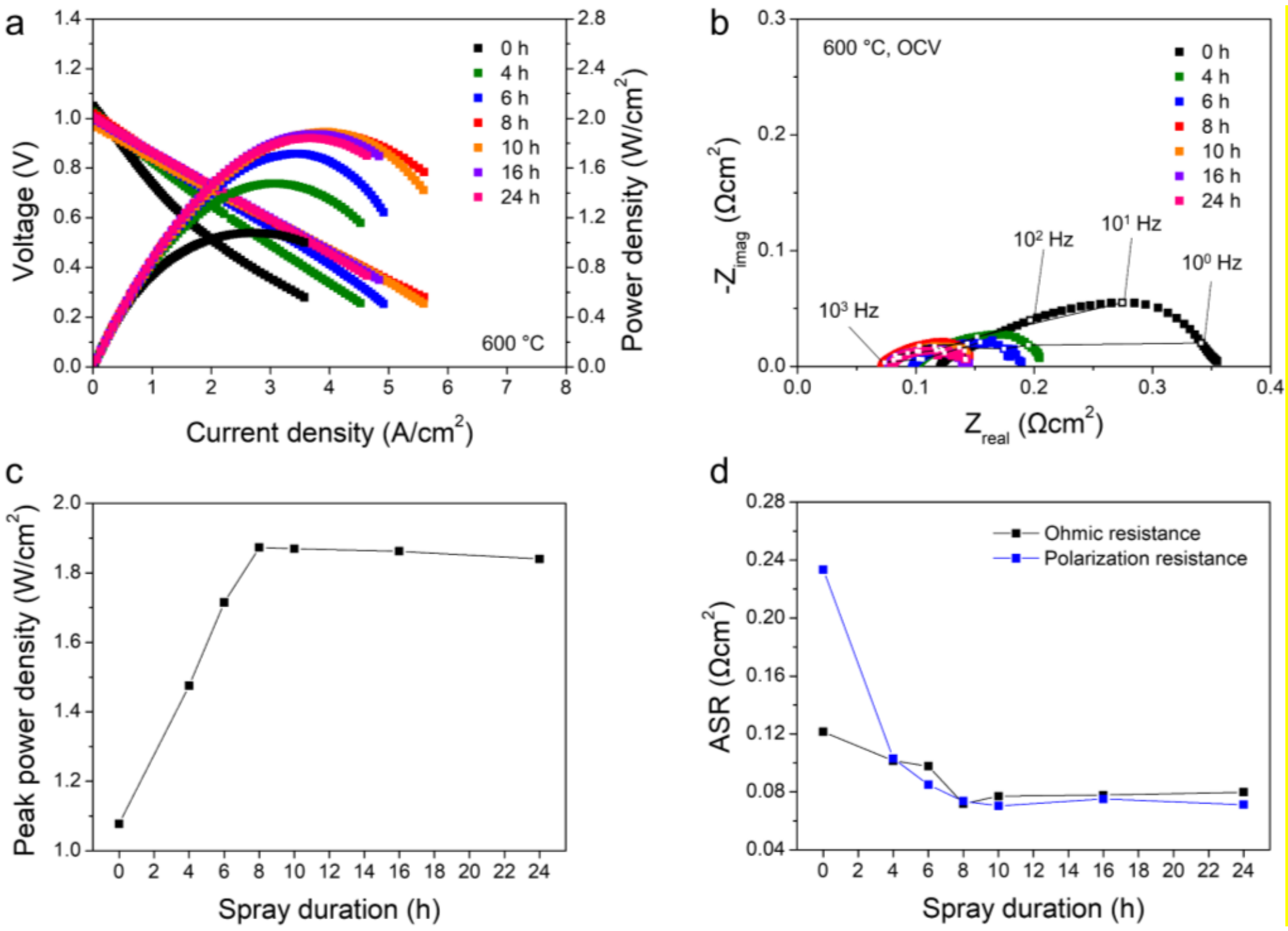

Supplementary Fig. 8. (a) I–V–P curves and (b) Nyquist plots of the reference and interlayer-deposited cells at different spray durations. (c) Peak power density and (d) ohmic and polarization resistances of the corresponding cells measured at 600 °C. Electrochemical analyses showed a concurrent increase in peak power density and a decrease in both ohmic and polarization resistances with increasing spray duration up to 8 h. Beyond 8 h, further thickening of the interlayer led to a slight increase in the ohmic resistance, which we attribute to an increased ion diffusion length through a thicker interlayer. Accordingly, 8 h was selected as the optimal spray duration.

Regarding the generality of the proposed interlayer strategy:

We have performed additional experiments using three representative electrode materials with distinct characteristics: a triple conducting oxide (TCO), $Gd_{0.3}Ca_{2.7}Co_{3.82}Cu_{0.18}O_{9-\delta}$ (GCCCO), and a mixed ionic and electronic conductor (MIEC), $Ba_{0.5}Sr_{0.5}Co_{0.8}Fe_{0.2}O_{3-\delta}$ (BSCF), as an air electrode in protonic ceramic fuel cells (Ni-BZCYYb/BZCYYb/air electrode), and $La_{0.6}Sr_{0.4}CoO_{3-\delta}$ (LSC) as an air electrode in a conventional solid oxide fuel cell (Ni-YSZ/YSZ/GDC/air electrode) [R25-R27]. In all cases, a precursor-based interlayer was deposited using the corresponding electrode precursors, forming a conformal, dense, and thin layer similar to PBSCF-based interlayer in the manuscript, confirming that the fabrication approach is not limited to the specific material. (Fig. S28).

Electrochemical measurements show that the introduction of the interlayer consistently enhances the peak power density in all electrode materials. (Fig. S29). In particular, the ohmic resistance exhibits a comparable reduction for all electrode materials, indicating that improved interfacial contact coverage consistently reduces ohmic resistance regardless of the electrode material. The polarization resistance also decreases for all electrode materials, although the magnitude of reduction varies, reflecting differences in intrinsic reaction kinetics. These results demonstrate that the proposed interlayer strategy is not limited to a specific electrode material, but is broadly applicable to different electrode materials, supporting the generality of the proposed interface engineering approach.

We have revised the manuscript and Supplementary Information as follows:

[line 2, page 11 in the revised manuscript]

These values represent the highest reported values for state-of-the-art PCECs, especially those operating below 500 °C (Figs. 3(c and f) and Supplementary Table 8).[1,5-10,36-43] To further examine the generality of the interface-engineering strategy, the precursor-based interlayer strategy was further tested with additional air electrode materials, including $Gd_{0.3}Ca_{2.7}Co_{3.82}Cu_{0.18}O_{9-\delta}$ (GCCCO)[9], $Ba_{0.5}Sr_{0.5}Co_{0.8}Fe_{0.2}O_{3-\delta}$ (BSCF)[44], and $La_{0.6}Sr_{0.4}CoO_{3-\delta}$ (LSC)[45] (Supplementary Figs. 28 and 29). The consistent performance improvements observed in these systems indicate that the interlayer strategy is not limited to a specific electrode material and support its broad applicability. This generality is particularly important because, as PCECs increasingly adopt thinner electrolytes to enhance performance, the interfacial resistance becomes more critical due to its independence from the electrolyte thickness. In addition, at lower temperatures, polarization resistance, primarily governed by interfacial processes, becomes the major performance-limiting factor. Therefore, the interlayer-based approach offers a scalable and effective strategy to overcome the key challenges in advancing high-performance, low-temperature PCECs.

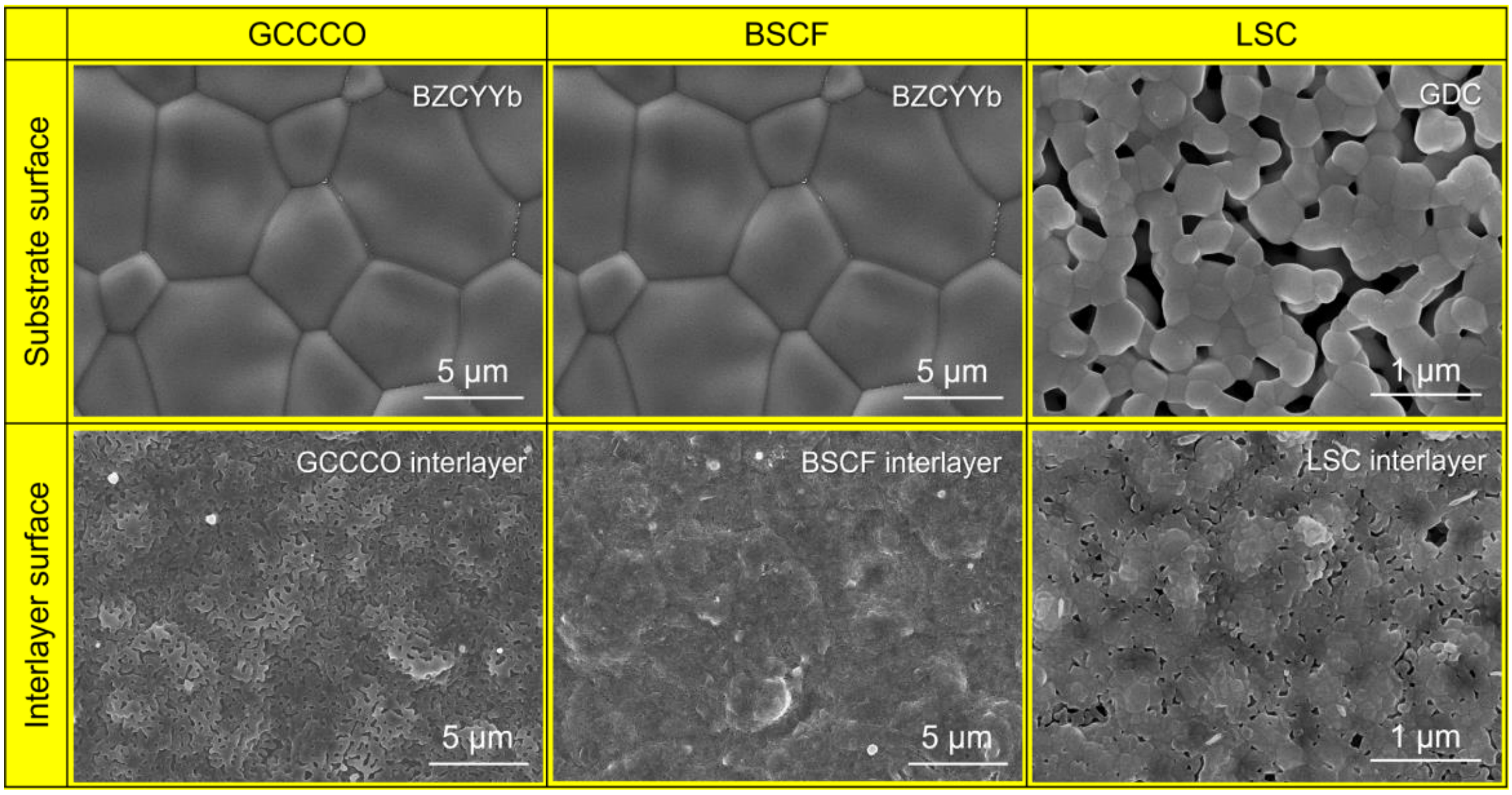

Supplementary Fig. 28. Surface SEM images of the substrates and the precursor-based interlayers.

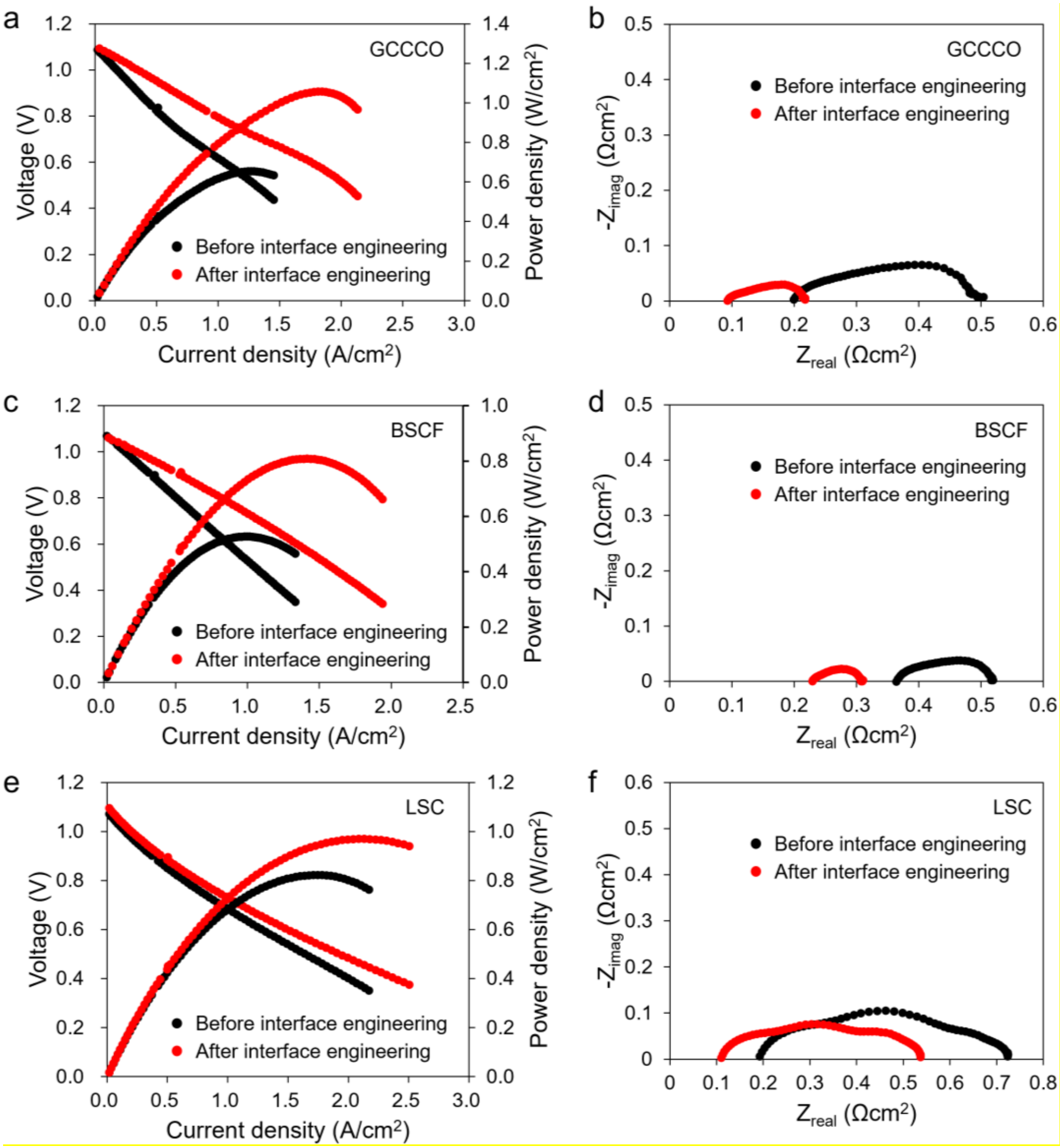

Supplementary Fig. 29. (a) I-V-P curves of the GCCCO interlayer in a Ni-BZCYYb/BZCYYb/GCCCO configuration at 600 °C and (b) Nyquist plots under OCV conditions. (c) I-V-P curves of the BSCF interlayer in a Ni-BZCYYb/BZCYYb/BSCF configuration at 600 °C and (d) Nyquist plots under OCV conditions. (e) I-V-P curves of the LSC interlayer in a Ni-YSZ/YSZ/GDC/LSC configuration at 650 °C and (f) Nyquist plots under OCV conditions.

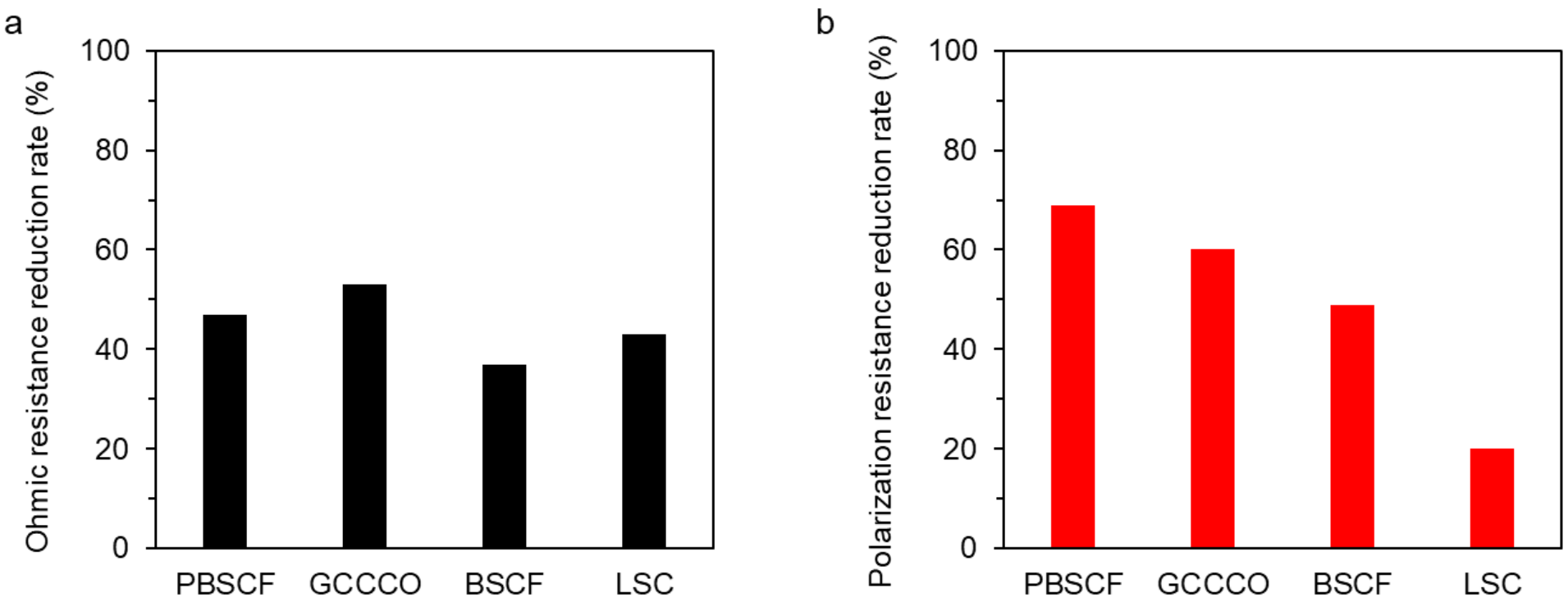

Fig. R21. Reduction rates of (a) ohmic and (b) polarization resistances upon introduction of the interlayer for various electrode materials.

[R25] Saqib, M. et al. Transition from perovskite to misfit-layered structure materials: a highly oxygen-deficient and stable oxygen electrode catalyst. *Energy Environ. Sci.* **14**, 2472–2484 (2021).

[R26] Liu, Z. et al. Strategic atomic trapping at heterointerfaces for protonic ceramic cells. *Nat. Commun.* **16**, 10405 (2025).

[R27] Rehman, S. U. et al. Designing the nano-scale architecture of the air electrode for high-performance and robust reversible solid oxide cells. *Appl. Catal. B* Environ. Energy **333**, 122784 (2023).

Revision Requested: NCOMMS-25-101349A

We thank your consideration of our manuscript for publication in *Nature Communications*. We have revised our manuscript reflecting the reviewers' comments as listed below.

Reviewer #2:

The authors have substantially addressed the major concerns raised in the previous review. The revised manuscript is significantly strengthened by additional control experiments, post-cycling DRT analysis, adhesion tests, and EIS measurements under alternative fuel conditions. The central conclusions are now better supported. Publication can be recommended after minor revision.

1. The adhesion-strength data, fracture-surface SEM images, and Raman results for the intermediate samples IL-25%, IL-50%, and IL-75% should be incorporated into the Supplementary Information, rather than only being discussed in the response letter. In addition, the main text should briefly clarify that this relationship represents an experimentally observed correlation under the present testing conditions, rather than a universal mechanical scaling law.

Thank you for the comment. We have revised the manuscript and the related Supplementary Information as follows:

[line 19, page 13 in the revised manuscript]

The mechanical pull-off test further validated the enhanced adhesion strength (Fig. 4(f)); IL-100% showed ~14.4 MPa, which was ~2.4 times higher than that of IL-0% (~6.0 MPa). For the intermediate samples (IL-25%, IL-50%, and IL-75%), the adhesion strength increased gradually with increasing interlayer fraction (Supplementary Fig. 37). It should be noted, however, that this trend represents an experimentally observed correlation under the present conditions rather than a universal mechanical scaling law, given the complex fracture behavior associated with a multi-interface system. SEM images after the pull-off test revealed that the detachment occurred at the electrode/electrolyte interface in IL-0% and at the electrode/interlayer interface in IL-100% (Supplementary Fig. 38), implying even stronger bonding at the interlayer/electrolyte interface, likely due to co-sintering of compositionally identical materials. Raman spectroscopy confirmed the clear detachment surfaces after the pull-off tests without the residual adhesive, verifying the reliability of the characterization method (Supplementary Fig. 39).

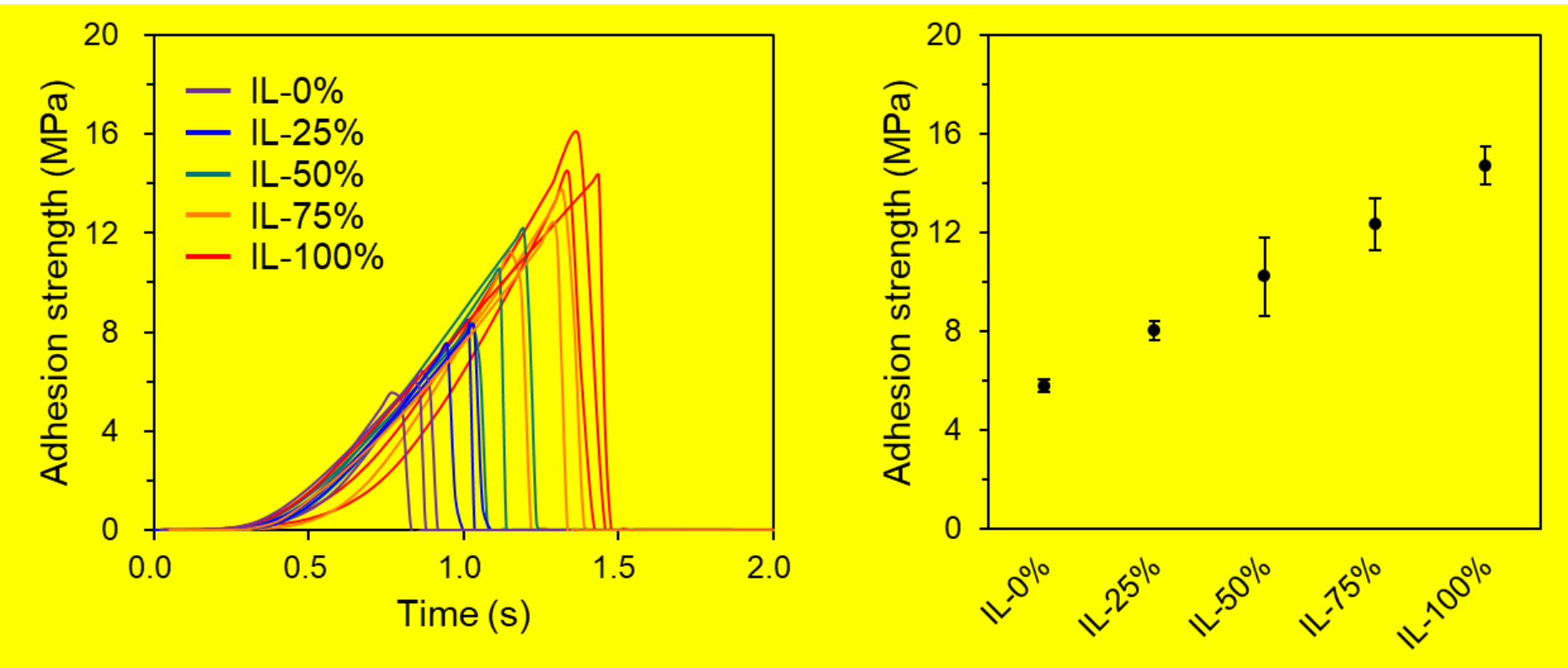

Supplementary Fig. 37. Adhesion strengths at the interfaces of IL-0%, IL-25%, IL-50%, IL-75%, and IL-100% using the mechanical pull-off test. Each sample was tested three times to ensure reliability and reproducibility.

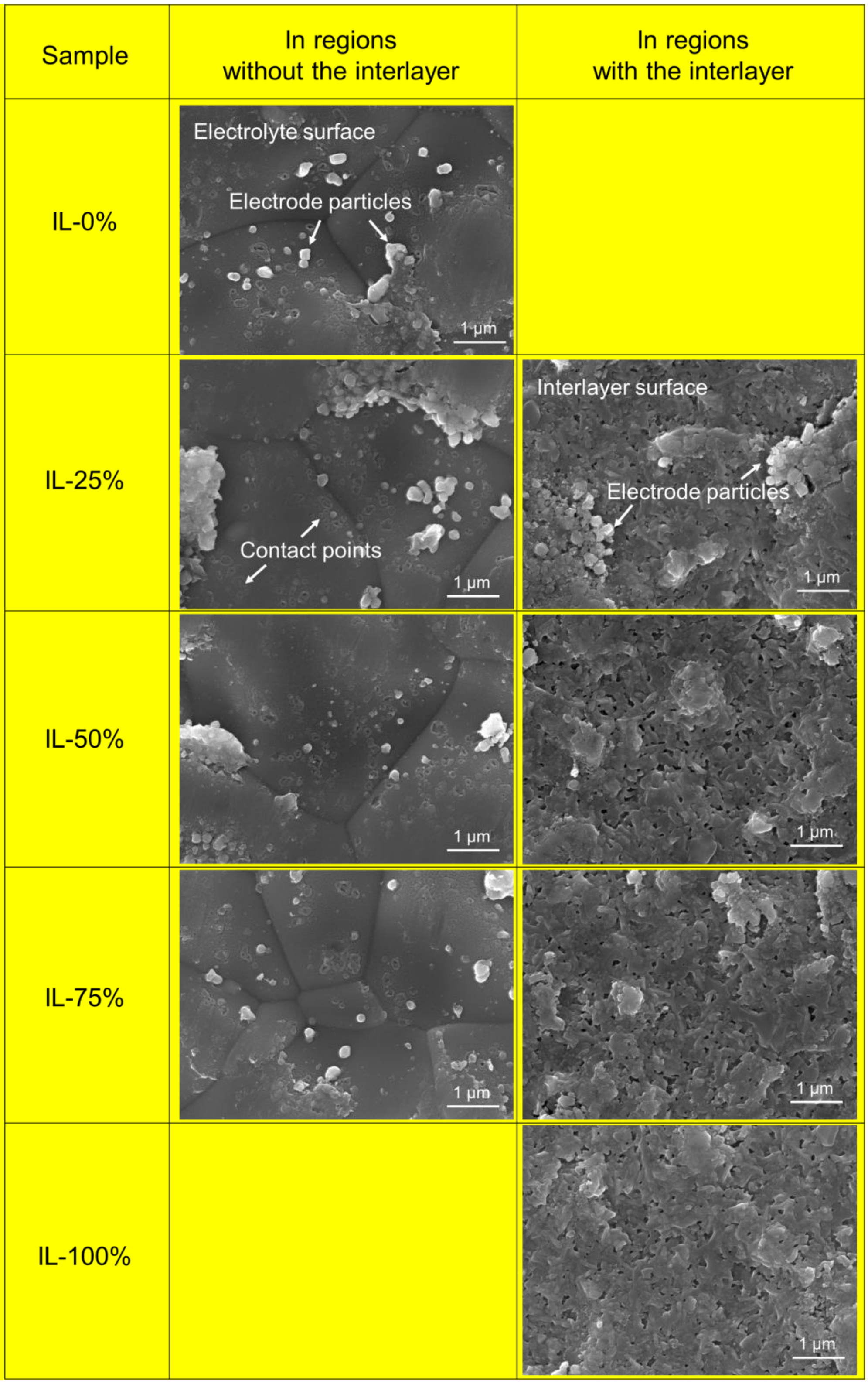

Supplementary Fig. 38. Post-test fracture surface SEM images of IL-0%, IL-25%, IL-50%, IL-75%, and IL-100%.

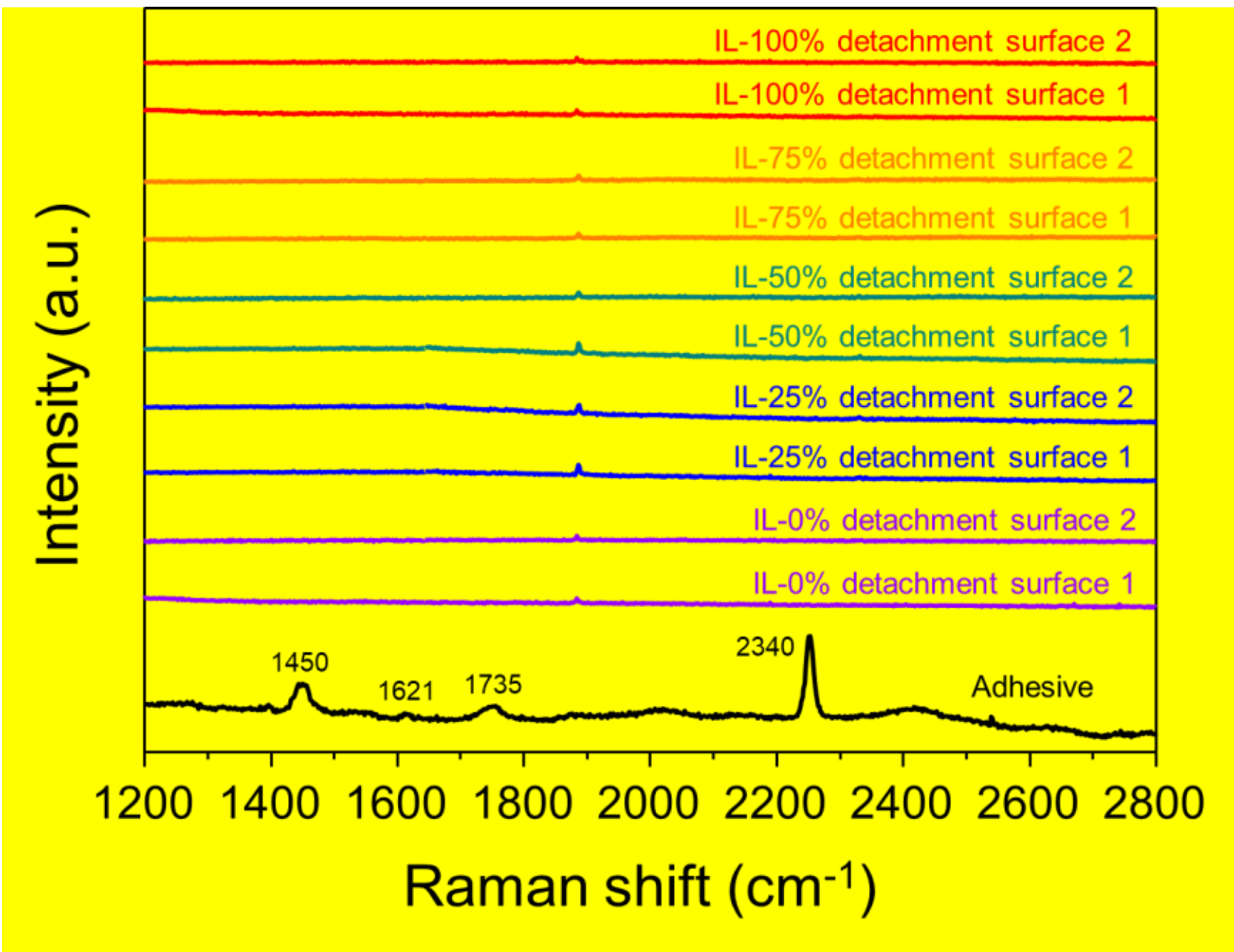

Supplementary Fig. 39. Raman spectroscopy of the detached surfaces after the pull-off test and adhesive. The detachment surfaces on the electrolyte side and the electrode side were designated as detachment surface 1 and 2, respectively.

2. Several minor textual and formatting issues remain in the manuscript. For example, "stem-to-carbon ratio" in the caption of Fig. 5 should be corrected to "steam-to-carbon ratio", and "fundings" in line 444 should be corrected to "findings". These issues should be corrected before publication.

Thank you for the comment. We have corrected the typographical errors in the revised manuscript as follows:

[line 10, page 14 in the revised manuscript]

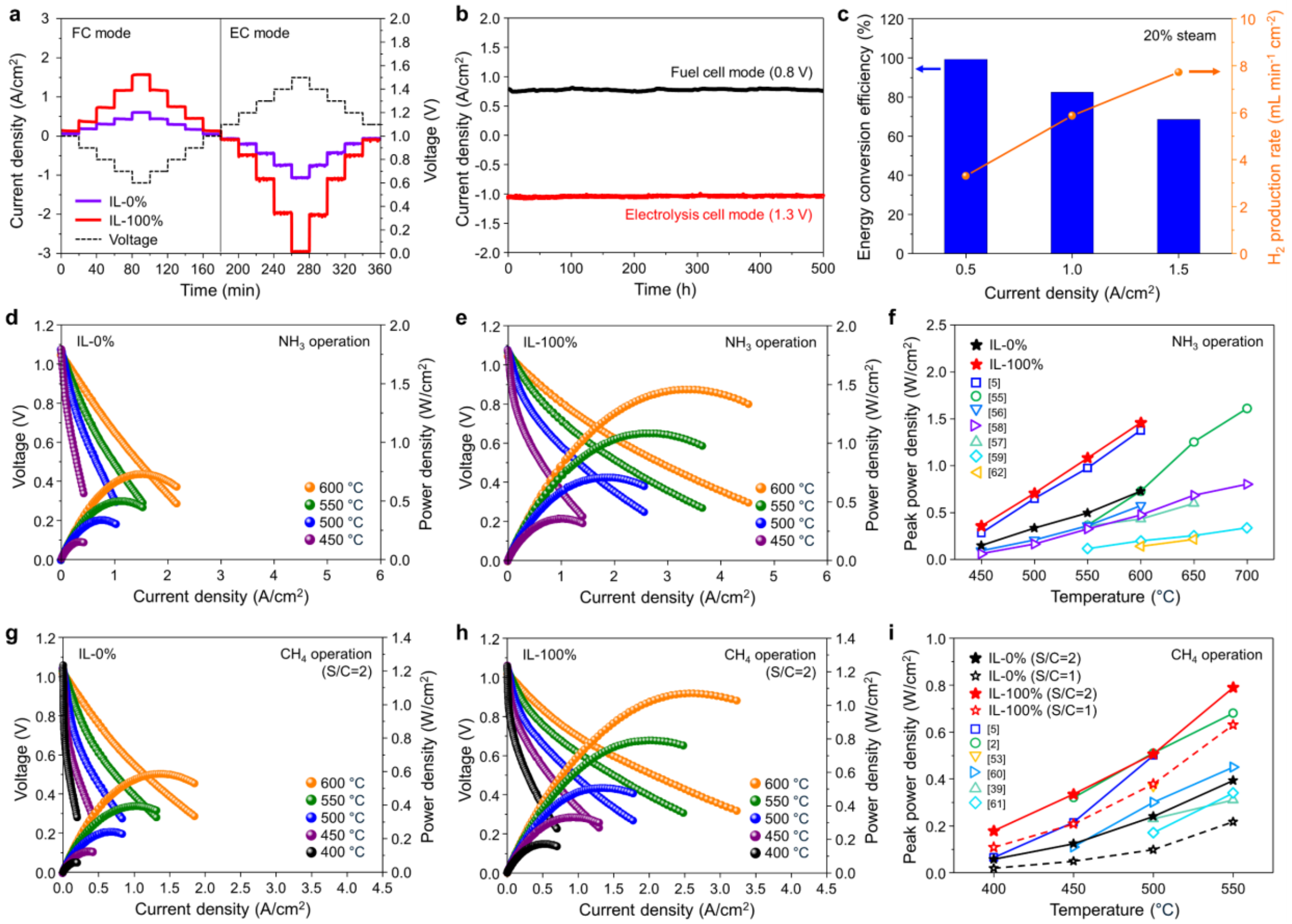

Fig. 5: Exceptionally high-performance and robust PCEC platform demonstrating a versatile energy conversion capability. (a) Current densities under voltage ranges of 0.6–1.0 V (FC mode) and 1.1–1.5 V (EC mode) at 500 ˚C. (b) Long-term stability test at 500 °C under constant voltages of 0.8 V (FC mode) and 1.3 V (EC mode). (c) Energy conversion efficiency and hydrogen production rate of IL-100% evaluated at different current densities under 20% steam (air electrode) at 500 °C. I-V-P curves using ammonia fuel for (d) IL-0% and (e) IL-100% in the temperature range of 600–450 °C. (f) Comparison of peak power densities with previously reported protonic ceramic fuel cells operating with ammonia. I-V-P curves using methane fuel with a steam-to-carbon ratio of 2 for (g) IL-0% and (h) IL-100% in the temperature range of 600–400 °C. (i) Comparison of peak power densities with previously reported protonic ceramic fuel cells and solid oxide fuel cells operating with methane.

[line 11, page 18 in the revised manuscript]

The data that support the findings of this study are available from the corresponding author upon reasonable request.

Reviewer #4:

The manuscript "Interface-Driven Boost in the Performance and Stability of Protonic Ceramic Electrochemical Cells" reported a precursor-based electrostatic spray deposition method to introduce an interlayer to engineer the interface between the protonic ceramic electrolyte and the triple-conducting oxygen electrode. The ohmic resistance, polarization resistance, and interfacial bonding were improved, resulting in improved fuel cell/electrolysis cell performance and thermal cycle stability. The interfacial coverage was correlated with the ohmic area-specific resistance (ASR) and oxygen polarization resistances. I read through the manuscript and rebuttal letter. Although I acknowledge that it is good work. However, I was not convinced to accept this manuscript for publication because of the following two big concerns.

1. The novelty needs to be further justified. As the manuscript notes, even the authors themselves have reported an interfacial-layer-enhancement effect on ohmic ASR, polarization and thermal stability. The authors claim that the method is scalable, which, however, is supported by the large-area cell performance, ASR, and thermal stability. In addition, there is no clue why this method is better than other methods. The method's operating principles are unclear. Why can the precursor deposition prepare dense thin films? The method is electrostatic deposition. It is not clear whether the method is suitable for other material systems.

Thank you for the comments. We address the comments separately as below.

1-1. Novelty relative to our prior work

The novelty needs to be further justified. As the manuscript notes, even the authors themselves have reported an interfacial-layer-enhancement effect on ohmic ASR, polarization and thermal stability.

We acknowledge that our recent work established a quantitative framework for interface engineering in PCECs, including the use of contact coverage as a key descriptor of interfacial properties. While our previous work established a quantitative framework for interface engineering, the present study focuses on its experimental realization and validation through a precursor-based interlayer design.

More specifically, the present study goes beyond the prior work in three important aspects. First, from a processing perspective, it introduces a precursor-based ESD strategy that enables the formation of a thin, dense, and highly conformal interlayer, providing a practical and scalable route to realize the proposed interface engineering framework. In contrast to the powder-based process in the previous work, which inherently limited the achievable contact coverage to ~50%, the present study extends the accessible contact coverage range to nearly complete contact coverage (~92.6%), enabling validation of the framework in a regime that was not accessible in the previous study and leads to a larger reduction in interfacial resistance.

Second, from a modeling and validation perspective, the present study extends the framework beyond a single powder electrode/electrolyte interface. Specifically, it considers structurally distinct interfacial configurations, including the conventional powder electrode/electrolyte interface and the precursor-derived interlayer/electrolyte interface, and introduces an expanded equivalent-circuit framework to analyze and compare their interfacial properties in a unified manner. The significance of this extension is not just the inclusion of two interfaces, but the

ability to quantitatively evaluate how different interfacial architectures with different contact characteristics contribute to the overall electrochemical behavior. This is particularly important because direct quantification of interfacial properties such as contact coverage, and their systematic correlation with electrochemical performance, remains experimentally challenging and is still rarely demonstrated in a rigorous manner.

Third, from the material extension ability perspective, the present study demonstrates that the precursor-based ESD strategy is not limited to PBSCF, but can be applicable to other oxygen electrode materials. Additional experiments were conducted using electrode materials with distinct transport characteristics, including triple conducting oxides (TCOs) and mixed ionic-electronic conductors (MIECs). The precursor-based process produced thin, dense, and highly conformal interlayers across these materials, leading to improved electrochemical performance. These results indicate that the proposed strategy is not limited to a specific electrode material and provide a broader experimental basis for the interface engineering framework.

Therefore, while the conceptual basis was introduced in our previous work, the present work provides its experimental realization, expanded quantitative validation, implementation across structurally distinct interfaces, and extension to multiple electrode materials, thereby substantially broadening the applicability and significance of the proposed interface engineering framework.

1-2. Scalability validation

The authors claim that the method is scalable, which, however, is supported by the large-area cell performance, ASR, and thermal stability.

We agree that the scalability claim in the original manuscript was not sufficiently supported by the larger area cell performance, ASR, and thermal stability. To address this concern within the scope of the present study, we conducted two additional experiments: (i) the larger area PCEC validation with an electrode area of 1 cm^2, and (ii) large area coating uniformity analysis over a 100 cm^2 substrate.

First, to examine whether the interlayer-induced reduction in interfacial resistance is retained in a larger PCEC platform, we fabricated and tested the largest PCEC platform currently available in our laboratory. The cell had a total area of 4 cm^2 and an electrode active area of 1 cm^2, corresponding a 5-fold increase in an active area compared with the cells used in the originally submitted manuscript (the cell area of 1 cm^2 and an electrode active area of 0.2 cm^2). The optical images of the deposited PBSCF interlayer and the subsequently deposited PBSCF electrode are shown in Fig. S28a. IL-100% (1 cm^2) exhibited performance improvements comparable to those observed in IL-100% (0.2 cm^2). At 500 °C, IL-100% (1 cm^2) achieved a peak power density of ~1.0 W/cm^2, corresponding to an improvement of ~106% over IL-0% (1 cm^2), compared with the ~110% improvement observed for the 0.2 cm^2 cells (Fig. S28b). Furthermore, IL-100% (1 cm^2) exhibited an ohmic resistance of ~0.14 Ωcm^2 and a polarization resistance of ~0.36 Ωcm^2, representing reductions of ~39% and ~78%, respectively, relative to IL-0% (1 cm^2). These reductions are comparable to those observed in the 0.2 cm^2 cells, which showed reductions of ~45% and ~77%, respectively (Fig. S28c). These results indicate that the interlayer-induced reduction in interfacial resistance remains effective on a larger cell area.

Second, to evaluate the thermal stability in the large area cell, we performed 100 rapid thermal cycles at 0.8 V with EIS measurements at every 4 cycles after stabilization at 600 °C for 2 h (marked by red circles), the same protocol used for the originally submitted manuscript in a smaller area, as shown in Fig. S29a. IL-0% showed gradual deterioration and failed to recover

its initial performance, while IL-100% demonstrated excellent durability with no detectable performance degradation (Figs. S29b-c). Notably, consistent with the smaller area cells, IL-100% operated under a broader current density range (0.2–1.7 A/cm^2), which was ~50% wider than IL-0% (0.1–1.1 A/cm^2). Nyquist plots (Figs. S29d-e) revealed that IL-0% (1 cm^2) exhibited ~123% and ~88% increases in ohmic and polarization resistances after 100 h, respectively, whereas IL-100% maintained its initial resistances. These trends are consistent with those observed in the 0.2 cm^2 cells, which exhibited corresponding reductions of ~113% and ~106%, respectively. Post-cycling cross-sectional SEM analysis confirmed delamination at the BZCYYb electrolyte/PBSCF electrode interface in IL-0% (Fig. S29f), whereas the IL-100% preserved its initial interfacial structure without delamination (Fig. S29g), consistent with the observations in the 0.2 cm^2 cells. These results demonstrate that the improved interfacial mechanical integrity provided by the precursor-based interlayer is retained in the larger area cell.

Third, to evaluate the area scalability of the precursor-based ESD process, we extended the deposition area to 100 cm^2 on a commercial Ni-YSZ based cell (Elcogen). We note that this experiment was intended to assess large area deposition uniformity rather than PCEC device performance, because commercially available large area PCEC platforms remain highly limited. Structural and compositional uniformity was evaluated at five representative positions across the deposited area. SEM images confirmed that the PBSCF interlayer forms a thin, dense, and conformal layer at all positions (Figs. S30a-c), indicating uniform morphology over the large area substrate. XRD and XPS analyses showed nearly identical diffraction and spectral features across all positions, consistent with those observed in the button cells (Figs. S30d-e). These results support the area scalability of the precursor-based ESD process and show that uniform interlayer formation is not limited to a small area.

We have revised the manuscript and Supplementary Information as follows:

[line 1, page 11 in the revised manuscript]

These values represent the highest reported values for state-of-the-art PCECs, especially those operating below 500 °C (Figs. 3(c and f) and Supplementary Table 9).[1,5-10,36-43] The area scalability of the precursor-based ESD process was evaluated using a larger PCEC platform with an active area of 1 cm^2 and a commercial Ni-YSZ substrate with a deposition area of 100 cm^2. The larger area PCEC maintained the performance enhancement enabled by the interlayer, and structural and compositional analyses confirmed the uniform formation of a thin, dense, and conformal PBSCF interlayer over a 100 cm^2 substrate (Supplementary Figs. 28-30).

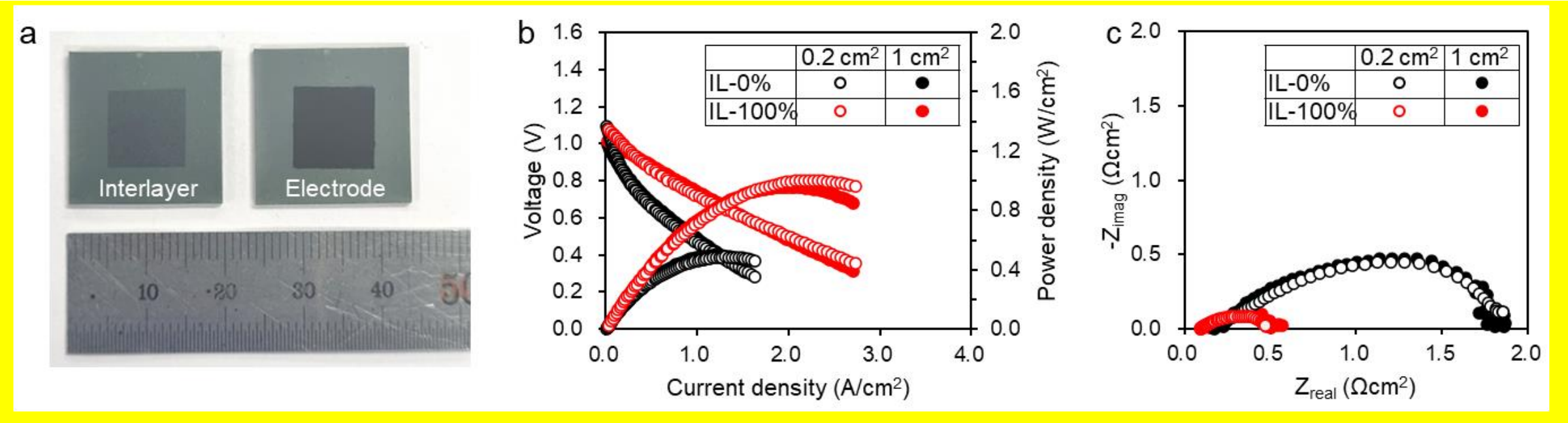

Supplementary Fig. 28. (a) Optical images of PCECs (cell size: 2 × 2 cm^2; active area: 1 × 1 cm^2) after deposition of the PBSCF interlayer and PBSCF electrode. (b) I-V curves of IL-0% and IL-100% at 500 °C and (c) the corresponding Nyquist plots measured under OCV conditions, comparing cells with active areas of 0.2 and 1 cm^2.

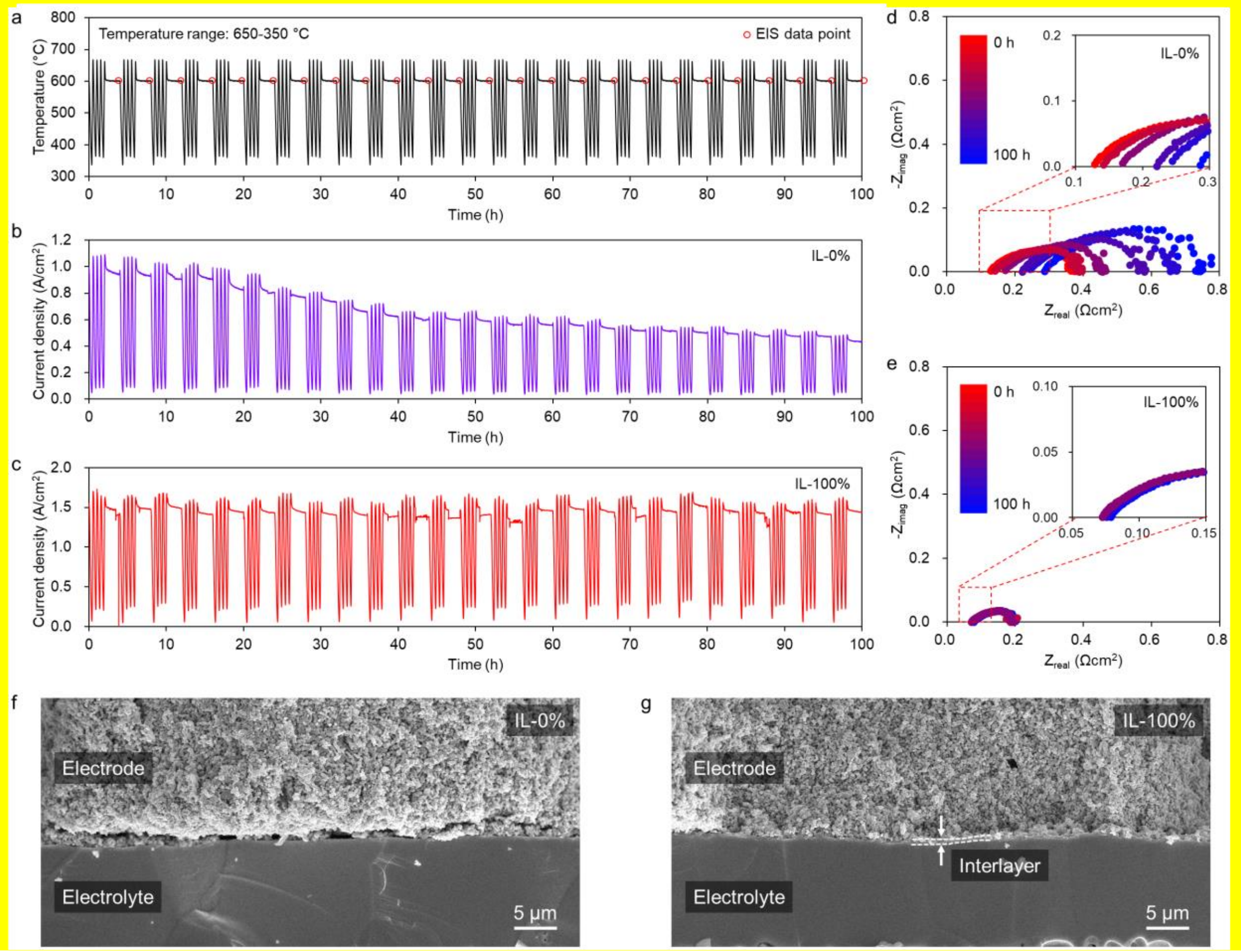

Supplementary Fig. 29. Thermal cycling behavior of larger area PCECs (cell size: 2 × 2 cm^2; effective area: 1 × 1 cm^2). (a) Thermal cycling temperature profile and corresponding output current density under a constant voltage of 0.8 V for (b) IL-0% and (c) IL-100%. Nyquist plots at 600 °C under OCV conditions during thermal cycling for (d) IL-0% and (e) IL-100%. Cross-sectional SEM images at the electrode/electrolyte interface after thermal cycling for (f) IL-0% and (g) IL-100%.

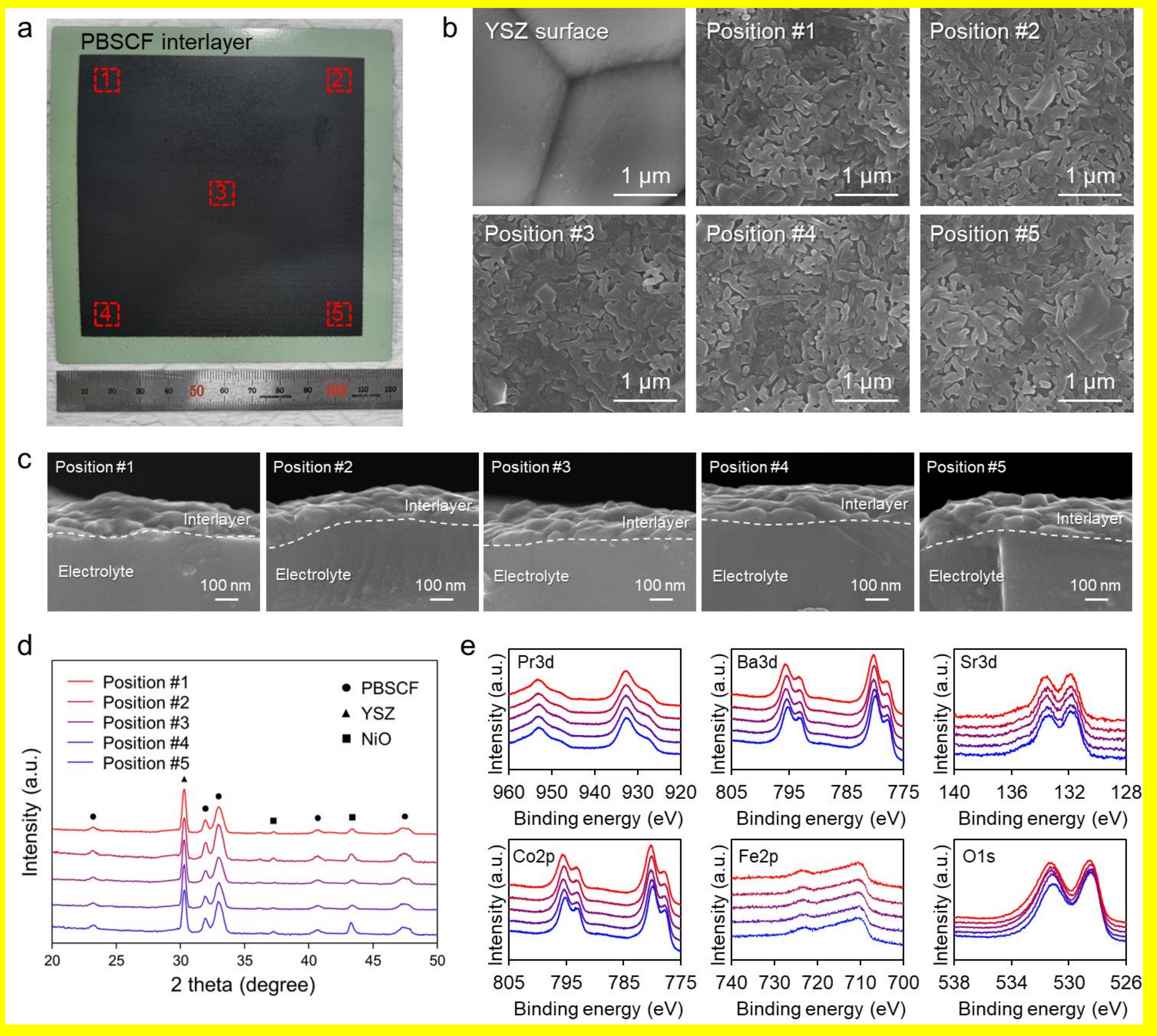

Supplementary Fig. 30. Structural and chemical uniformity of the PBSCF interlayer over an area of 100 cm². (a) Optical image of the PBSCF interlayer. The red dashed boxes indicate the five representative locations selected for the subsequent SEM, XRD, and XPS analyses. (b) Surface SEM images of the YSZ substrate and the PBSCF interlayer. (c) Cross-sectional SEM images of the PBSCF interlayer. (d) XRD patterns of the PBSCF interlayer. (e) XPS spectra of Pr 3d, Ba 3d, Sr 3d, Co 2p, Fe 2p, and O 1s acquired at the PBSCF interlayer. Panels (d) and (e) share the same legend.

1-3. Method comparison and film formation mechanism

In addition, there is no clue why this method is better than other methods. The method's operating principles are unclear. Why can the precursor deposition prepare dense thin films?

We agree that the operating principle of the precursor-based ESD process and its distinction from other interlayer fabrication methods were not sufficiently explained in the original manuscript. We have therefore revised the manuscript and Supplementary Information to clarify the deposition mechanism, the origin of the thin and dense interlayer, and the practical reasons for selecting this method. We have also added a qualitative comparison with representative interlayer fabrication techniques.

Electrostatic spray deposition (ESD) is based on the electrohydrodynamic atomization of a liquid feedstock under an applied electric field. A stable Taylor cone forms at the nozzle tip and continuously emits charged droplets. As the droplets travel toward the heated substrate, solvent evaporation reduces their size and increases the solute concentration within each droplet. Further solvent removal after the droplets reach the heated substrate forms a precursor-derived deposit on the electrolyte surface. A schematic illustration of the process has been added in Fig. S4. An important distinction of the present process is the use of a homogeneous solution containing dissolved metal precursors rather than a suspension of preformed ceramic particles. In powder-based deposition, the formation of a continuous nanoscale layer is constrained by the size and aggregation of the suspended particles. In contrast, the precursor-based ESD delivers dissolved metal species to the substrate, which are subsequently converted into a crystalline oxide layer through thermal decomposition and crystallization during sintering. The resulting interlayer thickness is therefore not directly limited by the particle size of the

feedstock. This facilitates the uniform distribution of the deposited species over the electrolyte surface and the formation of a continuous interlayer at a nanoscale thickness that are difficult to achieve using powder-based coating methods.

The formation of the thin and dense interlayer results from the combined control of substrate temperature, spray duration, and subsequent sintering. The substrate temperature governs droplet evaporation and solute redistribution during deposition. At low substrate temperatures, the droplets retain substantial solvent upon reaching the substrate, resulting in droplet coalescence and agglomerated deposits. With increasing substrate temperature, enhanced solvent evaporation induces lateral capillary flow within the droplets, transporting the solute toward the droplet edges and producing non-uniform ring-like features. When the substrate temperature is sufficiently high for the solvent to evaporate completely before the droplets reach the substrate, lateral solute migration is minimized, resulting in finer and more uniformly distributed precursor deposits. Subsequent sintering decomposes the nitrate precursors and crystallizes the uniformly distributed deposits into a continuous and dense PBSCF interlayer. The spray duration was then optimized to balance contact coverage and interlayer thickness. Insufficient deposition resulted in incomplete coverage, whereas excessive deposition increases the ion transport path by thickening the interlayer. Based on this process-structure relationship, the substrate temperature and spray duration were systematically optimized to obtain a thin, dense, and highly conformal interlayer (Figs. S5-S8).

We do not claim that precursor-based ESD is universally superior to all other deposition methods. Each technique offers distinct advantages depending on the target material, substrate geometry, and fabrication scale. For the present interface engineering strategy, however, an appropriate fabrication method should simultaneously provide a dense and conformal interlayer with a sufficiently small and controllable thickness to minimize additional ionic

transport resistance, accommodate complex oxide compositions, and support large area non-vacuum processing. Vacuum deposition methods such as pulsed laser deposition and sputtering provide precise thickness control, but generally require vacuum equipment and are less straightforward to apply to large areas or geometrically complex substrates. Atomic layer deposition offers excellent thickness control and conformality, but its materials range is constrained by the availability of suitable volatile precursors and requires a controlled reactor process. Screen printing and powder-based coating methods are readily scalable and compositionally flexible, but the fabrication of continuous and dense layers with sub-micrometer thickness is challenging due to the particle size and ink rheology. As summarized in Table S1, precursor-based ESD provides a practical combination of non-vacuum processing, molecular precursor feedstock, nanoscale thickness control, conformal coverage, compositional flexibility, and large area compatibility. These characteristics make precursor-based ESD particularly suitable for implementing the contact coverage-based interface engineering strategy in this work.

We have revised the manuscript and Supplementary Information as follows:

[line 15, page 4 in the revised manuscript]

To achieve these characteristics through effective and scalable nanoscale engineering, we employed ESD, which enables the precise control of nanoscale features by regulating atomized droplet dynamics through tailored process parameters (Supplementary Fig. 4).[14,21-23] In particular, a precursor-based ESD was employed as a favorable method for producing desired conformal, dense, and thin films due to its finer structural resolution over powder-based

ESD.[14,20,24] The characteristic of precursor-based ESD relevant to interlayer fabrication are compared with those of representative deposition methods in Supplementary Table 1. We optimized two key ESD process parameters of the substrate temperature and spray duration, as shown in Supplementary Figs. 5–8.

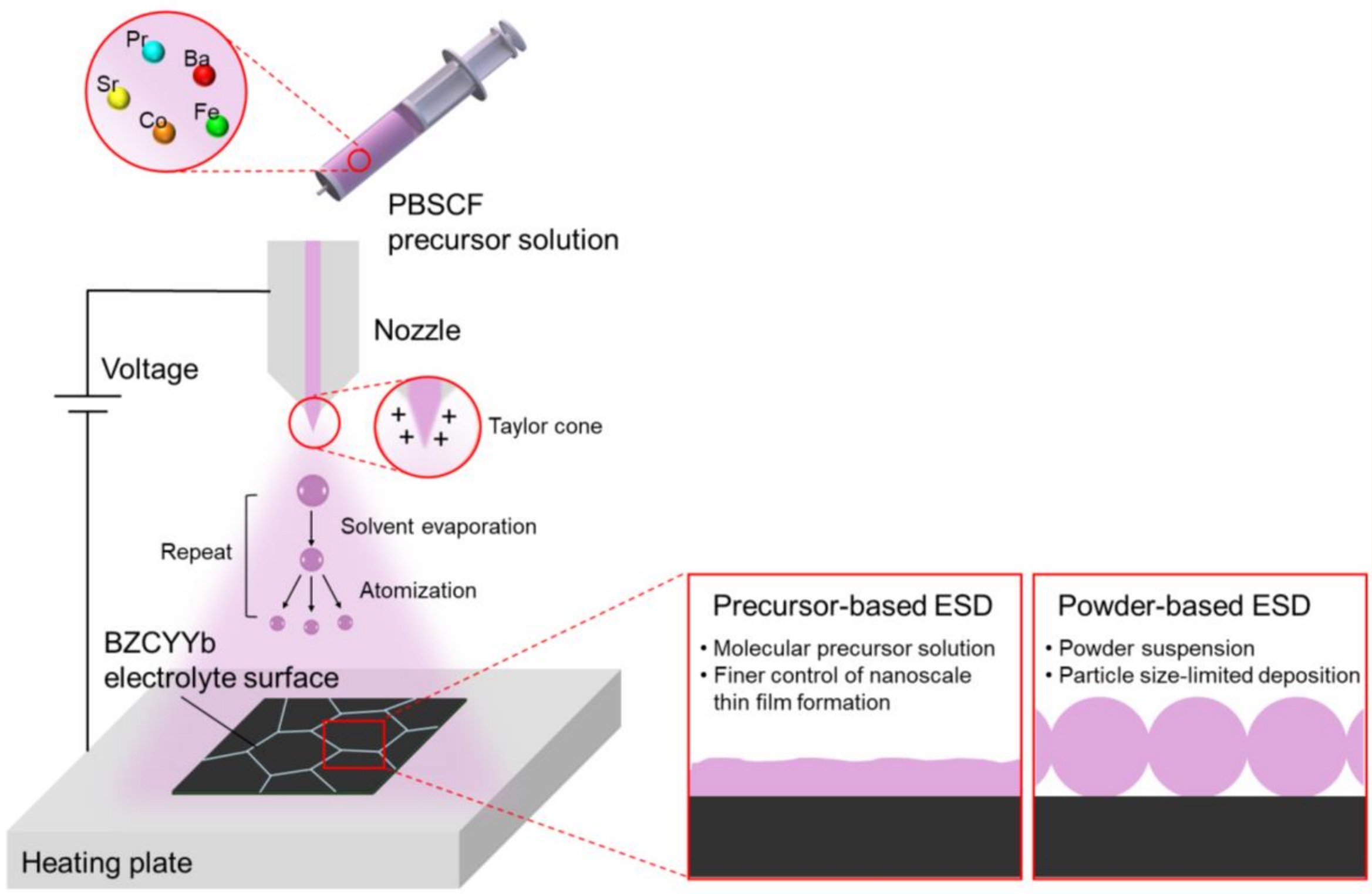

Supplementary Fig. 4. Operating principle of precursor-based electrostatic spray deposition.

Under an applied electric field, a liquid feedstock forms a stable Taylor cone forms at the nozzle tip and emitted as charged droplets. During flight toward the heated substrate, solvent evaporation reduces the droplet size and increases the solute concentration. Further solvent removal after the droplets reach the substrate forms a precursor-derived deposit on the electrolyte surface. In precursor-based ESD process, the feedstock is a homogeneous solution containing dissolved metal precursors rather than a suspension of preformed ceramic particles.

Subsequent sintering converts the deposited species into a continuous crystalline oxide interlayer through thermal decomposition and crystallization. This distinction is important because the deposited layer is not formed from preformed ceramic particles, and its nanoscale thickness is therefore not directly constrained by the particle size of the feedstock. This enables finer control over the formation of continuous nanoscale thin films.

Supplementary Table 1. Qualitative comparison of representative coating techniques based on characteristics relevant to interlayer fabrication.

Method	Feedstock	Vacuum	Area scalability	Thickness control	Conformality	Materials flexibility
Pulsed laser deposition	Ceramic target	Required	Limited to moderate	Excellent	Moderate	Moderate
Sputtering	Ceramic or metal target	Required	Moderate	Excellent	Limited to moderate	Moderate
Atomic layer deposition	Volatile chemical precursor	Required or controlled reactor	Moderate to high	Excellent	Excellent	Limited
Screen printing	Powder ink or paste	Not required	High	Limited below μm scale	Limited	High
Powder-based ESD	Powder suspension	Not required	High	Moderate	Moderate	High
Precursor-based ESD (This work)	Molecular precursor solution	Not required	High	High	High	High

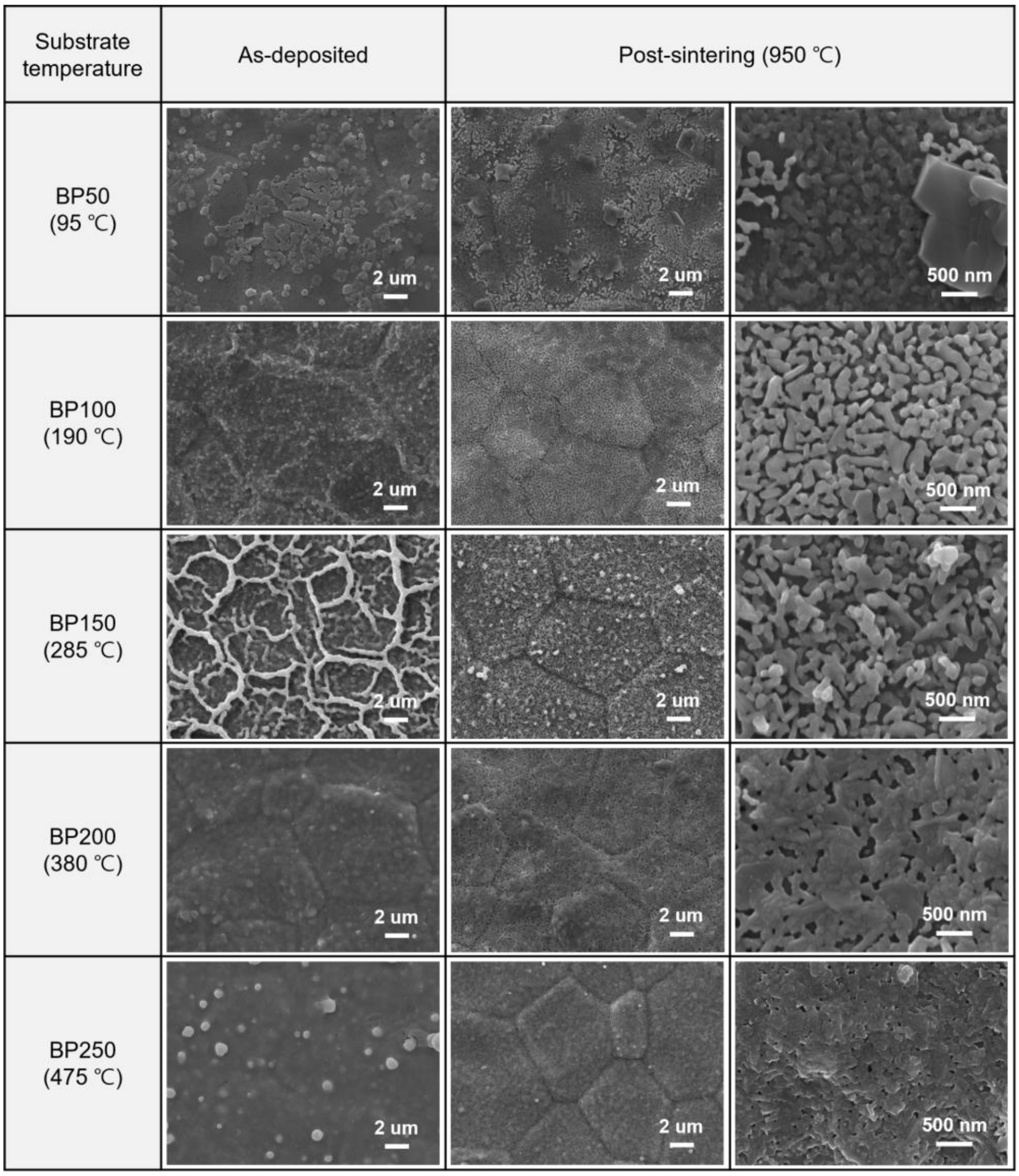

Supplementary Fig. 5. Structural characterization of the precursor-based interlayer was conducted at five substrate temperatures: BP50, BP100, BP150, BP200, and BP250, corresponding to 50%, 100%, 150%, 200%, and 250% of the solvent boiling point (190 ℃), respectively. Surface SEM images were analyzed to evaluate the interlayer morphology in both the as-deposited state and after sintering at 950 ℃. In droplet-based deposition processes, substrate temperature strongly affects solvent evaporation behavior and droplet size, thereby regulating capillary flow and the resulting film morphology. Five substrate temperatures (BP50, BP100, BP150, BP200, and BP250), corresponding to 50%, 100%, 150%, 200%, and 250% of the solvent boiling point (190 ℃), were investigated. At a substrate temperature of BP50

(95 °C), insufficient solvent evaporation resulted in partially agglomerated structures. Increasing the substrate temperature to BP100 (190 °C) and BP150 (285 °C) intensified capillary flow, driving solutes toward droplet edges and forming non-uniform ring-like structures. At the substrate temperatures of BP200 (380 °C) and BP250 (475 °C), the droplet size decreased below the critical threshold for capillary flow generation, resulting in a highly concentrated and relatively uniform deposition.

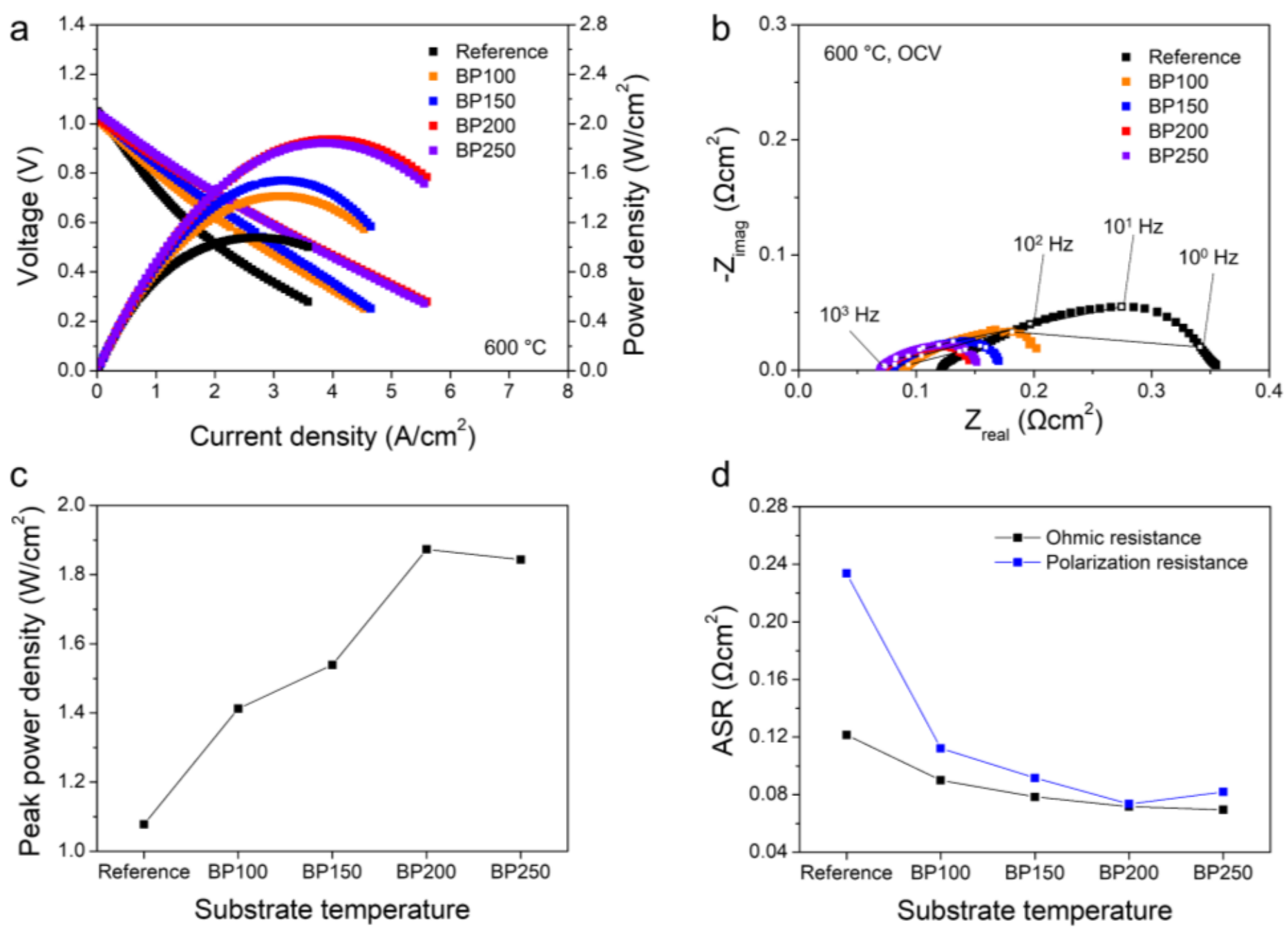

Supplementary Fig. 6. (a) I–V–P curves and (b) Nyquist plots of the reference and interlayer-deposited cells at four substrate temperatures. (c) Peak power density and (d) ohmic and polarization resistances of the corresponding cells measured at 600 °C. The peak power density reached its maximum, while both ohmic and polarization resistances were minimized, as the substrate temperature increase up to BP200 (380 °C), and then became saturated at higher temperature.

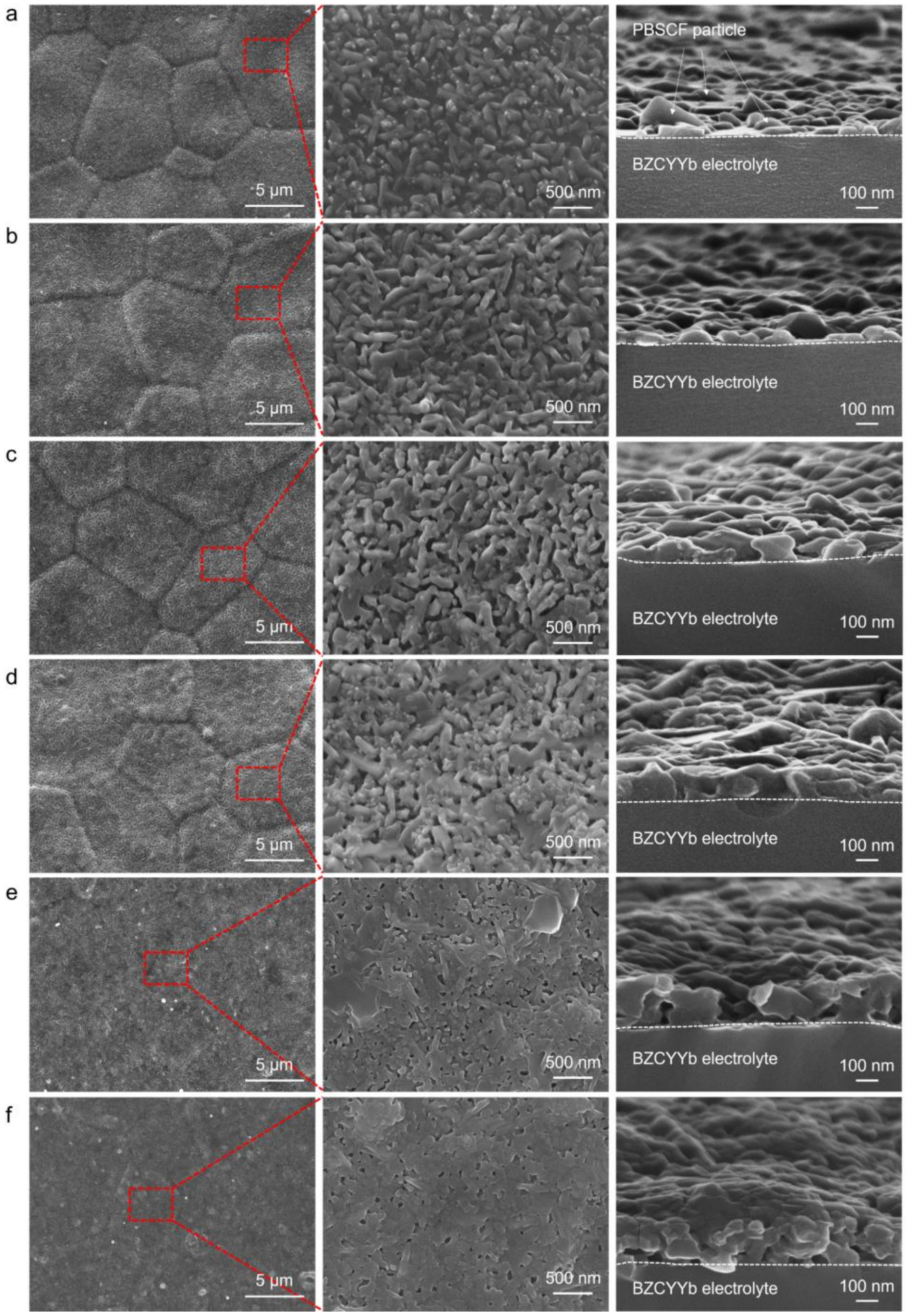

Supplementary Fig. 7. Structural characterization of the precursor-based interlayer deposited with varying spray durations. Surface and tilted SEM images for spray durations of (a) 4 h, (b) 6 h, (c) 8 h, (d) 10 h, (e) 16 h, and (f) 24 h. The coverage on the BZCYYb electrolyte surface increased with longer spray duration from 4 to 8 h and became saturated with longer spray duration, while the interlayer thickness continuously increased up to 24 h. (~150 nm for 8 h and ~400 nm for 24 h).

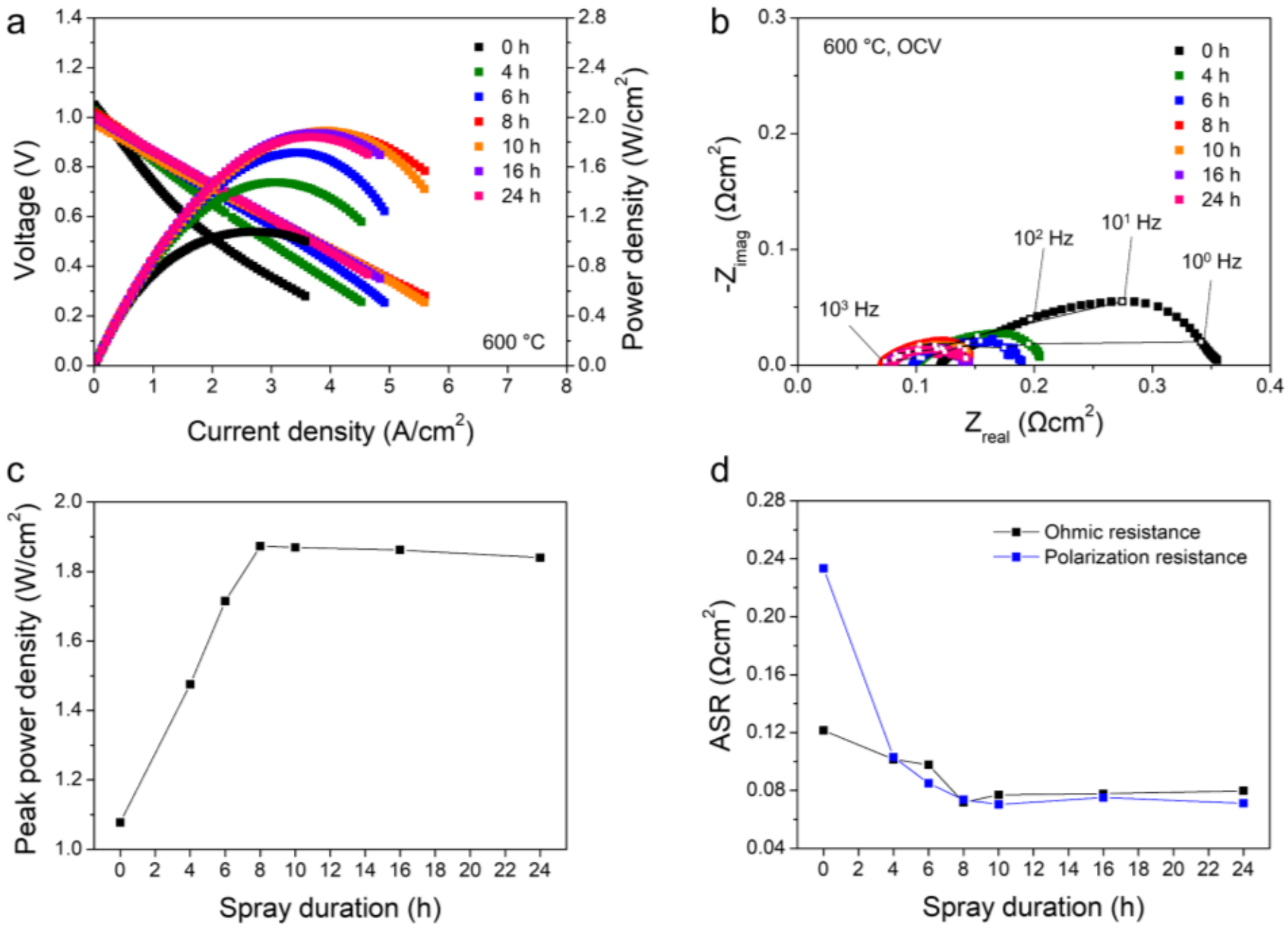

Supplementary Fig. 8. (a) I–V–P curves and (b) Nyquist plots of the reference and interlayer-deposited cells at different spray durations. (c) Peak power density and (d) ohmic and polarization resistances of the corresponding cells measured at 600 °C. Electrochemical analyses showed a concurrent increase in peak power density and a decrease in both ohmic and polarization resistances with increasing spray duration up to 8 h. Beyond 8 h, further thickening of the interlayer led to a slight increase in the ohmic resistance, which we attribute to an increased ion diffusion length through a thicker interlayer. Accordingly, 8 h was selected as the optimal spray duration.

1-4. Applicability to other electrode materials

The method is electrostatic deposition. It is not clear whether the method is suitable for other material systems.

In precursor-based ESD, an applied electric field atomizes a precursor solution into charged droplets and transports them toward the heated substrate. Film formation proceeds through solvent evaporation during deposition, followed by thermal decomposition and crystallization during subsequent sintering. Because the process depends primarily on stable electrohydrodynamic atomization of the precursor solution and the thermal conversion of the deposited species, it is not intrinsically restricted to a specific oxide composition. Its application, however, requires that the constituent cations can be formulated into a stable precursor solution capable of sustaining an electrospray and that the deposited species can be converted into the desired crystalline phase under compatible heat treatment conditions. Therefore, the deposition and heat treatment conditions may need to be adjusted according to the precursor chemistry and target material.

To address this point, we have performed additional experiments using three representative air electrode materials with distinct characteristics: $Gd_{0.3}Ca_{2.7}Co_{3.82}Cu_{0.18}O_{9-\delta}$ (GCCCO), a triple conducting oxide (TCO), and $Ba_{0.5}Sr_{0.5}Co_{0.8}Fe_{0.2}O_{3-\delta}$ (BSCF), a mixed ionic and electronic conductor (MIEC), were examined in protonic ceramic fuel cells with a Ni-BZCYYb/BZCYYb/air electrode configuration. $La_{0.6}Sr_{0.4}CoO_{3-\delta}$ (LSC), another MIEC, was examined in a conventional solid oxide fuel cell with a Ni-YSZ/YSZ/GDC/air electrode configuration [R1-R3]. For each composition, an interlayer was fabricated using the corresponding precursor solution. Thin, dense, and conformal layers with surface morphologies comparable to that of the PBSCF interlayer were obtained in all three systems (Fig. S31). These results demonstrate that precursor-based ESD is not limited to the PBSCF system and can be extended to multiple oxide compositions and cell architectures.

Electrochemical measurements provided further functional validation of the fabricated interlayers. The introduction of the interlayer increased the peak power density and reduced

both the ohmic and polarization resistances in all tested systems (Fig. S32). In particular, comparable reduction rates in the ohmic resistance were observed for all electrode materials, indicating that improved interfacial contact coverage consistently reduces ohmic resistance regardless of the electrode material and cell architecture (Fig. R1a). The polarization resistance also decreased for all electrode materials, although the reduction rate varied depending on intrinsic reaction kinetics and transport properties (Fig. R1b). These results demonstrate that the proposed interlayer strategy is not limited to a specific electrode material, but can be extended to different electrode materials, supporting the broader applicability of the proposed interface engineering approach.

We have revised the related description in the manuscript and Supplementary Information as follows:

[line 8, page 11 in the revised manuscript]

To examine whether the interface-engineering strategy can be extended to other electrode materials, the precursor-based interlayers were fabricated using additional air electrode materials, including $Gd_{0.3}Ca_{2.7}Co_{3.82}Cu_{0.18}O_{9-\delta}$ (GCCCO)[9], $Ba_{0.5}Sr_{0.5}Co_{0.8}Fe_{0.2}O_{3-\delta}$ (BSCF)[44], and $La_{0.6}Sr_{0.4}CoO_{3-\delta}$ (LSC)[45] (Supplementary Figs. 31 and 32). The resulting interlayers exhibited morphologies comparable to that of the PBSCF interlayer, and their introduction consistently improved the electrochemical performance in all systems. These results indicate that the interlayer strategy is not limited to a specific electrode material and support its broad applicability.

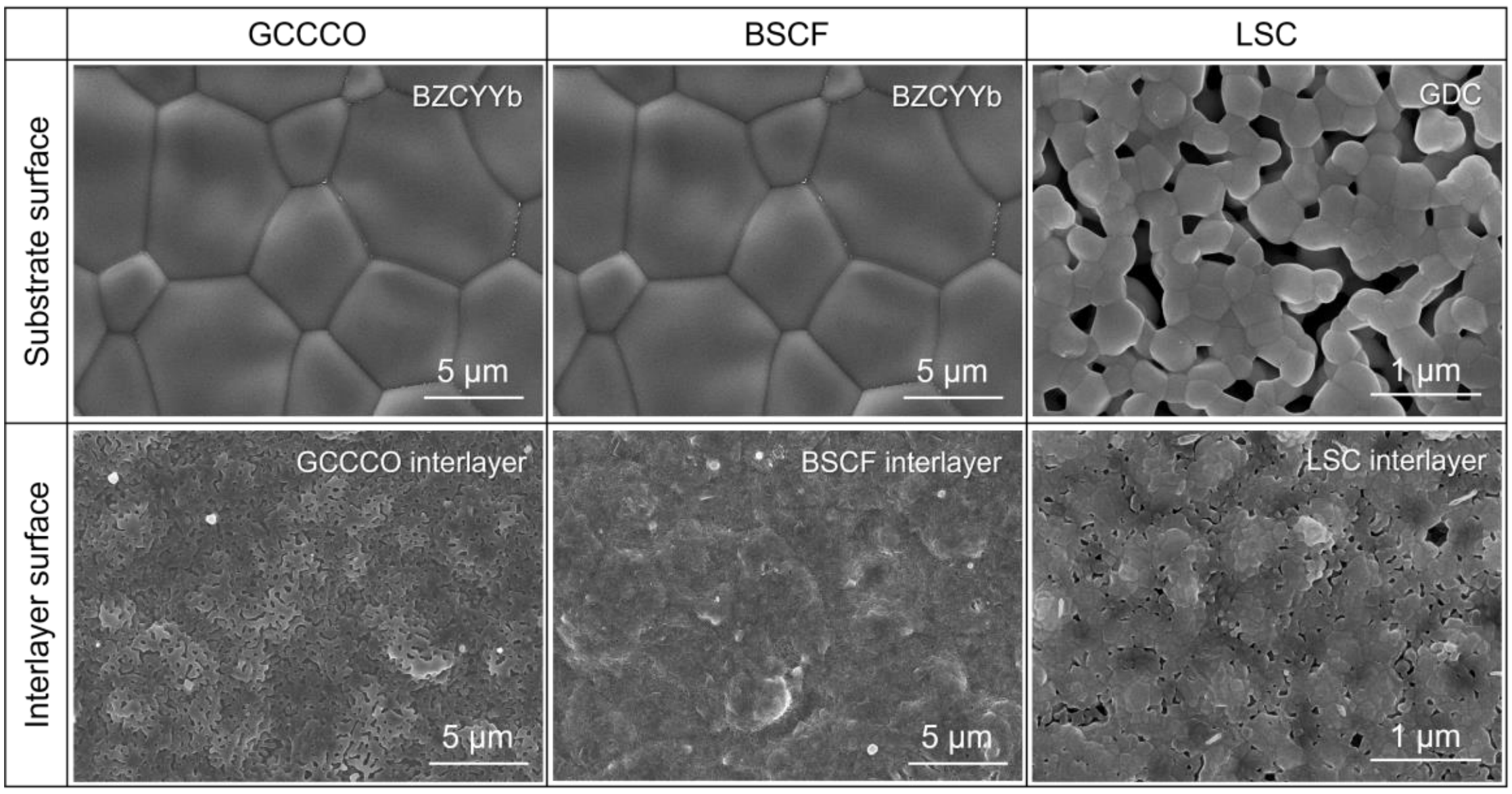

Supplementary Fig. 31. Surface SEM images of the substrates and the precursor-based interlayers.

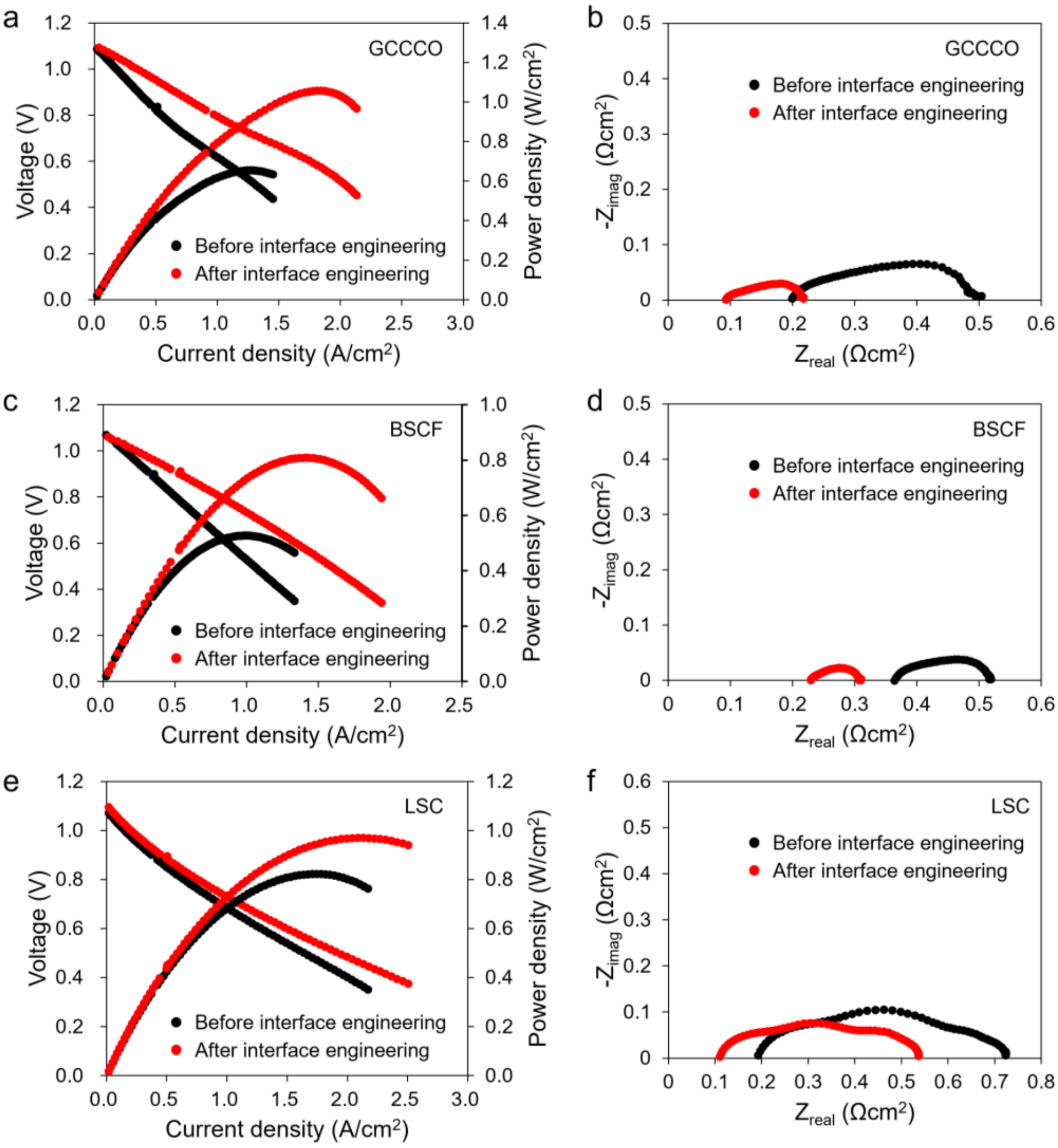

Supplementary Fig. 32. (a) I-V-P curves of the GCCCO interlayer in a Ni-BZCYYb/BZCYYb/GCCCO configuration at 600 °C and (b) Nyquist plots under OCV conditions. (c) I-V-P curves of the BSCF interlayer in a Ni-BZCYYb/BZCYYb/BSCF configuration at 600 °C and (d) Nyquist plots under OCV conditions. (e) I-V-P curves of the LSC interlayer in a Ni-YSZ/YSZ/GDC/LSC configuration at 650 °C and (f) Nyquist plots under OCV conditions.

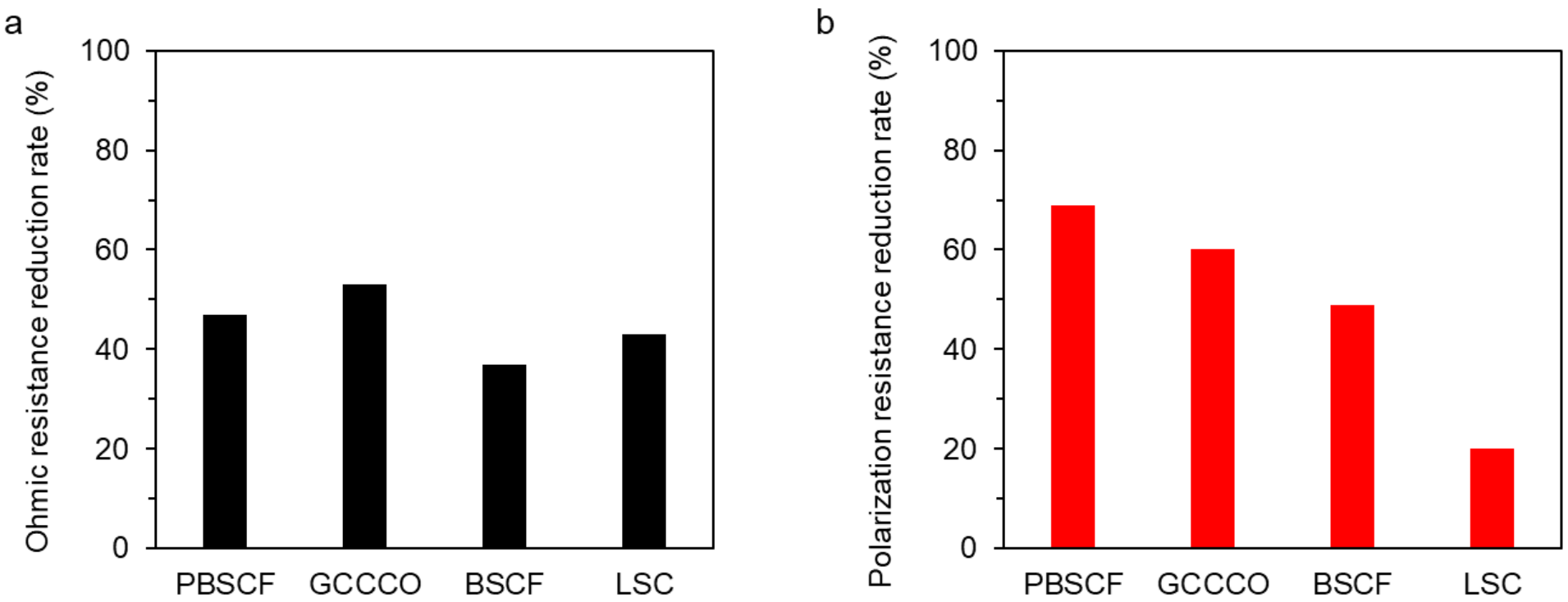

Fig. R1. Reduction rates of (a) ohmic and (b) polarization resistances upon introduction of the interlayer for various electrode materials.

[R1] Saqib, M. et al. Transition from perovskite to misfit-layered structure materials: a highly oxygen-deficient and stable oxygen electrode catalyst. *Energy Environ. Sci.* **14**, 2472–2484 (2021).

[R2] Liu, Z. et al. Strategic atomic trapping at heterointerfaces for protonic ceramic cells. *Nat. Commun.* **16**, 10405 (2025).

[R3] Rehman, S. U. et al. Designing the nano-scale architecture of the air electrode for high-performance and robust reversible solid oxide cells. *Appl. Catal. B Environ. Energy* **333**, 122784 (2023).

2. The authors claim that the quantitively correlated the coverage to specific resistances, which has been mentioned in their previous AEM paper. In addition, the linear relationships between coverage and the interfacial ohmic resistance and the partial polarization resistance were not explained well. It is not clear what the physics are there. Why is the slope 1 rather than some

other value? Based on the description, the interface is a dense film; it is unclear whether the interlayer's triple ionic and electronic conductivities and the bulk electrode's conductivities affect the results and fitting conclusions. For example, the authors claim that the number of active sites increased upon interlayer introduction. Will the conclusions be different with an electrode with a different partial conductivity? Therefore, the claimed quantitative correlation needs a deeper understanding. The partial conductivity effect should be included. Will the different tripe conductive electrodes show the same linear relationship and the same fitted slope values? In addition, the low-frequency peak of DRT was related to coverage and attributed to surface reaction sites. More physical explanations of the reaction sites at the interface and the bulk surface should be provided.

[REPLY REDACTED]
